# Expedient Synthesis of Alkyl and Aryl Thioethers Using Xanthates as Thiol-Free Reagents

**DOI:** 10.3390/molecules29112485

**Published:** 2024-05-24

**Authors:** Jinli Nie, Ziqing He, Sijie Xie, Yibiao Li, Runfa He, Lu Chen, Xiai Luo

**Affiliations:** 1 Jiangmen Key Laboratory of Synthetic Chemistry and Cleaner Production, School of Environmental & Chemical Engineering, Wuyi University, Jiangmen 529020, China; wyuchemnjl@126.com (J.N.); wyuchemhzq@126.com (Z.H.); wyuchemxsj@126.com (S.X.); wyuchemhrf@126.com (R.H.); wyuchemcl@126.com (L.C.); 2 Hunan Province Key Laboratory for Synthetic Biology of Traditional Chinese Medicine, School of Pharmaceutical Sciences, Hunan University of Medicine, Huaihua 418000, China

**Keywords:** thioethers, xanthate, sulfidation, odorless

## Abstract

Thioethers are critical in the fields of pharmaceuticals and organic synthesis, but most of the methods for synthesis alkyl thioethers employ foul-smelling thiols as starting materials or generate them as by-products. Additionally, most thiols are air-sensitive and are easily oxidized to produce disulfides under atmospheric conditions; thus, a novel method for synthesizing thioethers is necessary. This paper reports a simple, effective, green method for synthesizing dialkyl or alkyl aryl thioether derivatives using odorless, stable, low-cost ROCS_2_K as a thiol surrogate. This transformation offers a broad substrate scope and good functional group tolerance with excellent selectivity. The reaction likely proceeds via xanthate intermediates, which can be readily generated via the nucleophilic substitution of alkyl halides or aryl halides with ROCS_2_K under transition-metal-free and base-free conditions.

## 1. Introduction

The development of efficient, sustainable methods for synthesizing thioethers has attracted increasing attention due to the importance of these compounds in the fields of fine chemicals, pharmaceuticals, functional materials, and organic synthesis. Among the most studied S-containing compounds, alkyl thioether derivatives have attracted considerable interest owing to their biological potential, and have been exploited in developing drugs, such as Griseoviridin [1], Viracept [2], Montelukast sodium [3], and Cilastatin [4]. Therefore, the direct synthesis of alkyl thioethers is an active area of research. Most of the strategies used in synthesizing alkyl thioethers employ thiols as starting materials or involve the addition of an organometallic reagent to a disulfide [5,6]. The disadvantage of these methods is the use of malodorous thiols as starting materials or the production of thiols as by-products. Most thiols are air-sensitive and readily oxidized to produce disulfides under aerobic atmospheric conditions. To overcome these limitations, the attractive alternative methods for the sulfuration of alkyl halides and aryl halides involve the use of other sulfurizing agents, e.g., S powder [7,8,9,10], bunte salts [11,12,13], dimethyl sulfoxide (DMSO) [14,15], carenesulfonyl cyanides [16], S-methylisothiourea [17], N-substituted sulfanylsuccinimides [18,19], disulfides [20,21,22], sulfonyl chlorides [23,24], sodium sulfinates [25,26,27], and sulfonyl hydrazides [28,29], because these off-the-shelf thiol-free sulfurizing agents generally release little to no odors.

**Scheme 1:**
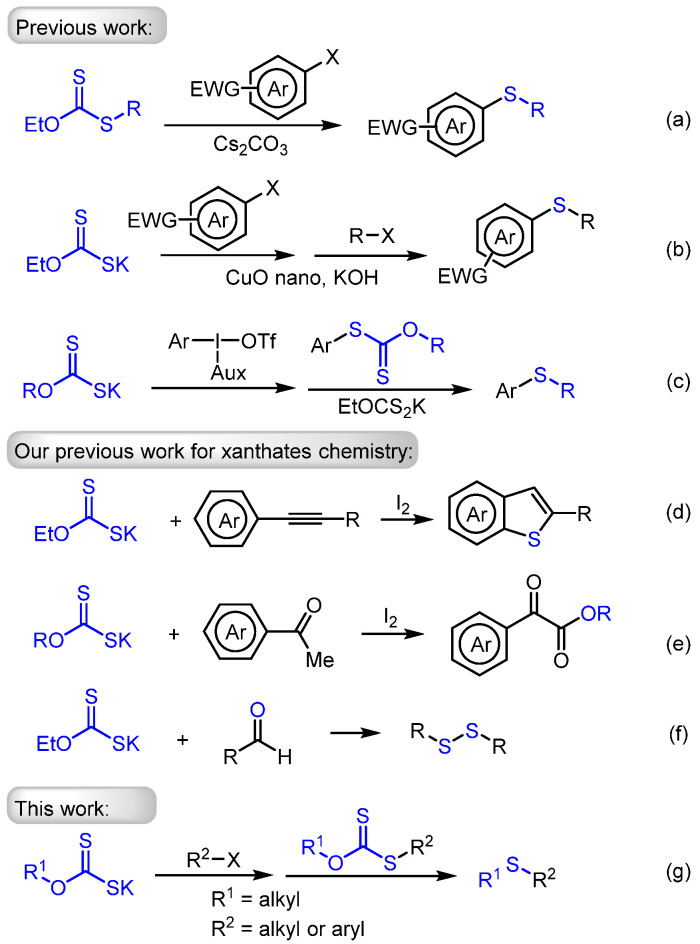
Methods of sulfide synthesis using xanthates (**a**–**c**) and xanthate chemistry in our laboratory (**d**–**g**). EWG, electron-withdrawing group; OTf, triflate.

Xanthates are attractive sulfurizing agents used in both transition-metal-catalyzed and transition-metal-free transformations because they are odorless and have low toxicities and can be readily prepared on large scales using low-cost alcohols and CS_2_ [30,31,32,33]. The addition of alkyl halides and aryl halides to xanthates may provide a general route for preparing thioethers without using odorous thiol starting materials. Baranov [34] and Kakulapati [35] reported the use of xanthates as thiol surrogates in nucleophilic substitution or cross-coupling with aryl halides in synthesizing aryl thioethers (Scheme 1a,b). Karchava et al. described the visible-light-driven S-arylation of EtOCS_2_K (Et = ethyl) using aryl halides in synthesizing aryl thioethers [36], followed by the reaction of diaryliodonium salts with xanthate salts to prepare the corresponding alkyl aryl thioether compounds (Scheme 1c) [37]. Nevertheless, xanthates, which are generally used as thiol surrogates, react with aryl halides to generate aryl thioethers, but the synthesis of dialkyl thioethers using xanthates has rarely been reported. To the best of our knowledge, the sole attempt to directly transform xanthates into dialkyl thioethers was reported by Degani et al. They obtained only small amounts of the desired dialkyl thioethers via the sulfurization of alkyl halides using EtOCS_2_K as a sulfurizing agent [38]. Based on our research on the development of xanthate chemistry (Scheme 1d–f) [39,40,41], we herein report a facile approach for use in generating various dialkyl thioethers and aryl thioethers. This approach involves the sulfuration of alkyl halides and aryl halides using ROCS_2_K as a thiol-free sulfurizing and alkylating reagent (Scheme 1g).

## 2. Results and Discussion

To evaluate this synthesis of dialkyl thioethers hypothesis, we screened the reaction conditions using 4-(chloromethyl)biphenyl (**1a**), EtOCS_2_K (**2a**), and dimethylformamide (DMF) as a model reaction. Gratifyingly, sulfidation proceeds at a reaction temperature of 150 °C to afford the dialkyl thioether **3a** in 77% yield (Table 1, entry 1). The screening of various solvents reveals that the solvent is critical in the sulfidation reaction (Table 1, entries 1–5), and a trace amount of the thioether **3a** is obtained when non-polar solvent o-xylene is used (Table 1, entry 2). The optimal results are obtained when the reaction is conducted in DMSO at 150 °C (Table 1, entry 3), whereas unsatisfactory results are obtained using dimethylacetamide (DMAc) or N-methyl-2-pyrrolidone (NMP) as the solvent (Table 1, entries 4–5). The yield of the thioether **3a** does not change significantly when the amount of DMSO is decreased (Table 1, entry 6). Notably, the yield decreases significantly when the dosage of **2a** is decreased (Table 1, entry 7). Further studies indicate that decreasing the reaction temperature does not decrease the yield of sulfidation (Table 1, entries 8 and 9). However, when the reaction temperature is further decreased to 80 °C, the yield of the sulfidation product **3a** decreases slightly (89%; Table 1, entry 10). Subsequently, we investigated the reaction time, and thus, the yield of sulfidation is unaffected when the reaction time is shortened to 1 h, but shortening the time further to 0.5 h affords a significant decrease in yield (Table 1, entries 11–13). Based on these results, the optimized reaction conditions are **1a** (0.5 mmol) and **2a** (1.0 mmol) in 1.0 mL DMSO at 100 °C for 1 h (Table 1, entry 12).

**Scheme 2:**
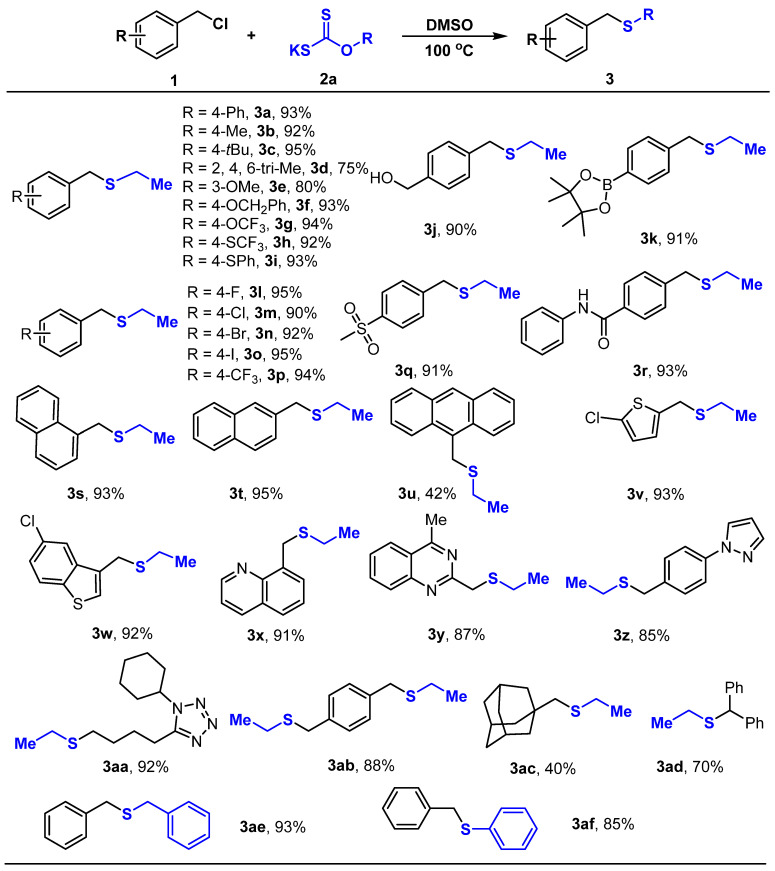
Synthesis of thioethers using various alkyl chlorides ^a,b^. ^a^ Reaction conditions: alkyl halide (0.5 mmol), ROCS_2_K (1.0 mmol) in DMSO (1.0 mL) at 100 °C in a sealed tube in an air atmosphere for 1 h ^b^ isolated yields.

With the optimized conditions for use in synthesizing dialkyl thioethers established, the alkyl halides applicable in the sulfuration reaction were investigated (Scheme 2). Firstly, various substituted benzyl chlorides are compatible under the optimized conditions. Aromatic rings with electron-donating and electron-withdrawing substituents are compatible under the standard conditions. Electron-donating groups, such as –Me, –*^t^*Bu, –TMS, –OMe, –OCH_2_Ph, –OCF_3_, –SCF_3_, –SPh, –CH_2_OH, and –BPin_2_ (Me = methyl, *^t^*Bu = *tert*-butyl, TMS = trimethylsilyl, Ph = phenyl, Pin = pinacol), are successfully sulfated to produce dialkyl thioethers in good yields (Scheme 2, **3a**–**3k**). Hindered 2-(chloromethyl)-1,3,5-trimethylbenzene, in particular, successfully undergoes the reaction, affording ethyl(2,4,6-trimethylbenzyl)sulfane **3d** in 75% yield. Remarkably, the –BPin_2_ group remains intact on the aromatic ring in 91% yield and is very useful in transition-metal-catalyzed cross-coupling reactions (Scheme 2, **3k**). A crucial feature of this reaction is its tolerance of various halides, including –F, –Cl, –Br, –I, and –CF_3_, with no dehalogenated by-products observed (Scheme 2, **3l**–**3p**). Additionally, benzyl chlorides substituted with strong electron-withdrawing groups, such as sulfone and amide, successfully undergo the reaction, furnishing thioethers **3q** and **3r** in yields of 91% and 93%, respectively. Moreover, fused-ring and heterocyclic-substituted alkyl halides, such as naphthalene (**3s**–**3t**), anthracene (**3u**), thiophene (**3v**), benzothiophene (**3w**), quinolone (**3x**), quinazoline (**3y**), pyrazole (**3z**), and tetrazole (**3aa**), can undergo the reaction to produce the desired products in moderate-to-good yields. Next, 1,4-bis(chloromethyl)benzene, 1-(chloromethyl)adamantane, and (chloromethylene)dibenzene successfully undergo the reaction, indicating that the sulfuration reaction is characterized by a good functional group tolerance (Scheme 2, **3ab**–**3ad**). Finally, the use of aromatic xanthates as substituents was investigated. Gratifyingly, benzyl and phenyl substituent xanthates were well tolerated under the optimized conditions, affording the corresponding thioethers in good yields (Scheme 2, **3ae**–**3af**).

To evaluate this synthesis of aryl alkyl thioethers’ hypothesis, we screened the reaction conditions using 3-iodopyridine (**1ae**), EtOCS_2_K (**2a**), additive, and DMF as a model reaction. Initially, sulfidation proceeds at a reaction temperature of 150 °C for 24 h to afford the 3-(ethylthio)pyridine **4a** in 37% yield (Table 2, entry 1). The screening of various reaction times reveals that the reaction time is critical in the sulfidation reaction, and excellent results are obtained when the sulfidation reaction was carried out in 36 h (Table 2, entries 1–3). Notably, the yield decreases significantly when the dosage of EtOCS_2_K (**2a**) or I_2_ is decreased (Table 2, entries 4–5). With EtOCS_2_K (**2a**) as the sulfur source, the examination of different additives showed that NH_4_I and HI was inefficient (Table 2, entries 6–7). Further optimum solvents showed that DMF was the best choice; the other solvents—DMSO, NMP, and DMAc—all decreased the yield of **4a** (Table 2, entries 8–10). Furthermore, decreasing the reaction temperature led to a decrease in yield (Table 2, entry 11). Without the use of an iodine reagent, only a trace of the sulfidation reaction product was obtained; mostly the starting material was recovered (Table 2, entry 12). Based on these results, the optimized reaction conditions are halopyridine (0.5 mmol) and EtOCS_2_K (1.2 mmol) and I_2_ (1.5 mmol) in 3.0 mL DMF at 150 °C for 36 h (Table 1, entry 3).

**Scheme 3:**
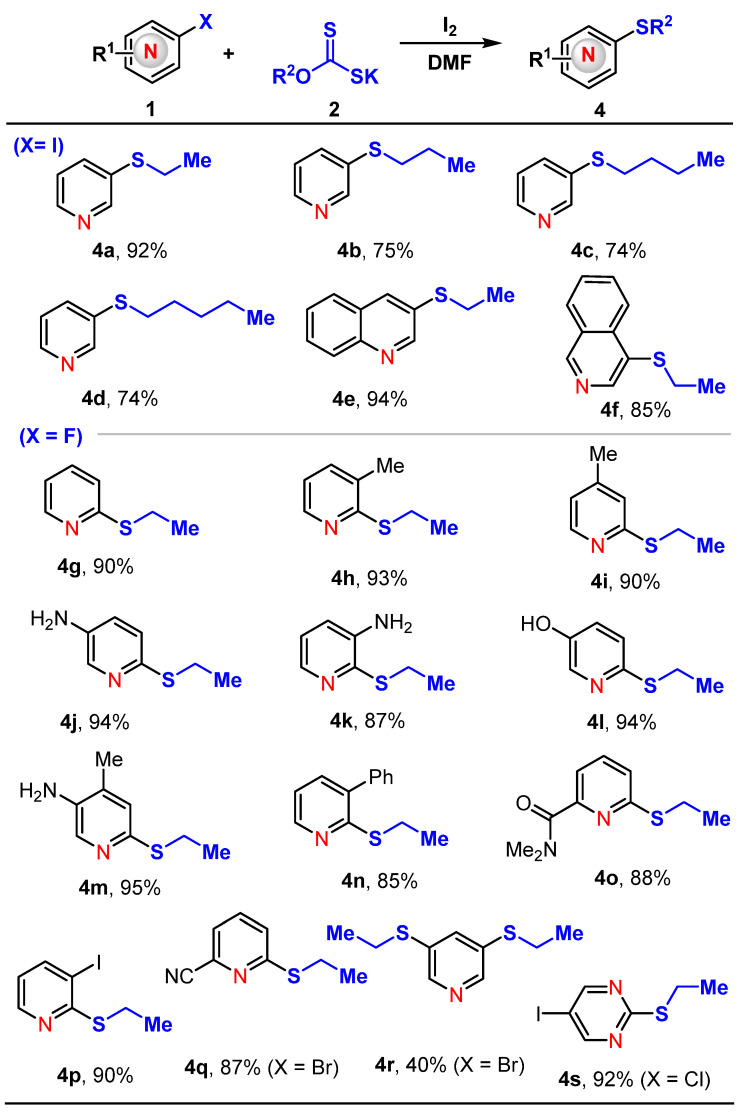
Substrate scope of the sulfuration reaction ^a,b^. ^a^ Reaction conditions: pyridyl halide **1** (0.5 mmol), ROCS_2_K **2** (1.2 mmol), and I_2_ (1.5 mmol) in DMF (3.0 mL) at 150 °C for 36 h in a sealed tube in an air atmosphere. ^b^ Isolated yields.

The iodopyridine reactions with various substituted potassium xanthates also proceed with smooth conversions under the optimized conditions, furnishing the corresponding thioethers in moderate-to-good yields (Scheme 3). Notably, substituted potassium xanthates with ethyl (**4a**), *n*-propyl (**4b**), *n*-butyl (**4c**), and *n*-pentyl groups (**4d**) are tolerated well under mild reaction conditions. When sulfuration is conducted using 3-iodoquinoline and 4-iodoisoquinoline, the thioether products **4e**–**4f** are obtained in yields of 94% and 85%, respectively. 2-Fluoropyridines bearing various functional groups are completely converted in the presence of **2a** to furnish the corresponding sulfides in good yields. 2-Fluoropyridines, substituted with both electron-donating and electron-withdrawing groups, react with **2a** to generate the corresponding sulfuration products **4g**–**4o** in good yields. The reaction tolerates various substituents, including –Me, –Ph, –NH_2_, –OH, –I, –OCNMe_2_, and –CN groups, and whether the substituent is at the 3-, 4-, 5-, or 6-position of the pyridine ring does not affect the yield of the reaction. When 2-fluoro-3-iodopyridine is used as the starting material, the F atom at the 2-position of the pyridine ring exhibits a higher reactivity, and the reaction affords the 2-(ethylthio)-3-iodopyridine product (**4p**) in 90% yield. Remarkably, when 3,5-dibromopyridine is used, the disulfuration product **4r** is obtained in 40% yield. The activities of the halogen atoms depend more on their positions when 2-chloro-5-iodopyrimidine is used as the starting material, affording product **4s** in 92% yield.

**Scheme 4:**
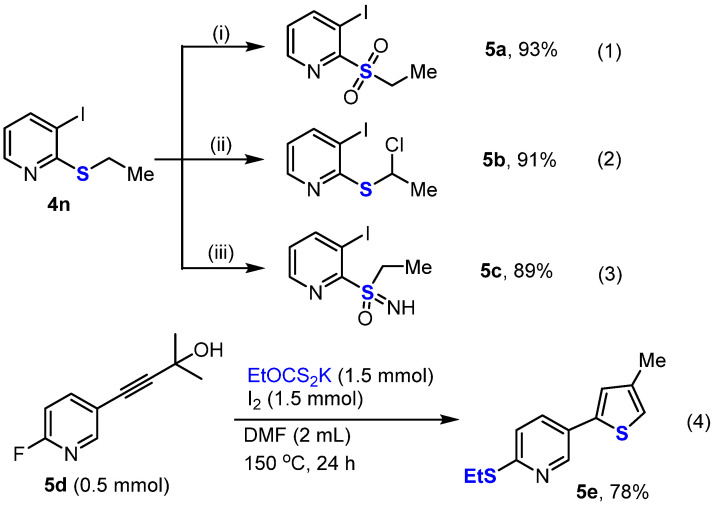
Synthetic utilization. Reaction conditions: (i) **4n** (0.5 mmol), *m*-CPBA (2.0 equiv.), dichloromethane (DCM, 3 mL), room temperature (r.t.), 8 h; (ii) **4n** (0.5 mmol), NCS (1.2 equiv.), DCM (3 mL), r.t. 8 h; (iii) **4n** (0.5 mmol), PIDA (2.3 equiv.), (NH_4_)_2_CO_3_ (1.5 equiv.), methanol (3 mL), r.t., 8 h.

To explore the synthetic applicability of the sulfuration reaction, the newly formed thioethers were utilized in various synthetic transformations (Scheme 4). First, 2-(ethylsulfonyl)-3-iodopyridine (**5a**) may be generated in 93% yield via *m*-chloroperoxybenzoic acid (*m*-CPBA) oxidation (Equation (1)). Furthermore, **4n** may be smoothly converted via NCS-promoted chlorination to 2-((1-chloroethyl)thio)-3-iodopyridine (**5b**) in 91% yield (Equation (2)) [42]. Remarkably, the thioether **4n** reacts successfully with (diacetoxyiodo)benzene (PIDA) and (NH_4_)_2_CO_3_ to produce sulfoximine **5c**, which has gained considerable attention owing to its unique structure and applications in medicinal chemistry, in 89% yield (Equation (3)) [43]. Finally, in the presence of I_2_ and **2a**, a novel, efficient protocol affords the substituted thiophene **5e** in 78% yield via the sulfidation and sulfur cyclization of 4-(6-fluoropyridin-3-yl)-2-methylbut-3-yn-2-ol **5d** with **2a** (Equation (4)) [44].

**Scheme 5:**
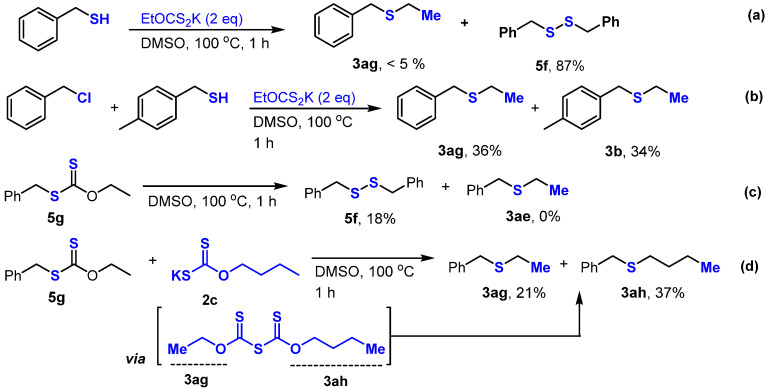
Control experiments for synthesis of dialkyl thioethers. (**a**) thioetherification of thiols; (**b**) cross thioetherification of benzyl chlorides with thiols; (**c**) hydrolysis and thioetherification of xanthate **5g**; (**d**) cross thioetherification of xanthate **5g** with potassium butylxanthate **2c**.

To enhance our understanding of the reaction mechanism, we designed control experiments, as shown in Scheme 5. First, the reaction of phenylmethanethiol with **2a** furnishes thioether **3ag** in trace amounts and 1,2-dibenzyldisulfane **5f** in 87% yield, suggesting that thiol alone cannot undergo the sulfation reaction with **2a** (Scheme 5a). When benzyl chloride and *p*-tolylmethanethiol are mixed as substrates, the results of gas chromatography–mass spectrometry reveal that the thioether products **3ag** and **3b** are obtained in yields of 36% and 33%, respectively. This suggests that xanthate **5g** generated via the nucleophilic substitution of benzyl chloride with **2a** may be the reaction intermediate (Scheme 5b–d). However, when directly using *S*-benzyl *O*-ethyl carbonodithioate **5g** to complete the reaction in the absence of **2a**, the expected thioether **3ae** is not produced, and a small amount of the 1,2-dibenzyldisulfane **5f** is generated instead (Scheme 5c). Unexpectedly, when **5g** and *^n^*BuOCS_2_K (*^n^*Bu = *n*-butyl) are used concurrently, sulfation proceeds smoothly to produce a mixture of thioethers **3ag** and **3ah**, indicating that ROCS_2_K is indispensable in the reaction (Scheme 5d).

**Scheme 6:**
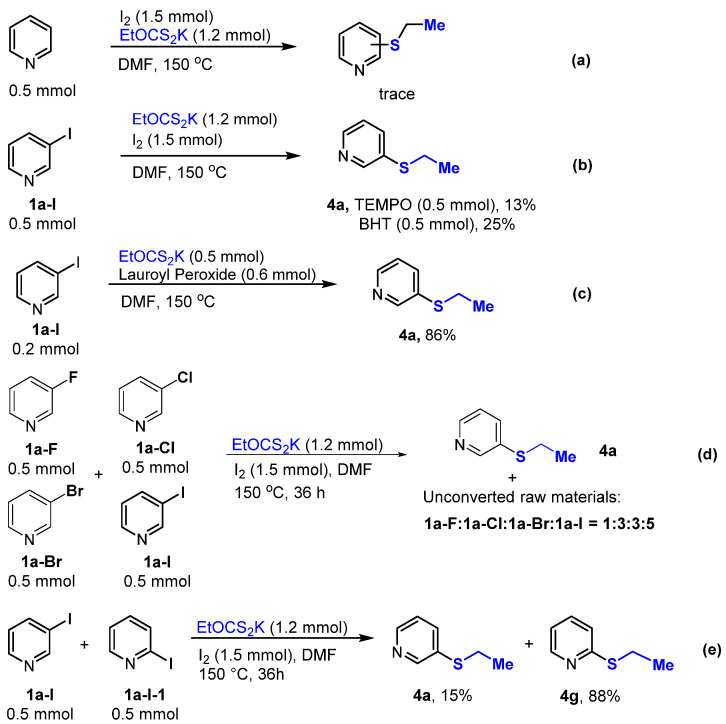
Control experiments for synthesis of alkyl aryl thioethers. (**a**) thioetherification of pyridines; (**b**) Free radical inhibition experiment; (**c**) lauroyl peroxide as radical initiator for thioetherification reaction; (**d**) competitive thioetherification of halogenated pyridines; (**e**) competitive thioetherification of 3-iodopyridines and 2-iodopyridines.

The reaction mechanism of the synthesis of pyridine thioether was then explored. First, pyridine molecules without halogen substituents do not undergo sulfation under the standard reaction conditions, indicating the necessity of halogen substituents or suitable leaving groups (Scheme 6a). When 2,2,6,6-tetramethyl-1-piperidinyloxy (TEMPO) or butylated hydroxytoluene (BHT) is added as a radical scavenger, the sulfuration of 3-iodopyridine is heavily inhibited (Scheme 6b), and thus, the sulfuration reaction may proceed via a radical pathway. When radical initiator lauroyl peroxide was used instead of I_2_, we were pleased to find that the sulfation reaction could still be completed and give **4a** in 86% yields (Scheme 6c). When using 3-fluoropyridine, 3-chloropyridine, 3-bromopyridine, and 3-iodopyridine mixed with **2a** (1.2 mmol), the reactivities of the halogens follow the order F > Cl ≈ Br > I (Scheme 6d) [45]. When 3-iodopyridine and 2-iodopyridine are involved in the reaction, the halogen at the 2-position of pyridine exhibits a higher reactivity (Scheme 6e).

**Scheme 7:**
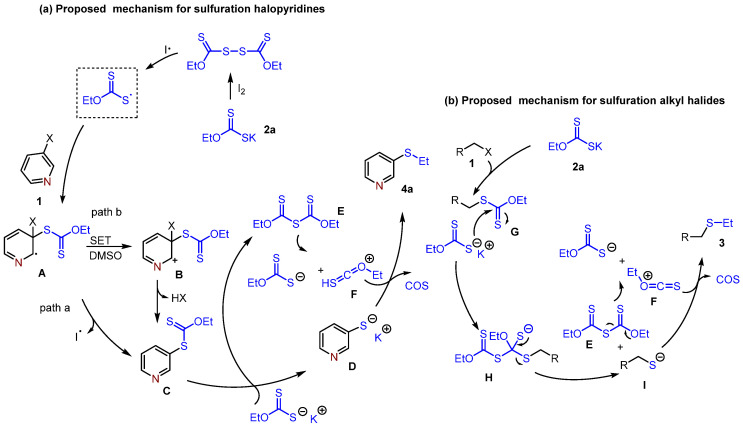
Proposed reaction mechanism.

Based on these results, a plausible mechanism for the sulfuration reaction is proposed, as shown in Scheme 7. Initially, I_2_ liberates an iodine radical (I•), which activates **2a** to afford EtOCSS•, with the concomitant release of electrons [46,47]. The addition of the EtOCSS• radical to 3-iodopyridine then produces radical cation **A** [48,49], which then releases iodine radicals to generate the intermediate xanthate **C**. Subsequently, xanthate **C** undergoes a hydrolysis reaction to produce pyridine-3-thiolate **D**. Moreover, xanthate **C** releases *O*-ethyl ethoxycarbothioylsulfanylmethanethioate **E**, which decomposes to generate ethyl(thioxomethylidene)oxonium **F** and xanthate anions. Finally, the nucleophilic substitution reaction of the pyridine-3-thiolate **D** with oxonium **F** furnishes 3-(ethylthio)pyridine **4a** and releases COS. Alternatively, the intermediate xanthate **C** may be formed through a further single-electron oxidation of intermediate **A** by DMSO or O_2_ to afford the intermediate xanthate carbocation **B** and then releases iodine positive ions [50,51].

As shown in Scheme 7b, the sulfidation reaction of benzyl halides with **2a** proceeds via a similar process. The difference is that benzyl halides react more easily with **2a** via nucleophilic substitution to afford a similar intermediate, i.e., xanthate **G**, without free radical process. The reaction under a nucleophilic attack of EtOCS_2_K on the thiocarbonyl group form xanthate intermediate **G** and subsequently undergoes an intramolecular elimination reaction forming intermediate thiol anion **I** and *O*-ethyl ethoxycarbothioylsulfanylmethanethioate **E**. Finally, the nucleophilic substitution reaction of the thiol anion **I** with oxonium **F** furnishes dialkyl thioether **3** and releases COS.

## 3. Materials and Methods

### 3.1. General Methods (Chemistry)

General methods are described in the Supplementary Materials.

### 3.2. General Procedures for the Preparation of Compounds ***3a**–**3af***

A mixture of 4-(chloromethyl)-1,1′-biphenyl **1a** (101 mg, 0.5 mmol), EtOCS_2_K (160 mg, 1.0 mmol) and DMSO (1 mL) was added successively in a 15 mL Schlenk tube. The Schlenk tube was then immersed in an oil bath at 100 °C in a sealed tube in an air atmosphere stirring for 1 h. After cooling down to room temperature, the solution was filtered through a small amount of silica gel. Then the residue was concentrated in vacuo and the crude was purified by flash chromatography with n-hexane/ethyl acetate (50/1, *v*/*v*).

*([1,1′-biphenyl]-4-ylmethyl)(ethyl)sulfane* (**3a**)

Yellow liquid (106 mg, 93% yield); *R_f_* = 0.6 (Hexane/EtOAc = 50:1); ^1^H NMR (400 MHz, CDCl_3_) δ 7.63–7.55 (m, 4H), 7.49–7.39 (m, 4H), 7.39–7.33 (m, 1H), 3.79 (s, 2H), 2.51 (d, *J* = 7.4 Hz, 2H), 1.29 (t, *J* = 7.4 Hz, 3H); ^13^C NMR (100 MHz, CDCl_3_) δ 140.8, 139.8, 137.7, 129.2 (2C), 128.7 (2C), 127.2, 127.2 (2C), 127.0 (2C), 35.5, 25.2, 14.4; HRMS (ESI-TOF) (*m*/*z*): [M + H]^+^ calcd for C_15_H_17_S^+^, 229.1045; found, 229.1042.

*Ethyl(4-methylbenzyl)sulfane* (**3b**)

Yellow liquid (76 mg, 92% yield); *R_f_* = 0.6 (Hexane/EtOAc = 50:1); ^1^H NMR (400 MHz, CDCl_3_) δ 7.25–7.19 (m, 2H), 7.15–7.10 (m, 2H), 3.87 (s, 2H), 2.48 (q, *J* = 7.3 Hz, 2H), 2.34 (s, 3H), 1.24 (t, *J* = 7.4 Hz, 3H); ^13^C NMR (100 MHz, CDCl_3_) δ 137.1, 134.4, 129.2 (2C), 129.1 (2C), 43.5, 32.4, 21.1, 14.3; HRMS (ESI-TOF) (*m*/*z*): [M + K]^+^ calcd for C_10_H_14_KS^+^, 205.0448; found, 205.0445.

*(4-(tert-butyl)benzyl)(ethyl)sulfane* (**3c**)

Yellow liquid (99 mg, 95% yield); *R_f_* = 0.6 (Hexane/EtOAc = 50:1); ^1^H NMR (400 MHz, CDCl_3_) δ 7.37–7.32 (m, 2H), 7.28–7.23 (m, 2H), 3.71 (s, 2H), 2.47 (q, *J* = 7.4 Hz, 2H), 1.33 (s, 9H), 1.26 (t, *J* = 7.4 Hz, 3H); ^13^C NMR (100 MHz, CDCl_3_) δ 149.7, 135.5, 128.4 (2C), 125.3 (2C), 35.4, 34.4, 31.3, 25.3, 14.3; HRMS (ESI-TOF) (*m*/*z*): [M + K]^+^ calcd for C_13_H_20_KS^+^, 247.0917; found, 247.0914.

*Ethyl(2,4,6-trimethylbenzyl)sulfane* (**3d**)

Yellow liquid (73 mg, 75% yield); *R_f_* = 0.6 (Hexane/EtOAc = 50:1); ^1^H NMR (400 MHz, CDCl_3_) δ 6.85 (s, 2H), 3.78 (s, 2H), 2.61 (q, *J* = 7.4 Hz, 2H), 2.40 (s, 6H), 2.27 (s, 3H), 1.33 (t, *J* = 7.4 Hz, 3H); ^13^C NMR (100 MHz, CDCl_3_) δ 136.8 (2C), 136.3, 131.4, 129.0 (2C), 30.5, 26.8, 20.9, 19.6 (2C), 14.8; HRMS (ESI-TOF) (*m*/*z*): [M + H]^+^ calcd for C_12_H_19_S^+^, 195.1202; found, 195.1207.

*Ethyl(3-methoxybenzyl)sulfane* (**3e**)

Yellow liquid (73 mg, 80% yield); *R_f_* = 0.4 (Hexane/EtOAc = 10:1); ^1^H NMR (400 MHz, CDCl_3_) δ 7.22 (t, *J* = 7.8 Hz, 1H), 6.94–6.85 (m, 2H), 6.82–6.76 (m, 1H), 3.81 (s, 3H), 3.70 (s, 2H), 2.45 (q, *J* = 7.4 Hz, 2H), 1.24 (t, *J* = 7.4 Hz, 3H); ^13^C NMR (100 MHz, CDCl_3_) δ 159.7, 140.2, 129.4, 121.2, 114.3, 112.4, 55.2, 35.9, 25.3, 14.3; HRMS (ESI-TOF) (*m*/*z*): [M + K]^+^ calcd for C_10_H_14_KOS^+^, 221.0397; found, 221.0395.

*(4-(benzyloxy)benzyl)(ethyl)sulfane* (**3f**)

Yellow solid (120 mg, 93% yield), MP: 61–62 °C; *R_f_* = 0.4 (Hexane/EtOAc = 20:1); ^1^H NMR (400 MHz, CDCl_3_) δ 7.46–7.37 (m, 4H), 7.36–7.30 (m, 1H), 7.26–7.21 (m, 2H), 6.95–6.90 (m, 2H), 5.06 (s, 2H), 3.69 (s, 2H), 2.44 (q, *J* = 7.2 Hz, 2H), 1.24 (t, *J* = 7.4 Hz, 3H); ^13^C NMR (100 MHz, CDCl_3_) δ 157.7, 137.0, 130.9, 129.8 (2C), 128.6 (2C), 127.9, 127.4 (2C), 114.8 (2C), 79.7–74.5 (m), 70.0, 35.2, 25.1, 14.4; HRMS (ESI-TOF) (*m*/*z*): [M + H]^+^ calcd for C_16_H_19_OS^+^, 259.1151; found, 259.1149.

*Ethyl(4-(trifluoromethoxy)benzyl)sulfane* (**3g**)

Yellow liquid (111 mg, 94% yield); *R_f_* = 0.5 (Hexane/EtOAc = 50:1); ^1^H NMR (400 MHz, CDCl_3_) δ 7.34 (d, *J* = 8.3 Hz, 2H), 7.15 (d, *J* = 8.2 Hz, 2H), 3.71 (s, 2H), 2.44 (q, *J* = 7.4 Hz, 2H), 1.23 (t, *J* = 7.4 Hz, 3H); ^13^C NMR (100 MHz, CDCl_3_) δ 148.1 (q, *J* = 1.8 Hz), 137.4, 130.1 (2C), 121.0 (2C), 120.5 (q, *J* = 204.2 Hz), 35.1, 25.3, 14.3; HRMS (ESI-TOF) (*m*/*z*): [M + Na]^+^ calcd for C_10_H_11_F_3_NaOS^+^, 259.0375; found, 259.0371.

*Ethyl(4-((trifluoromethyl)thio)benzyl)sulfane* (**3h**)

Yellow liquid (166 mg, 92% yield); *R_f_* = 0.6 (Hexane/EtOAc = 50:1); ^1^H NMR (400 MHz, CDCl_3_) δ 7.63–7.56 (m, 2H), 7.40–7.34 (m, 2H), 3.73 (s, 2H), 2.44 (q, *J* = 7.4 Hz, 2H), 1.23 (t, *J* = 7.4 Hz, 3H); ^13^C NMR (100 MHz, CDCl_3_) δ 142.0, 136.4 (2C), 129.9 (2C), 129.5 (q, *J* = 306.1 Hz), 122.6 (q, *J* = 2.3 Hz), 35.4, 25.4, 14.3; HRMS (ESI-TOF) (*m*/*z*): [M + H]^+^ calcd for C_10_H_12_F_3_S_2_^+^, 253.0327; found, 253.0320.

*Ethyl(4-(phenylthio)benzyl)sulfane* (**3i**)

Yellow liquid (121 mg, 93% yield); *R_f_* = 0.5 (Hexane/EtOAc = 50:1); ^1^H NMR (400 MHz, CDCl_3_) δ 7.37–7.21 (m, 9H), 3.70 (s, 2H), 2.45 (q, *J* = 7.4 Hz, 2H), 1.24 (t, *J* = 7.4 Hz, 3H); ^13^C NMR (100 MHz, CDCl_3_) δ 137.7, 135.8, 134.0, 131.2 (4C), 130.8 (4C), 129.6 (4C), 129.1 (4C), 126.9, 35.4, 25.3, 14.3; HRMS (ESI-TOF) (*m*/*z*): [M + Na]^+^ calcd for C_15_H_16_NaS_2_^+^, 283.0586; found, 283.0593.

*(4-((ethylthio)methyl)phenyl)methanol* (**3j**)

Yellow liquid (82 mg, 90% yield); *R_f_* = 0.5 (Hexane/EtOAc = 2:1); ^1^H NMR (400 MHz, CDCl_3_) δ 7.33–7.27 (m, 4H), 4.65 (s, 2H), 3.71 (s, 2H), 2.42 (q, *J* = 7.3 Hz, 2H), 1.91 (s, 1H), 1.22 (t, *J* = 7.4 Hz, 3H); ^13^C NMR (100 MHz, CDCl_3_) δ 139.5, 138.0, 128.9 (2C), 127.1 (2C), 65.0, 35.5, 25.2, 14.3; HRMS (ESI-TOF) (*m*/*z*): [M + K]^+^ calcd for C_10_H_14_KOS^+^, 221.0397; found, 221.0395.

*2-(4-((ethylthio)methyl)phenyl)-4,4,5,5-tetramethyl-1,3,2-dioxaborolane* (**3k**)

Yellow liquid (127 mg, 91% yield); *R_f_* = 0.5 (Hexane/EtOAc = 20:1); ^1^H NMR (400 MHz, CDCl_3_) δ 7.78–7.73 (m, 2H), 7.36–7.30 (m, 2H), 3.72 (s, 2H), 2.41 (q, *J* = 7.4 Hz, 2H), 1.34 (s, 12H), 1.21 (t, *J* = 7.4 Hz, 3H); ^13^C NMR (100 MHz, CDCl_3_) δ 141.9, 141.8, 134.9 (2C), 128.2 (2C), 83.7 (2C), 35.9, 25.1, 24.8 (4C), 14.3; HRMS (ESI-TOF) (*m*/*z*): [M + H]^+^ calcd for C_15_H_24_BO_2_S^+^, 278.1621; found, 278.1624.

*Ethyl(4-fluorobenzyl)sulfane* (**3l**)

Yellow liquid (81 mg, 95% yield); *R_f_* = 0.6 (Hexane/EtOAc = 50:1); ^1^H NMR (400 MHz, CDCl_3_) δ 7.31–7.25 (m, 2H), 7.03–6.95 (m, 2H), 3.69 (s, 2H), 2.43 (q, *J* = 7.3 Hz, 2H), 1.23 (t, *J* = 7.4 Hz, 3H); ^13^C NMR (100 MHz, CDCl_3_) δ 161.8 (d, *J* = 245.2 Hz), 134.3 (d, *J* = 3.0 Hz), 130.3 (d, *J* = 8.0 Hz, 2C), 115.3 (d, *J* = 21.4 Hz, 2C), 35.1, 25.2, 14.3; HRMS (ESI-TOF) (*m*/*z*): [M + Na]^+^ calcd for C_9_H_11_FNaS^+^, 193.0458; found, 193.0466.

*(4-chlorobenzyl)(ethyl)sulfane* (**3m**)

Yellow liquid (84 mg, 90% yield); *R_f_* = 0.6 (Hexane/EtOAc = 50:1); ^1^H NMR (400 MHz, CDCl_3_) δ 7.30–7.26 (m, 2H), 7.24 (d, *J* = 8.7 Hz, 2H), 3.68 (s, 2H), 2.42 (q, *J* = 7.4 Hz, 2H), 1.22 (t, *J* = 7.4 Hz, 3H); ^13^C NMR (100 MHz, CDCl_3_) δ 137.1, 132.6, 130.1 (2C), 128.6 (2C), 35.2, 25.2, 14.3; HRMS (ESI-TOF) (*m*/*z*): [M + Na]^+^ calcd for C_9_H_11_ClNaS^+^, 209.0162; found, 209.0152.

*(4-bromobenzyl)(ethyl)sulfane* (**3n**)

Yellow liquid (106 mg, 92% yield); *R_f_* = 0.6 (Hexane/EtOAc = 50:1); ^1^H NMR (400 MHz, CDCl_3_) δ 7.47–7.39 (m, 2H), 7.22–7.16 (m, 2H), 3.66 (s, 2H), 2.42 (q, *J* = 7.4 Hz, 2H), 1.22 (t, *J* = 7.4 Hz, 3H); ^13^C NMR (100 MHz, CDCl_3_) δ 137.7, 131.5 (2C), 130.5 (2C), 120.6, 35.2, 25.2, 14.3; HRMS (ESI-TOF) (*m*/*z*): [M + H]^+^ calcd for C_9_H_12_BrS^+^, 230.9838; found, 230.9828.

*Ethyl(4-Iodobenzyl)sulfane* (**3o**)

Yellow liquid (132 mg, 95% yield); *R_f_* = 0.6 (Hexane/EtOAc = 50:1); ^1^H NMR (400 MHz, CDCl_3_) δ 7.67–7.58 (m, 2H), 7.10–7.03 (m, 2H), 3.65 (s, 2H), 2.41 (q, *J* = 7.4 Hz, 2H), 1.22 (t, *J* = 7.4 Hz, 3H); ^13^C NMR (100 MHz, CDCl_3_) δ 138.3, 137.5 (2C), 130.8 (2C), 92.1, 35.3, 25.2, 14.3; HRMS (ESI-TOF) (*m*/*z*): [M + H]^+^ calcd for C_9_H_12_IS^+^, 278.9699; found, 278.9693.

*Ethyl(4-(trifluoromethyl)benzyl)sulfane* (**3p**)

Yellow liquid (103 mg, 94% yield); *R_f_* = 0.6 (Hexane/EtOAc = 50:1); ^1^H NMR (400 MHz, CDCl_3_) δ 7.57 (d, *J* = 8.0 Hz, 2H), 7.43 (d, *J* = 8.0 Hz, 2H), 3.75 (s, 2H), 2.43 (q, *J* = 7.4 Hz, 2H), 1.24 (t, *J* = 7.4 Hz, 3H); ^13^C NMR (100 MHz, CDCl_3_) δ 142.9, 129.1 (2C), 129.1 (q, *J* = 31.7 Hz), 125.4 (q, *J* = 3.8 Hz, 2C), 124.2 (q, *J* = 269.9 Hz), 35.4, 25.3, 14.2; HRMS (ESI-TOF) (*m*/*z*): [M + Na]^+^ calcd for C_10_H_11_F_3_NaOS^+^, 259.0375; found, 259.0371.

*Ethyl(4-(methylsulfonyl)benzyl)sulfane* (**3q**)

Yellow liquid (105 mg, 91% yield); *R_f_* = 0.4 (Hexane/EtOAc = 1:1); ^1^H NMR (400 MHz, CDCl_3_) δ 7.91–7.82 (m, 2H), 7.54–7.46 (m, 2H), 3.75 (s, 2H), 3.03 (s, 3H), 2.42 (q, *J* = 7.4 Hz, 2H), 1.21 (t, *J* = 7.4 Hz, 3H); ^13^C NMR (100 MHz, CDCl_3_) δ 145.3, 138.9, 129.6 (2C), 127.5 (2C), 44.4, 35.4, 25.4, 14.2; HRMS (ESI-TOF) (*m*/*z*): [M + H]^+^ calcd for C_10_H_15_O_2_S_2_^+^, 231.0508; found, 231.0503.

*4-((ethylthio)methyl)-N-phenylbenzamide* (**3r**)

White solid (126 mg, 93% yield), MP: 118–120 °C; *R_f_* = 0.4 (Hexane/EtOAc = 3:1); ^1^H NMR (400 MHz, CDCl_3_) δ 8.08 (s, 1H), 7.83–7.76 (m, 2H), 7.66–7.61 (m, 2H), 7.40–7.30 (m, 4H), 7.16–7.10 (m, 1H), 3.74 (s, 2H), 2.50–2.36 (m, 2H), 1.23 (t, *J* = 7.4 Hz, 3H); ^13^C NMR (100 MHz, CDCl_3_) δ 165.6, 142.8, 137.9, 133.5, 129.1 (2C), 129.0 (2C), 127.3 (2C), 124.5, 120.3 (2C), 35.5, 25.3, 14.3; HRMS (ESI-TOF) (*m*/*z*): [M + H]^+^ calcd for C_16_H_18_NOS^+^, 272.1104; found, 272.1098.

*Ethyl(naphthalen-1-ylmethyl)sulfane* (**3s**)

Yellow liquid (94 mg, 93% yield); *R_f_* = 0.6 (Hexane/EtOAc = 50:1); ^1^H NMR (400 MHz, CDCl_3_) δ 8.16 (d, *J* = 8.3 Hz, 1H), 7.89–7.84 (m, 1H), 7.81–7.73 (m, 1H), 7.59–7.53 (m, 1H), 7.52–7.47 (m, 1H), 7.44–7.35 (m, 2H), 4.19 (s, 2H), 2.51 (q, *J* = 7.3 Hz, 2H), 1.28 (t, *J* = 7.4 Hz, 3H); ^13^C NMR (100 MHz, CDCl_3_) δ 134.1, 133.9, 131.4, 128.8, 128.0, 126.9, 126.1, 125.8, 125.1, 124.1, 33.7, 26.0, 14.4; HRMS (ESI-TOF) (*m*/*z*): [M]^+^ calcd for C_13_H_14_S^+^, 202.0811; found, 202.0816.

*Ethyl(naphthalen-2-ylmethyl)sulfane* (**3t**)

Yellow liquid (96 mg, 95% yield); *R_f_* = 0.6 (Hexane/EtOAc = 50:1); ^1^H NMR (400 MHz, CDCl_3_) δ 7.87–7.79 (m, 3H), 7.72 (s, 1H), 7.55–7.44 (m, 3H), 3.90 (s, 2H), 2.46 (q, *J* = 7.4 Hz, 2H), 1.26 (t, *J* = 7.4 Hz, 3H); ^13^C NMR (100 MHz, CDCl_3_) δ 135.9, 133.2, 132.5, 128.3, 127.6, 127.6, 127.1, 126.1, 125.6, 36.1, 25.1, 14.3; HRMS (ESI-TOF) (*m*/*z*): [M]^+^ calcd for C_13_H_14_S^+^, 202.0811; found, 202.0818.

*(Anthracen-9-ylmethyl)(ethyl)sulfane* (**3u**)

Yellow solid (53 mg, 42% yield), MP: 68–70 °C; *R_f_* = 0.6 (Hexane/EtOAc = 50:1); ^1^H NMR (400 MHz, CDCl_3_) δ 8.39 (s, 1H), 8.35 (d, *J* = 8.9 Hz, 2H), 8.01 (d, *J* = 8.4 Hz, 2H), 7.61–7.53 (m, 2H), 7.52–7.44 (m, 2H), 4.75 (s, 2H), 2.70 (q, *J* = 7.4 Hz, 2H), 1.37 (t, *J* = 7.4 Hz, 3H); ^13^C NMR (100 MHz, CDCl_3_) δ 131.5 (2C), 129.9 (2C), 129.5, 129.2 (2C), 127.2, 126.0 (2C), 125.0 (2C), 124.2 (2C), 28.7, 27.1, 14.8; HRMS (ESI-TOF) (*m*/*z*): [M + H]^+^ calcd for C_17_H_17_S^+^, 253.1045; found, 253.1045.

*2-chloro-5-((ethylthio)methyl)thiophene* (**3v**)

Yellow liquid (89 mg, 93% yield); *R_f_* = 0.6 (Hexane/EtOAc = 50:1); ^1^H NMR (400 MHz, CDCl_3_) δ 6.73–6.66 (m, 2H), 3.82 (s, 2H), 2.51 (q, *J* = 7.4 Hz, 2H), 1.24 (t, *J* = 7.4 Hz, 3H); ^13^C NMR (100 MHz, CDCl_3_) δ 141.4, 128.8, 125.5, 125.0, 30.4, 25.4, 14.2; HRMS (ESI-TOF) (*m*/*z*): [M + H]^+^ calcd for C_7_H_10_ClS_2_^+^, 192.9907; found, 192.9902.

*5-chloro-3-((ethylthio)methyl)benzo[b]thiophene* (**3w**)

Yellow liquid (111 mg, 92% yield); *R_f_* = 0.6 (Hexane/EtOAc = 50:1); ^1^H NMR (400 MHz, CDCl_3_) δ 7.88–7.84 (m, 1H), 7.74 (d, *J* = 8.6 Hz, 1H), 7.35–7.29 (m, 2H), 3.92 (s, 3H), 2.48 (q, *J* = 7.4 Hz, 2H), 1.26 (t, *J* = 7.4 Hz, 3H); ^13^C NMR (100 MHz, CDCl_3_) δ 139.1, 138.7, 131.9, 130.4, 125.4, 124.9, 123.8, 121.9, 28.9, 25.7, 14.2; HRMS (ESI-TOF) (*m*/*z*): [M + Na]^+^ calcd for C_11_H_11_ClNaS_2_^+^, 264.9883; found, 264.9884.

*8-((ethylthio)methyl)quinoline* (**3x**)

Yellow liquid (92 mg, 91% yield); *R_f_* = 0.4 (Hexane/EtOAc = 10:1); ^1^H NMR (400 MHz, CDCl_3_) δ 8.97–8.93 (m, 1H), 8.16–8.09 (m, 1H), 7.71 (d, *J* = 7.7 Hz, 2H), 7.52–7.45 (m, 1H), 7.42–7.37 (m, 1H), 4.44 (s, 2H), 2.56 (q, *J* = 7.4 Hz, 2H), 1.28 (t, *J* = 7.4 Hz, 3H); ^13^C NMR (100 MHz, CDCl_3_) δ 149.6, 146.3, 137.3, 136.3, 129.3, 128.5, 127.0, 126.1, 121.1, 31.1, 26.1, 14.5; HRMS (ESI-TOF) (*m*/*z*): [M + H]^+^ calcd for C_12_H_14_NS^+^, 204.0842; found, 204.0843.

*2-((ethylthio)methyl)-4-methylquinazoline* (**3y**)

Yellow solid (95 mg, 87% yield), MP: 52–54 °C; *R_f_* = 0.6 (Hexane/EtOAc = 50:1); ^1^H NMR (400 MHz, CDCl_3_) δ 8.03–7.98 (m, 1H), 7.95–7.89 (m, 1H), 7.82–7.76 (m, 1H), 7.56–7.50 (m, 1H), 4.01 (s, 2H), 2.89 (s, 3H), 2.62 (q, *J* = 7.4 Hz, 2H), 1.24 (t, *J* = 7.4 Hz, 3H); ^13^C NMR (100 MHz, CDCl_3_) δ 168.8, 163.6, 149.6, 133.5, 128.5, 126.9, 124.8, 122.5, 39.1, 25.9, 21.7, 14.4; HRMS (ESI-TOF) (*m*/*z*): [M + H]^+^ calcd for C_12_H_15_N_2_S^+^, 219.0950; found, 219.0942.

*1-(4-((ethylthio)methyl)phenyl)-1H-pyrazole* (**3z**)

Yellow liquid (93 mg, 85% yield); *R_f_* = 0.5 (Hexane/EtOAc = 10:1); ^1^H NMR (400 MHz, CDCl_3_) δ 7.95–7.88 (m, 1H), 7.74–7.70 (m, 1H), 7.64 (d, *J* = 8.3 Hz, 2H), 7.45–7.37 (m, 2H), 3.75 (s, 2H), 2.45 (q, *J* = 7.3, 6.8 Hz, 2H), 1.24 (t, *J* = 7.3 Hz, 3H); ^13^C NMR (100 MHz, CDCl_3_) δ 141.0, 139.0, 136.9, 129.8 (2C), 126.7, 119.3 (2C), 107.6, 35.3, 25.2, 14.4; HRMS (ESI-TOF) (*m*/*z*): [M + H]^+^ calcd for C_12_H_15_N_2_S^+^, 219.0950; found, 219.0946.

*1-cyclohexyl-5-(4-(ethylthio)butyl)-1H-tetrazole* (**3aa**)

Yellow liquid (123 mg, 92% yield); *R_f_* = 0.4 (Hexane/EtOAc = 2:1); ^1^H NMR (400 MHz, CDCl_3_) δ 4.21–4.04 (m, 1H), 2.85 (t, 2H), 2.61–2.48 (m, 3H), 2.09–1.87 (m, 8H), 1.82–1.66 (m, 4H), 1.48–1.30 (m, 3H), 1.24 (t, *J* = 7.4 Hz, 3H); ^13^C NMR (100 MHz, CDCl_3_) δ 153.5, 57.6, 32.9 (2C), 30.9, 28.6, 26.2, 25.9, 25.3 (2C), 24.8, 22.9, 14.8; HRMS (ESI-TOF) (*m*/*z*): [M + H]^+^ calcd for C_13_H_25_N_4_S^+^, 269.1794; found, 269.1790.

*1,4-bis((ethylthio)methyl)benzene* (**3ab**)

Yellow liquid (99 mg, 88% yield); *R_f_* = 0.5 (Hexane/EtOAc = 50:1); ^1^H NMR (400 MHz, CDCl_3_) δ 7.25 (s, 4H), 3.70 (s, 4H), 2.43 (q, *J* = 7.4 Hz, 4H), 1.23 (t, *J* = 7.4 Hz, 6H); ^13^C NMR (100 MHz, CDCl_3_) δ 137.2 (2C), 128.9 (4C), 35.5, 25.2, 14.4; HRMS (ESI-TOF) (*m*/*z*): [M]^+^ calcd for C_12_H_18_S_2_^+^, 226.0845; found, 226.0836.

*(((1s,3s)-adamantan-1-yl)methyl)(ethyl)sulfane* (**3ac**)

Yellow liquid (42 mg, 40% yield); *R_f_* = 0.6 (Hexane/EtOAc = 50:1); ^1^H NMR (400 MHz, CDCl_3_) δ 2.51 (q, *J* = 7.4 Hz, 2H), 2.32 (s, 2H), 1.97 (s, 3H), 1.73–1.66 (m, 3H), 1.65–1.59 (m, 3H), 1.58–1.54 (m, 6H), 1.24 (t, *J* = 7.4 Hz, 3H); ^13^C NMR (100 MHz, CDCl_3_) δ 47.3, 41.9 (3C), 36.9 (3C), 33.9, 28.6 (3C), 28.2, 15.0; HRMS (ESI-TOF) (*m*/*z*): [M + K]^+^ calcd for C_13_H_22_KS^+^, 249.1074; found, 249.1075.

*Benzhydryl(ethyl)sulfane* (**3ad**)

Yellow liquid (80 mg, 70% yield); *R_f_* = 0.6 (Hexane/EtOAc = 50:1); ^1^H NMR (400 MHz, CDCl_3_) δ 7.50–7.44 (m, 4H), 7.38–7.31 (m, 4H), 7.30–7.21 (m, 2H), 5.22 (s, 1H), 2.44 (q, *J* = 7.4 Hz, 2H), 1.25 (t, *J* = 7.4 Hz, 3H); ^13^C NMR (100 MHz, CDCl_3_) δ 141.5 (2C), 128.5 (4C), 128.2 (4C), 127.0 (2C), 53.7, 26.2, 14.2; HRMS (ESI-TOF) (*m*/*z*): [M + H]^+^ calcd for C_15_H_17_S^+^, 229.1045; found, 229.1046.

*Dibenzylsulfane* (**3ae**) [52]

White solid (100 mg, 93% yield), MP: 61–62 °C; *R_f_* = 0.6 (Hexane/EtOAc = 50:1); ^1^H NMR (400 MHz, CDCl_3_) δ 7.36–7.22 (m, 10H), 3.61 (s, 4H); ^13^C NMR (100 MHz, CDCl_3_) δ 138.1 (2C), 129.0 (4C), 128.5 (4C), 127.0 (2C), 35.6 (2C).

*benzyl(phenyl)sulfane* (**3af**) [53]

White solid (90 mg, 90% yield), MP: 39–40 °C; *Rf* = 0.6 (Hexane/EtOAc = 50:1); 1H NMR (400 MHz, CDCl_3_) δ 7.34–7.22 (m, 9H), 7.21–7.15 (m, 1H), 4.13 (s, 2H); ^13^C NMR (100 MHz, CDCl_3_) δ 137.4, 136.3, 129.8 (2C), 128.8 (2C), 128.8 (2C), 128.5 (2C), 127.2, 126.3, 39.0.

*Benzyl(ethyl)sulfane* (**3ag**)

Yellow liquid (27 mg, 36% yield); *Rf* = 0.6 (Hexane/EtOAc = 50:1); ^1^H NMR (500 MHz, CDCl_3_) δ 7.35–7.28 (m, 4H), 7.26–7.21 (m, 1H), 3.73 (s, 2H), 2.44 (q, *J* = 7.4 Hz, 2H), 1.23 (t, *J* = 7.4 Hz, 3H); ^13^C NMR (125 MHz, CDCl3) δ 138.6, 128.8 (2C), 128.4 (2C), 126.8, 35.8, 25.2, 14.3; HRMS (ESI-TOF) (*m*/*z*): [M + H]+ calcd for C_9_H_13_S^+^, 153.0732; found, 153.0735.

*Benzyl(butyl)sulfane* (**3ah**)

Yellow liquid (33 mg, 37% yield); *Rf* = 0.6 (Hexane/EtOAc = 50:1); ^1^H NMR (400 MHz, CDCl_3_) δ 7.35–7.25 (m, 4H), 7.28–7.19 (m, 1H), 3.71 (s, 2H), 2.49–2.35 (m, 2H), 1.60–1.49 (m, 2H), 1.38 (dt, *J* = 8.1, 7.0 Hz, 2H), 0.89 (t, *J* = 7.3 Hz, 3H); ^13^C NMR (100 MHz, CDCl3) 138.7, 128.8 (2C), 128.4 (2C), 126.8, 36.2, 31.3, 31.0, 22.0, 13.7; HRMS (ESI-TOF) (*m*/*z*): [M + H]+ calcd for C_11_H_17_S^+^, 181.1045; found, 181.1050.

### 3.3. General Procedures for the Preparation of Compounds ***4a–4s***

A mixture of 3-Iodine pyridine (103 mg, 0.5 mmol), EtOCS_2_K (192 mg, 1.2 mmol), I_2_ (381 mg, 1.5 mmol), and DMF (3 mL) was added successively in a 15 mL Schlenk tube. The Schlenk tube was then immersed in an oil bath at 150 °C in a sealed tube in an air atmosphere stirring for 36 h. After cooling down to room temperature, the solution was filtered through a small amount of silica gel. Then the residue was concentrated in vacuo and the crude was purified by flash chromatography with n-hexane/ethyl acetate (3/1, *v*/*v*).

*3-(Ethylthio)pyridine* (**4a**) [54]

Yellow liquid (64 mg, 92% yield); *R_f_* = 0.5 (Hexane/EtOAc = 3:1); ^1^H NMR (500 MHz, CDCl_3_) δ 8.56 (s, 1H), 8.41 (d, *J* = 4.8 Hz, 1H), 7.66 (ddd, *J* = 8.0, 2.4, 1.5 Hz, 1H), 7.23 (dd, *J* = 8.0, 4.8 Hz, 1H), 2.96 (q, *J* = 7.4 Hz, 2H), 1.32 (t, *J* = 7.4 Hz, 3H); ^13^C NMR (125 MHz, CDCl_3_) δ 149.6, 146.5, 137.1, 134.0, 123.7, 27.7, 14.3.

*3-(Propylthio)pyridine* (**4b**) [55]

Yellow liquid (58 mg, 75%); *R_f_* = 0.5 (Hexane/EtOAc = 3:1); ^1^H NMR (500 MHz, CDCl_3_) δ 8.56 (d, *J* = 1.8 Hz, 1H), 8.48–8.35 (m, 1H), 7.69–7.60 (m, 1H), 7.21 (dd, *J* = 7.9, 4.8 Hz, 1H), 2.96–2.74 (m, 2H), 1.67 (h, *J* = 7.3 Hz, 2H), 1.03 (t, *J* = 7.4 Hz, 3H); ^13^C NMR (125 MHz, CDCl_3_) δ 149.9, 146.7, 136.8, 134.1, 123.6, 35.7, 22.4, 13.3.

*3-(Butylthio)pyridine* (**4c**) [56]

Yellow liquid (62 mg, 74%); *R_f_* = 0.5 (Hexane/EtOAc = 3:1); ^1^H NMR (500 MHz, CDCl_3_) δ 8.55 (s, 1H), 8.41 (d, *J* = 4.2 Hz, 1H), 7.66 (dt, *J* = 8.0, 1.8 Hz, 1H), 7.24 (dd, *J* = 7.9, 4.8 Hz, 1H), 2.97–2.90 (m, 2H), 1.63 (p, *J* = 7.4 Hz, 2H), 1.45 (dq, *J* = 14.6, 7.3 Hz, 2H), 0.92 (t, *J* = 7.3 Hz, 3H); ^13^C NMR (125 MHz, CDCl_3_) δ 149.2, 146.2, 137.0, 134.6, 123.7, 33.3, 31.1, 21.8, 13.6.

*3-(Pentylthio)pyridine* (**4d**)

Yellow liquid (67 mg, 74%); *R_f_* = 0.5 (Hexane/EtOAc = 3:1); ^1^H NMR (500 MHz, CDCl_3_) δ 8.55 (s, 1H), 8.41 (s, 1H), 7.66 (d, *J* = 7.9 Hz, 1H), 7.24–7.21 (m, 1H), 2.92 (t, *J* = 7.4 Hz, 2H), 1.64 (p, *J* = 7.4 Hz, 2H), 1.40 (dt, *J* = 14.3, 6.9 Hz, 2H), 1.32 (dq, *J* = 14.3, 6.9 Hz, 2H), 0.89 (t, *J* = 7.2 Hz, 3H); ^13^C NMR (125 MHz, CDCl_3_) δ 149.4, 146.4, 136.9, 134.4, 123.6, 33.6, 30.8, 28.7, 22.2, 13.9. HRMS (ESI-TOF) (*m*/*z*): [M + H]^+^ calcd for C_10_H_16_NS^+^, 182.0998; found, 182.0995.

*3-(Ethylthio)quinoline* (**4e**) [57]

Yellow liquid (89 mg, 94% yield); *R_f_* = 0.4 (Hexane/EtOAc = 5:1); ^1^H NMR (500 MHz, CDCl_3_) δ 8.84 (d, *J* = 2.3 Hz, 1H), 8.10 (d, *J* = 8.4 Hz, 1H), 8.07 (d, *J* = 2.3 Hz, 1H), 7.75 (dd, *J* = 8.2, 1.4 Hz, 1H), 7.68 (ddd, *J* = 8.4, 6.9, 1.4 Hz, 1H), 7.56 (ddd, *J* = 8.2, 6.9, 1.2 Hz, 1H), 3.06 (q, *J* = 7.4 Hz, 2H), 1.37 (t, *J* = 7.4 Hz, 3H); ^13^C NMR (125 MHz, CDCl_3_) δ 151.6, 146.2, 134.8, 130.6, 129.3, 129.0, 128.2, 127.2, 126.9, 27.9, 14.3.

*4-(Ethylthio)isoquinoline* (**4f**) [57]

Yellow liquid (80 mg, 85% yield); *R_f_* = 0.4 (Hexane/EtOAc = 5:1); ^1^H NMR (500 MHz, CDCl_3_) δ 9.12 (s, 1H), 8.55 (s, 1H), 8.31 (d, *J* = 8.4 Hz, 1H), 7.98 (d, *J* = 8.1 Hz, 1H), 7.77 (ddd, *J* = 8.3, 6.8, 1.3 Hz, 1H), 7.64 (ddd, *J* = 8.1, 6.8, 1.1 Hz, 1H), 3.01 (q, *J* = 7.3 Hz, 2H), 1.32 (t, *J* = 7.4 Hz, 3H); ^13^C NMR (125 MHz, CDCl_3_) δ 151.0, 143.7, 135.9, 130.9, 128.7, 128.4, 128.2, 127.67, 124.1, 28.3, 14.5.

*2-(Ethylthio)pyridine* (**4g**) [58]

Yellow liquid (63 mg, 90% yield); *R_f_* = 0.4 (Hexane/EtOAc = 5:1); ^1^H NMR (500 MHz, CDCl_3_) δ 8.41 (ddd, *J* = 5.0, 1.9, 1.0 Hz, 1H), 7.50–7.41 (m, 1H), 7.15 (dt, *J* = 8.1, 1.1 Hz, 1H), 6.95 (ddd, *J* = 7.3, 4.9, 1.1 Hz, 1H), 3.16 (q, *J* = 7.4 Hz, 2H), 1.36 (t, *J* = 7.4 Hz, 3H); ^13^C NMR (125 MHz, CDCl_3_) δ 159.3, 149.3, 135.8, 122.1, 119.2, 24.4, 14.5.

*2-(Ethylthio)-3-methylpyridine* (**4h**)

Yellow liquid (71 mg, 93% yield); *R_f_* = 0.4 (Hexane/EtOAc = 5:1); ^1^H NMR (500 MHz, CDCl_3_) δ 8.29 (dd, *J* = 4.9, 1.7 Hz, 1H), 7.30 (ddd, *J* = 7.4, 1.8, 0.9 Hz, 1H), 6.90 (dd, *J* = 7.4, 4.9 Hz, 1H), 3.22 (q, *J* = 7.4 Hz, 2H), 2.24 (s, 3H), 1.38 (t, *J* = 7.4 Hz, 3H); ^13^C NMR (125 MHz, CDCl_3_) δ 158.2, 146.4, 136.2, 130.9, 118.8, 24.0, 18.6, 14.6. HRMS (ESI-TOF) (*m*/*z*): [M + H]^+^ calcd for C_8_H_12_NS^+^, 154.0685; found, 154.0685.

*4-2-(Ethylthio)-4-methylpyridine* (**4i**)

Yellow liquid (69 mg, 90% yield); *R_f_* = 0.4 (Hexane/EtOAc = 5:1); ^1^H NMR (500 MHz, CDCl_3_) δ 8.28 (d, *J* = 5.1 Hz, 1H), 6.99 (s, 1H), 6.79 (dd, *J* = 5.2, 1.5 Hz, 1H), 3.15 (q, *J* = 7.4 Hz, 2H), 2.26 (s, 3H), 1.36 (t, *J* = 7.4 Hz, 3H); ^13^C NMR (125 MHz, CDCl_3_) δ 158.9, 148.9, 147.2, 122.7, 120.7, 24.4, 20.8, 14.6. HRMS (ESI-TOF) (*m*/*z*): [M + H]^+^ calcd for C_8_H_12_NS^+^, 154.0685; found, 154.0685.

*6-(Ethylthio)pyridin-3-amine* (**4j**) [59]

Brown liquid (72 mg, 94% yield); *R_f_* = 0.4 (Hexane/EtOAc = 3:1); ^1^H NMR (500 MHz, CDCl_3_) δ 8.02 (d, *J* = 2.9 Hz, 1H), 7.04 (dd, *J* = 8.4, 0.7 Hz, 1H), 6.91 (dd, *J* = 8.4, 2.9 Hz, 1H), 3.51 (s, 2H), 3.07 (q, *J* = 7.3 Hz, 2H), 1.31 (t, *J* = 7.4 Hz, 3H); ^13^C NMR (125 MHz, CDCl_3_) δ 146.5, 139.9, 136.9, 123.9, 123.4, 25.9, 14.7.

*2-(Ethylthio)pyridin-3-amine* (**4k**) [60]

Brown liquid (67 mg, 87% yield); *R_f_* = 0.4 (Hexane/EtOAc = 3:1); ^1^H NMR (500 MHz, CDCl_3_) δ 7.97 (dd, *J* = 4.2, 2.0 Hz, 1H), 6.93–6.83 (m, 2H), 3.23 (q, *J* = 7.4 Hz, 2H), 1.35 (t, *J* = 7.4 Hz, 3H); ^13^C NMR (125 MHz, CDCl_3_) δ 143.0, 140.9, 139.4, 120.6, 120.4, 25.2, 14.9.

*6-(Ethylthio)pyridin-3-ol* (**4l**)

Pale-yellow solid (73 mg, 94% yield); *R_f_* = 0.4 (Hexane/EtOAc = 5:1); ^1^H NMR (500 MHz, CDCl_3_) δ 8.12 (dd, *J* = 2.8, 0.8 Hz, 1H), 7.22 (dd, *J* = 8.6, 2.8 Hz, 1H), 7.18 (dd, *J* = 8.7, 0.7 Hz, 1H), 3.02 (q, *J* = 7.3 Hz, 2H), 1.29 (t, *J* = 7.3 Hz, 3H); ^13^C NMR (125 MHz, CDCl_3_) δ 152.0, 148.1, 137.0, 125.8, 125.1, 26.9, 14.5. HRMS (ESI-TOF) (*m*/*z*): [M + H]^+^ calcd for C_7_H_10_NOS^+^, 156.0478; found, 156.0476.

*6-(Ethylthio)-4-methylpyridin-3-amine* (**4m**)

Red liquid (80 mg, 95% yield); *R_f_* = 0.5 (Hexane/EtOAc = 2:1); ^1^H NMR (500 MHz, CDCl_3_) δ 7.93 (s, 1H), 6.95 (t, *J* = 0.7 Hz, 1H), 3.07 (q, *J* = 7.4 Hz, 2H), 2.13 (d, *J* = 0.8 Hz, 3H), 1.31 (t, *J* = 7.3 Hz, 3H); ^13^C NMR (125 MHz, CDCl_3_) δ 146.7, 138.8, 136.5, 132.2, 124.9, 25.8, 16.8, 14.8. HRMS (ESI-TOF) (*m*/*z*): [M + H]^+^ calcd for C_8_H_13_N_2_S^+^, 169.0794; found, 169.0792.

*2-(Ethylthio)-3-phenylpyridine* (**4n**)

Yellow liquid (91 mg, 85% yield); *R_f_* = 0.5 (Hexane/EtOAc = 10:1); ^1^H NMR (500 MHz, CDCl_3_) δ 8.46 (dd, *J* = 4.9, 1.7 Hz, 1H), 7.50–7.42 (m, 5H), 7.41 (dd, *J* = 7.4, 1.8 Hz, 1H), 7.06 (dd, *J* = 7.5, 4.9 Hz, 1H), 3.19 (q, *J* = 7.3 Hz, 2H), 1.35 (t, *J* = 7.4 Hz, 3H); ^13^C NMR (125 MHz, CDCl_3_) δ 157.5, 147.9, 138.2, 136.3, 136.0, 129.1, 128.3, 128.0, 118.8, 24.6, 14.3. HRMS (ESI-TOF) (*m*/*z*): [M + H]^+^ calcd for C_13_H_14_NS^+^, 216.0841; found, 216.0839.

*6-(Ethylthio)-N,N-dimethylpicolinamide* (**4o**)

Yellow liquid (92 mg, 88% yield); *R_f_* = 0.4 (Hexane/EtOAc = 2:1); ^1^H NMR (500 MHz, CDCl_3_) δ 7.54 (t, *J* = 7.8 Hz, 1H), 7.29 (d, *J* = 7.5 Hz, 1H), 7.16 (d, *J* = 8.1 Hz, 1H), 3.15 (q, *J* = 7.4 Hz, 2H), 3.10 (d, *J* = 17.2 Hz, 6H), 1.34 (t, *J* = 7.4 Hz, 3H); ^13^C NMR (125 MHz, CDCl_3_) δ 168.5, 158.1, 154.0, 136.6, 122.7, 119.1, 39.0, 35.8, 24.2, 14.6. HRMS (ESI-TOF) (*m*/*z*): [M + Na]^+^ calcd for C_10_H_14_N_2_NaOS^+^, 233.0719; found, 233.0715.

*2-(Ethylthio)-3-iodopyridine* (**4p**)

Brown liquid (119 mg, 90% yield); *R_f_* = 0.4 (Hexane/EtOAc = 5:1); ^1^H NMR (500 MHz, CDCl_3_) δ 8.39 (dd, *J* = 4.7, 1.6 Hz, 1H), 7.90 (dd, *J* = 7.7, 1.6 Hz, 1H), 6.70 (dd, *J* = 7.7, 4.7 Hz, 1H), 3.13 (q, *J* = 7.4 Hz, 2H), 1.37 (t, *J* = 7.4 Hz, 3H); ^13^C NMR (125 MHz, CDCl_3_) δ 161.8, 148.1, 145.7, 119.9, 93.7, 26.9, 14.0. HRMS (ESI-TOF) (*m*/*z*): [M + H]^+^ calcd for C_7_H_9_INS^+^, 265.9500; found, 265.9504.

*6-(Ethylthio)picolinonitrile* (**4q**)

Brown solid (71 mg, 87% yield); MP: 50–52 °C, *R_f_* = 0.4 (Hexane/EtOAc = 3:1); ^1^H NMR (500 MHz, CDCl_3_) δ 8.64 (d, *J* = 2.5 Hz), 7.63 (dd, *J* = 8.4, 2.2 Hz), 7.21 (dd, *J* = 8.4, 0.9 Hz), 3.19 (q, *J* = 7.4 Hz, 2H), 1.37 (t, *J* = 7.4 Hz, 3H); ^13^C NMR (125 MHz, CDCl_3_) δ 165.6, 152.1, 137.6, 121.7, 117.1, 104.3, 24.5, 14.2. HRMS (ESI-TOF) (*m*/*z*): [M + H]^+^ calcd for C_8_H_9_N_2_S^+^, 165.0481; found, 165.0481.

*3,5-Bis(ethylthio)pyridine* (**4r**)

Brown liquid (40 mg, 40% yield); *R_f_* = 0.4 (Hexane/EtOAc = 5:1); ^1^H NMR (500 MHz, CDCl_3_) δ 8.32 (s, 2H), 7.55 (s, 1H), 2.95 (q, *J* = 7.4 Hz, 4H), 1.31 (t, *J* = 7.4 Hz, 6H); ^13^C NMR (125 MHz, CDCl_3_) δ 146.5 (2C), 136.6 (2C), 134.0, 27.6 (2C), 14.2 (2C). HRMS (ESI-TOF) (*m*/*z*): [M + H]^+^ calcd for C_9_H_14_NS_2_^+^, 200.0562; found, 200.0559.

*2-(Ethylthio)-5-iodopyrimidine* (**4s**)

Brown solid (122 mg, 92% yield); MP: 64–65 °C, *R_f_* = 0.5 (Hexane/EtOAc = 3:1); ^1^H NMR (500 MHz, CDCl_3_) δ 8.64 (s, 2H), 3.09 (q, *J* = 7.4 Hz, 2H), 1.36 (t, *J* = 7.4 Hz, 3H); ^13^C NMR (125 MHz, CDCl_3_) δ 171.1, 162.2 (2C), 86.2, 25.4, 14.2. HRMS (ESI-TOF) (*m*/*z*): [M + H]^+^ calcd for C_6_H_8_IN_2_S^+^, 266.9447; found, 266.9450.

*4-(Ethylsulfonyl)aniline* (**5a**)

Yellow liquid (138 mg, 93% yield); *R_f_* = 0.5 (Hexane/EtOAc = 3:1); ^1^H NMR (500 MHz, CDCl_3_) δ 8.58 (dd, *J* = 4.6, 1.5 Hz, 1H), 8.40 (dd, *J* = 8.0, 1.5 Hz, 1H), 7.20 (dd, *J* = 8.0, 4.5 Hz, 1H), 3.67 (q, *J* = 7.4 Hz, 2H), 1.46 (t, *J* = 7.4 Hz, 3H); ^13^C NMR (125 MHz, CDCl_3_) δ 157.3, 150.9, 147.2, 127.3, 86.1, 46.0, 7.2. HRMS (ESI-TOF) (*m*/*z*): [M]^+^ calcd for C_7_H_8_INO_2_S^+^, 296.9553; found, 296.9551.

*2-((1-Chloroethyl)thio)-3-iodopyridine* (**5b**)

Yellow liquid (136 mg, 91% yield); *R_f_* = 0.5 (Hexane/EtOAc = 5:1); ^1^H NMR (500 MHz, CDCl_3_) δ 8.50 (dt, *J* = 4.7, 1.2 Hz, 1H), 7.98 (dt, *J* = 7.7, 1.2 Hz, 1H), 6.82 (ddd, *J* = 7.8, 4.7, 0.8 Hz, 1H), 6.19 (q, *J* = 6.9 Hz, 1H), 2.01 (d, *J* = 6.9 Hz, 3H); ^13^C NMR (125 MHz, CDCl_3_) δ 158.8, 148.4, 146.2, 121.1, 93.2, 61.9, 25.7. HRMS (ESI-TOF) (*m*/z): [M + Na]^+^ calcd for C_7_H_7_ClINNaS^+^, 321.8925; found, 321.8917.

*Ethyl(imino)(3-iodopyridin-2-yl)-^λ^6-sulfanone* (**5c**)

Yellow liquid (132 mg, 89% yield); *R_f_* = 0.5 (Hexane/EtOAc = 3:1); ^1^H NMR (500 MHz, CDCl_3_) δ 8.51 (dd, *J* = 4.6, 1.5 Hz, 1H), 8.32 (dd, *J* = 7.9, 1.5 Hz, 1H), 7.11 (dd, *J* = 7.9, 4.6 Hz, 1H), 3.71 (ddt, *J* = 70.8, 14.2, 7.2 Hz, 2H), 1.43 (t, *J* = 7.4 Hz, 3H); ^13^C NMR (125 MHz, CDCl_3_) δ 159.3, 150.4, 147.1, 126.4, 85.1, 46.5, 7.7. HRMS (ESI-TOF) (*m*/*z*): [M + H]^+^ calcd for C_7_H_10_IN_2_OS^+^, 296.9553; found, 296.9551.

*2-(Ethylthio)-5-(4-methylthiophen-2-yl)pyridine* (**5e**)

Yellow liquid (92 mg, 78% yield); *R_f_* = 0.4 (Hexane/EtOAc = 5:1); ^1^H NMR (500 MHz, CDCl_3_) δ 8.88 (s, 1H), 8.58 (d, *J* = 4.5 Hz, 1H), 8.06 (dt, *J* = 7.9, 2.0 Hz, 1H), 7.39 (dd, *J* = 7.9, 4.9 Hz, 1H), 7.08 (d, *J* = 1.2 Hz, 1H), 2.52 (q, *J* = 7.4 Hz, 3H), 2.36 (d, *J* = 1.0 Hz, 2H), 1.01 (t, *J* = 7.4 Hz, 3H); ^13^C NMR (125 MHz, CDCl_3_) δ 149.3, 147.9, 142.2, 141.9, 137.4, 131.1, 128.9, 123.3, 120.7, 29.9, 16.0, 14.5. HRMS (ESI-TOF) (*m*/*z*): [M + H]^+^ calcd for C_12_H_14_NS_2_^+^, 236.0557; found, 236.0562.

*1,2-dibenzyldisulfane* (**5f**) [61]

White solid (107 mg, 87% yield), MP: 71–72 °C; *R_f_* = 0.5 (Hexane/EtOAc = 20:1); ^1^H NMR (400 MHz, CDCl_3_) δ 7.36–7.29 (m, 5H), 7.29–7.23 (m, 5H), 3.62 (s, 4H); ^13^C NMR (100 MHz, CDCl_3_) δ 137.4 (2C), 129.4 (4C), 128.5 (4C), 127.4 (2C), 43.4 (2C).

## 4. Conclusions

Thus, in this study, a practical, efficient, metal-free protocol was successfully developed for use in synthesizing dialkyl thioethers and alkyl aryl thioether derivatives involving the nucleophilic sulfuration of alkyl halides and aryl halides in the presence of ROCS_2_K, which is a thiol-free sulfur reagent. This efficient, odorless sulfuration reaction provides straightforward access to thioethers, which are biologically relevant in the fields of pharmaceutical chemistry and materials science.

## Figures and Tables

**Table 1:** Optimization of the reaction conditions ^a,b^.

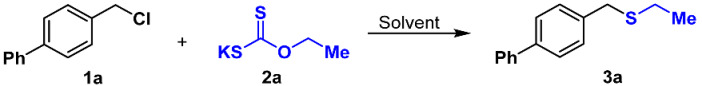					
Entry	EtOCS_2_K (mmol)	Solvent (mL)	T (°C)	T (h)	Yield (%)
1	EtOCS_2_K (1)	DMF (2)	150	6	77
2	EtOCS_2_K (1)	o-xylene (2)	150	6	<5
3	EtOCS_2_K (1)	DMSO (2)	150	6	95
4	EtOCS_2_K (1)	DMAc (2)	150	6	57
5	EtOCS_2_K (1)	NMP (2)	150	6	45
6	EtOCS_2_K (1)	DMSO (1)	150	6	94
7	EtOCS_2_K (0.8)	DMSO (1)	150	6	78
8	EtOCS_2_K (1)	DMSO (1)	130	6	95
9	EtOCS_2_K (1)	DMSO (1)	100	6	94
10	EtOCS_2_K (1)	DMSO (1)	80	6	89
11	EtOCS_2_K (1)	DMSO (1)	100	3	94
12	EtOCS_2_K (1)	DMSO (1)	100	1	93
13	EtOCS_2_K (1)	DMSO (1)	100	0.5	87

^a^ Reaction conditions: 4-(chloromethyl)biphenyl **1a** (0.5 mmol), EtOCS_2_K **2a**, and solvent in a sealed tube in an air atmosphere. ^b^ Isolated yields.

**Table 2:** Optimization of reaction condition ^a,b^.

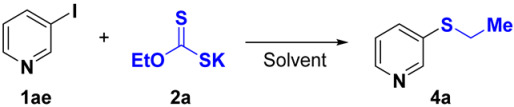					
Entry	EtOCS_2_K (mmol)	Additive (mmol)	Solvent	Time (h)	Yield (%)
1	EtOCS_2_K (1.2)	I_2_ (1.5)	DMF	24	37
2	EtOCS_2_K (1.2)	I_2_ (1.5)	DMF	30	45
3	EtOCS_2_K (1.2)	I_2_ (1.5)	DMF	36	92
4	EtOCS_2_K (0.8)	I_2_ (1.5)	DMF	48	69
5	EtOCS_2_K (1.2)	I_2_ (0.7)	DMF	36	10
6	EtOCS_2_K (1.2)	NH_4_I (1.5)	DMF	36	<5
7	EtOCS_2_K (1.2)	HI (1.5)	DMF	36	15
8	EtOCS_2_K (1.2)	I_2_ (1.5)	DMSO	36	<5
9	EtOCS_2_K (1.2)	I_2_ (1.5)	NMP	36	<5
10	EtOCS_2_K (1.2)	I_2_ (1.5)	DMAc	36	43
11 ^c^	EtOCS_2_K (1.2)	I_2_ (1.5)	DMF	36	39
12	EtOCS_2_K (1.2)	-	DMF	36	<5

^a^ Reaction conditions: 3-Iodine pyridine (0.5 mmol), EtOCS_2_K, and additive in solvent (3.0 mL) at 150 °C in a sealed tube in an air atmosphere. ^b^ Isolated yields. ^c^ 140 °C.

## Data Availability

All data are available from the authors.

## Supplementary Materials

The following supporting information can be downloaded at: https://www.mdpi.com/article/10.3390/molecules29112485/s1, NMR spectra of compounds **3a**–**3af**, **4a**–**4s** and **5a**–**5c**, **5f**.
