# Supplementary material for: Expedient Synthesis of Alkyl and Aryl Thioethers Using Xanthates as Thiol-Free Reagents
Source: Molecules. 2024 May 24;29(11):2485. doi: 10.3390/molecules29112485 (PMC11174007; doi:10.3390/molecules29112485)

Supporting Information

Expedient Synthesis of Alkyl and Aryl Thioethers using Xanthates as Thiol-Free Reagents

Jinli Nie [1], Ziqing He [1], Sijie Xie [1], Yibiao Li [1,*] Runfa He [1], Lu Chen [1] and Xiai Luo [2,*]

[1] Jiangmen Key Laboratory of Synthetic Chemistry and Cleaner Production, School of Environmental & Chemical Engineering, Wuyi University, Jiangmen 529020, China

[2] Hunan Province Key Laboratory for Synthetic Biology of Traditional Chinese Medicine, School of Pharmaceutical Sciences, Hunan University of Medicine, Huaihua, 418000, China

[3] Guangdong Provincial Laboratory of Chemistry and Fine Chemical Engineering Jieyang Center

* Correspondence: luoxiai83@163.com (X. Luo); leeyib268@126.com (Y. Li)

Table of Contents

A. General Information 2

B. General reaction procedures 2

C. Control experiment for detection of COS 3

D. Characterization data for all prepared compounds 4

E. ^{1}H NMR and ^{13}C NMR spectra for products 19

A. General Information

Chemicals and solvents were purchased from commercial suppliers, such as Bidepharm and Energy Chemical, and used as received unless noted. All products were purified by flash chromatography on silica gel. The chemical yields referred are isolated products. ^{1}H NMR and ^{13}C NMR spectra were recorded on 400 MHz, 500MHz and 600 MHz Bruker spectrometers. Chemical shifts of ^{1}H were reported in part per million relative to the $CDCl_3$ residual peak (δ 7.260). Chemical shifts of ^{13}C NMR were reported relative to $CDCl_3$ (δ 77.0). The used abbreviations are as follows: s (singlet), d (doublet), t (triplet), quart. (quartet), quint (quintet), m (multiplet), br (broad). Multiplets which arise from accidental equality of coupling constants of magnetically non-equivalent protons are marked as virtual (*virt.*). High resolution mass spectra (HRMS) data were measured on a ESI-microTOF II. The crystal data were collected by a diffractometer Rigaku Oxford Diffraction Supernova Dual Source, Cu at Zero equipped with an AtlasS2 CCD using Cu Kα radiation (1.54178 Å) by using a w scan mode. Major elements, such as Zn, Cd, Cu, Ni, Cr et al. in reaction system were determined by using Microwave plasma-atomic emission spectrometry (Agilent/4210 MP-AES). Melting points were measured on a SGW® X-4B and are not corrected. Reactions were monitored by TLC analysis using silica gel 60 Å F-254 thin layer plates and compounds were visualized with a UV light at 254 nm or 365 nm.

B. General reaction procedures

General methods for the synthesis of ([1,1'-biphenyl]-4-ylmethyl)(ethyl)sulfane

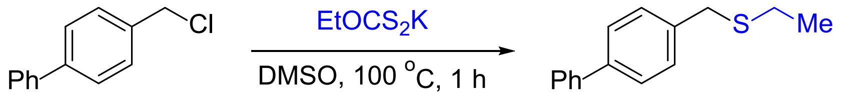

1a **3a**

A mixture of 4-(chloromethyl)-1,1'-biphenyl (101 mg, 0.5 mmol), $EtOCS_2K$ (160 mg, 1.0 mmol) and DMSO (2 mL) was added successively in a 15 mL Schlenk tube. The Schlenk tube was then immersed in an oil bath at 100 °C stirring for 1 h. After cooling down to room temperature, the solution was filtered through a small amount of silica gel. Then the residue was concentrated in vacuo and the crude was purified by flash

chromatography with n-hexane/ethyl acetate (50/1, v/v) to afford the ([1,1'-biphenyl]-4-ylmethyl)(ethyl)sulfane **3a** as a yellow liquid (106 mg, 93%).

General methods for the synthesis of thiophenepyridines

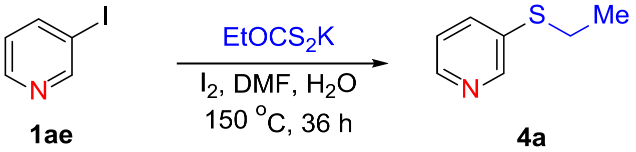

A mixture of 3-Iodine pyridine (103 mg, 0.5 mmol), $EtOCS_2K$ (192 mg, 1.2 mmol), I_2 (381 mg, 1.5 mmol), and DMF (3 mL) was added successively in a 15 mL Schlenk tube. The Schlenk tube was then immersed in an oil bath at 150 °C stirring for 36 h. After cooling down to room temperature, the solution was filtered through a small amount of silica gel. Then the residue was concentrated in vacuo and the crude was purified by flash chromatography with n-hexane/ethyl acetate (3/1, v/v) to afford the 3-(ethylthio)pyridine **4a** as a yellow liquid (64 mg, 92%).

C. Control experiment for detection of COS

A mixture of 3-Iodine pyridine (21 mg, 0.1 mmol), $EtOCS_2K$ (38 mg, 0.25 mmol), I_2 (76 mg, 0.3 mmol), and DMF (2 mL) was added successively in a 10 mL Schlenk tube. The Schlenk tube was then immersed in an oil bath at 150 °C stirring for 24 h. After cooling down to room temperature, the reaction is quenched by the addition of ethyl acetate (10 mL) and saturated brine (5 mL). The organic layer was subsequently stratified and analyzed by GC-MS.

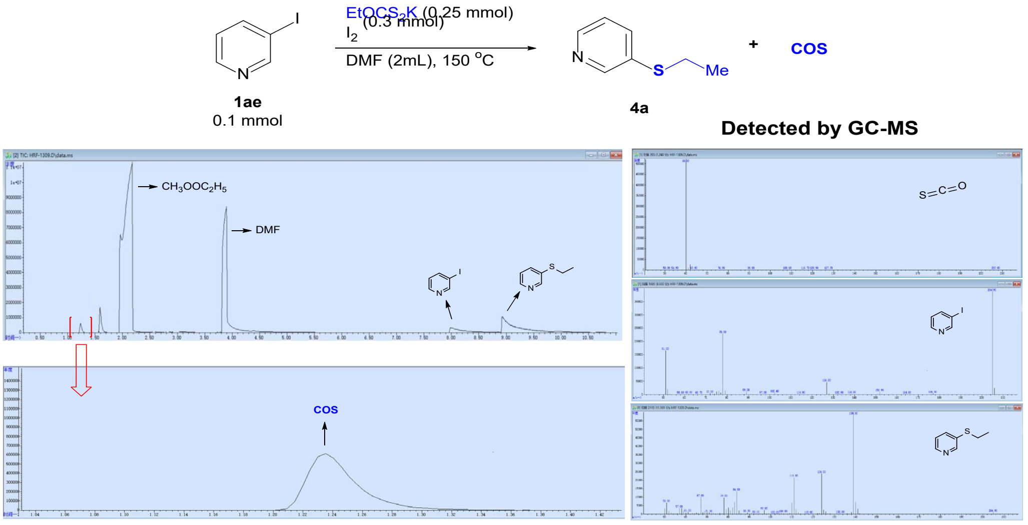

Scheme S1. Detection of COS by GC-MS.

D. Characterization data for all prepared compounds

([1,1'-biphenyl]-4-ylmethyl)(ethyl)sulfane (**3a**)

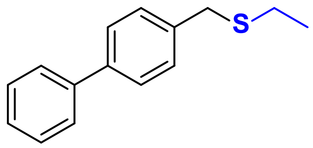

Yellow liquid (106 mg, 93% yield); R_f = 0.6 (Hexane /EtOAc = 50:1); ^{1}H NMR (400 MHz, $CDCl_3$) δ 7.63 – 7.55 (m, 4H), 7.49 – 7.39 (m, 4H), 7.39 – 7.33 (m, 1H), 3.79 (s, 2H), 2.51 (d, J = 7.4 Hz, 2H), 1.29 (t, J = 7.4 Hz, 3H); ^{13}C NMR (100 MHz, $CDCl_3$) δ 140.8, 139.8, 137.7, 129.2 (2C), 128.7 (2C), 127.2, 127.2 (2C), 127.0 (2C), 35.5, 25.2, 14.4; HRMS (ESI-TOF) (m/z): [M+H]$^+$ calcd for $C_{15}H_{17}S^+$, 229.1045; found, 229.1042.

Ethyl(4-methylbenzyl)sulfane (**3b**)

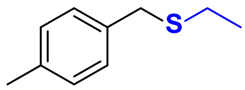

Yellow liquid (76 mg, 92% yield); R_f = 0.6 (Hexane /EtOAc = 50:1); ^{1}H NMR (400 MHz, $CDCl_3$) δ 7.25 – 7.19 (m, 2H), 7.15 – 7.10 (m, 2H), 3.87 (s, 2H), 2.48 (q, J = 7.3 Hz, 2H), 2.34 (s, 3H), 1.24 (t, J = 7.4 Hz, 3H); ^{13}C NMR (100 MHz, $CDCl_3$) δ 137.1, 134.4, 129.2 (2C), 129.1 (2C), 43.5, 32.4, 21.1, 14.3; HRMS (ESI-TOF) (m/z): [M+K]$^+$ calcd for $C_{10}H_{14}KS^+$, 205.0448; found, 205.0445.

(4-(tert-butyl)benzyl)(ethyl)sulfane (**3c**)

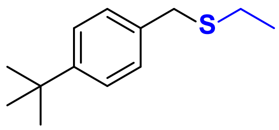

Yellow liquid (99 mg, 95% yield); R_f = 0.6 (Hexane /EtOAc = 50:1); ^{1}H NMR (400 MHz, $CDCl_3$) δ 7.37 – 7.32 (m, 2H), 7.28 – 7.23 (m, 2H), 3.71 (s, 2H), 2.47 (q, J = 7.4 Hz, 2H), 1.33 (s, 9H), 1.26 (t, J = 7.4 Hz, 3H); ^{13}C NMR (100 MHz, $CDCl_3$) δ 149.7, 135.5, 128.4 (2C), 125.3 (2C), 35.4, 34.4, 31.3, 25.3, 14.3; HRMS (ESI-TOF) (m/z): [M+K]$^+$ calcd for $C_{13}H_{20}KS^+$, 247.0917; found, 247.0914.

Ethyl(2,4,6-trimethylbenzyl)sulfane (**3d**)

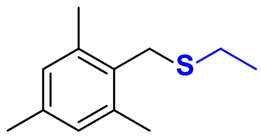

Yellow liquid (73 mg, 75% yield); R_f = 0.6 (Hexane /EtOAc = 50:1); ^{1}H NMR (400 MHz, $CDCl_3$) δ 6.85 (s, 2H), 3.78 (s, 2H), 2.61 (q, J = 7.4 Hz, 2H), 2.40 (s, 6H), 2.27 (s, 3H), 1.33 (t, J = 7.4 Hz, 3H); ^{13}C NMR (100 MHz, $CDCl_3$) δ 136.8 (2C), 136.3, 131.4, 129.0 (2C), 30.5, 26.8, 20.9, 19.6 (2C), 14.8; HRMS (ESI-TOF) (m/z): [M+H]$^+$ calcd for $C_{12}H_{19}S^+$, 195.1202; found, 195.1207.

Ethyl(3-methoxybenzyl)sulfane (**3e**)

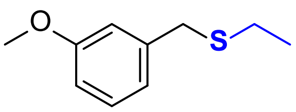

Yellow liquid (73 mg, 80% yield); R_f= 0.4 (Hexane /EtOAc = 10:1); ^{1}H NMR (400 MHz, $CDCl_3$) δ 7.22 (t, J = 7.8 Hz, 1H), 6.94 – 6.85 (m, 2H), 6.82 – 6.76 (m, 1H), 3.81 (s, 3H), 3.70 (s, 2H), 2.45 (q, J = 7.4 Hz, 2H), 1.24 (t, J = 7.4 Hz, 3H); ^{13}C NMR (100 MHz, $CDCl_3$) δ 159.7, 140.2, 129.4, 121.2, 114.3, 112.4, 55.2, 35.9, 25.3, 14.3; HRMS (ESI-TOF) (*m/z*): $[M+K]^+$ calcd for $C_{10}H_{14}KOS^+$, 221.0397; found, 221.0395.

(4-(benzyloxy)benzyl)(ethyl)sulfane (**3f**)

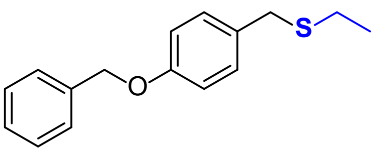

Yellow solid (120 mg, 93% yield), MP: 61-62 °C; R_f= 0.4 (Hexane /EtOAc = 20:1); ^{1}H NMR (400 MHz, $CDCl_3$) δ 7.46 – 7.37 (m, 4H), 7.36 – 7.30 (m, 1H), 7.26 – 7.21 (m, 2H), 6.95 – 6.90 (m, 2H), 5.06 (s, 2H), 3.69 (s, 2H), 2.44 (q, J = 7.2 Hz, 2H), 1.24 (t, J = 7.4 Hz, 3H); ^{13}C NMR (100 MHz, $CDCl_3$) δ 157.7, 137.0, 130.9, 129.8 (2C), 128.6 (2C), 127.9, 127.4 (2C), 114.8 (2C), 79.7 – 74.5 (m), 70.0, 35.2, 25.1, 14.4; HRMS (ESI-TOF) (*m/z*): $[M+H]^+$ calcd for $C_{16}H_{19}OS^+$, 259.1151; found, 259.1149.

Ethyl(4-(trifluoromethoxy)benzyl)sulfane (**3g**)

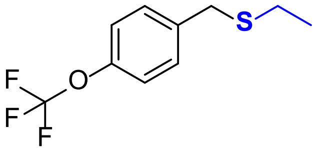

Yellow liquid (111 mg, 94% yield); R_f= 0.5 (Hexane /EtOAc = 50:1); ^{1}H NMR (400 MHz, $CDCl_3$) δ 7.34 (d, J = 8.3 Hz, 2H), 7.15 (d, J = 8.2 Hz, 2H), 3.71 (s, 2H), 2.44 (q, J = 7.4 Hz, 2H), 1.23 (t, J = 7.4 Hz, 3H); ^{13}C NMR (100 MHz, $CDCl_3$) δ 148.1 (q, J = 1.8 Hz), 137.4, 130.1 (2C), 121.0 (2C), 120.5 (q, J = 204.2 Hz), 35.1, 25.3, 14.3; ^{19}F NMR (376 MHz, $CDCl_3$) δ -57.86; HRMS (ESI-TOF) (*m/z*): $[M+Na]^+$ calcd for $C_{10}H_{11}F_3NaOS^+$, 259.0375; found, 259.0371.

Ethyl(4-((trifluoromethyl)thio)benzyl)sulfane (**3h**)

Yellow liquid (166 mg, 92% yield); R_f= 0.6 (Hexane /EtOAc = 50:1); ^{1}H NMR (400 MHz, $CDCl_3$) δ 7.63 – 7.56 (m, 2H), 7.40 – 7.34 (m, 2H), 3.73 (s, 2H), 2.44 (q, J = 7.4 Hz, 2H), 1.23 (t, J = 7.4 Hz, 3H); ^{13}C NMR (100 MHz, $CDCl_3$) δ 142.0, 136.4 (2C), 129.9 (2C), 129.5 (q, J = 306.1 Hz), 122.6 (q, J = 2.3 Hz), 35.4, 25.4, 14.3; ^{19}F NMR (376 MHz, Chloroform-*d*) δ -42.58; HRMS (ESI-TOF) (*m/z*): $[M+H]^+$ calcd for $C_{10}H_{12}F_3S_2^+$, 253.0327; found, 253.0320.

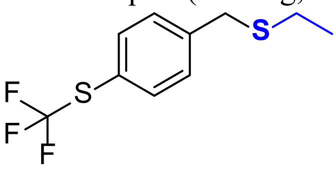

Ethyl(4-(phenylthio)benzyl)sulfane (**3i**)

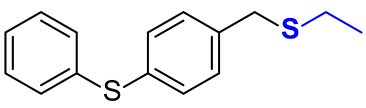

Yellow liquid (121 mg, 93% yield); R_f = 0.5 (Hexane /EtOAc = 50:1); ^{1}H NMR (400 MHz, $CDCl_3$) δ 7.37 – 7.21 (m, 9H), 3.70 (s, 2H), 2.45 (q, J = 7.4 Hz, 2H), 1.24 (t, J = 7.4 Hz, 3H); ^{13}C NMR (100 MHz, $CDCl_3$) δ 137.7, 135.8, 134.0, 131.2 (4C), 130.8 (4C), 129.6 (4C), 129.1 (4C), 126.9, 35.4, 25.3, 14.3; HRMS (ESI-TOF) (*m/z*): $[M+Na]^+$ calcd for $C_{15}H_{16}NaS_2^+$, 283.0586; found, 283.0593.

(4-((ethylthio)methyl)phenyl)methanol (**3j**)

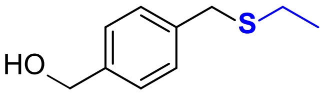

Yellow liquid (82 mg, 90% yield); R_f = 0.5 (Hexane /EtOAc = 2:1); ^{1}H NMR (400 MHz, $CDCl_3$) δ 7.33 – 7.27 (m, 4H), 4.65 (s, 2H), 3.71 (s, 2H), 2.42 (q, J = 7.3 Hz, 2H), 1.91 (s, 1H), 1.22 (t, J = 7.4 Hz, 3H); ^{13}C NMR (100 MHz, $CDCl_3$) δ 139.5, 138.0, 128.9 (2C), 127.1 (2C), 65.0, 35.5, 25.2, 14.3; HRMS (ESI-TOF) (*m/z*): $[M+K]^+$ calcd for $C_{10}H_{14}KOS^+$, 221.0397; found, 221.0395.

2-(4-((ethylthio)methyl)phenyl)-4,4,5,5-tetramethyl-1,3,2-dioxaborolane (**3k**)

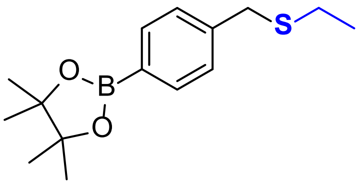

Yellow liquid (127 mg, 91% yield); R_f = 0.5 (Hexane /EtOAc = 20:1); ^{1}H NMR (400 MHz, $CDCl_3$) δ 7.78 – 7.73 (m, 2H), 7.36 – 7.30 (m, 2H), 3.72 (s, 2H), 2.41 (q, J = 7.4 Hz, 2H), 1.34 (s, 12H), 1.21 (t, J = 7.4 Hz, 3H); ^{13}C NMR (100 MHz, $CDCl_3$) δ 141.9, 141.8, 134.9 (2C), 128.2 (2C), 83.7 (2C), 35.9, 25.1, 24.8 (4C), 14.3; HRMS (ESI-TOF) (*m/z*): $[M+H]^+$ calcd for $C_{15}H_{24}BO_2S^+$, 278.1621; found, 278.1624.

Ethyl(4-fluorobenzyl)sulfane (**3l**)

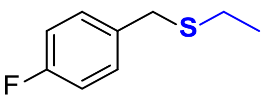

Yellow liquid (81 mg, 95% yield); R_f = 0.6 (Hexane /EtOAc = 50:1); ^{1}H NMR (400 MHz, $CDCl_3$) δ 7.31 – 7.25 (m, 2H), 7.03 – 6.95 (m, 2H), 3.69 (s, 2H), 2.43 (q, J = 7.3 Hz, 2H), 1.23 (t, J = 7.4 Hz, 3H); ^{13}C NMR (100 MHz, $CDCl_3$) δ 161.8 (d, J = 245.2 Hz), 134.3 (d, J = 3.0 Hz), 130.3 (d, J = 8.0 Hz, 2C), 115.3 (d, J = 21.4 Hz, 2C), 35.1, 25.2, 14.3; ^{19}F NMR (376 MHz, $CDCl_3$) δ -112.92 (ddd, J = 13.9, 8.6, 5.2 Hz); HRMS (ESI-TOF) (*m/z*): $[M+Na]^+$ calcd for $C_9H_{11}FNaS^+$, 193.0458; found, 193.0466.

(4-chlorobenzyl)(ethyl)sulfane (**3m**)

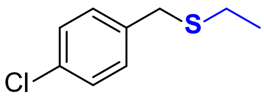

Yellow liquid (84 mg, 90% yield); R_f = 0.6 (Hexane /EtOAc = 50:1); ^{1}H NMR (400 MHz, $CDCl_3$) δ 7.30 – 7.26 (m, 2H), 7.24 (d, J = 8.7 Hz, 2H), 3.68 (s, 2H), 2.42 (q, J = 7.4 Hz, 2H), 1.22 (t, J = 7.4 Hz, 3H); ^{13}C NMR (100 MHz, $CDCl_3$) δ 137.1, 132.6, 130.1 (2C), 128.6 (2C), 35.2, 25.2, 14.3; HRMS (ESI-TOF) (*m/z*): [M+Na]$^+$ calcd for $C_9H_{11}ClNaS^+$, 209.0162; found, 209.0152.

(4-bromobenzyl)(ethyl)sulfane (**3n**)

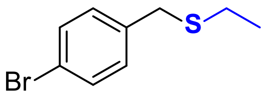

Yellow liquid (106 mg, 92% yield); R_f = 0.6 (Hexane /EtOAc = 50:1); ^{1}H NMR (400 MHz, $CDCl_3$) δ 7.47 – 7.39 (m, 2H), 7.22 – 7.16 (m, 2H), 3.66 (s, 2H), 2.42 (q, J = 7.4 Hz, 2H), 1.22 (t, J = 7.4 Hz, 3H); ^{13}C NMR (100 MHz, $CDCl_3$) δ 137.7, 131.5 (2C), 130.5 (2C), 120.6, 35.2, 25.2, 14.3; HRMS (ESI-TOF) (*m/z*): [M+H]$^+$ calcd for $C_9H_{12}BrS^+$, 230.9838; found, 230.9828.

Ethyl(4-Iodobenzyl)sulfane (**3o**)

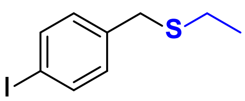

Yellow liquid (132 mg, 95% yield); R_f = 0.6 (Hexane /EtOAc = 50:1); ^{1}H NMR (400 MHz, $CDCl_3$) δ 7.67 – 7.58 (m, 2H), 7.10 – 7.03 (m, 2H), 3.65 (s, 2H), 2.41 (q, J = 7.4 Hz, 2H), 1.22 (t, J = 7.4 Hz, 3H); ^{13}C NMR (100 MHz, $CDCl_3$) δ 138.3, 137.5 (2C), 130.8 (2C), 92.1, 35.3, 25.2, 14.3; HRMS (ESI-TOF) (*m/z*): [M+H]$^+$ calcd for $C_9H_{12}IS^+$, 278.9699; found, 278.9693.

Ethyl(4-(trifluoromethyl)benzyl)sulfane (**3p**)

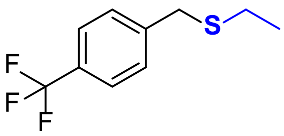

Yellow liquid (103 mg, 94% yield); R_f = 0.6 (Hexane /EtOAc = 50:1); ^{1}H NMR (400 MHz, $CDCl_3$) δ 7.57 (d, J = 8.0 Hz, 2H), 7.43 (d, J = 8.0 Hz, 2H), 3.75 (s, 2H), 2.43 (q, J = 7.4 Hz, 2H), 1.24 (t, J = 7.4 Hz, 3H); ^{13}C NMR (100 MHz, $CDCl_3$) δ 142.9, 129.1 (2C), 129.1 (q, J = 31.7 Hz), 125.4 (q, J = 3.8 Hz, 2C), 124.2 (q, J = 269.9 Hz), 35.4, 25.3, 14.2; ^{19}F NMR (376 MHz, $CDCl_3$) δ -62.71; HRMS (ESI-TOF) (*m/z*): [M+Na]$^+$ calcd for $C_{10}H_{11}F_3NaOS^+$, 259.0375; found, 259.0371.

Ethyl(4-(methylsulfonyl)benzyl)sulfane (**3q**)

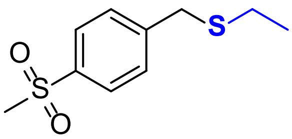

Yellow liquid (105 mg, 91% yield); R_f = 0.4 (Hexane /EtOAc = 1:1); ^{1}H NMR (400 MHz, $CDCl_3$) δ 7.91 – 7.82 (m, 2H), 7.54 – 7.46 (m, 2H), 3.75 (s, 2H), 3.03 (s, 3H), 2.42 (q, J = 7.4 Hz, 2H), 1.21 (t, J = 7.4 Hz, 3H); ^{13}C NMR (100 MHz, $CDCl_3$) δ 145.3, 138.9, 129.6

(2C), 127.5 (2C), 44.4, 35.4, 25.4, 14.2; HRMS (ESI-TOF) (*m/z*): $[M+H]^+$ calcd for $C_{10}H_{15}O_2S_2^+$, 231.0508; found, 231.0503.

4-((ethylthio)methyl)-N-phenylbenzamide (**3r**)

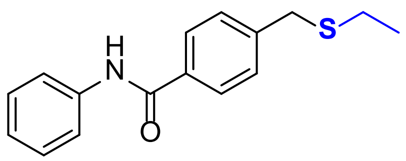

White solid (126 mg, 93% yield), MP: 118-120 ºC; R_f = 0.4 (Hexane /EtOAc = 3:1); ^{1}H NMR (400 MHz, $CDCl_3$) δ 8.08 (s, 1H), 7.83 – 7.76 (m, 2H), 7.66 – 7.61 (m, 2H), 7.40 – 7.30 (m, 4H), 7.16 – 7.10 (m, 1H), 3.74 (s, 2H), 2.50 – 2.36 (m, 2H), 1.23 (t, J = 7.4 Hz, 3H); ^{13}C NMR (100 MHz, $CDCl_3$) δ 165.6, 142.8, 137.9, 133.5, 129.1 (2C), 129.0 (2C), 127.3 (2C), 124.5, 120.3 (2C), 35.5, 25.3, 14.3; HRMS (ESI-TOF) (*m/z*): $[M+H]^+$ calcd for $C_{16}H_{18}NOS^+$, 272.1104; found, 272.1098.

Ethyl(naphthalen-1-ylmethyl)sulfane (**3s**)

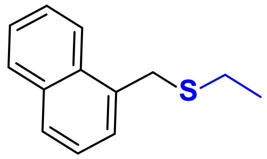

Yellow liquid (94 mg, 93% yield); R_f= 0.6 (Hexane /EtOAc = 50:1); ^{1}H NMR (400 MHz, $CDCl_3$) δ 8.16 (d, J = 8.3 Hz, 1H), 7.89 – 7.84 (m, 1H), 7.81 – 7.73 (m, 1H), 7.59 – 7.53 (m, 1H), 7.52 – 7.47 (m, 1H), 7.44 – 7.35 (m, 2H), 4.19 (s, 2H), 2.51 (q, J = 7.3 Hz, 2H), 1.28 (t, J = 7.4 Hz, 3H); ^{13}C NMR (100 MHz, $CDCl_3$) δ 134.1, 133.9, 131.4, 128.8, 128.0, 126.9, 126.1, 125.8, 125.1, 124.1, 33.7, 26.0, 14.4; HRMS (ESI-TOF) (*m/z*): $[M]^+$ calcd for $C_{13}H_{14}S^+$, 202.0811; found, 202.0816.

Ethyl(naphthalen-2-ylmethyl)sulfane (**3t**)

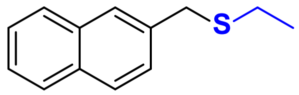

Yellow liquid (96 mg, 95% yield); R_f= 0.6 (Hexane /EtOAc = 50:1); ^{1}H NMR (400 MHz, $CDCl_3$) δ 7.87 – 7.79 (m, 3H), 7.72 (s, 1H), 7.55 – 7.44 (m, 3H), 3.90 (s, 2H), 2.46 (q, J = 7.4 Hz, 2H), 1.26 (t, J = 7.4 Hz, 3H); ^{13}C NMR (100 MHz, $CDCl_3$) δ 135.9, 133.2, 132.5, 128.3, 127.6, 127.6, 127.1, 126.1, 125.6, 36.1, 25.1, 14.3; HRMS (ESI-TOF) (*m/z*): $[M]^+$ calcd for $C_{13}H_{14}S^+$, 202.0811; found, 202.0818.

(Anthracen-9-ylmethyl)(ethyl)sulfane (**3u**)

Yellow solid (53 mg, 42% yield), MP: 68-70 ºC; R_f = 0.6 (Hexane /EtOAc = 50:1); ^{1}H NMR (400 MHz, $CDCl_3$) δ 8.39 (s, 1H), 8.35 (d, J = 8.9 Hz, 2H), 8.01 (d, J = 8.4 Hz, 2H), 7.61 – 7.53 (m, 2H), 7.52 – 7.44 (m, 2H), 4.75 (s, 2H), 2.70 (q, J = 7.4 Hz, 2H), 1.37 (t, J = 7.4 Hz, 3H); ^{13}C NMR (100 MHz, $CDCl_3$) δ 131.5 (2C), 129.9 (2C), 129.5, 129.2 (2C), 127.2, 126.0 (2C), 125.0 (2C), 124.2 (2C), 28.7, 27.1, 14.8; HRMS (ESI-TOF) (*m/z*): $[M+H]^+$ calcd for $C_{17}H_{17}S^+$, 253.1045; found, 253.1045.

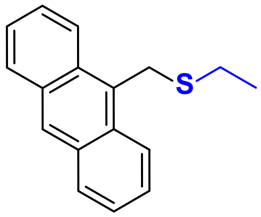

2-chloro-5-((ethylthio)methyl)thiophene (**3v**)

Yellow liquid (89 mg, 93% yield); R_f = 0.6 (Hexane /EtOAc = 50:1); ^{1}H NMR (400 MHz, $CDCl_3$) δ 6.73 – 6.66 (m, 2H), 3.82 (s, 2H), 2.51 (q, J = 7.4 Hz, 2H), 1.24 (t, J = 7.4 Hz, 3H); ^{13}C NMR (100 MHz, $CDCl_3$) δ 141.4, 128.8, 125.5, 125.0, 30.4, 25.4, 14.2; HRMS (ESI-TOF) (*m/z*): $[M+H]^+$ calcd for $C_7H_{10}ClS_2^+$, 192.9907; found, 192.9902.

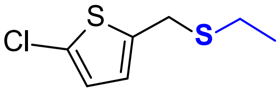

5-chloro-3-((ethylthio)methyl)benzo[b]thiophene (**3w**)

Yellow liquid (111 mg, 92% yield); R_f = 0.6 (Hexane /EtOAc = 50:1); ^{1}H NMR (400 MHz, $CDCl_3$) δ 7.88 – 7.84 (m, 1H), 7.74 (d, J = 8.6 Hz, 1H), 7.35 – 7.29 (m, 2H), 3.92 (s, 3H), 2.48 (q, J = 7.4 Hz, 2H), 1.26 (t, J = 7.4 Hz, 3H); ^{13}C NMR (100 MHz, $CDCl_3$) δ 139.1, 138.7, 131.9, 130.4, 125.4, 124.9, 123.8, 121.9, 28.9, 25.7, 14.2; HRMS (ESI-TOF) (*m/z*): $[M+Na]^+$ calcd for $C_{11}H_{11}ClNaS_2^+$, 264.9883; found, 264.9884.

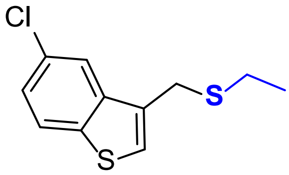

8-((ethylthio)methyl)quinoline (**3x**)

Yellow liquid (92 mg, 91% yield); R_f = 0.4 (Hexane /EtOAc = 10:1); ^{1}H NMR (400 MHz, $CDCl_3$) δ 8.97 – 8.93 (m, 1H), 8.16 – 8.09 (m, 1H), 7.71 (d, J = 7.7 Hz, 2H), 7.52 – 7.45 (m, 1H), 7.42 – 7.37 (m, 1H), 4.44 (s, 2H), 2.56 (q, J = 7.4 Hz, 2H), 1.28 (t, J = 7.4 Hz, 3H); ^{13}C NMR (100 MHz, $CDCl_3$) δ 149.6, 146.3, 137.3, 136.3, 129.3, 128.5, 127.0, 126.1, 121.1, 31.1, 26.1, 14.5; HRMS (ESI-TOF) (*m/z*): $[M+H]^+$ calcd for $C_{12}H_{14}NS^+$, 204.0842; found, 204.0843.

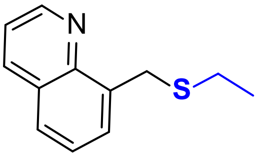

2-((ethylthio)methyl)-4-methylquinazoline (**3y**)

Yellow solid (95 mg, 87% yield), MP: 52-54 ºC; R_f = 0.6 (Hexane /EtOAc = 50:1); ^{1}H NMR (400 MHz, $CDCl_3$) δ 8.03 – 7.98 (m, 1H), 7.95 – 7.89 (m, 1H), 7.82 – 7.76 (m, 1H), 7.56 – 7.50 (m, 1H), 4.01 (s, 2H), 2.89 (s, 3H), 2.62 (q, J = 7.4 Hz, 2H), 1.24 (t, J = 7.4 Hz, 3H); ^{13}C NMR (100 MHz, $CDCl_3$) δ 168.8, 163.6, 149.6, 133.5, 128.5, 126.9, 124.8, 122.5, 39.1, 25.9, 21.7, 14.4; HRMS (ESI-TOF) (m/z): $[M+H]^+$ calcd for $C_{12}H_{15}N_2S^+$, 219.0950; found, 219.0942.

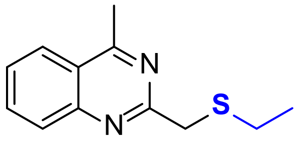

1-(4-((ethylthio)methyl)phenyl)-1H-pyrazole (**3z**)

Yellow liquid (93 mg, 85% yield); R_f = 0.5 (Hexane /EtOAc = 10:1); ^{1}H NMR (400 MHz, $CDCl_3$) δ 7.95 – 7.88 (m, 1H), 7.74 – 7.70 (m, 1H), 7.64 (d, J = 8.3 Hz, 2H), 7.45 – 7.37 (m, 2H), 3.75 (s, 2H), 2.45 (q, J = 7.3, 6.8 Hz, 2H), 1.24 (t, J = 7.3 Hz, 3H); ^{13}C NMR (100 MHz, $CDCl_3$) δ 141.0, 139.0, 136.9, 129.8 (2C), 126.7, 119.3 (2C), 107.6, 35.3, 25.2, 14.4; HRMS (ESI-TOF) (m/z): $[M+H]^+$ calcd for $C_{12}H_{15}N_2S^+$, 219.0950; found, 219.0946.

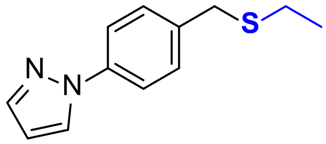

1-cyclohexyl-5-(4-(ethylthio)butyl)-1H-tetrazole (**3aa**)

Yellow liquid (123 mg, 92% yield); R_f = 0.4 (Hexane /EtOAc = 2:1); ^{1}H NMR (400 MHz, $CDCl_3$) δ 4.21 – 4.04 (m, 1H), 2.85 (t, 2H), 2.61 – 2.48 (m, 3H), 2.09 – 1.87 (m, 8H), 1.82 – 1.66 (m, 4H), 1.48 – 1.30 (m, 3H), 1.24 (t, J = 7.4 Hz, 3H); ^{13}C NMR (100 MHz, $CDCl_3$) δ 153.5, 57.6, 32.9 (2C), 30.9, 28.6, 26.2, 25.9, 25.3 (2C), 24.8, 22.9, 14.8; HRMS (ESI-TOF) (m/z): $[M+H]^+$ calcd for $C_{13}H_{25}N_4S^+$, 269.1794; found, 269.1790.

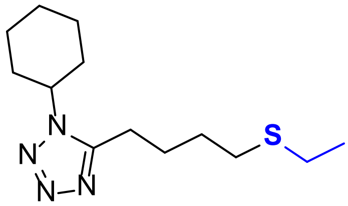

1,4-bis((ethylthio)methyl)benzene (**3ab**)

Yellow liquid (99 mg, 88% yield); R_f = 0.5 (Hexane /EtOAc = 50:1); ^{1}H NMR (400 MHz, $CDCl_3$) δ 7.25 (s, 4H), 3.70 (s, 4H), 2.43 (q, J = 7.4 Hz, 4H), 1.23 (t, J = 7.4 Hz, 6H); ^{13}C NMR (100 MHz, $CDCl_3$) δ 137.2 (2C), 128.9 (4C), 35.5, 25.2, 14.4; HRMS (ESI-TOF) (m/z): $[M]^+$ calcd for $C_{12}H_{18}S_2^+$, 226.0845; found, 226.0836.

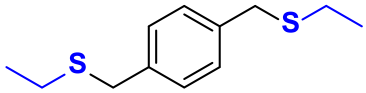

(((1s,3s)-adamantan-1-yl)methyl)(ethyl)sulfane (**3ac**)

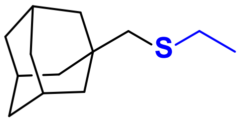

Yellow liquid (42 mg, 40% yield); R_f = 0.6 (Hexane /EtOAc = 50:1); ^{1}H NMR (400 MHz, $CDCl_3$) δ 2.51 (q, J = 7.4 Hz, 2H), 2.32 (s, 2H), 1.97 (s, 3H), 1.73 – 1.66 (m, 3H), 1.65 – 1.59 (m, 3H), 1.58 – 1.54 (m, 6H), 1.24 (t, J = 7.4 Hz, 3H); ^{13}C NMR (100 MHz, $CDCl_3$) δ 47.3, 41.9 (3C), 36.9 (3C), 33.9, 28.6 (3C), 28.2, 15.0; HRMS (ESI-TOF) (*m/z*): $[M+K]^+$ calcd for $C_{13}H_{22}KS^+$, 249.1074; found, 249.1075.

Benzhydryl(ethyl)sulfane (**3ad**)

Yellow liquid (80 mg, 70% yield); R_f = 0.6 (Hexane /EtOAc = 50:1); ^{1}H NMR (400

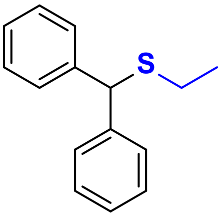

MHz, $CDCl_3$) δ 7.50 – 7.44 (m, 4H), 7.38 – 7.31 (m, 4H), 7.30 – 7.21 (m, 2H), 5.22 (s, 1H), 2.44 (q, J = 7.4 Hz, 2H), 1.25 (t, J = 7.4 Hz, 3H); ^{13}C NMR (100 MHz, $CDCl_3$) δ 141.5 (2C), 128.5 (4C), 128.2 (4C), 127.0 (2C), 53.7, 26.2, 14.2; HRMS (ESI-TOF) (*m/z*): $[M+H]^+$ calcd for $C_{15}H_{17}S^+$, 229.1045; found, 229.1046.

Dibenzylsulfane (**3ae**) [1]

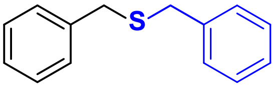

White solid (100 mg, 93% yield), MP: 61-62 °C; *Rf* = 0.6 (Hexane/EtOAc = 50:1); ^{1}H NMR (400 MHz, CDCl3) δ 7.36 – 7.22 (m, 10H), 3.61 (s, 4H); ^{13}C NMR (100 MHz, CDCl3) δ 138.1 (2C), 129.0 (4C), 128.5 (4C), 127.0 (2C), 35.6 (2C).

benzyl(phenyl)sulfane (**3af**) [2]

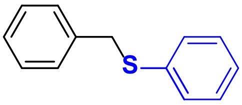

White solid (90 mg, 90% yield), MP: 39-40 °C; *Rf* = 0.6 (Hexane/EtOAc = 50:1); ^{1}H NMR (400 MHz, CDCl3) δ 7.34 – 7.22 (m, 9H), 7.21 – 7.15 (m, 1H), 4.13 (s, 2H); ^{13}C NMR (100 MHz, CDCl3) δ 137.4, 136.3, 129.8 (2C), 128.8 (2C), 128.8 (2C), 128.5 (2C), 127.2, 126.3, 39.0.

Benzyl(ethyl)sulfane (**3ag**)

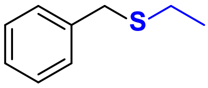

Yellow liquid (27 mg, 36% yield); R_f = 0.6 (Hexane /EtOAc = 50:1); ^{1}H NMR (500 MHz, $CDCl_3$) δ 7.35 – 7.28 (m, 4H), 7.26 – 7.21 (m, 1H), 3.73 (s, 2H), 2.44 (q, J = 7.4 Hz, 2H), 1.23 (t, J = 7.4 Hz, 3H); ^{13}C NMR (125 MHz, $CDCl_3$) δ 138.6, 128.8 (2C), 128.4 (2C), 126.8, 35.8, 25.2, 14.3; HRMS (ESI-TOF) (*m/z*): $[M+H]^+$ calcd for $C_9H_{13}S^+$, 153.0732; found, 153.0735.

Benzyl(butyl)sulfane (**3ah**)

Yellow liquid (33 mg, 37% yield); R_f = 0.6 (Hexane /EtOAc = 50:1); ^{1}H NMR (400

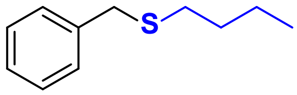

MHz, $CDCl_3$) δ 7.35 – 7.25 (m, 4H), 7.28 – 7.19 (m, 1H), 3.71 (s, 2H), 2.49 – 2.35 (m, 2H), 1.60 – 1.49 (m, 2H), 1.38 (dt, J = 8.1, 7.0 Hz, 2H), 0.89 (t, J = 7.3 Hz, 3H); ^{13}C NMR (100 MHz, $CDCl_3$) 138.7, 128.8 (2C), 128.4 (2C), 126.8, 36.2, 31.3, 31.0, 22.0, 13.7; HRMS (ESI-TOF) (m/z): $[M+H]^+$ calcd for $C_{11}H_{17}S^+$, 181.1045; found, 181.1050.

3-(Ethylthio)pyridine (**4a**)[3]

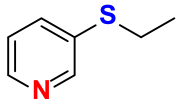

Yellow liquid (64 mg, 92 % yield); R_f = 0.5 (Hexane/EtOAc = 3:1); ^{1}H NMR (500 MHz, $CDCl_3$) δ 8.56 (s, 1H), 8.41 (d, J = 4.8 Hz, 1H), 7.66 (ddd, J = 8.0, 2.4, 1.5 Hz, 1H), 7.23 (dd, J = 8.0, 4.8 Hz, 1H), 2.96 (q, J = 7.4 Hz, 2H), 1.32 (t, J = 7.4 Hz, 3H); ^{13}C NMR (125 MHz, $CDCl_3$) δ 149.6, 146.5, 137.1, 134.0, 123.7, 27.7, 14.3.

3-(Propylthio)pyridine (**4b**)[4]

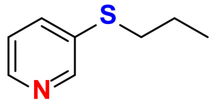

Yellow liquid (58 mg, 75%); R_f = 0.5 (Hexane/EtOAc = 3:1); ^{1}H NMR (500 MHz, $CDCl_3$) δ 8.56 (d, J = 1.8 Hz, 1H), 8.48 – 8.35 (m, 1H), 7.69 – 7.60 (m, 1H), 7.21 (dd, J = 7.9, 4.8 Hz, 1H), 2.96 – 2.74 (m, 2H), 1.67 (h, J = 7.3 Hz, 2H), 1.03 (t, J = 7.4 Hz, 3H); ^{13}C NMR (125 MHz, $CDCl_3$) δ 149.9, 146.7, 136.8, 134.1, 123.6, 35.7, 22.4, 13.3.

3-(Butylthio)pyridine (**4c**)[5]

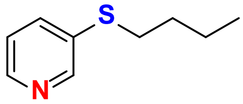

Yellow liquid (62 mg, 74%); R_f = 0.5 (Hexane/EtOAc = 3:1); ^{1}H NMR (500 MHz, $CDCl_3$) δ 8.55 (s, 1H), 8.41 (d, J = 4.2 Hz, 1H), 7.66 (dt, J = 8.0, 1.8 Hz, 1H), 7.24 (dd, J = 7.9, 4.8 Hz, 1H), 2.97 – 2.90 (m, 2H), 1.63 (p, J = 7.4 Hz, 2H), 1.45 (dq, J = 14.6, 7.3 Hz, 2H), 0.92 (t, J = 7.3 Hz, 3H); ^{13}C NMR (125 MHz, $CDCl_3$) δ 149.2, 146.2, 137.0, 134.6, 123.7, 33.3, 31.1, 21.8, 13.6.

3-(Pentylthio)pyridine (**4d**)

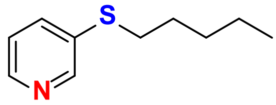

Yellow liquid (67 mg, 74%); R_f = 0.5 (Hexane/EtOAc = 3:1); ^{1}H NMR (500 MHz, $CDCl_3$) δ 8.55 (s, 1H), 8.41 (s, 1H), 7.66 (d, J = 7.9 Hz, 1H), 7.24 – 7.21 (m, 1H), 2.92 (t, J = 7.4 Hz, 2H), 1.64 (p, J = 7.4 Hz, 2H), 1.40 (dt, J = 14.3, 6.9 Hz, 2H), 1.32 (dq, J = 14.3, 6.9 Hz, 2H), 0.89 (t, J = 7.2 Hz, 3H); ^{13}C NMR (125 MHz, $CDCl_3$) δ 149.4, 146.4, 136.9, 134.4, 123.6, 33.6, 30.8, 28.7,

22.2, 13.9. HRMS (ESI-TOF) (m/z): $[M+H]^+$ calcd for $C_{10}H_{16}NS^+$, 182.0998; found, 182.0995.

3-(Ethylthio)quinoline (**4e**)[6]

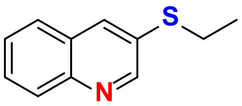

Yellow liquid (89 mg, 94% yield); R_f = 0.4 (Hexane/EtOAc = 5:1); ^{1}H NMR (500 MHz, $CDCl_3$) δ 8.84 (d, J = 2.3 Hz, 1H), 8.10 (d, J = 8.4 Hz, 1H), 8.07 (d, J = 2.3 Hz, 1H), 7.75 (dd, J = 8.2, 1.4 Hz, 1H), 7.68 (ddd, J = 8.4, 6.9, 1.4 Hz, 1H), 7.56 (ddd, J = 8.2, 6.9, 1.2 Hz, 1H), 3.06 (q, J = 7.4 Hz, 2H), 1.37 (t, J = 7.4 Hz, 3H)； ^{13}C NMR (125 MHz, $CDCl_3$) δ 151.6, 146.2, 134.8, 130.6, 129.3, 129.0, 128.2, 127.2, 126.9, 27.9, 14.3.

4-(Ethylthio)isoquinoline (**4f**)[6]

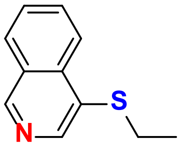

Yellow liquid (80 mg, 85% yield); R_f = 0.4 (Hexane/EtOAc = 5:1); ^{1}H NMR (500 MHz, $CDCl_3$) δ 9.12 (s, 1H), 8.55 (s, 1H), 8.31 (d, J = 8.4 Hz, 1H), 7.98 (d, J = 8.1 Hz, 1H), 7.77 (ddd, J = 8.3, 6.8, 1.3 Hz, 1H), 7.64 (ddd, J = 8.1, 6.8, 1.1 Hz, 1H), 3.01 (q, J = 7.3 Hz, 2H), 1.32 (t, J = 7.4 Hz, 3H); ^{13}C NMR (125 MHz, $CDCl_3$) δ 151.0, 143.7, 135.9, 130.9, 128.7, 128.4, 128.2, 127.67, 124.1, 28.3, 14.5.

2-(Ethylthio)pyridine (**4g**)[7]

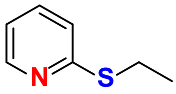

Yellow liquid (63 mg, 90% yield); R_f = 0.4 (Hexane/EtOAc = 5:1); ^{1}H NMR (500 MHz, $CDCl_3$) δ 8.41 (ddd, J = 5.0, 1.9, 1.0 Hz, 1H), 7.50 – 7.41 (m, 1H), 7.15 (dt, J = 8.1, 1.1 Hz, 1H), 6.95 (ddd, J = 7.3, 4.9, 1.1 Hz, 1H), 3.16 (q, J = 7.4 Hz, 2H), 1.36 (t, J = 7.4 Hz, 3H); ^{13}C NMR (125 MHz, $CDCl_3$) δ 159.3, 149.3, 135.8, 122.1, 119.2, 24.4, 14.5.

2-(Ethylthio)-3-methylpyridine (**4h**)

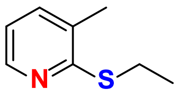

Yellow liquid (71 mg, 93% yield); R_f = 0.4 (Hexane/EtOAc = 5:1); ^{1}H NMR (500 MHz, $CDCl_3$) δ 8.29 (dd, J = 4.9, 1.7 Hz, 1H), 7.30 (ddd, J = 7.4, 1.8, 0.9 Hz, 1H), 6.90 (dd, J = 7.4, 4.9 Hz, 1H), 3.22 (q, J = 7.4 Hz, 2H), 2.24 (s, 3H), 1.38 (t, J = 7.4 Hz, 3H); ^{13}C NMR (125 MHz, $CDCl_3$) δ 158.2, 146.4, 136.2, 130.9, 118.8, 24.0, 18.6, 14.6. HRMS (ESI-TOF) (m/z): $[M+H]^+$ calcd for $C_8H_{12}NS^+$, 154.0685; found, 154.0685.

2-(Ethylthio)-4-methylpyridine (**4i**)

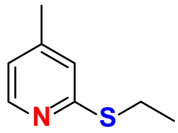

Yellow liquid (69 mg, 90% yield); R_f = 0.4 (Hexane/EtOAc = 5:1); ^{1}H NMR (500 MHz, $CDCl_3$) δ 8.28 (d, J = 5.1 Hz, 1H), 6.99 (s, 1H), 6.79 (dd, J = 5.2, 1.5 Hz, 1H), 3.15 (q, J = 7.4 Hz, 2H), 2.26 (s, 3H), 1.36 (t, J = 7.4 Hz, 3H); ^{13}C NMR (125 MHz, $CDCl_3$) δ 158.9, 148.9, 147.2, 122.7, 120.7, 24.4, 20.8, 14.6. HRMS (ESI-TOF) (m/z): $[M+H]^+$ calcd for $C_8H_{12}NS^+$, 154.0685; found, 154.0685.

6-(Ethylthio)pyridin-3-amine (**4j**)[8]

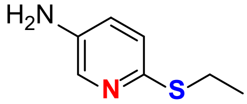

Brown liquid (72 mg, 94% yield); R_f = 0.4 (Hexane/EtOAc = 3:1); ^{1}H NMR (500 MHz, $CDCl_3$) δ 8.02 (d, J = 2.9 Hz, 1H), 7.04 (dd, J = 8.4, 0.7 Hz, 1H), 6.91 (dd, J = 8.4, 2.9 Hz, 1H), 3.51 (s, 2H), 3.07 (q, J = 7.3 Hz, 2H), 1.31 (t, J = 7.4 Hz, 3H); ^{13}C NMR (125 MHz, $CDCl_3$) δ 146.5, 139.9, 136.9, 123.9, 123.4, 25.9, 14.7.

2-(Ethylthio)pyridin-3-amine (**4k**)[9]

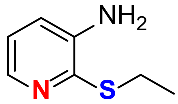

Brown liquid (67 mg, 87% yield); R_f = 0.4 (Hexane/EtOAc = 3:1); ^{1}H NMR (500 MHz, $CDCl_3$) δ 7.97 (dd, J = 4.2, 2.0 Hz, 1H), 6.93 – 6.83 (m, 2H), 3.23 (q, J = 7.4 Hz, 2H), 1.35 (t, J = 7.4 Hz, 3H); ^{13}C NMR (125 MHz, $CDCl_3$) δ 143.0, 140.9, 139.4, 120.6, 120.4, 25.2, 14.9.

6-(Ethylthio)pyridin-3-ol **(4l)**

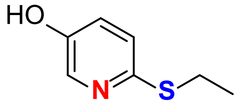

Pale-yellow solid (73 mg, 94% yield); R_f = 0.4 (Hexane/EtOAc = 5:1); ^{1}H NMR (500 MHz, $CDCl_3$) δ 8.12 (dd, J = 2.8, 0.8 Hz, 1H), 7.22 (dd, J = 8.6, 2.8 Hz, 1H), 7.18 (dd, J = 8.7, 0.7 Hz, 1H), 3.02 (q, J = 7.3 Hz, 2H), 1.29 (t, J = 7.3 Hz, 3H); ^{13}C NMR (125 MHz, $CDCl_3$) δ 152.0, 148.1, 137.0, 125.8, 125.1, 26.9, 14.5. HRMS (ESI-TOF) (m/z): $[M+H]^+$ calcd for $C_7H_{10}NOS^+$, 156.0478; found, 156.0476.

6-(Ethylthio)-4-methylpyridin-3-amine (**4m**)

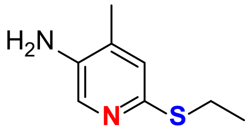

Red liquid (80 mg, 95% yield); R_f = 0.5 (Hexane/EtOAc = 2:1); ^{1}H NMR (500 MHz, $CDCl_3$) δ 7.93 (s, 1H), 6.95 (t, J = 0.7 Hz, 1H), 3.07 (q, J = 7.4 Hz, 2H), 2.13 (d, J = 0.8 Hz, 3H), 1.31 (t, J = 7.3 Hz, 3H); ^{13}C NMR (125 MHz, $CDCl_3$) δ 146.7, 138.8, 136.5, 132.2, 124.9, 25.8,

16.8, 14.8. HRMS (ESI-TOF) (m/z): $[M+H]^+$ calcd for $C_8H_{13}N_2S^+$, 169.0794; found, 169.0792.

2-(Ethylthio)-3-phenylpyridine (**4n**)

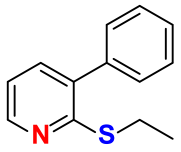

Yellow liquid (91 mg, 85% yield); R_f= 0.5 (Hexane/EtOAc = 10:1); ^1H NMR (500 MHz, $CDCl_3$) δ 8.46 (dd, J = 4.9, 1.7 Hz, 1H), 7.50 – 7.42 (m, 5H), 7.41 (dd, J = 7.4, 1.8 Hz, 1H), 7.06 (dd, J = 7.5, 4.9 Hz, 1H), 3.19 (q, J = 7.3 Hz, 2H), 1.35 (t, J = 7.4 Hz, 3H).; ^{13}C NMR (125 MHz, $CDCl_3$) δ 157.5, 147.9, 138.2, 136.3, 136.0, 129.1, 128.3, 128.0, 118.8, 24.6, 14.3. HRMS (ESI-TOF) (m/z): $[M+H]^+$ calcd for $C_{13}H_{14}NS^+$, 216.0841; found, 216.0839.

6-(Ethylthio)-N,N-dimethylpicolinamide (**4o**)

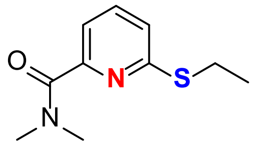

Yellow liquid (92 mg, 88% yield); R_f= 0.4 (Hexane/EtOAc = 2:1); ^1H NMR (500 MHz, $CDCl_3$) δ 7.54 (t, J = 7.8 Hz, 1H), 7.29 (d, J = 7.5 Hz, 1H), 7.16 (d, J = 8.1 Hz, 1H), 3.15 (q, J = 7.4 Hz, 2H), 3.10 (d, J = 17.2 Hz, 6H), 1.34 (t, J = 7.4 Hz, 3H); ^{13}C NMR (125 MHz, $CDCl_3$) δ 168.5, 158.1, 154.0, 136.6, 122.7, 119.1, 39.0, 35.8, 24.2, 14.6. HRMS (ESI-TOF) (m/z): $[M+Na]^+$ calcd for $C_{10}H_{14}N_2NaOS^+$, 233.0719; found, 233.0715.

2-(Ethylthio)-3-iodopyridine (**4p**)

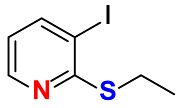

Brown liquid (119 mg, 90% yield); R_f= 0.4 (Hexane/EtOAc = 5:1); ^1H NMR (500 MHz, $CDCl_3$) δ 8.39 (dd, J = 4.7, 1.6 Hz, 1H), 7.90 (dd, J = 7.7, 1.6 Hz, 1H), 6.70 (dd, J = 7.7, 4.7 Hz, 1H), 3.13 (q, J = 7.4 Hz, 2H), 1.37 (t, J = 7.4 Hz, 3H); ^{13}C NMR (125 MHz, $CDCl_3$) δ 161.8, 148.1, 145.7, 119.9, 93.7, 26.9, 14.0. HRMS (ESI-TOF) (m/z): $[M+H]^+$ calcd for $C_7H_9INS^+$, 265.9500; found, 265.9504.

6-(Ethylthio)picolinonitrile (**4q**)

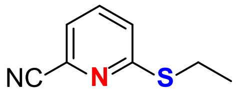

Brown solid (71 mg, 87% yield); MP: 50-52 °C, R_f = 0.4 (Hexane/EtOAc = 3:1); ^1H NMR (500 MHz, $CDCl_3$) δ 8.64 (d, J = 2.5 Hz), 7.63 (dd, J = 8.4, 2.2 Hz), 7.21 (dd, J = 8.4, 0.9 Hz), 3.19 (q, J = 7.4 Hz, 2H), 1.37 (t, J = 7.4 Hz, 3H); ^{13}C NMR (125 MHz, $CDCl_3$) δ 165.6, 152.1, 137.6, 121.7, 117.1, 104.3, 24.5, 14.2. HRMS (ESI-TOF) (m/z): $[M+H]^+$ calcd for $C_8H_9N_2S^+$, 165.0481; found, 165.0481.

3,5-Bis(ethylthio)pyridine (**4r**)

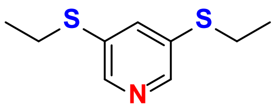

Brown liquid (40 mg, 40% yield); R_f = 0.4 (Hexane/EtOAc = 5:1); ^{1}H NMR (500 MHz, $CDCl_3$) δ 8.32 (s, 2H), 7.55 (s, 1H), 2.95 (q, J = 7.4 Hz, 4H), 1.31 (t, J = 7.4 Hz, 6H); ^{13}C NMR (125 MHz, $CDCl_3$) δ 146.5 (2C), 136.6 (2C), 134.0, 27.6 (2C), 14.2 (2C). HRMS (ESI-TOF) (m/z): $[M+H]^+$ calcd for $C_9H_{14}NS_2^+$, 200.0562; found, 200.0559.

2-(Ethylthio)-5-iodopyrimidine (**4s**)[5]

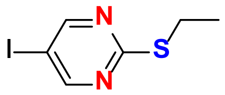

Brown solid (122 mg, 92% yield); MP: 64-65 °C, R_f = 0.5 (Hexane/EtOAc = 3:1); ^{1}H NMR (500 MHz, $CDCl_3$) δ 8.64 (s, 2H), 3.09 (q, J = 7.4 Hz, 2H), 1.36 (t, J = 7.4 Hz, 3H); ^{13}C NMR (125 MHz, $CDCl_3$) δ 171.1, 162.2 (2C), 86.2, 25.4, 14.2.

4-(Ethylsulfonyl)aniline (**5a**)

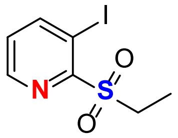

Yellow liquid (138 mg, 93% yield); R_f = 0.5 (Hexane/EtOAc = 3:1); ^{1}H NMR (500 MHz, $CDCl_3$) δ 8.58 (dd, J = 4.6, 1.5 Hz, 1H), 8.40 (dd, J = 8.0, 1.5 Hz, 1H), 7.20 (dd, J = 8.0, 4.5 Hz, 1H), 3.67 (q, J = 7.4 Hz, 2H), 1.46 (t, J = 7.4 Hz, 3H); ^{13}C NMR (125 MHz, $CDCl_3$) δ 157.3, 150.9, 147.2, 127.3, 86.1, 46.0, 7.2. HRMS (ESI-TOF) (m/z): $[M]^+$ calcd for $C_7H_8INO_2S^+$, 296.9553; found, 296.9551.

2-((1-Chloroethyl)thio)-3-iodopyridine (**5b**)

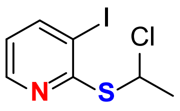

Yellow liquid (136 mg, 91% yield); R_f = 0.5 (Hexane/EtOAc = 5:1); ^{1}H NMR (500 MHz, $CDCl_3$) δ 8.50 (dt, J = 4.7, 1.2 Hz, 1H), 7.98 (dt, J = 7.7, 1.2 Hz, 1H), 6.82 (ddd, J = 7.8, 4.7, 0.8 Hz, 1H), 6.19 (q, J = 6.9 Hz, 1H), 2.01 (d, J = 6.9 Hz, 3H); ^{13}C NMR (125 MHz, $CDCl_3$) δ 158.8, 148.4, 146.2, 121.1, 93.2, 61.9, 25.7. HRMS (ESI-TOF) (m/z): $[M+Na]^+$ calcd for $C_7H_7ClINNaS^+$, 321.8925; found, 321.8917.

Ethyl(imino)(3-iodopyridin-2-yl)-$^{\lambda}$6-sulfanone (**5c**)

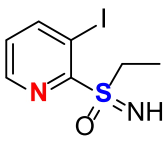

Yellow liquid (132 mg, 89% yield); R_f = 0.5 (Hexane/EtOAc = 3:1); ^{1}H NMR (500 MHz, $CDCl_3$) δ 8.51 (dd, J = 4.6, 1.5 Hz, 1H), 8.32 (dd, J = 7.9, 1.5 Hz, 1H), 7.11 (dd, J = 7.9, 4.6 Hz, 1H), 3.71 (ddt, J = 70.8, 14.2, 7.2 Hz, 2H), 1.43 (t, J = 7.4 Hz, 3H); ^{13}C NMR (125 MHz, $CDCl_3$) δ 159.3, 150.4,

147.1, 126.4, 85.1, 46.5, 7.7. HRMS (ESI-TOF) (m/z): $[M+H]^+$ calcd for $C_7H_{10}IN_2OS^+$, 296.9553; found, 296.9551.

2-(Ethylthio)-5-(4-methylthiophen-2-yl)pyridine (**5e**)

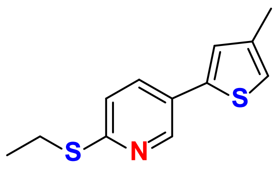

Yellow liquid (92 mg, 78% yield); R_f = 0.4 (Hexane/EtOAc = 5:1); ^{1}H NMR (500 MHz, $CDCl_3$) δ 8.88 (s, 1H), 8.58 (d, J = 4.5 Hz, 1H), 8.06 (dt, J = 7.9, 2.0 Hz, 1H), 7.39 (dd, J = 7.9, 4.9 Hz, 1H), 7.08 (d, J = 1.2 Hz, 1H), 2.52 (q, J = 7.4 Hz, 3H), 2.36 (d, J = 1.0 Hz, 2H), 1.01 (t, J = 7.4 Hz, 3H); ^{13}C NMR (125 MHz, $CDCl_3$) δ 149.3, 147.9, 142.2, 141.9, 137.4, 131.1, 128.9, 123.3, 120.7, 29.9, 16.0, 14.5. HRMS (ESI-TOF) (m/z): $[M+H]^+$ calcd for $C_{12}H_{14}NS_2^+$, 236.0557; found, 236.0562.

1,2-dibenzyldisulfane (**5f**)[10]

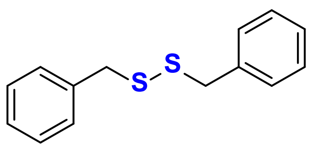

White solid (107 mg, 87% yield), MP: 71-72 °C; R_f = 0.5 (Hexane/EtOAc = 20:1); ^{1}H NMR (400 MHz, $CDCl_3$) δ 7.36 – 7.29 (m, 5H), 7.29 – 7.23 (m, 5H), 3.62 (s, 4H); ^{13}C NMR (100 MHz, $CDCl_3$) δ 137.4 (2C), 129.4 (4C), 128.5 (4C), 127.4 (2C), 43.4 (2C).

References:

[1] Itoh, T.; Mase, T. A General Palladium-Catalyzed Coupling of Aryl Bromides/Triflates and Thiols, *Org. Lett.*, **2004**, *6*, 4587–4590.

[2] Chen, B.; Wang, J.; Sun, W.; Pan, Y.; Ding, A.; Liu, W.; Guo, H. Thioxanthone-TfOH Complex (9-HTXTF) Photoredox Enabled Reduction of Sulfoxides, *Tetrahedron Letters*, **2024**, *139*, 155007.

[3] Liu, Y.; Kim, J.; Seo, H.; Park, S.; Chae, J. Copper(II)-Catalyzed Single-Step Synthesis of Aryl Thiols from Aryl Halides and 1,2-Ethanedithiol, *Adv. Synth. Catal.*, **2015**, *357*, 2205 - 2212.

[4] Ryota, I.; Miki, B.; Kei, M.; Junichiro, Y. Ni-Catalyzed Aryl Sulfide Synthesis through an Aryl Exchange Reaction, *J. Am. Chem. Soc.*, **2021**, *143*, 10333–10340.

[5] Zhang, H.; Cao, W.; Ma, D. L - Proline-Promoted CuI-Catalyzed C-S Bond Formation between Aryl Iodides and Thiols, *Synth. Commun.*, **2007**, *37*, 25-35.

[6] Anuradha, N.; Imran, K.; Somraj, G.; Govindasamy, S. Visible-Light-Driven Halogen-Bond-Assisted Direct Synthesis of Heteroaryl Thioethers Using Transition-Metal-Free One-Pot C-I Bond Formation/C-S Cross-Coupling Reaction, *J. Org. Chem.*, **2021**, *86*, 2570−2581.

[7] Benjamin, H.; Moritz, B.; Paul, K.; Thiolation of Pyridine-2-sulfonamides using Magnesium Thiolates, *Synthesis*, **2019**, *51*, 4452-4462.

[8] Forrest, H. S.; Walker, J. Chemotherapeutic Agents of the Sulphone type. Part V. 2: 5-Disubstituted Derivatives of Pyridine, *J. Chem. Soc.*, **1948**, 1939-1945.

[9] Beugelmansm, R.; Bois-Choussy, M.; Boudet, B. Etude des Reactions de srn1-partie 10: Action de Sulfanions sur les Halogenures d'aryle Fonctionnalises. Synthese Directe de Benzothiophenes et Thienopyridines, *Tetrahedron*, **1983**, *39*, 4153-4161.

[10] M. Obe; K. Tanaka; K. Nishiyama; W. Ando; Aerobic Oxidation of Thiols to Disulfides Catalyzed by Diaryl Tellurides under Photosensitized Conditions, *J. Org. Chem.*, **2011**, 76, 4173-4177.

E. ^{1}H NMR and ^{13}C NMR spectra for products

^{1}H NMR (400 MHz, $CDCl_3$) spectrum of compound 3a

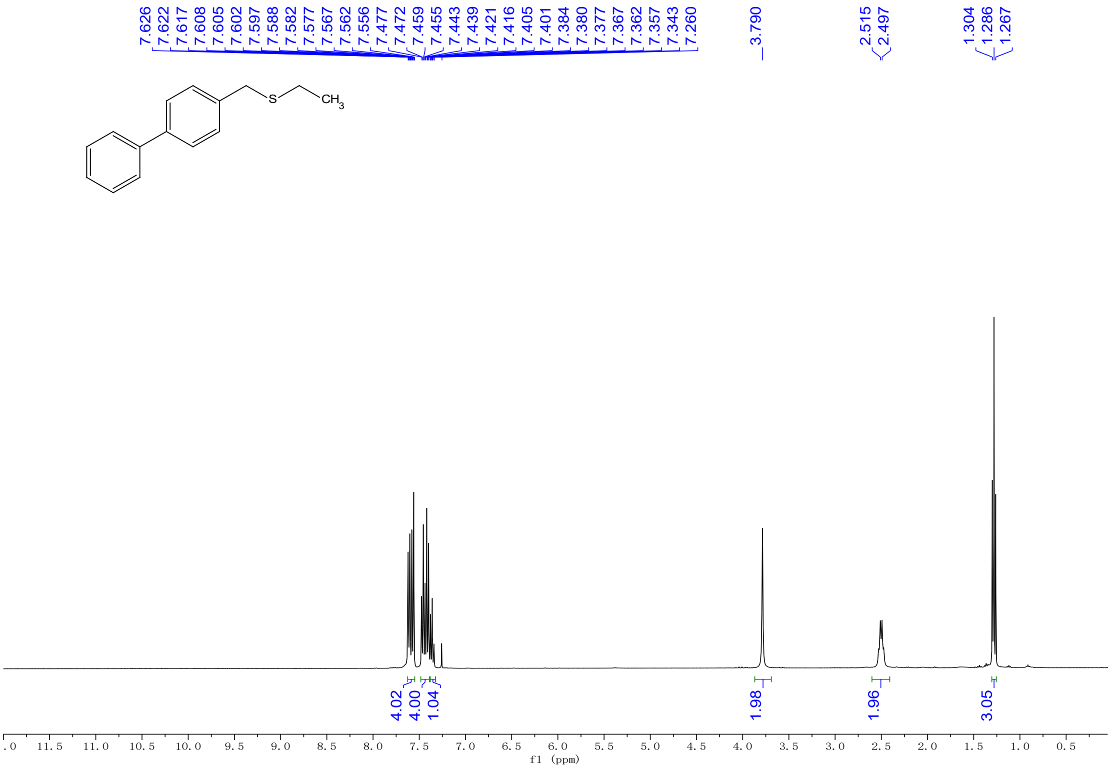

^{13}C NMR (100 MHz, $CDCl_3$) spectrum of compound 3a

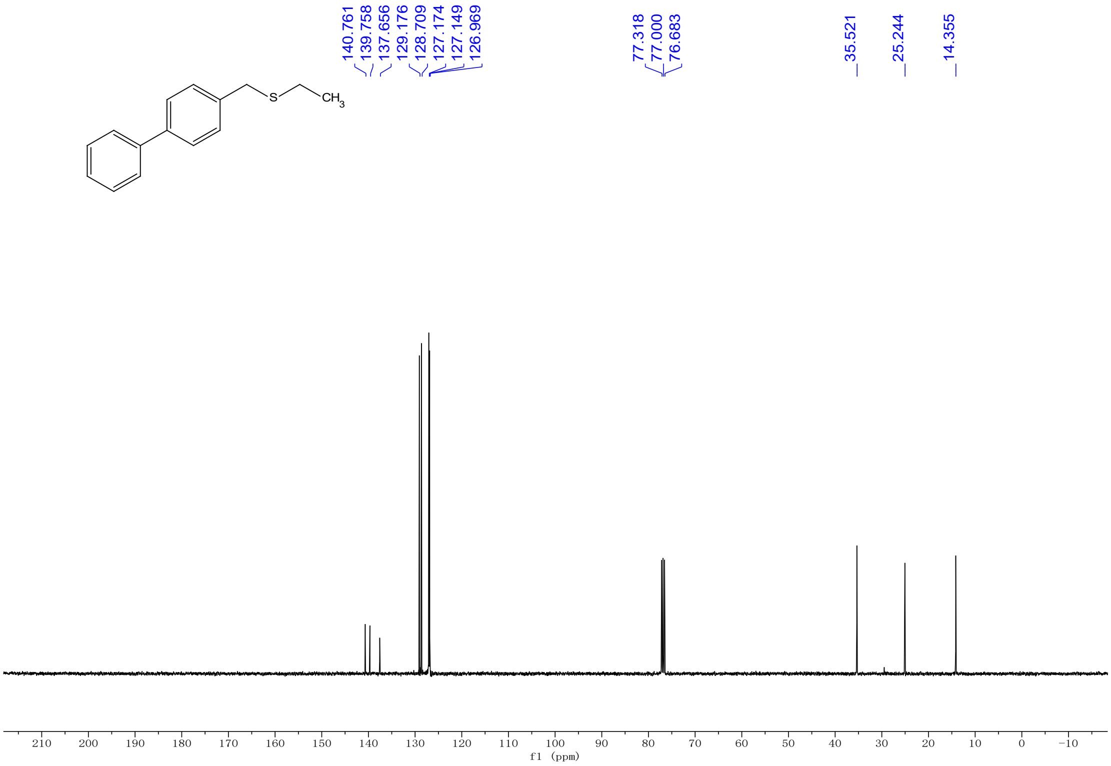

^{1}H NMR (400 MHz, $CDCl_3$) spectrum of compound 3b

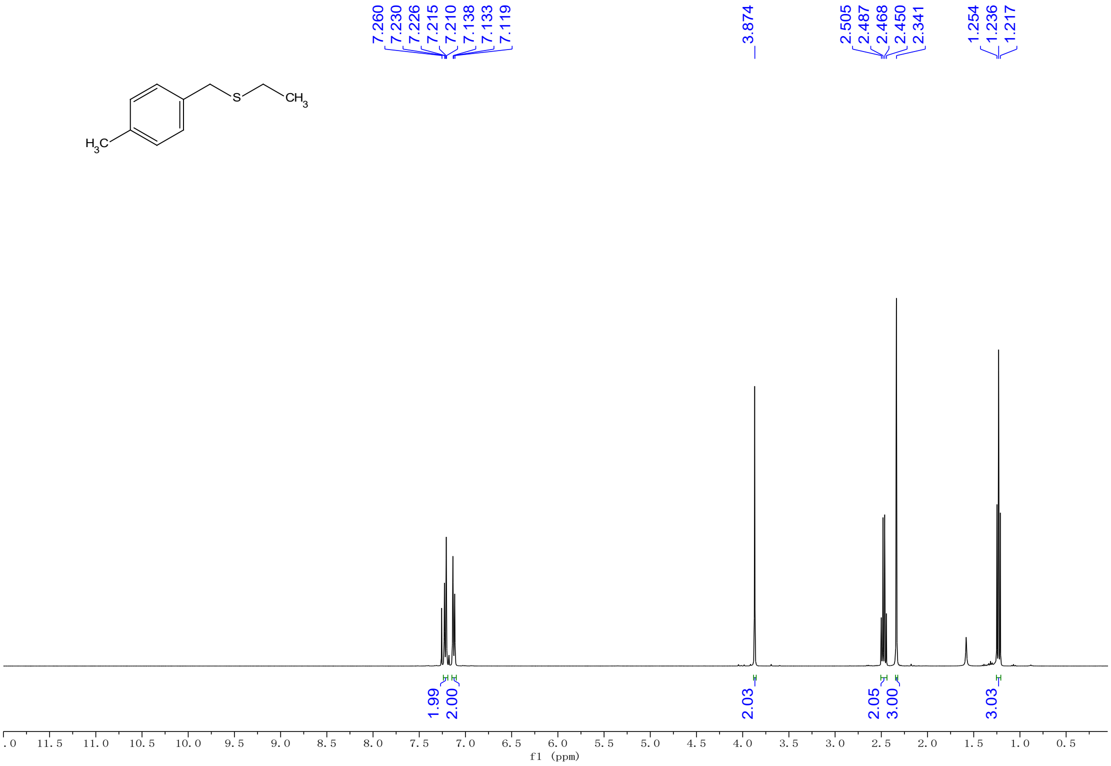

^{13}C NMR (100 MHz, $CDCl_3$) spectrum of compound 3b

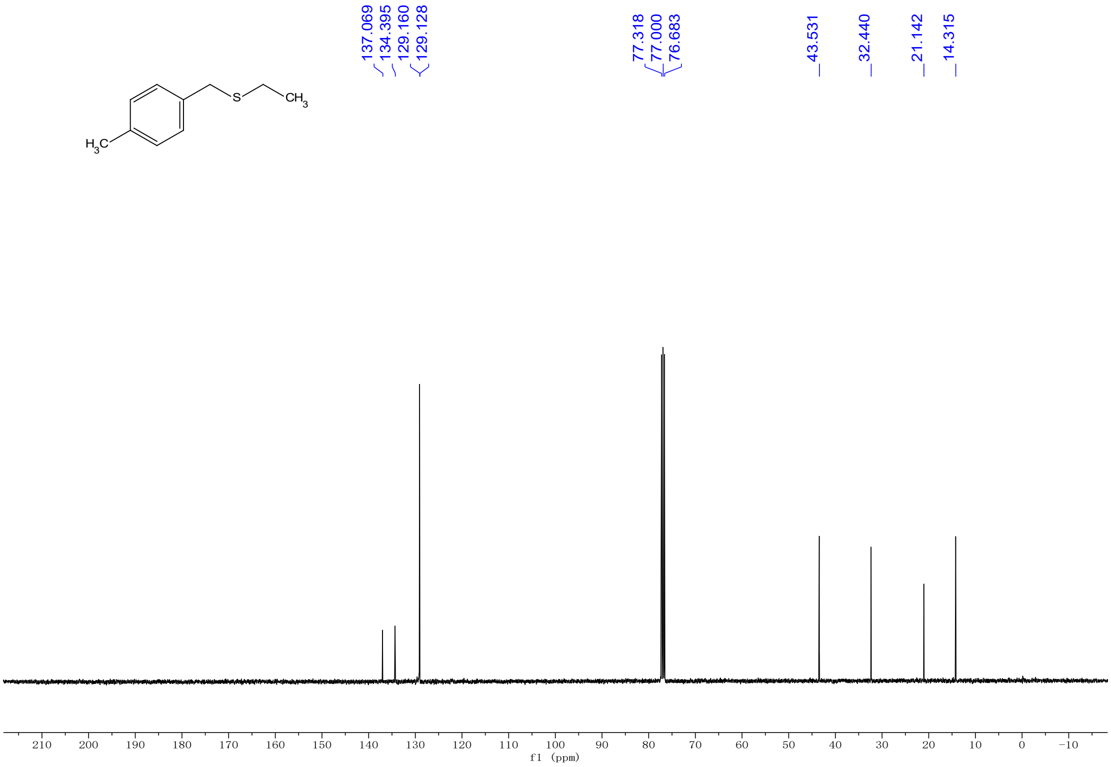

^{1}H NMR (400 MHz, $CDCl_3$) spectrum of compound 3c

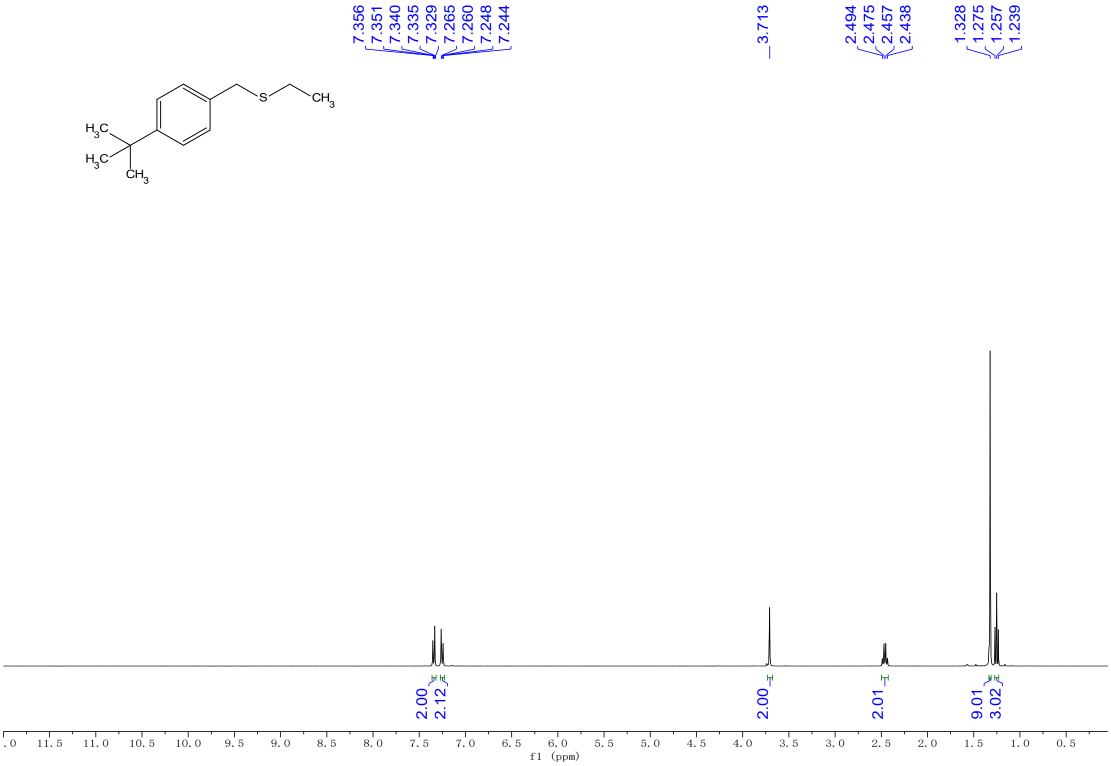

^{13}C NMR (100 MHz, $CDCl_3$) spectrum of compound 3c

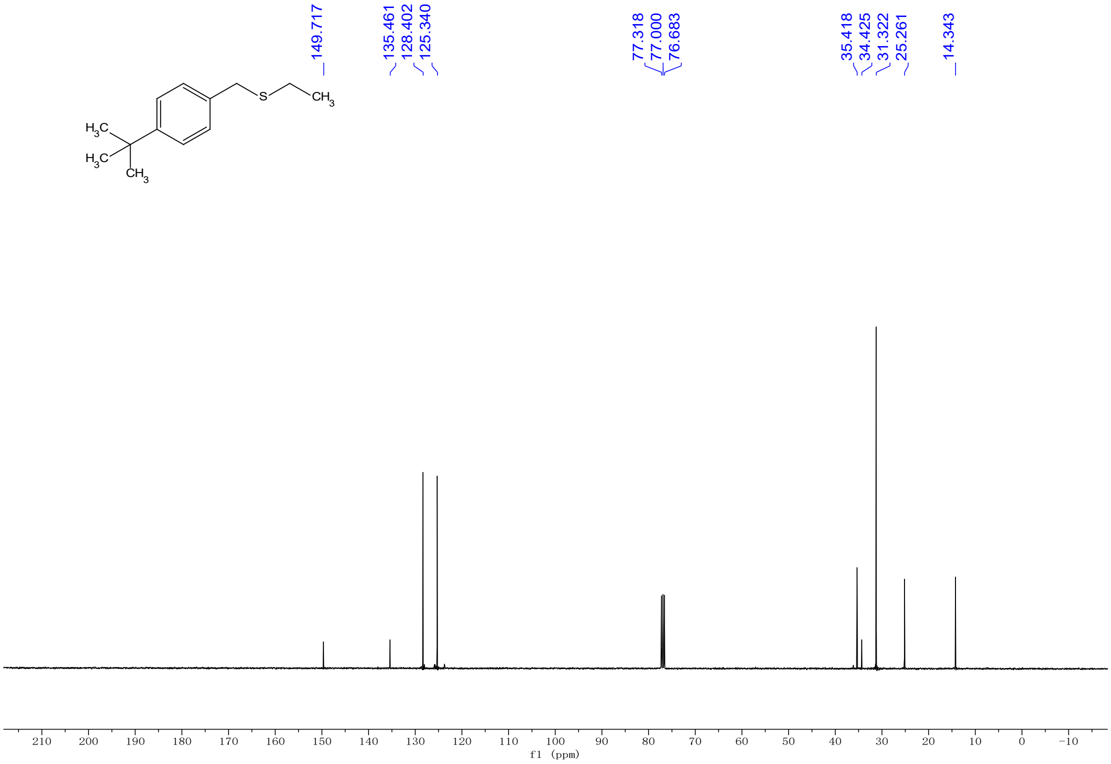

^{1}H NMR (400 MHz, $CDCl_3$) spectrum of compound 3d

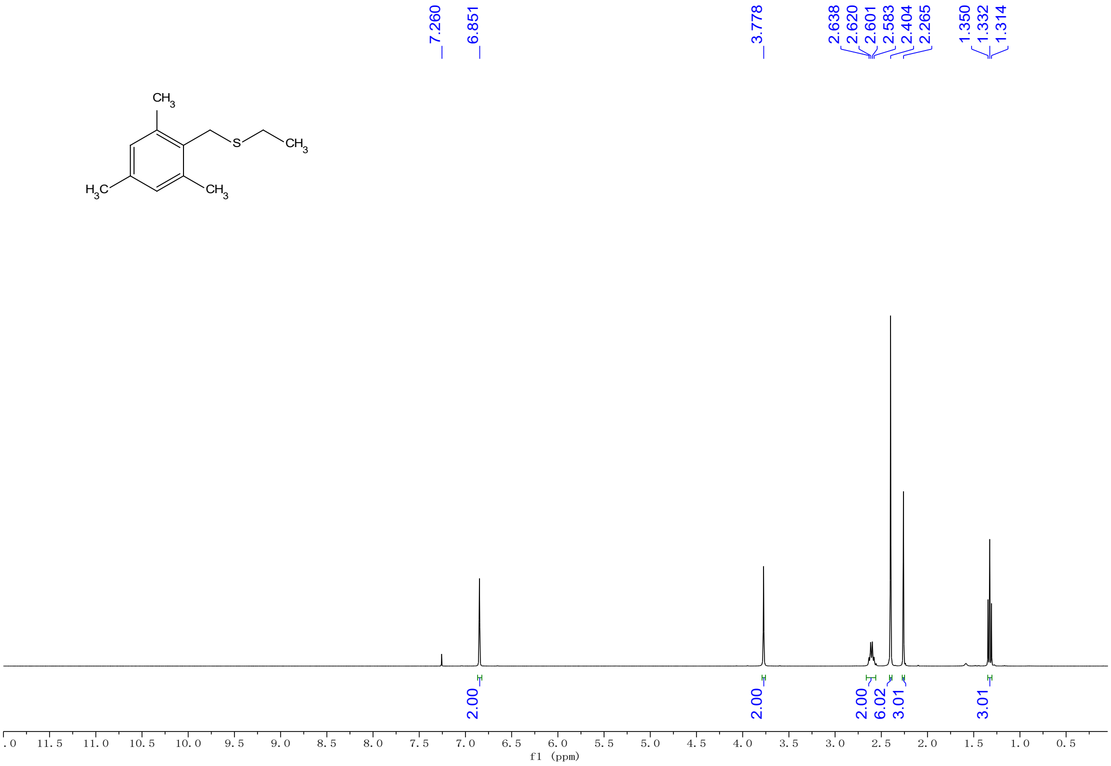

^{13}C NMR (100 MHz, $CDCl_3$) spectrum of compound 3d

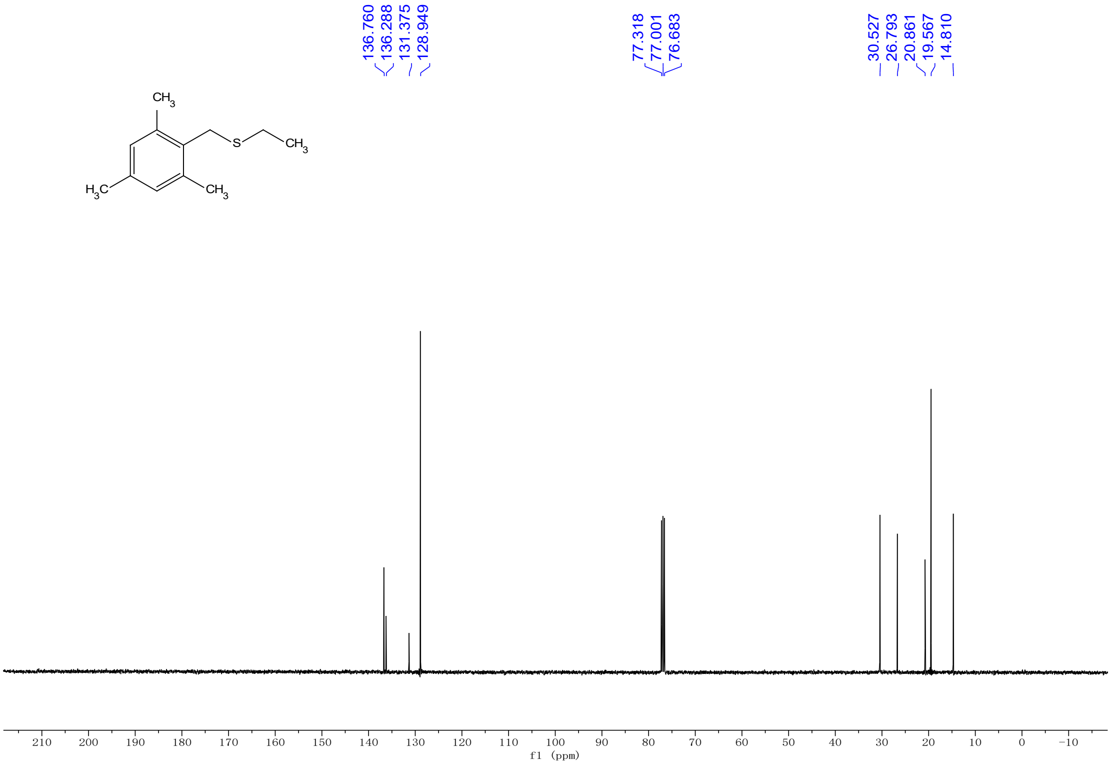

^{1}H NMR (400 MHz, $CDCl_3$) spectrum of compound 3e

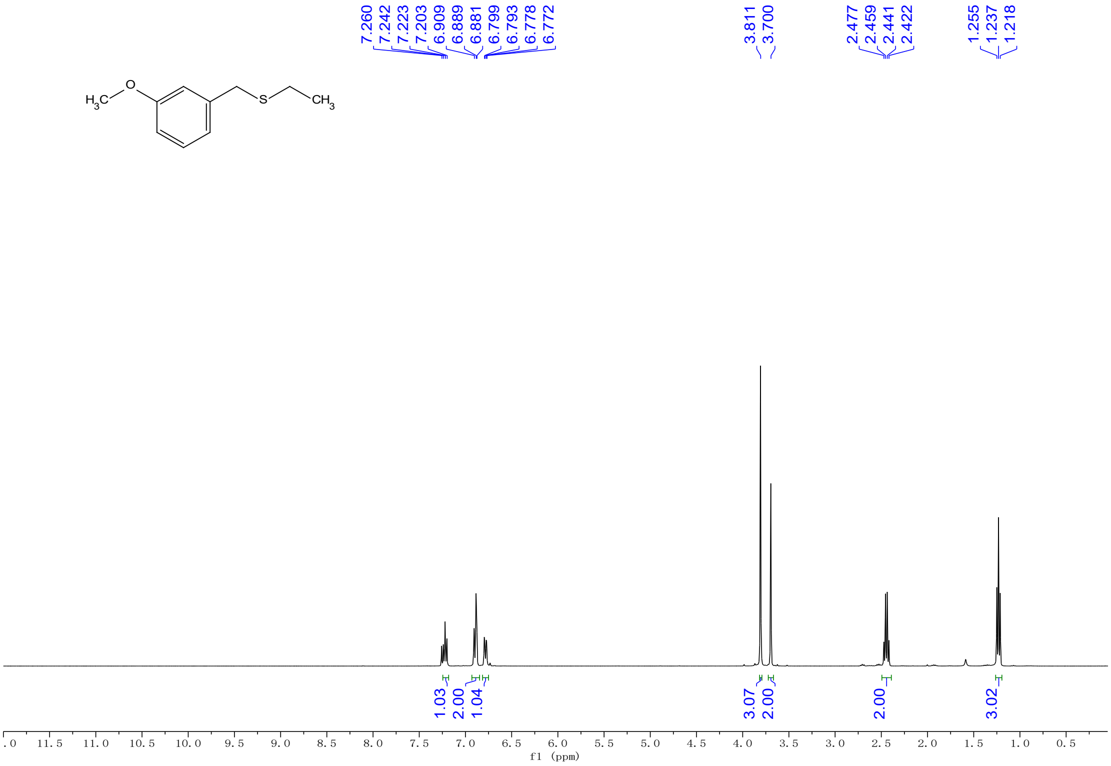

^{13}C NMR (100 MHz, $CDCl_3$) spectrum of compound 3e

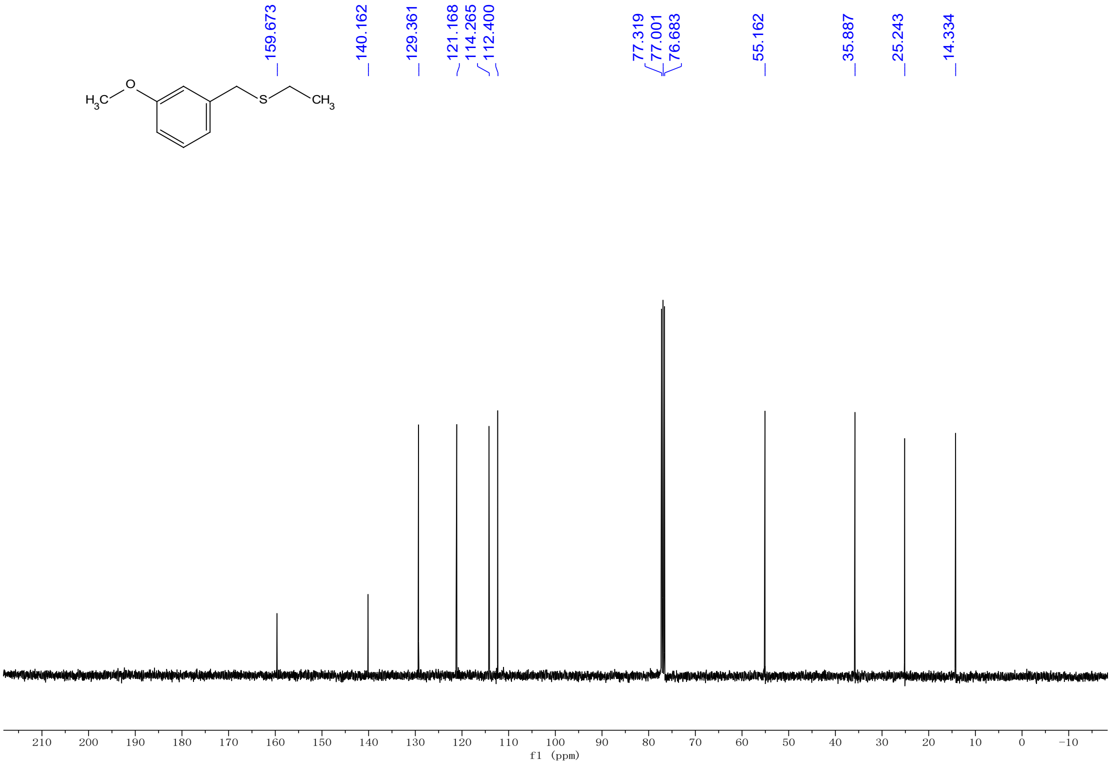

^{1}H NMR (400 MHz, CDCl$_3$) spectrum of compound 3f

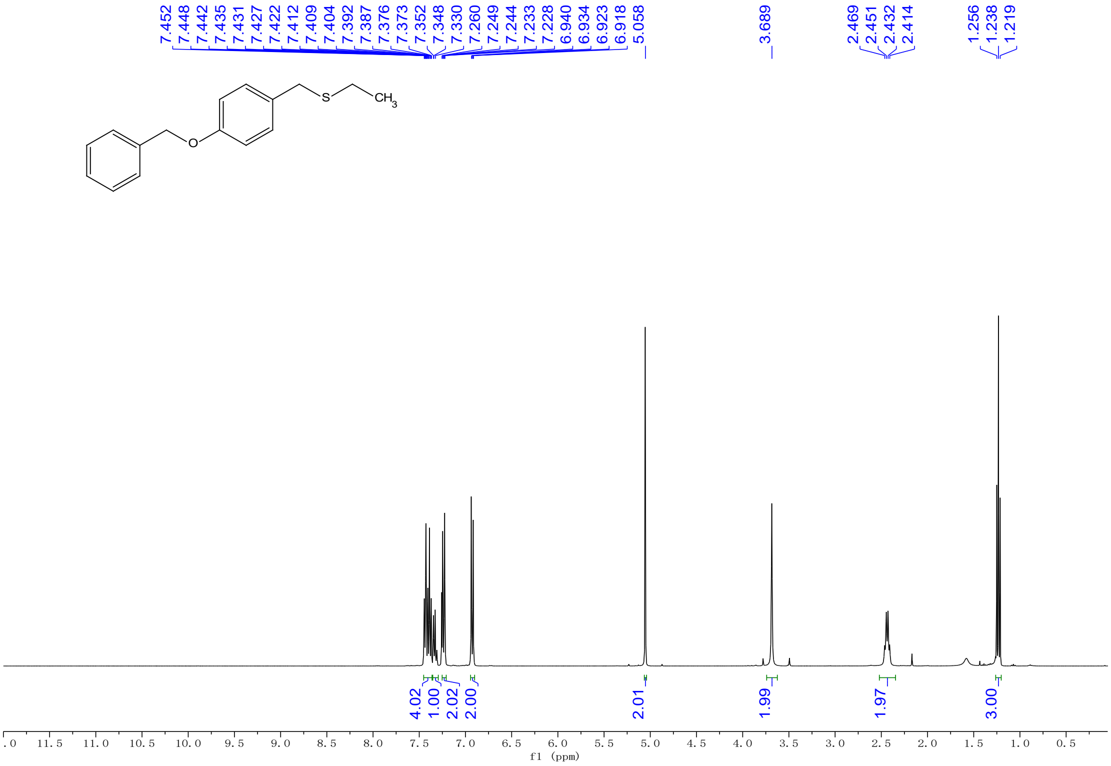

^{13}C NMR (100 MHz, CDCl$_3$) spectrum of compound 3f

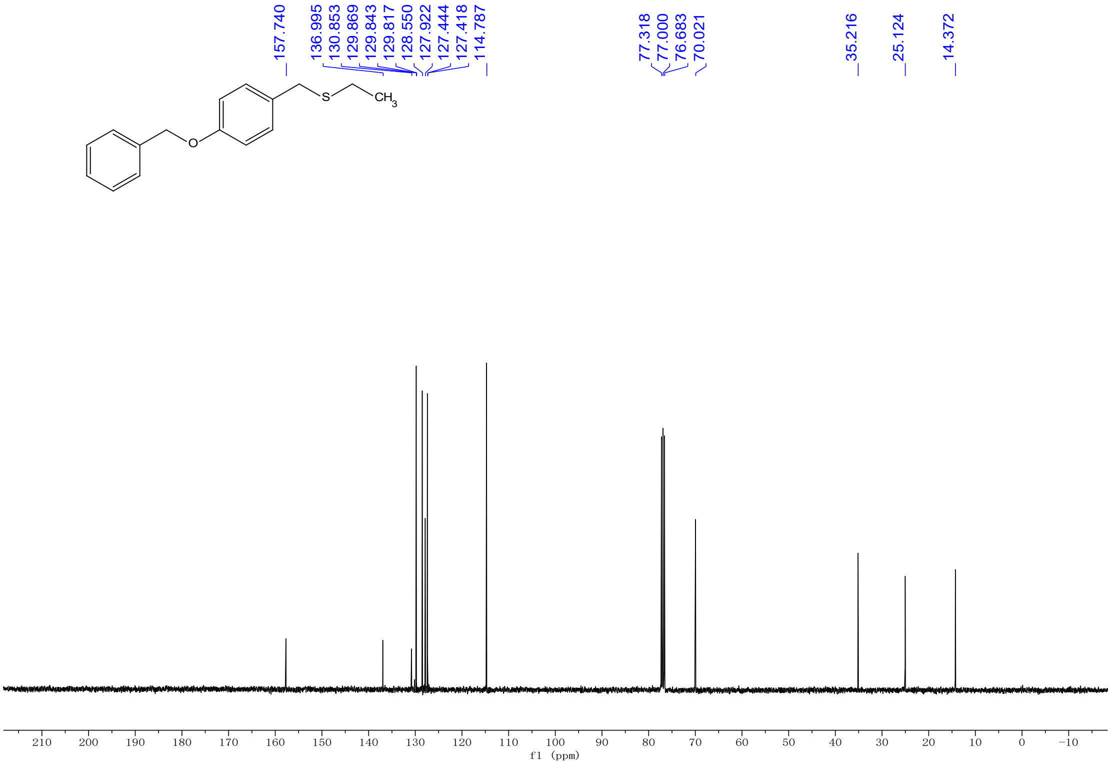

^{1}H NMR (400 MHz, $CDCl_3$) spectrum of compound 3g

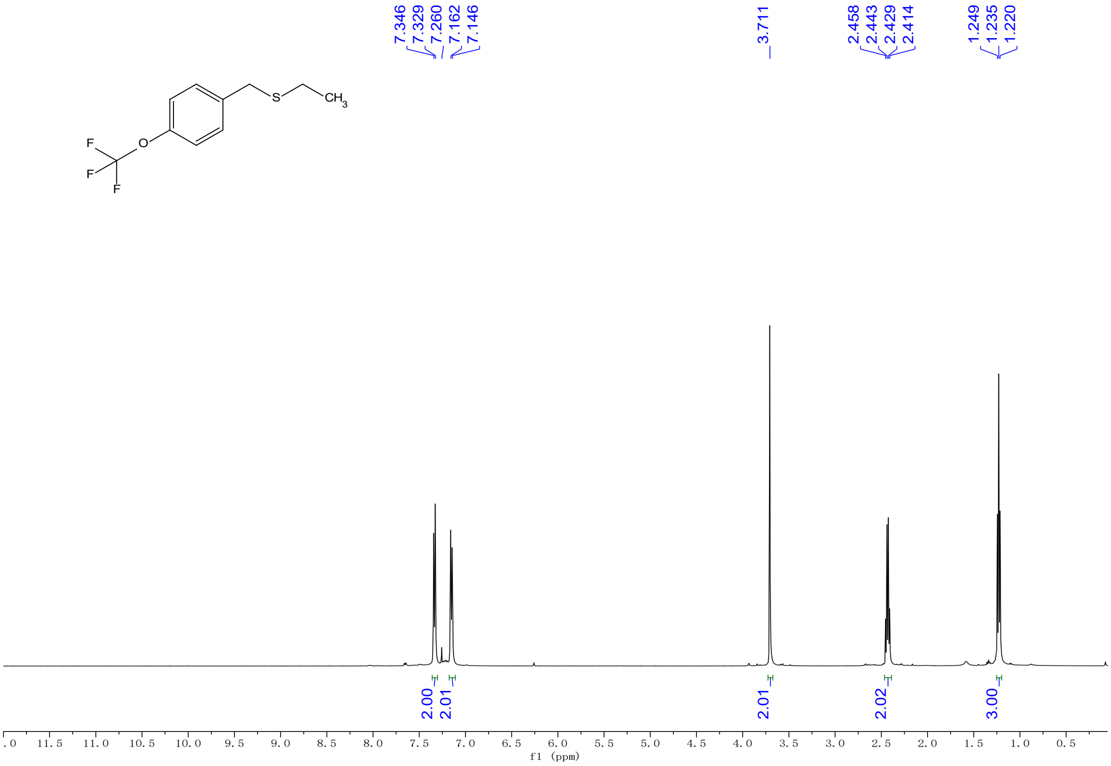

^{13}C NMR (100 MHz, $CDCl_3$) spectrum of compound 3g

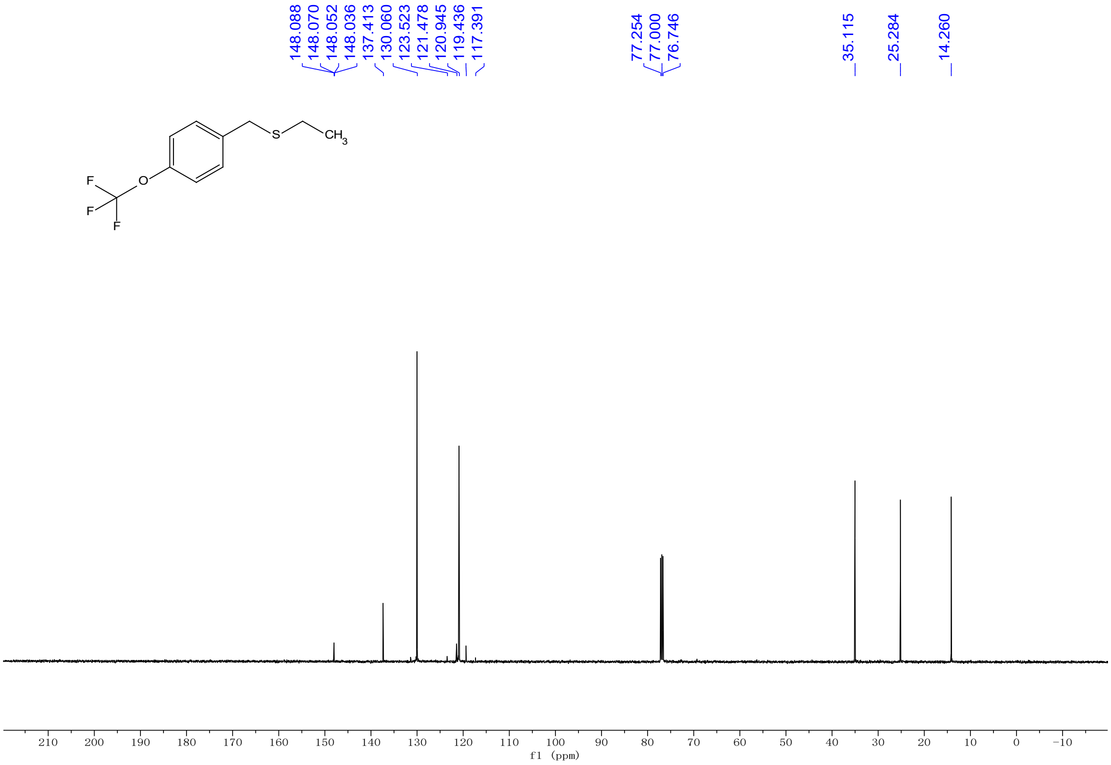

^{19}F NMR (376MHz, CDCl$_3$) spectrum of compound 3g

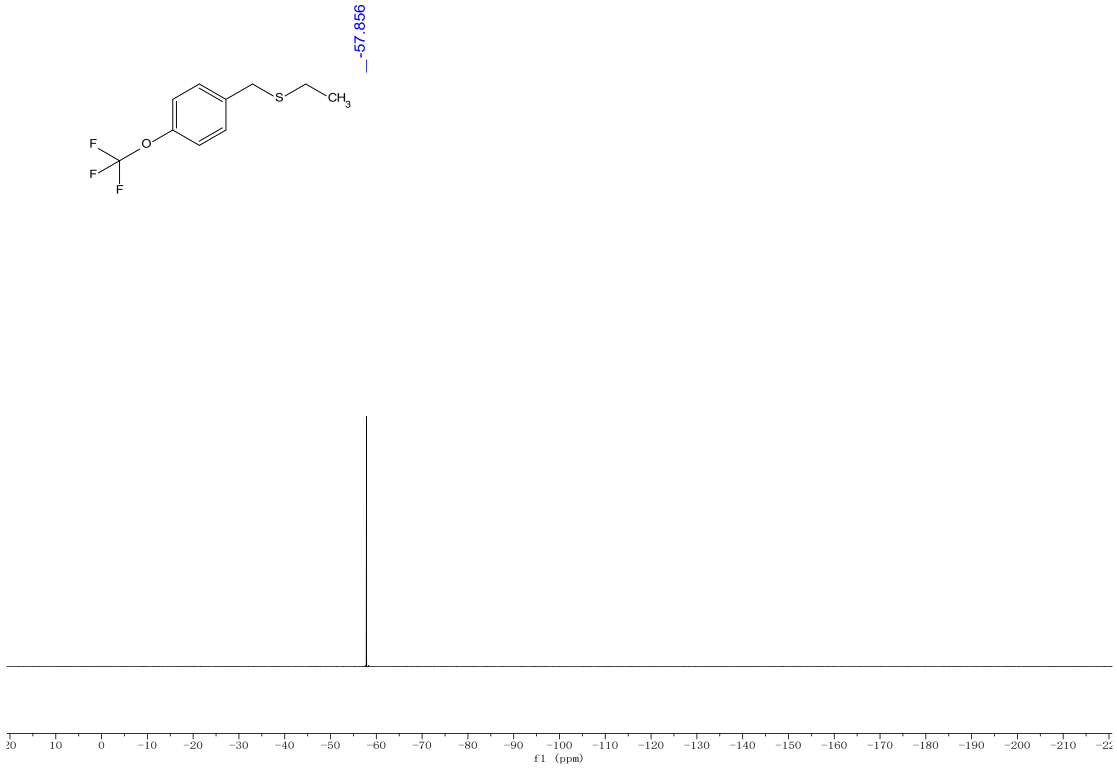

^{1}H NMR (400 MHz, CDCl$_3$) spectrum of compound 3h

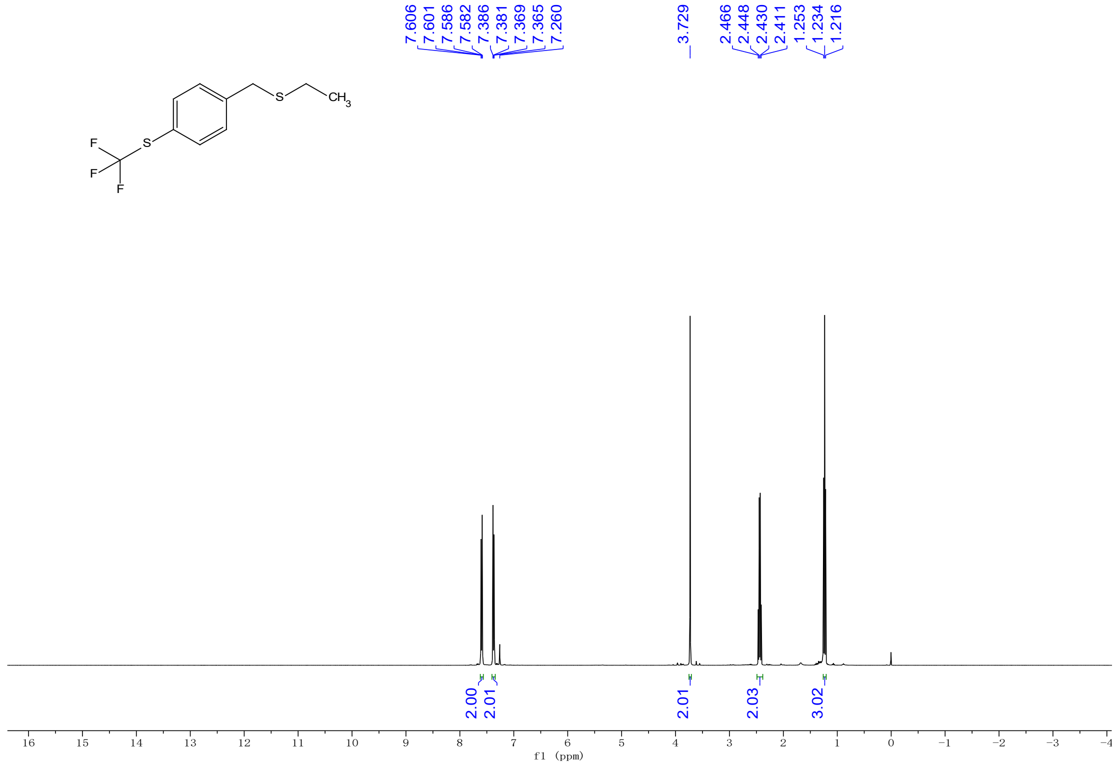

^{13}C NMR (100 MHz, $CDCl_3$) spectrum of compound 3h

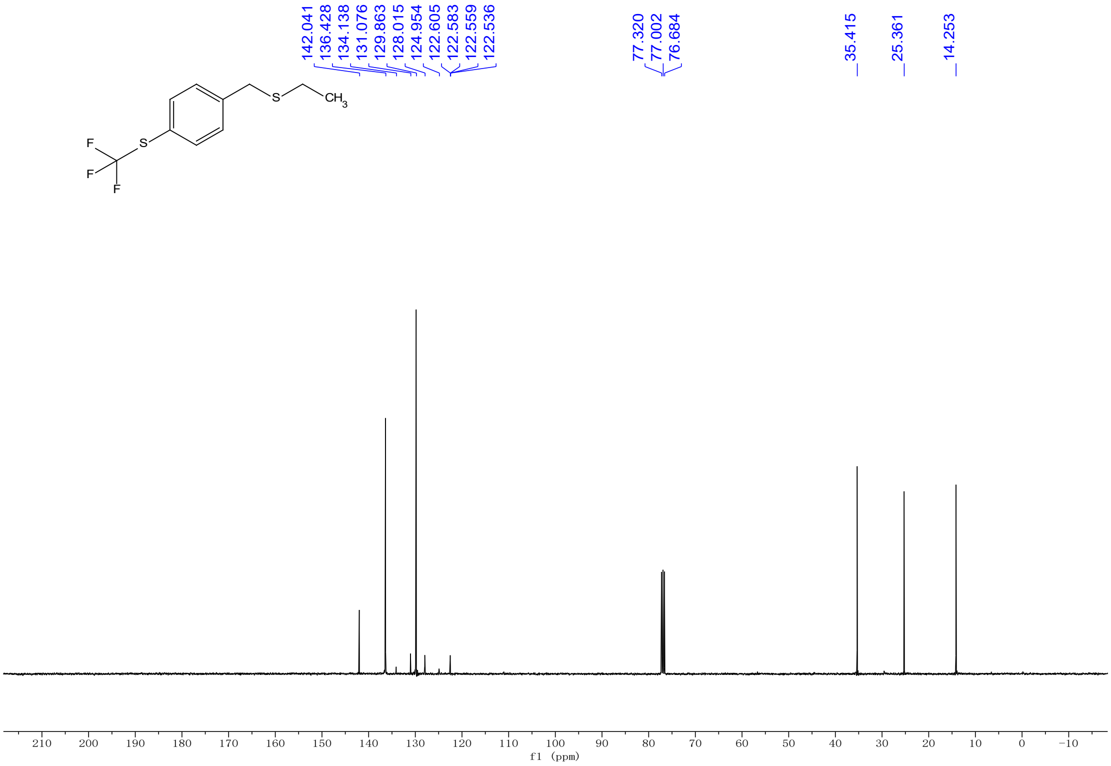

^{19}F NMR (376MHz, $CDCl_3$) spectrum of compound 3h

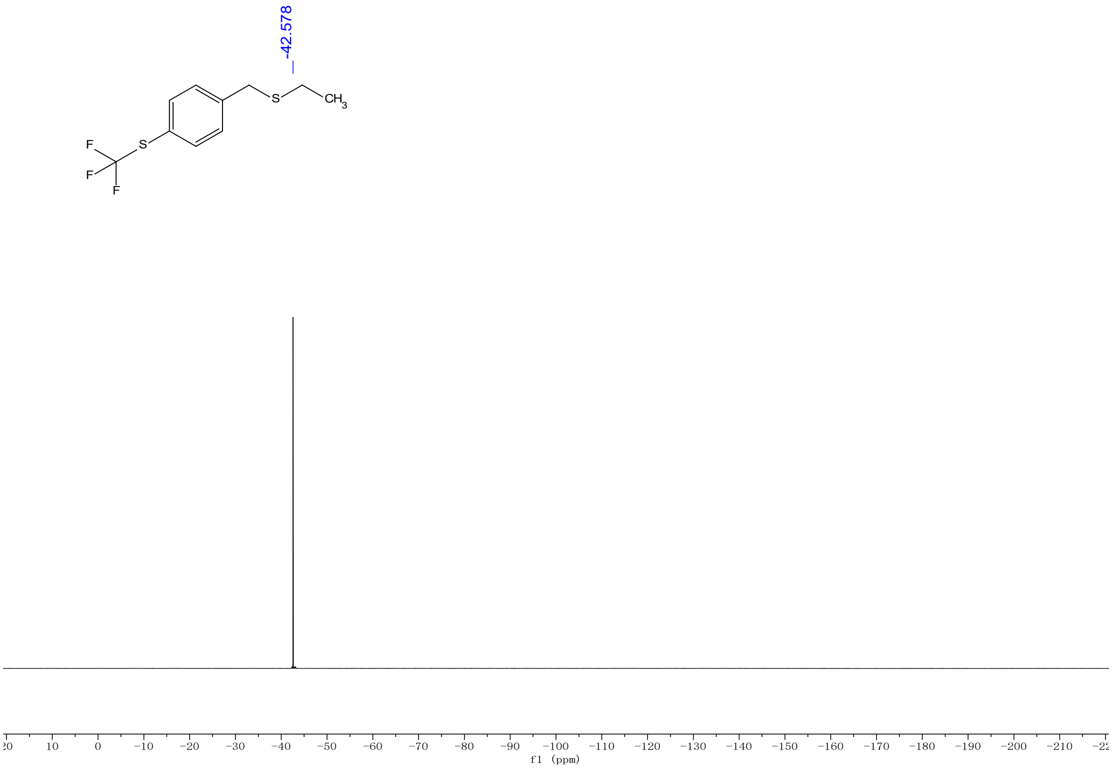

^{1}H NMR (400 MHz, $CDCl_3$) spectrum of compound 3i

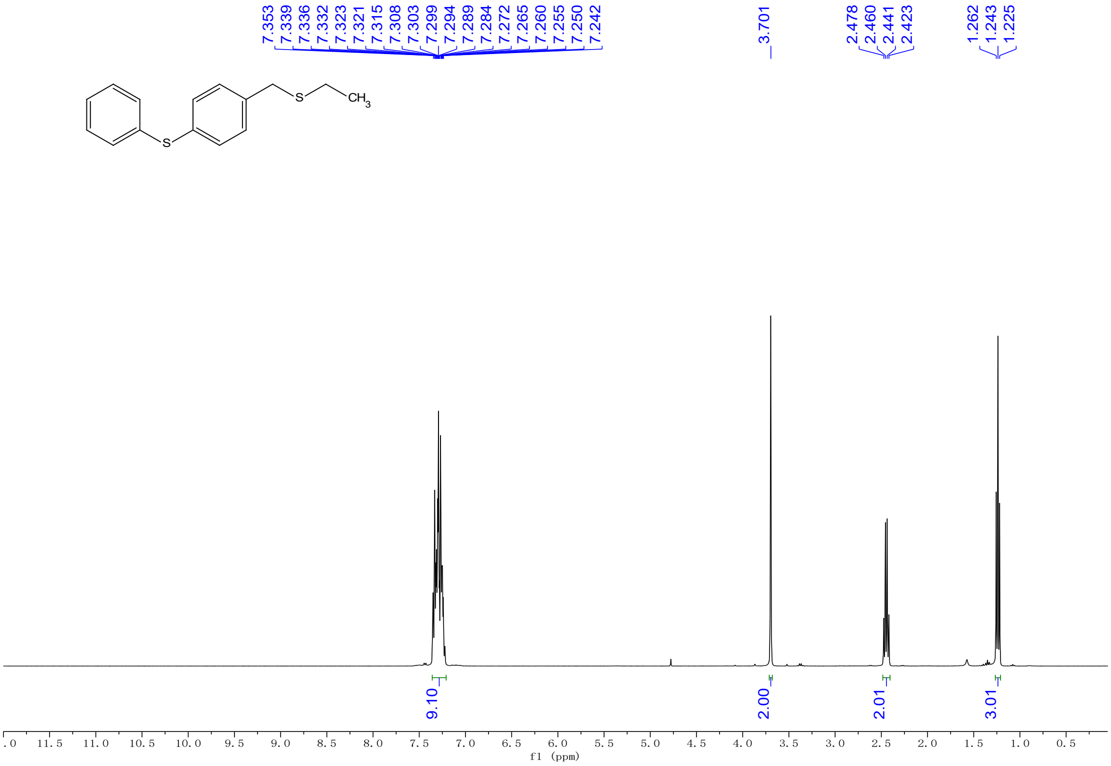

^{13}C NMR (100 MHz, $CDCl_3$) spectrum of compound 3i

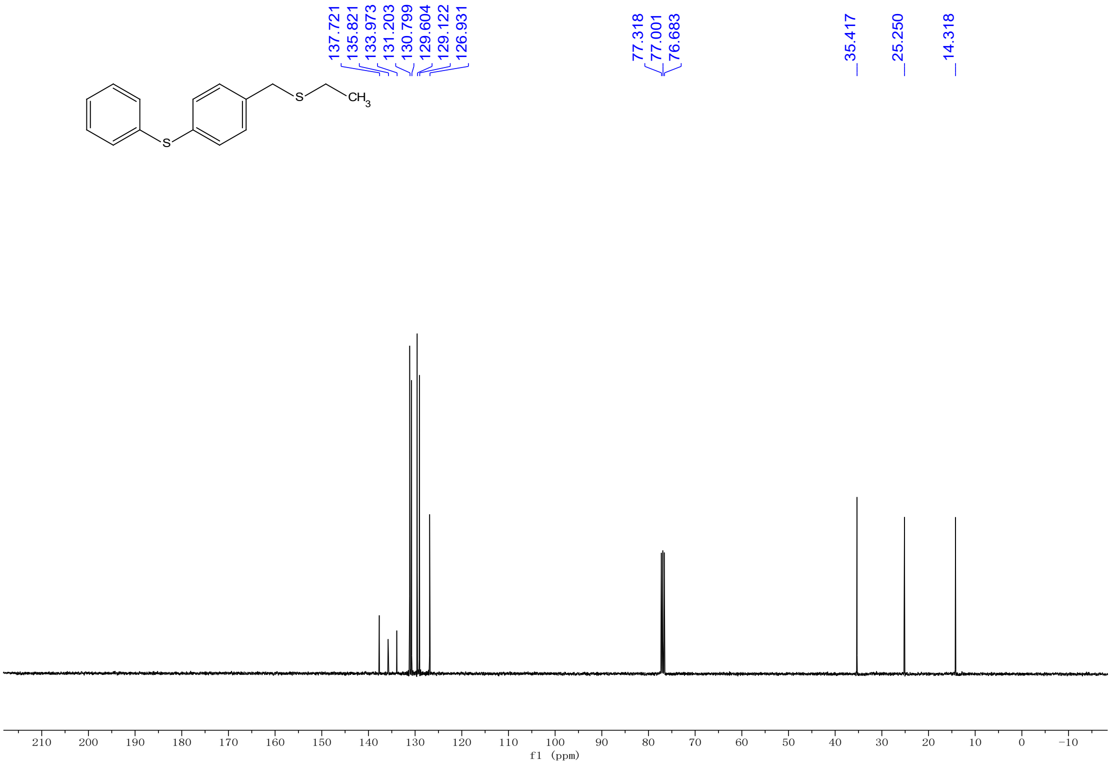

^{1}H NMR (400 MHz, $CDCl_3$) spectrum of compound 3j

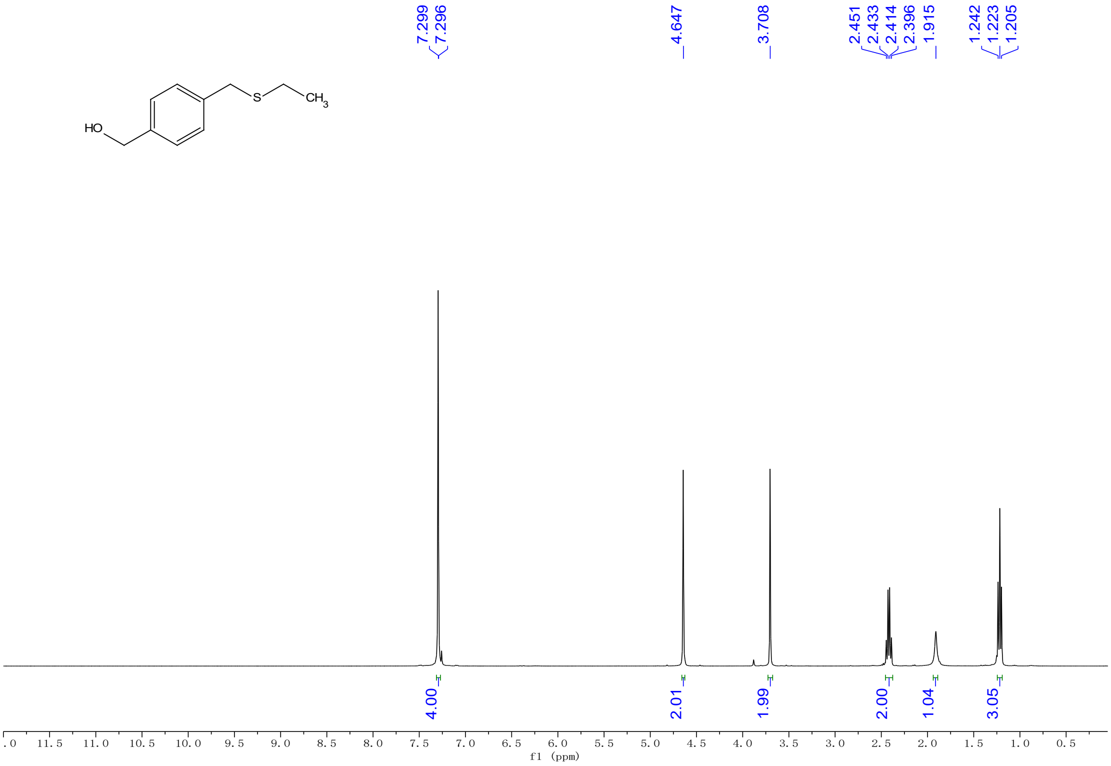

^{13}C NMR (100 MHz, $CDCl_3$) spectrum of compound 3j

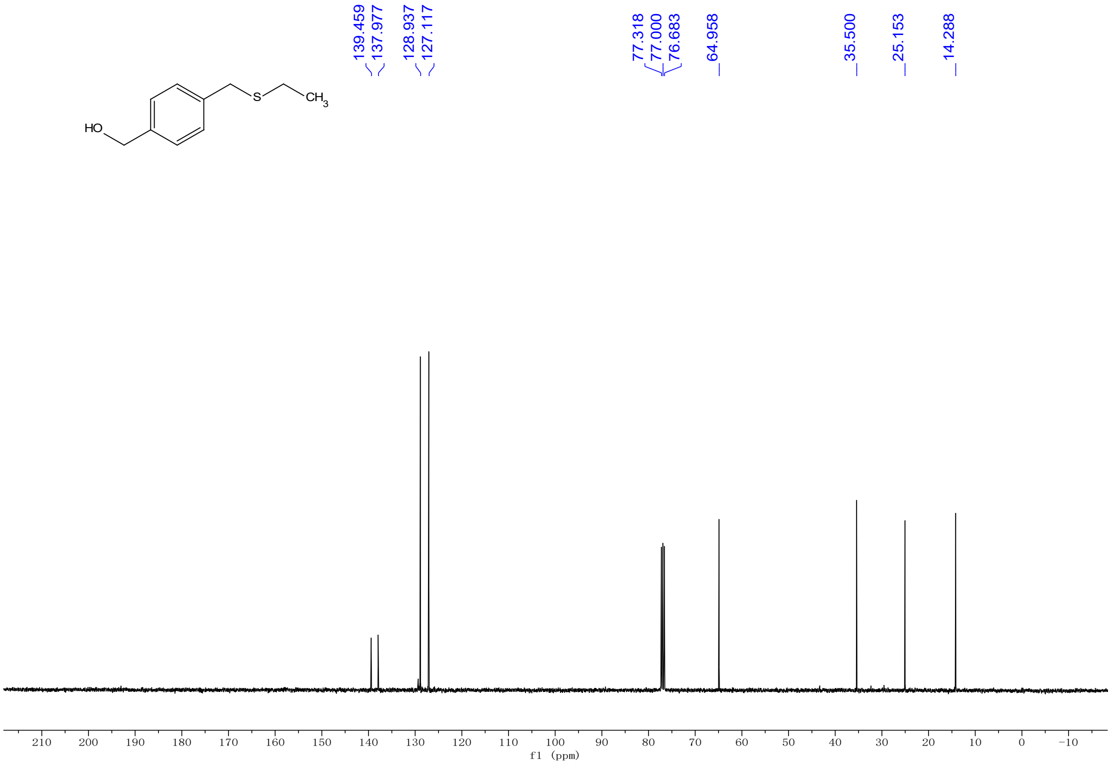

^{1}H NMR (400 MHz, $CDCl_3$) spectrum of compound 3k

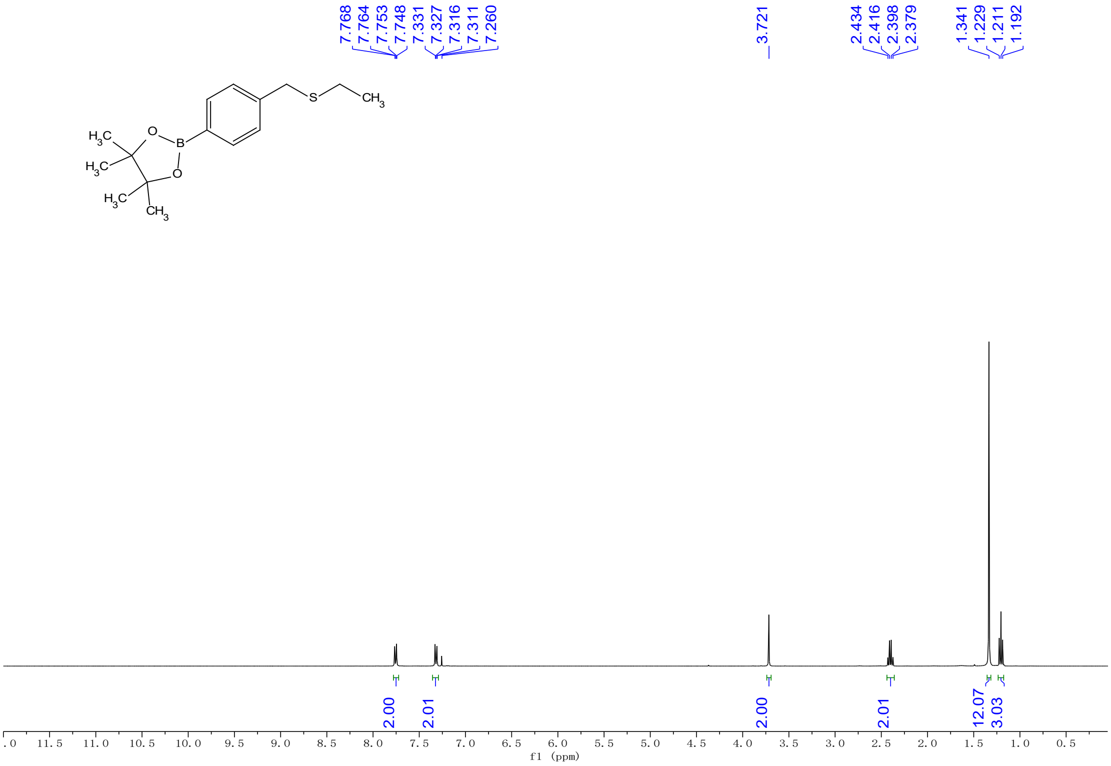

^{13}C NMR (100 MHz, $CDCl_3$) spectrum of compound 3k

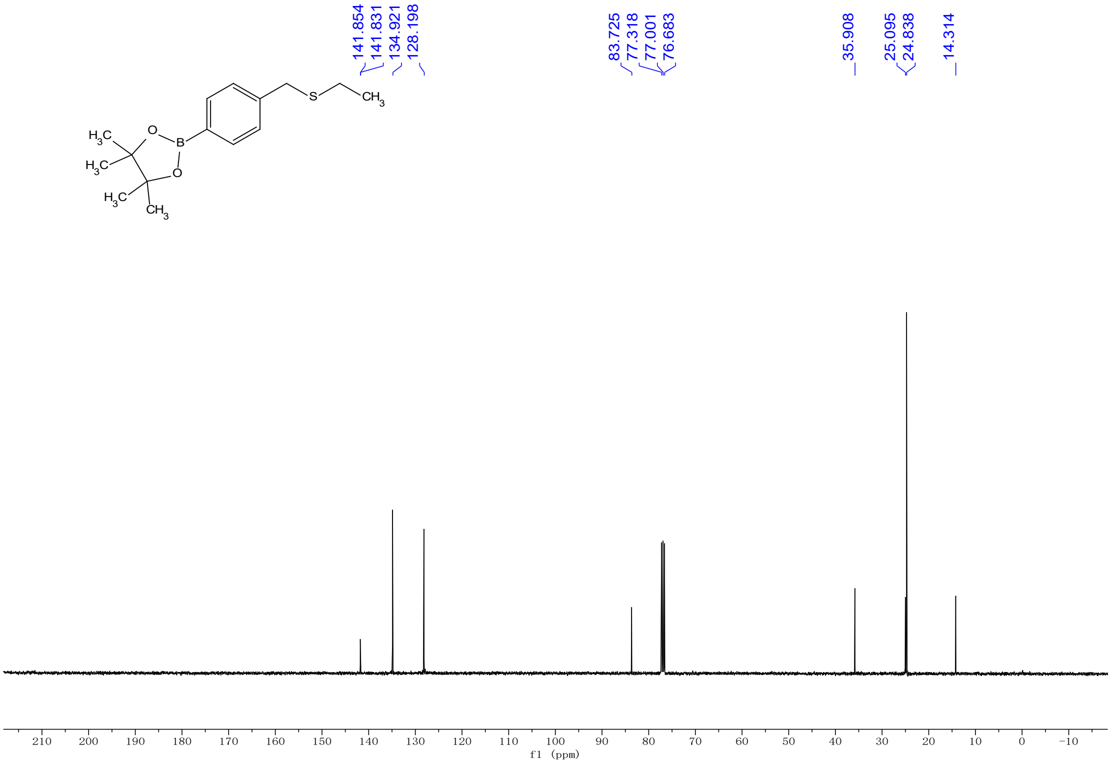

^{1}H NMR (400 MHz, $CDCl_3$) spectrum of compound 3l

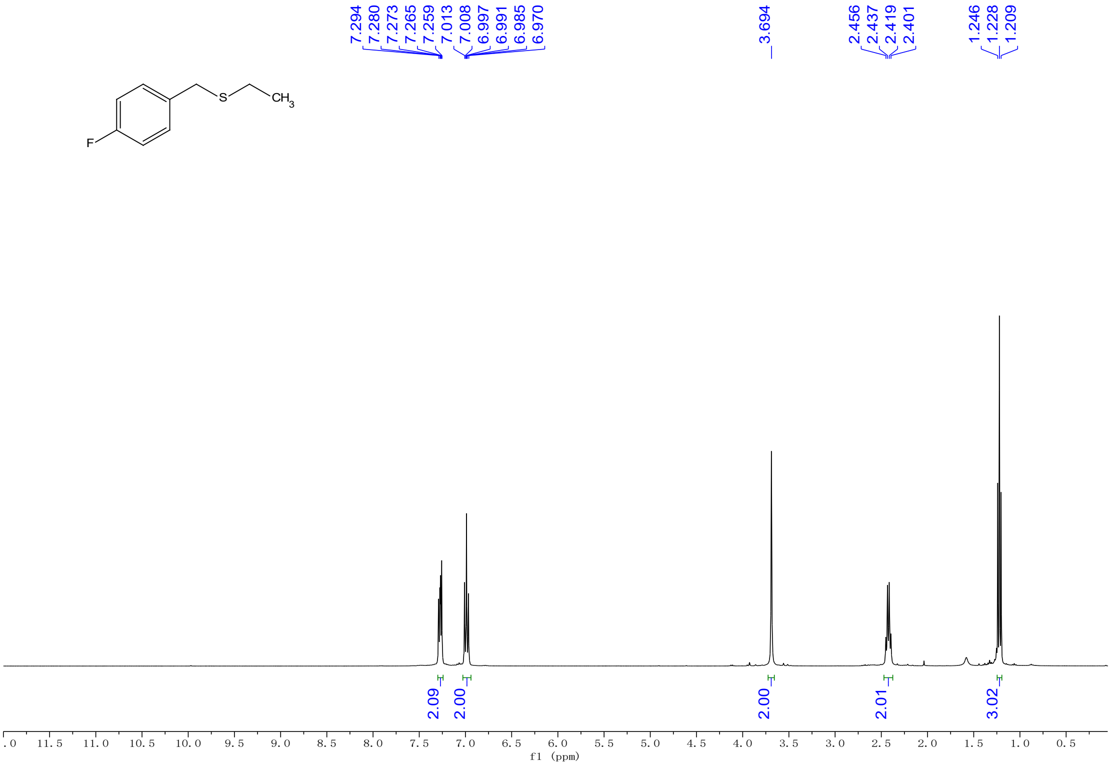

^{13}C NMR (100 MHz, $CDCl_3$) spectrum of compound 3l

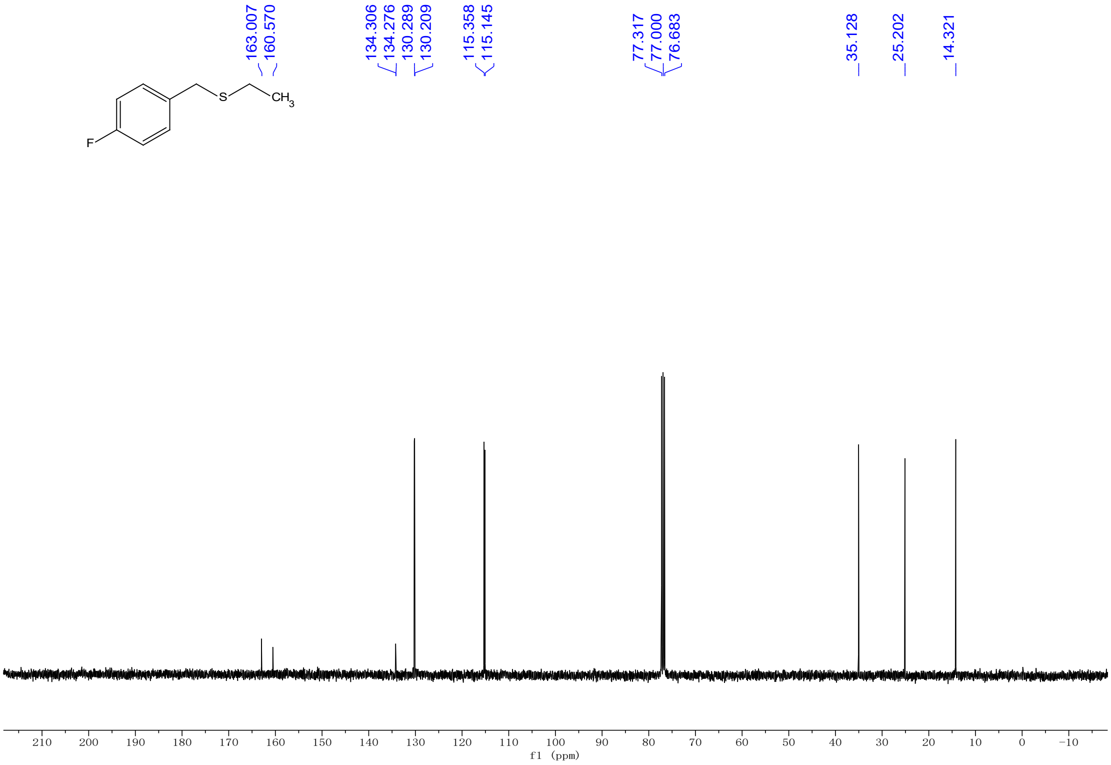

^{19}F NMR (376 MHz, CDCl3) spectrum of compound 3l

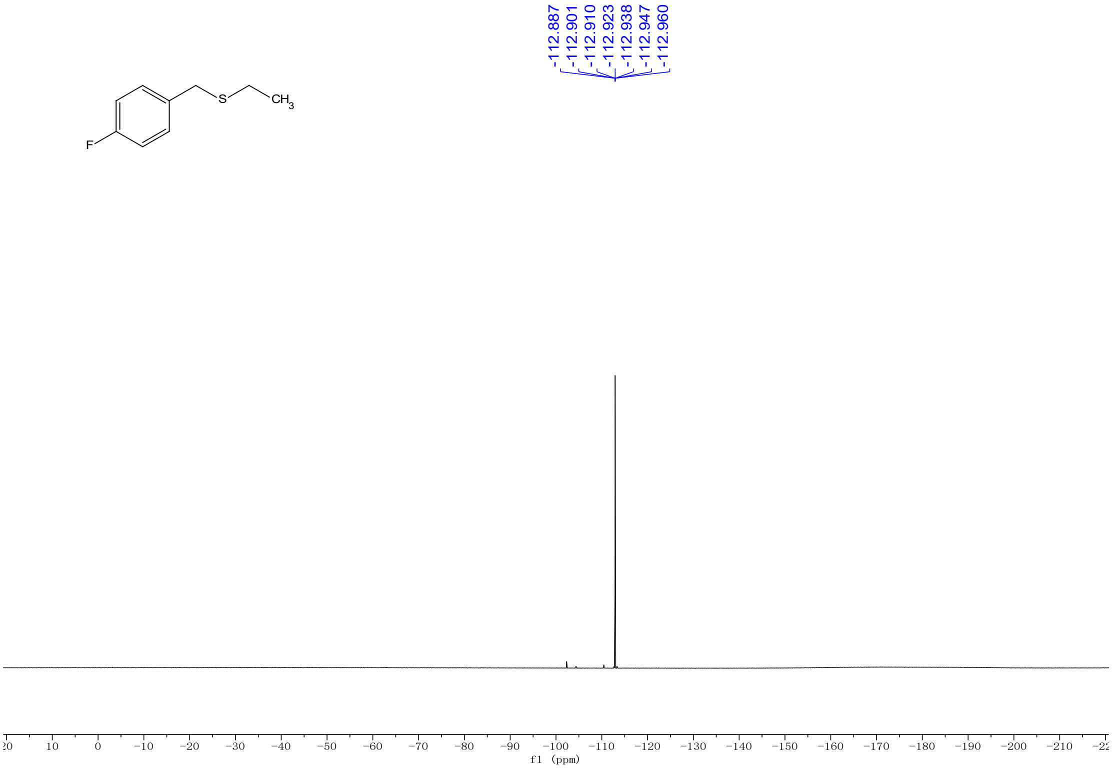

^{1}H NMR (400 MHz, CDCl3) spectrum of compound 3m

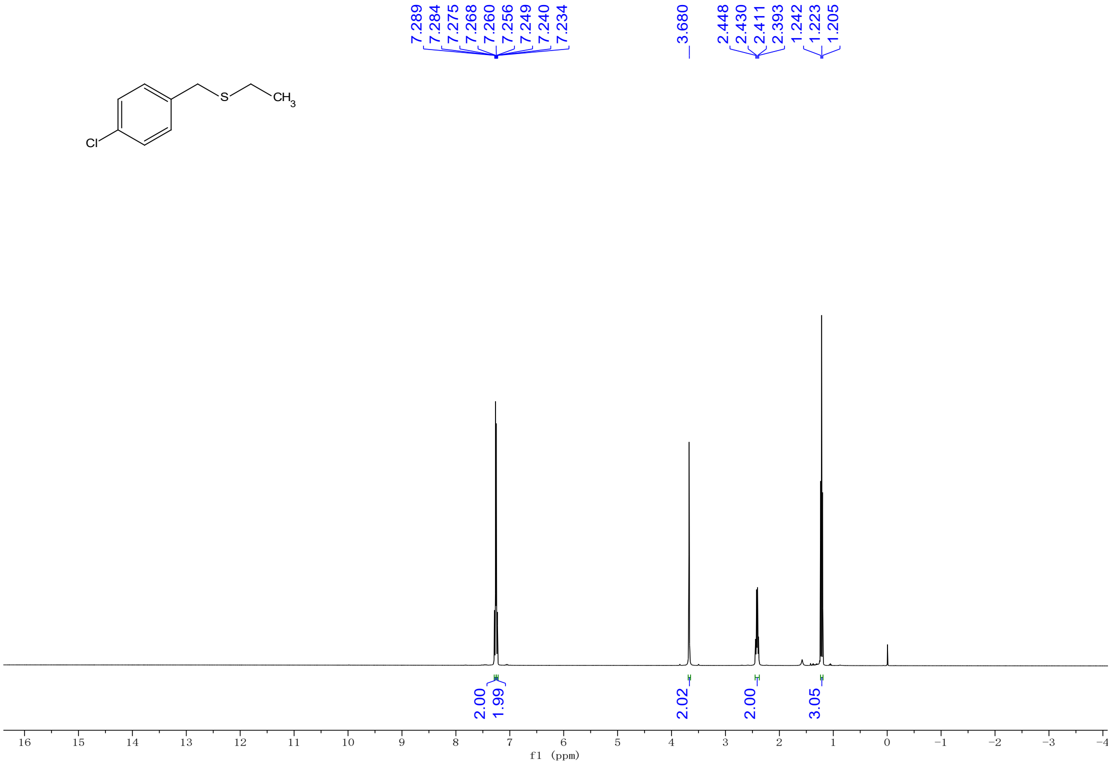

^{13}C NMR (100 MHz, $CDCl_3$) spectrum of compound 3m

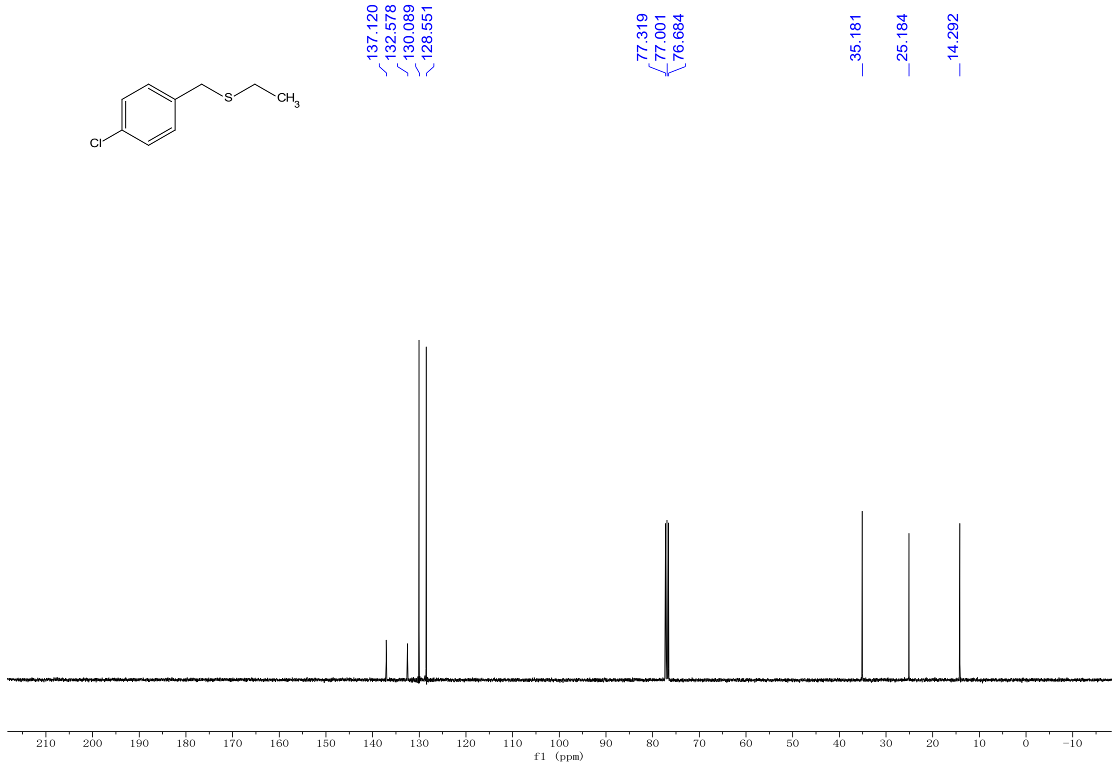

^{1}H NMR (400 MHz, $CDCl_3$) spectrum of compound 3n

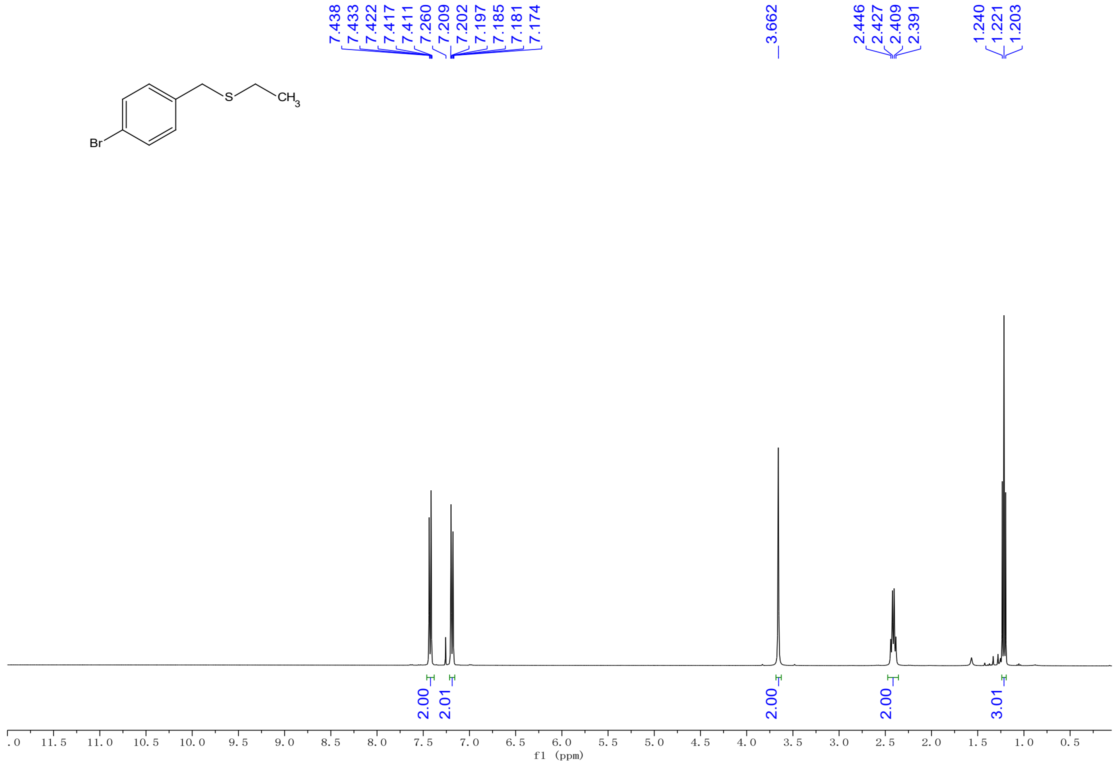

^{13}C NMR (100 MHz, $CDCl_3$) spectrum of compound 3n

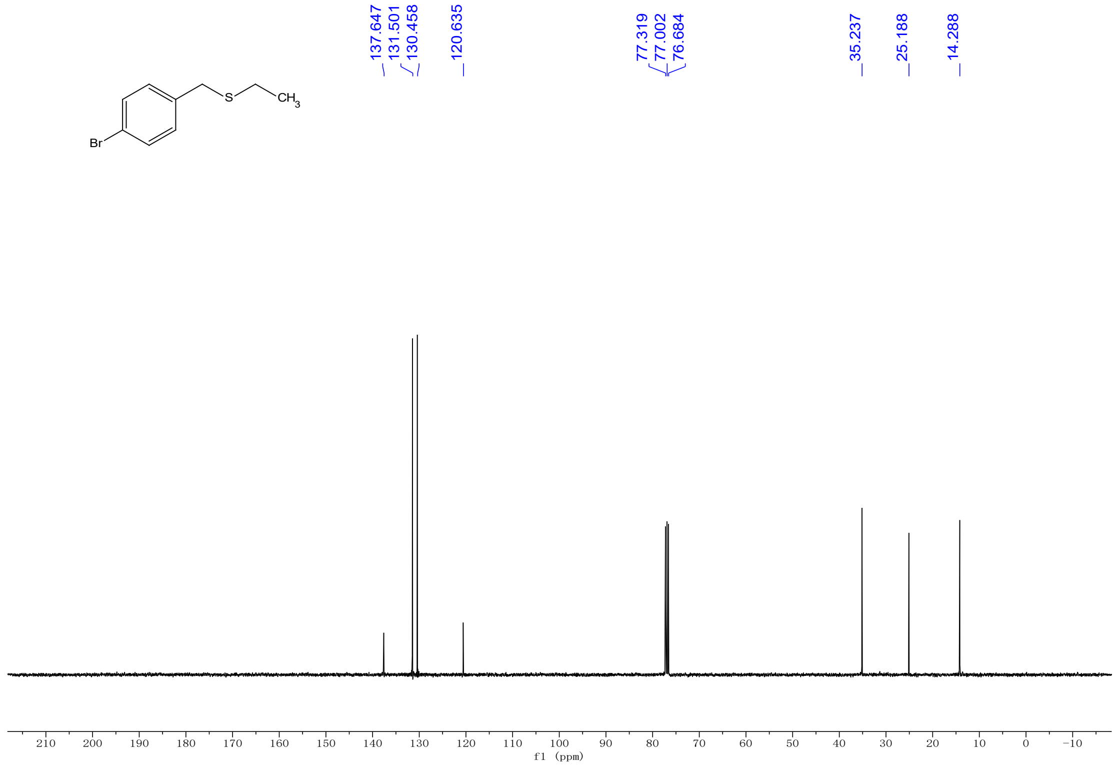

^1H NMR (400 MHz, $CDCl_3$) spectrum of compound 3o

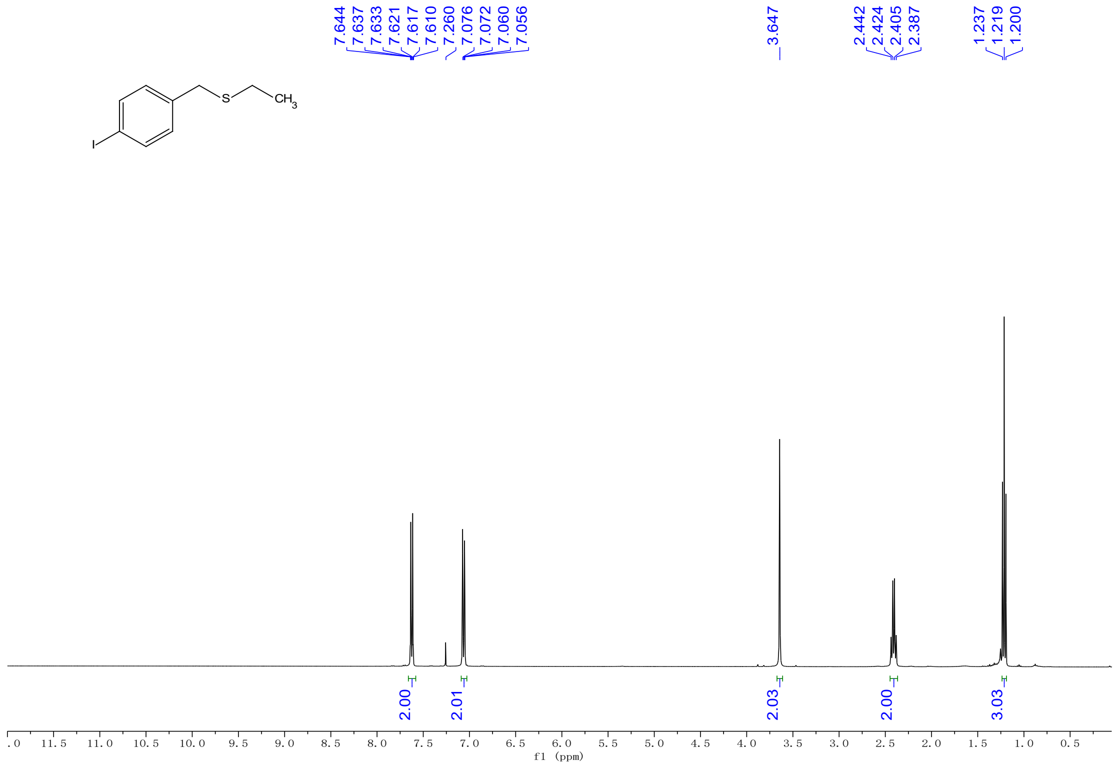

^{13}C NMR (100 MHz, $CDCl_3$) spectrum of compound 3o

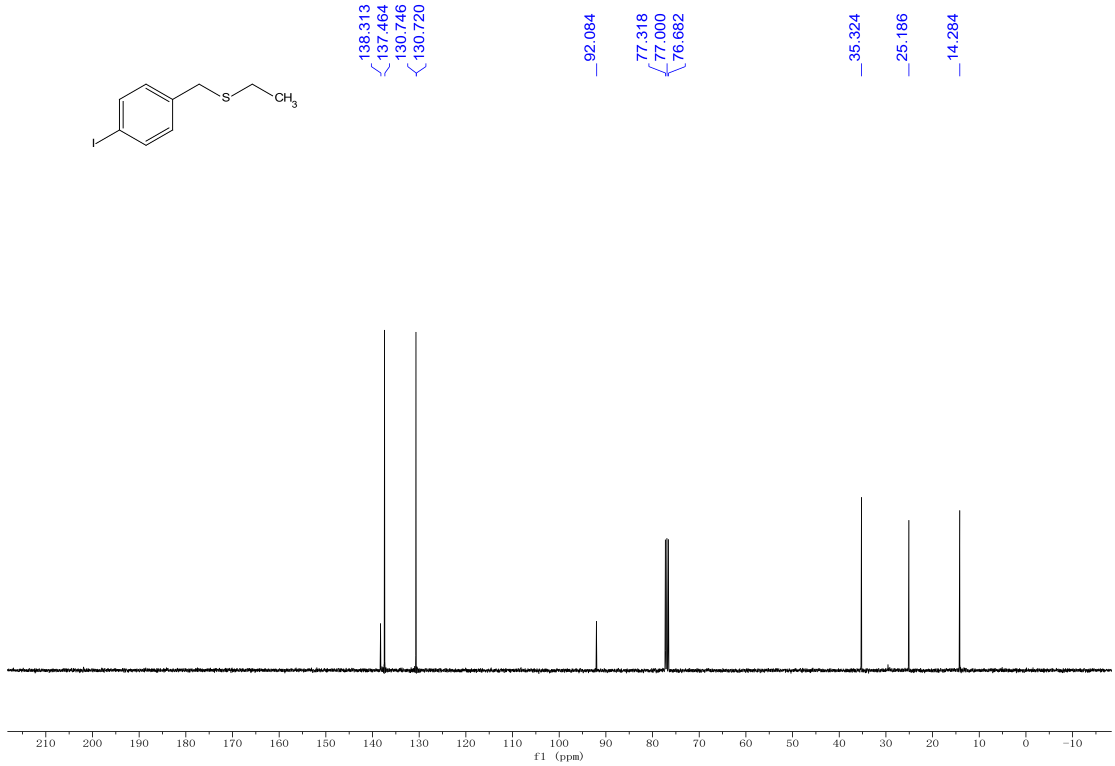

^{1}H NMR (400 MHz, $CDCl_3$) spectrum of compound 3p

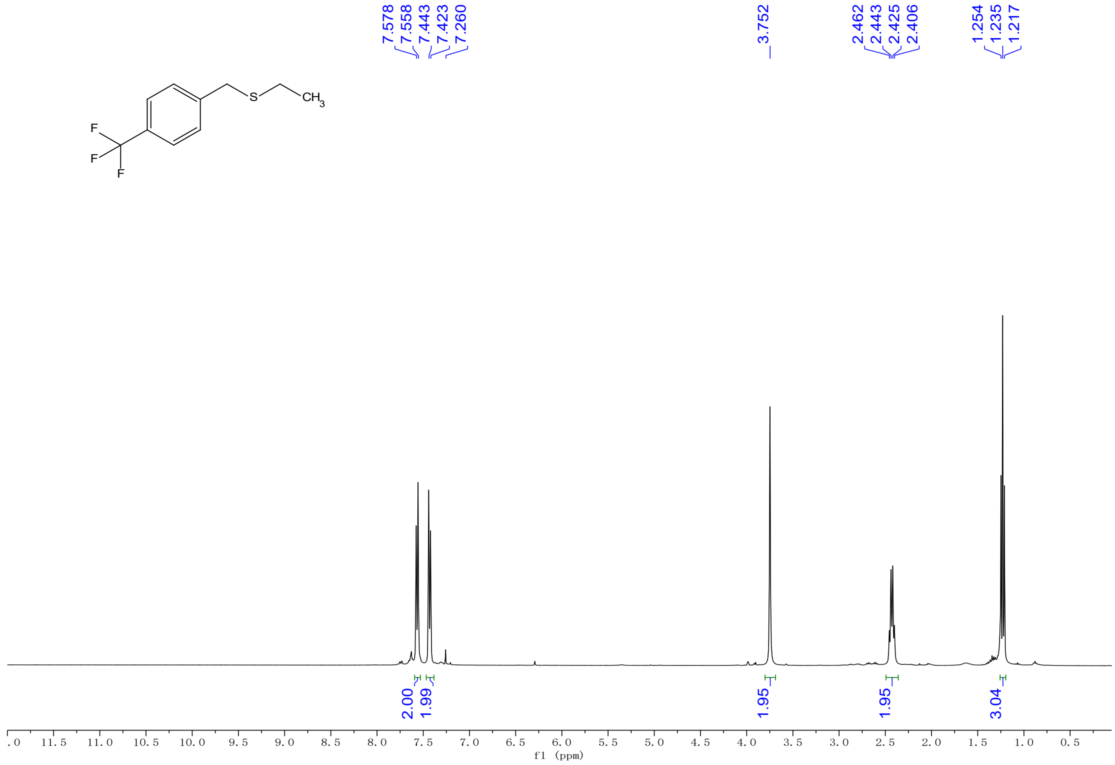

^{13}C NMR (100 MHz, $CDCl_3$) spectrum of compound 3p

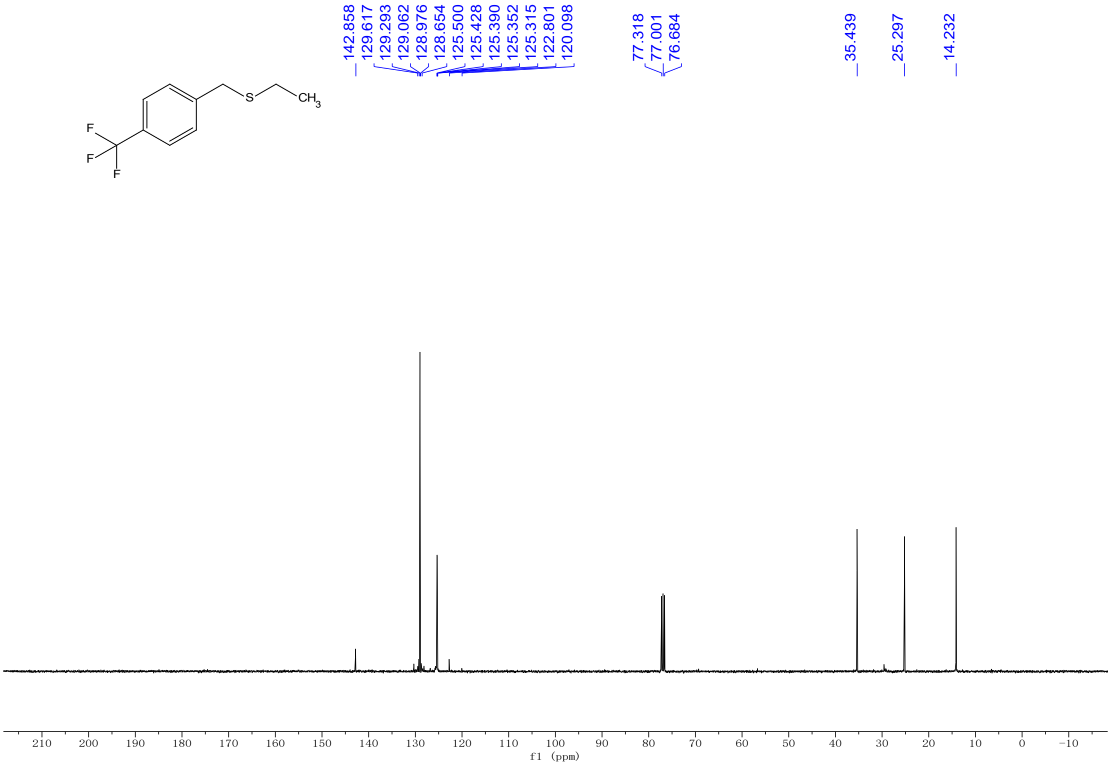

^{13}F NMR (376 MHz, $CDCl_3$) spectrum of compound 3p

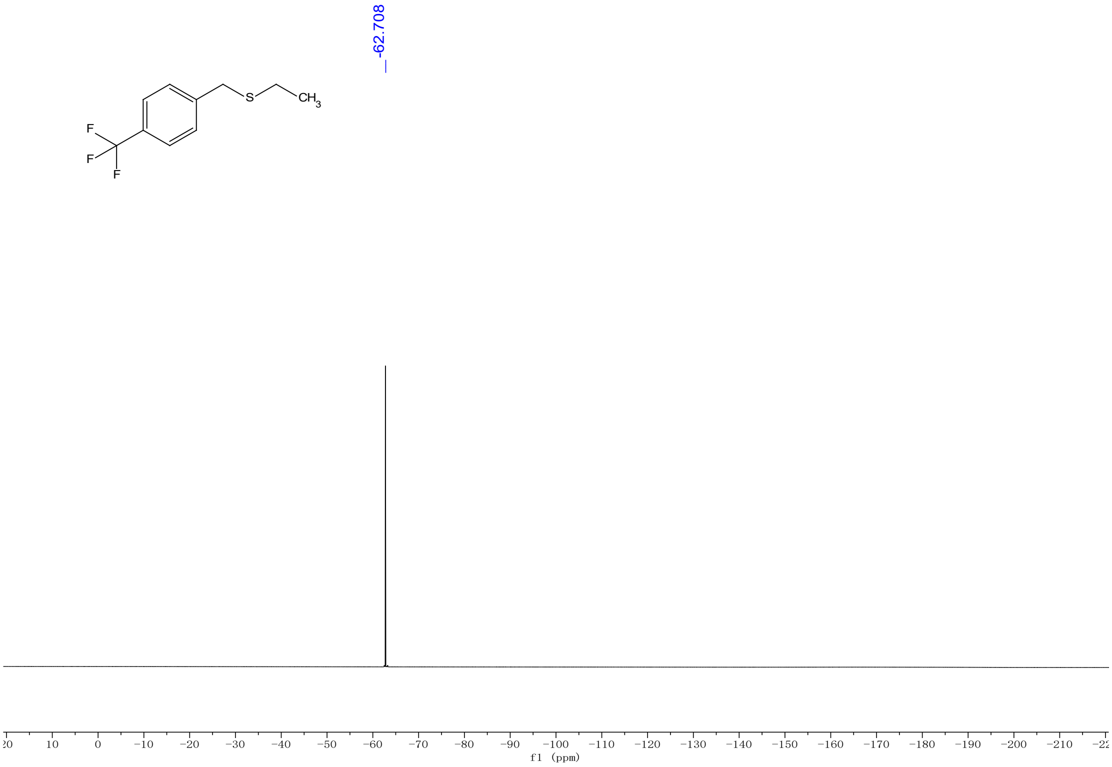

^{1}H NMR (400 MHz, $CDCl_3$) spectrum of compound 3q

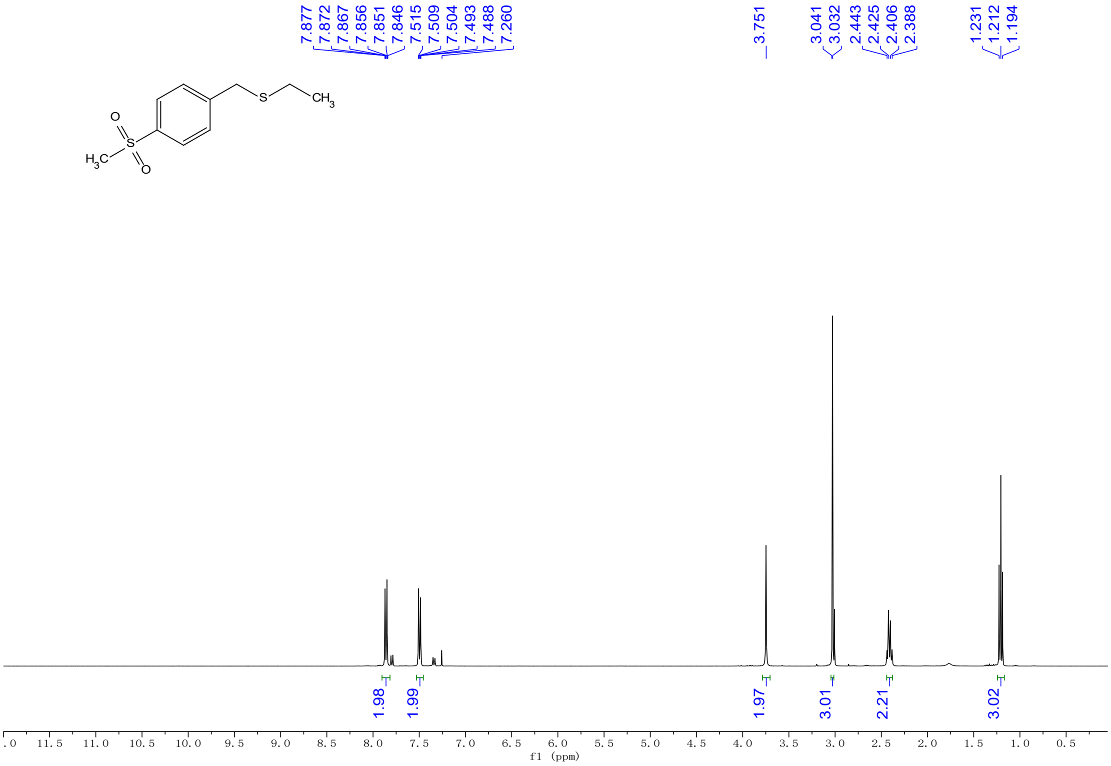

^{13}C NMR (100 MHz, $CDCl_3$) spectrum of compound 3q

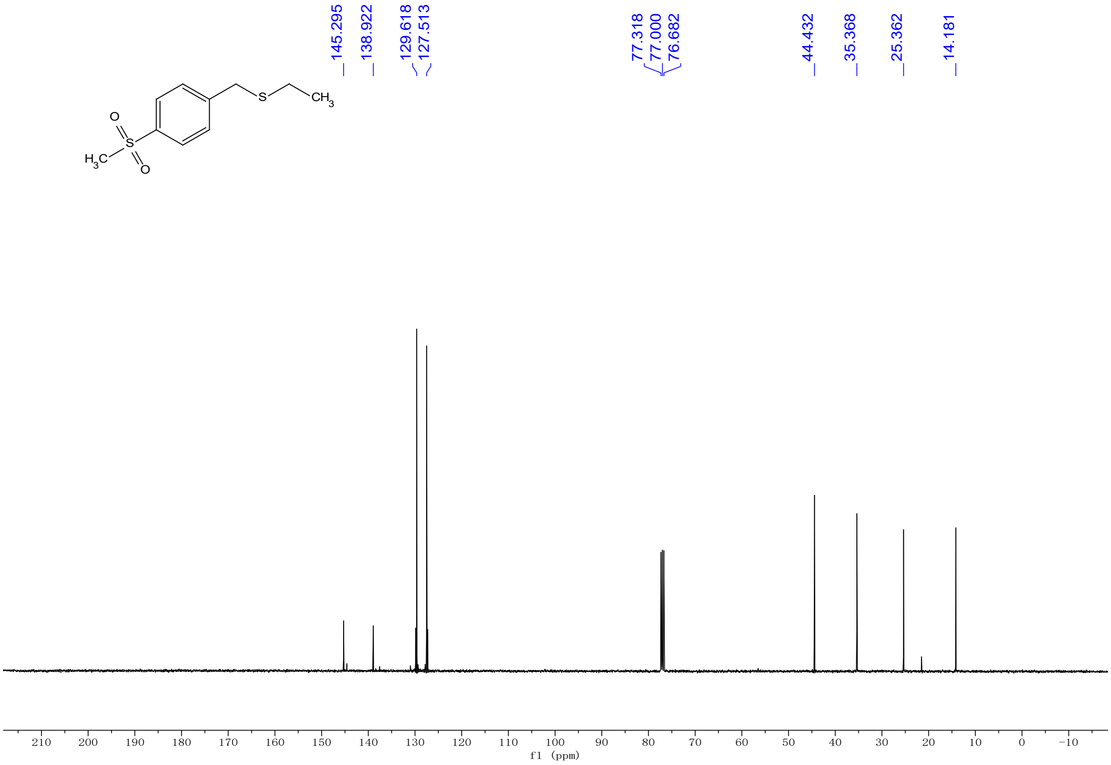

^{1}H NMR (400 MHz, $CDCl_3$) spectrum of compound 3r

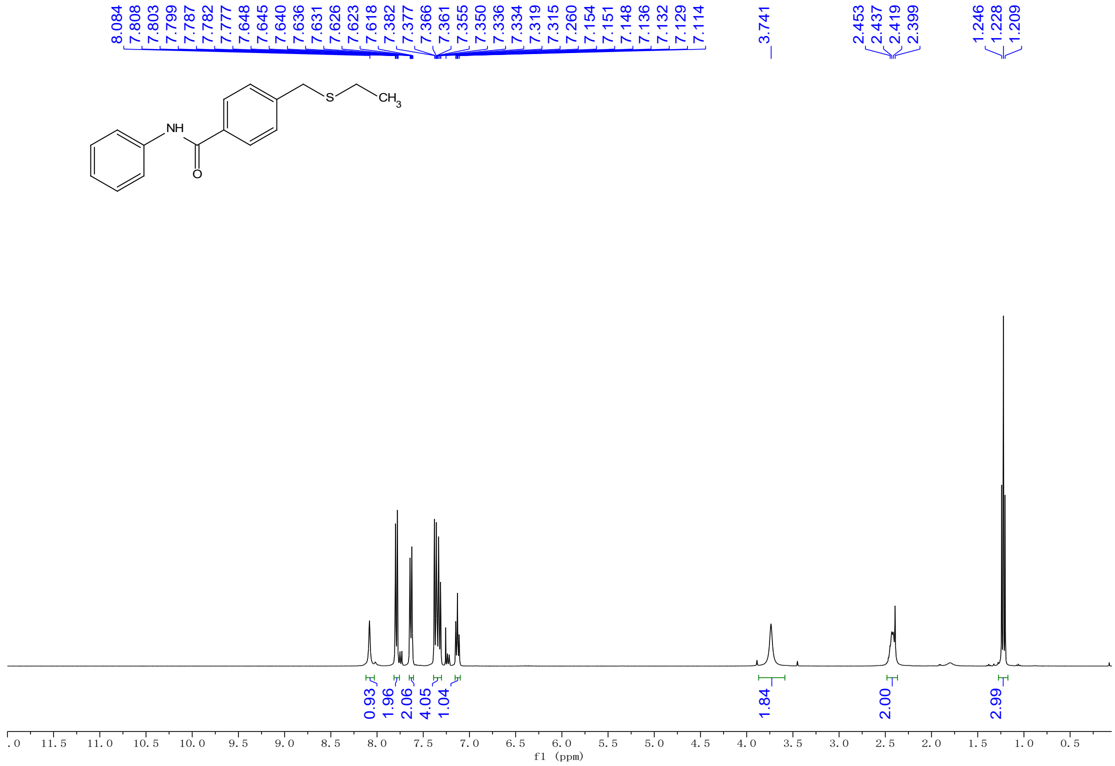

^{13}C NMR (100 MHz, $CDCl_3$) spectrum of compound 3r

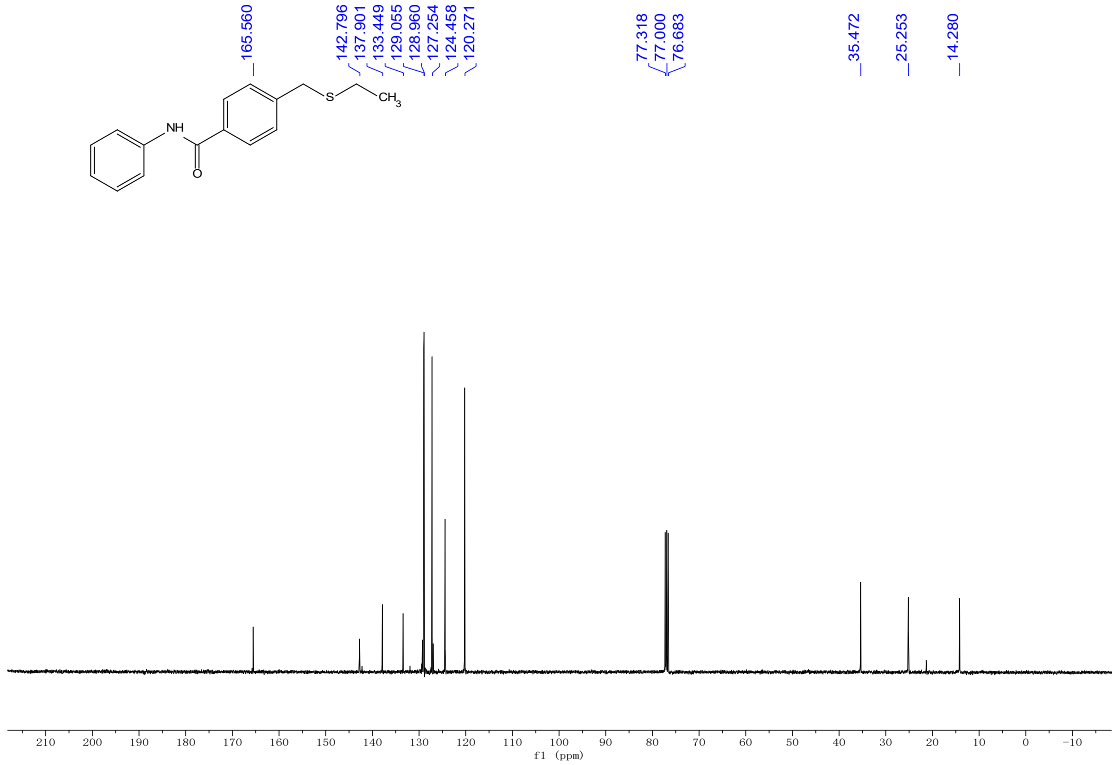

^{1}H NMR (400 MHz, $CDCl_3$) spectrum of compound 3s

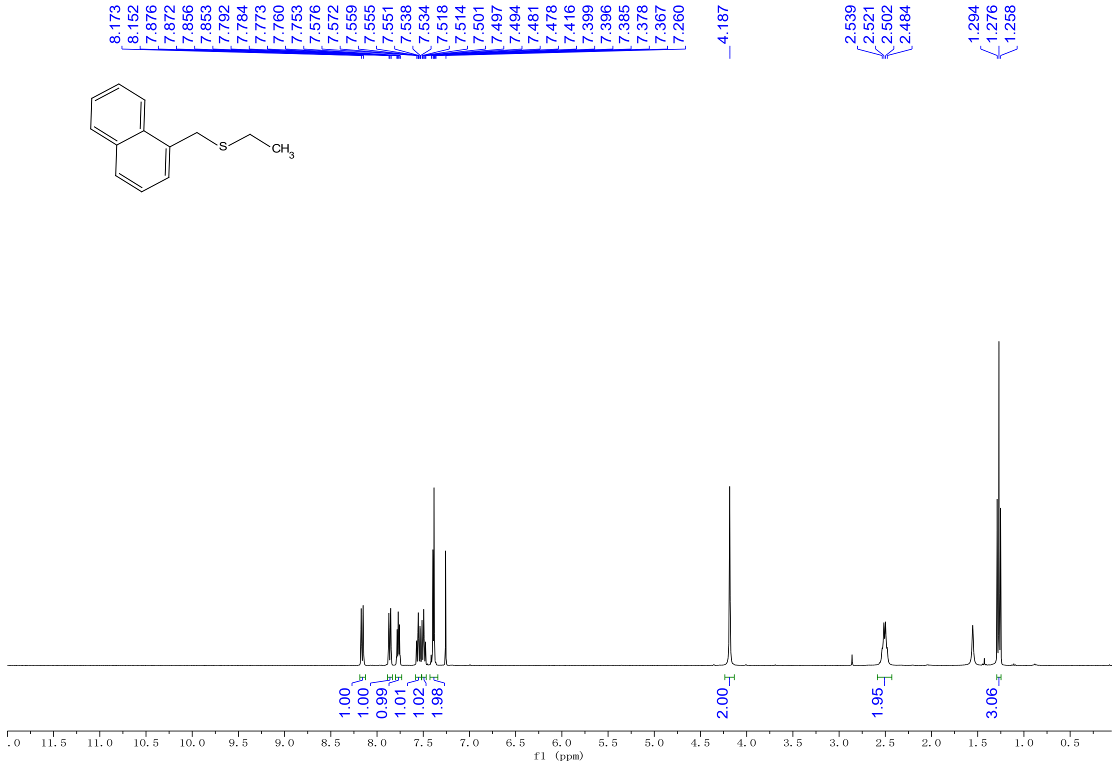

^{13}C NMR (100 MHz, $CDCl_3$) spectrum of compound 3s

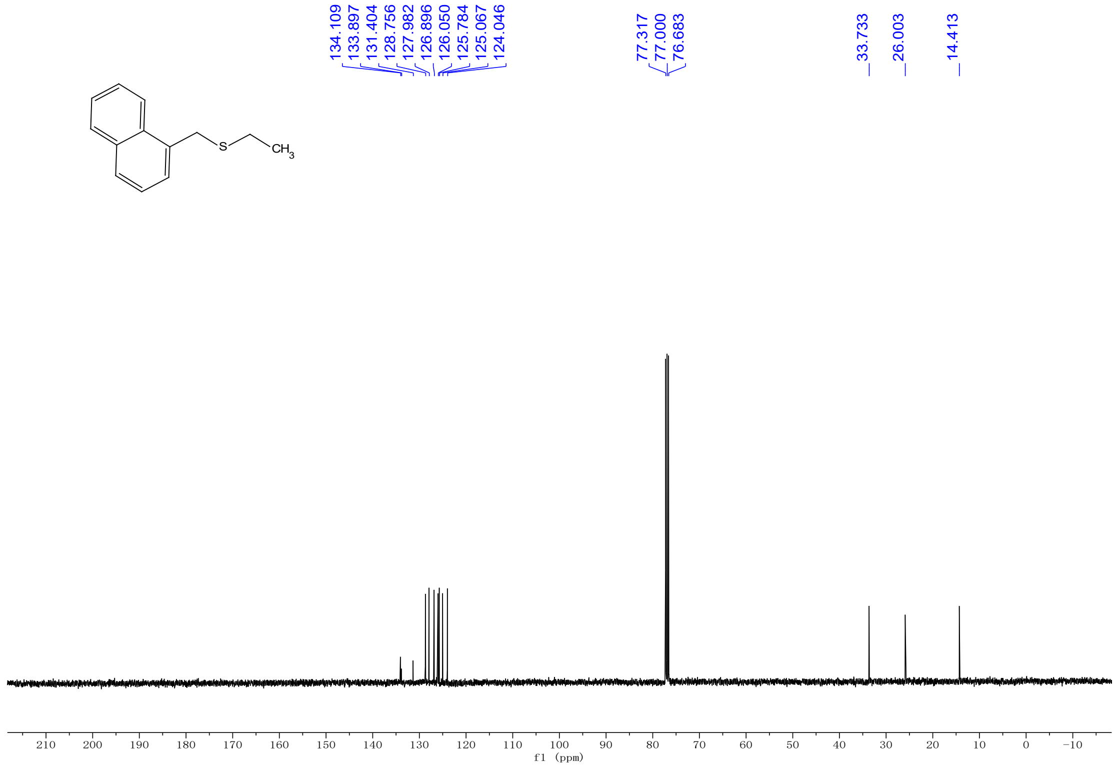

^{1}H NMR (400 MHz, $CDCl_3$) spectrum of compound 3t

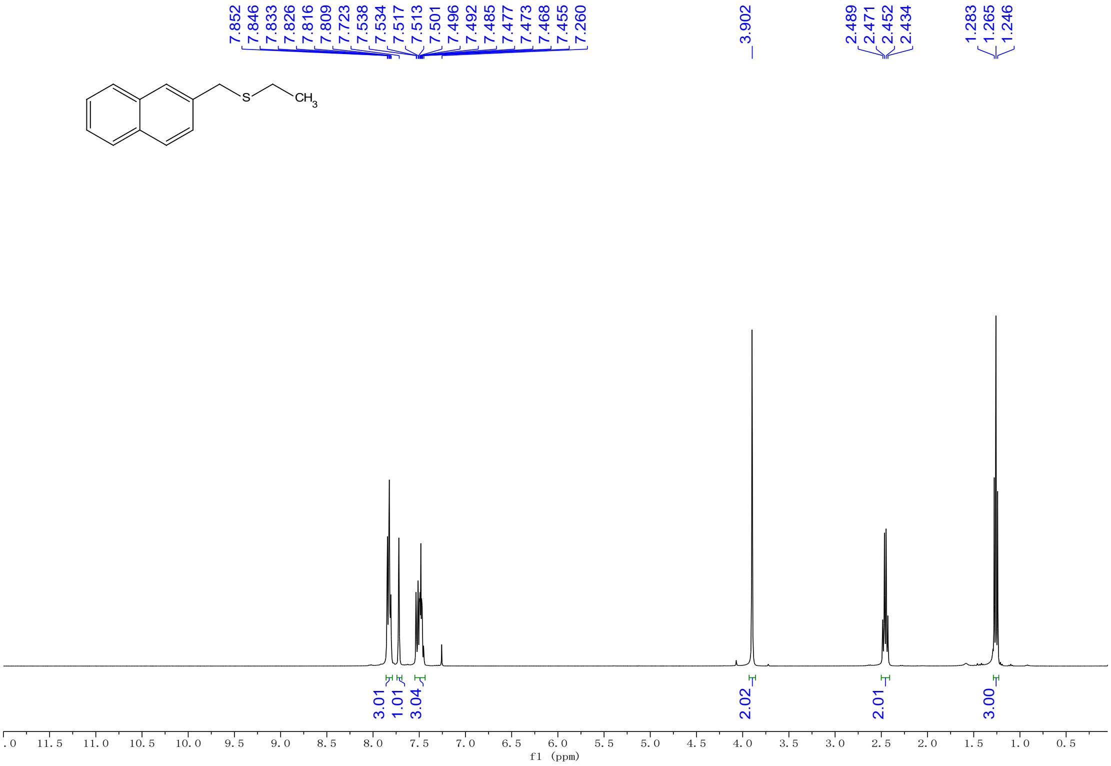

^{13}C NMR (100 MHz, $CDCl_3$) spectrum of compound 3t

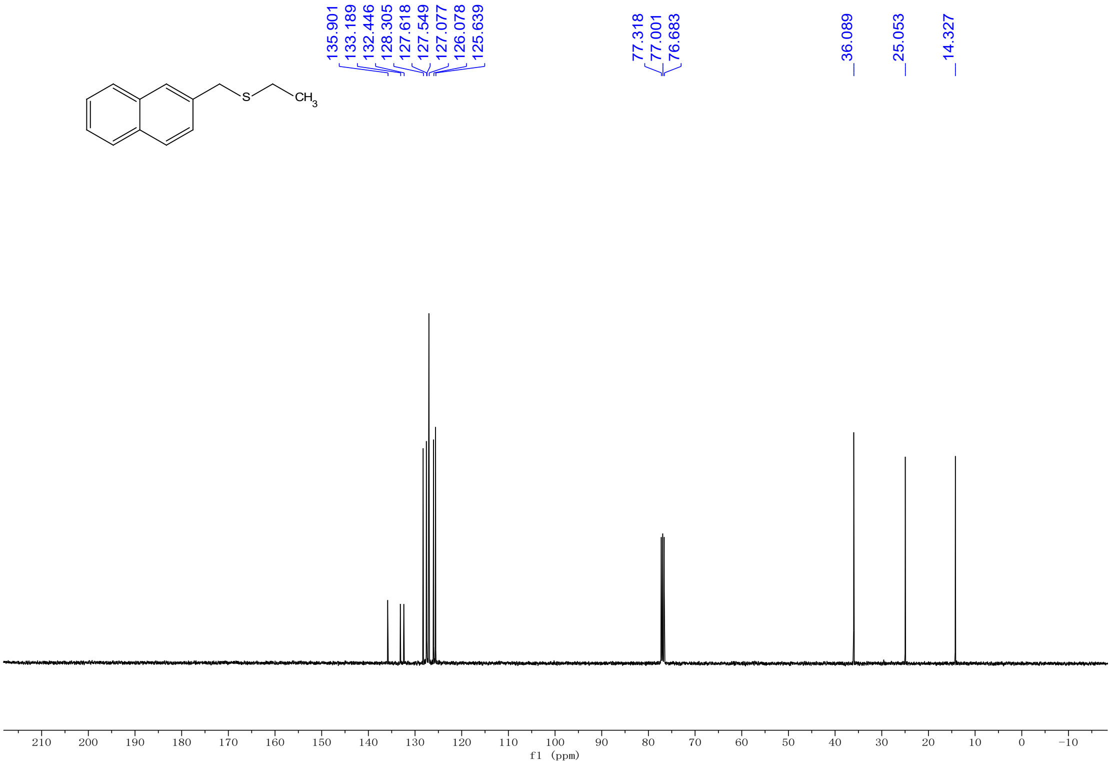

^{1}H NMR (400 MHz, $CDCl_3$) spectrum of compound 3u

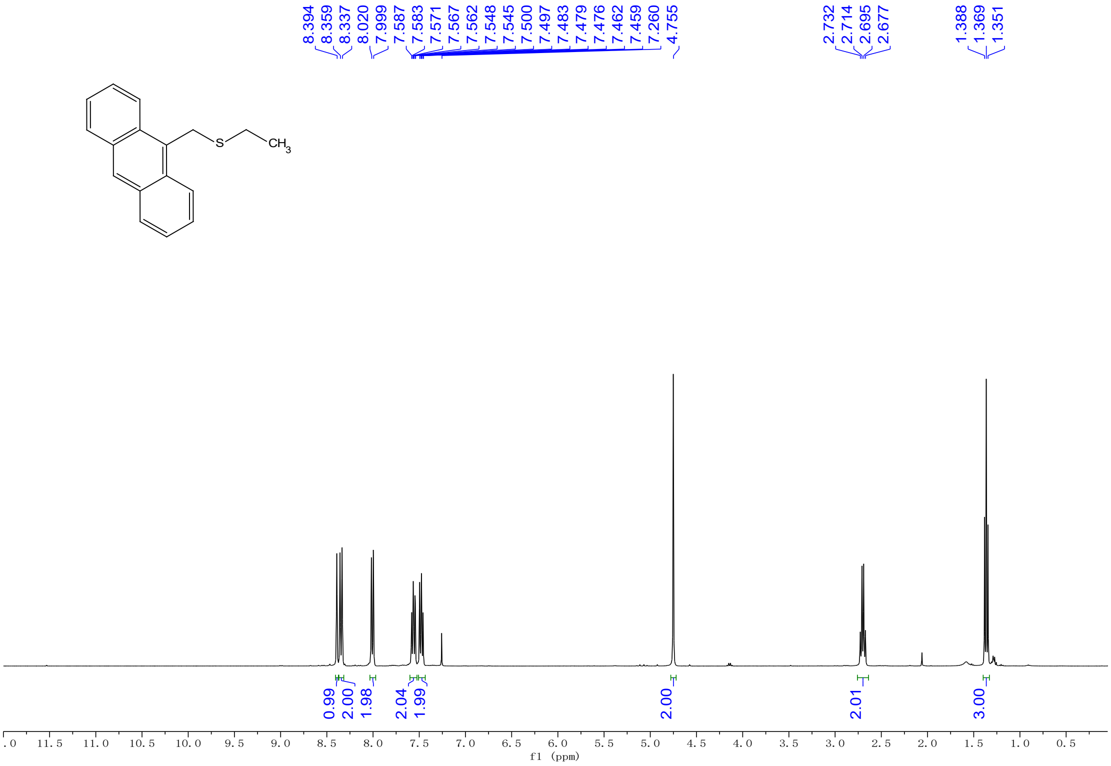

^{13}C NMR (100 MHz, $CDCl_3$) spectrum of compound 3u

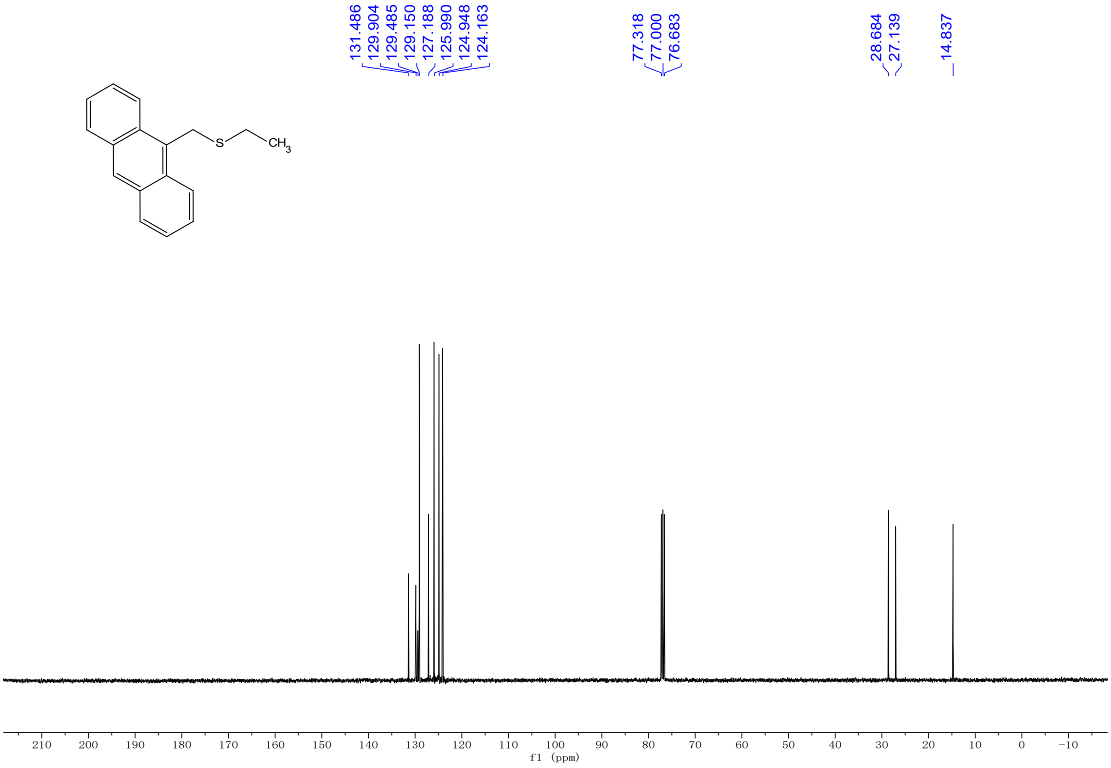

^{1}H NMR (400 MHz, $CDCl_3$) spectrum of compound 3v

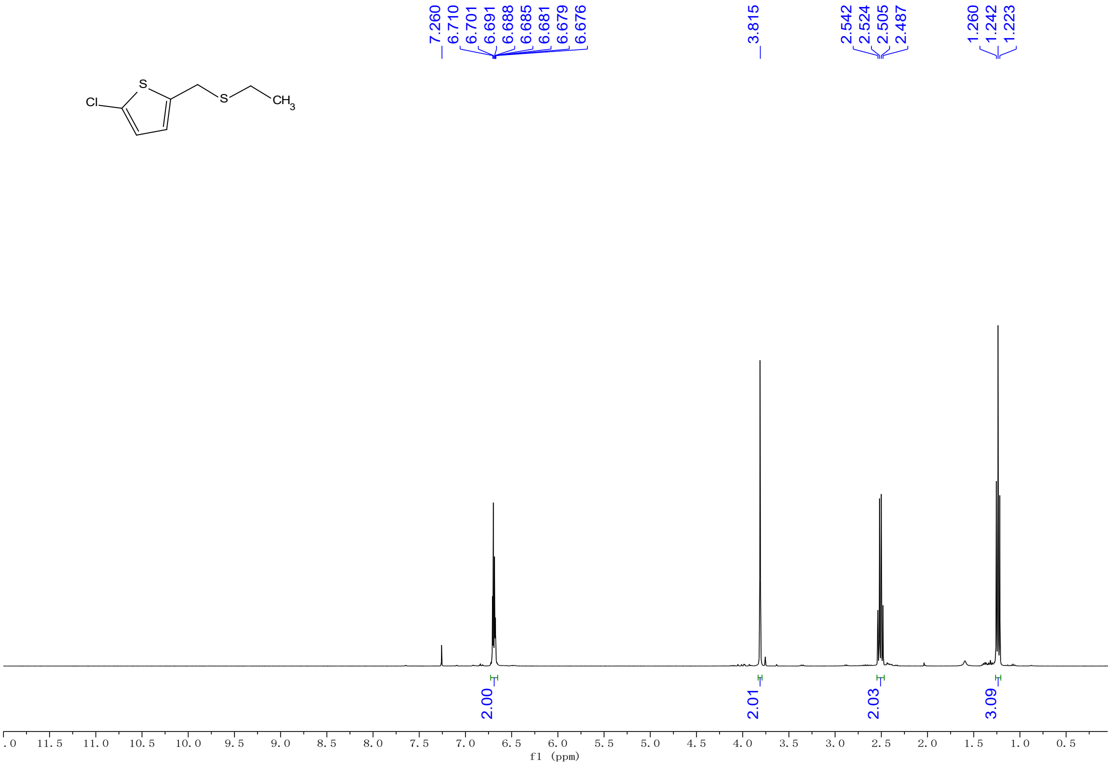

^{13}C NMR (100 MHz, $CDCl_3$) spectrum of compound 3v

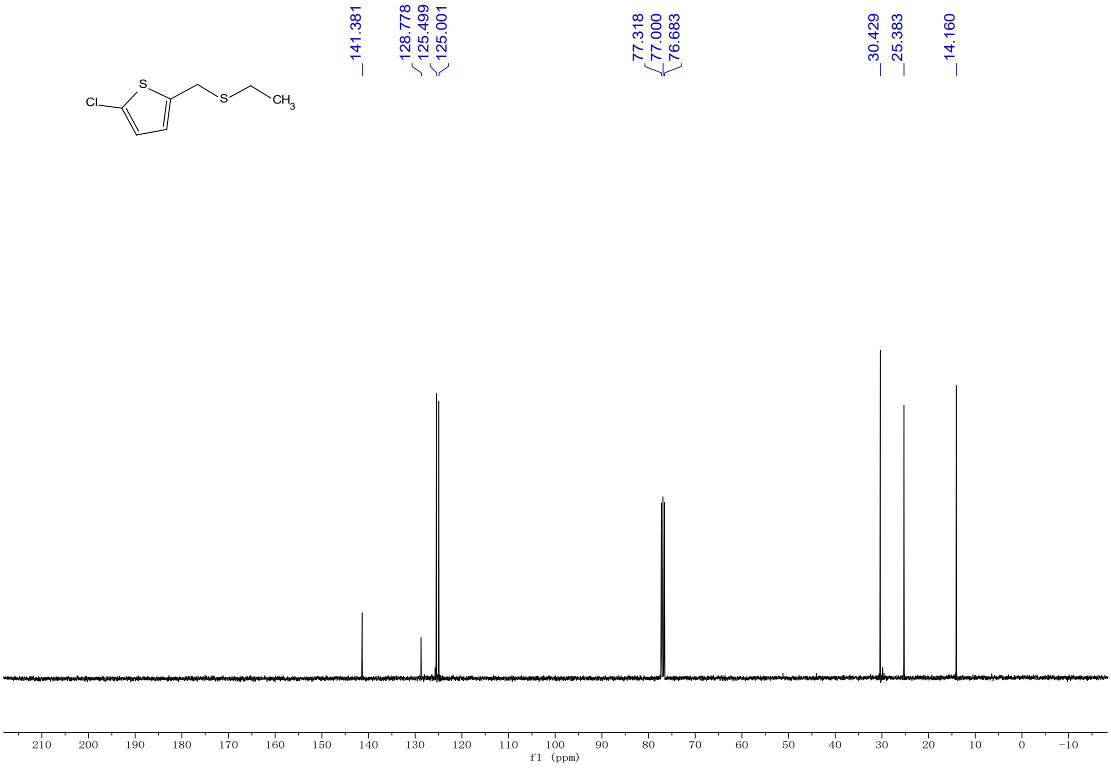

^{1}H NMR (400 MHz, $CDCl_3$) spectrum of compound 3w

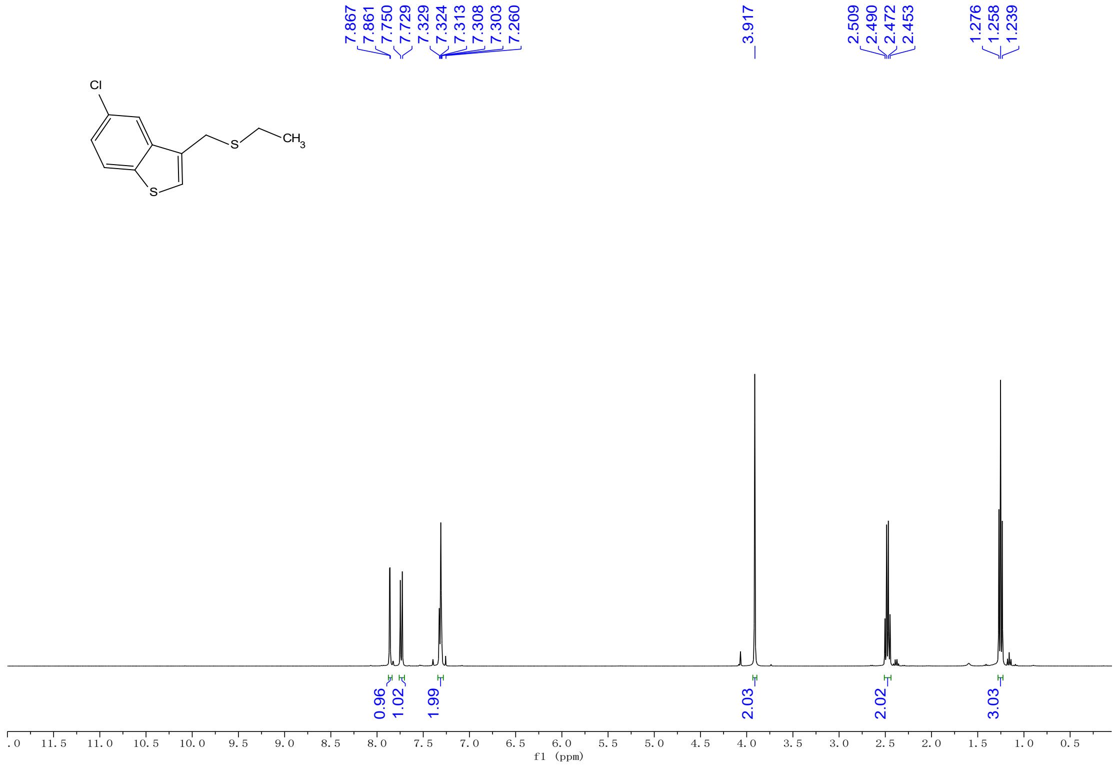

^{13}C NMR (100 MHz, $CDCl_3$) spectrum of compound 3w

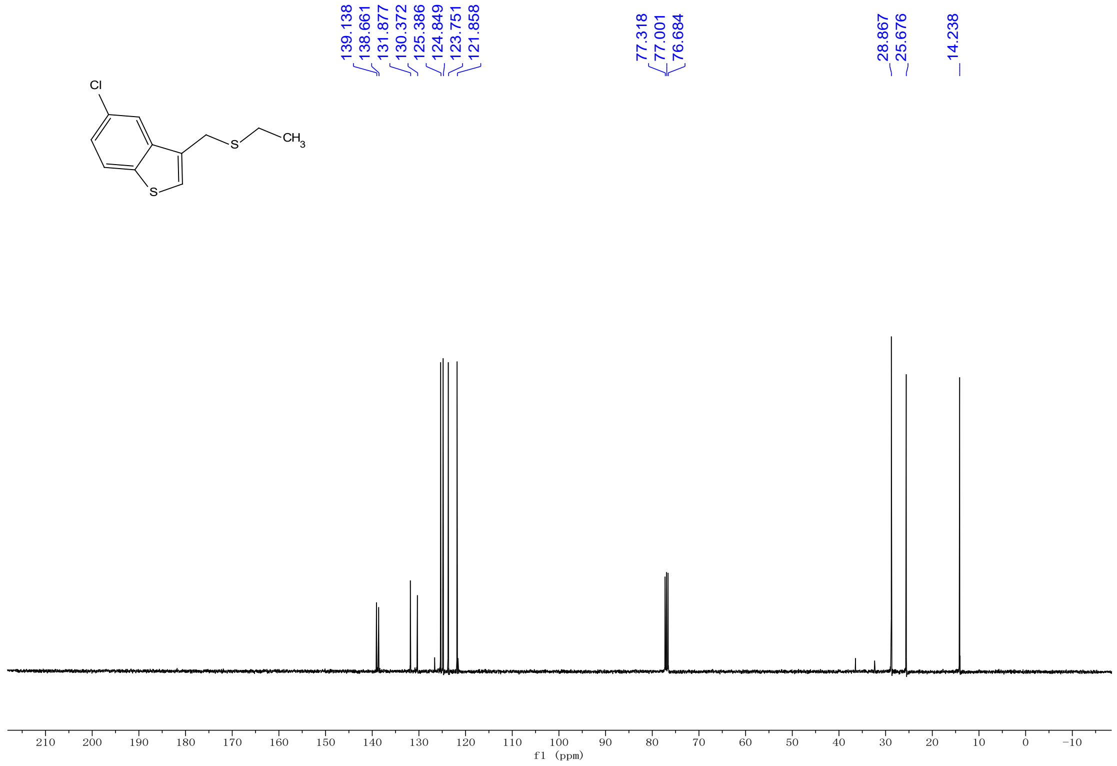

^{1}H NMR (400 MHz, $CDCl_3$) spectrum of compound 3x

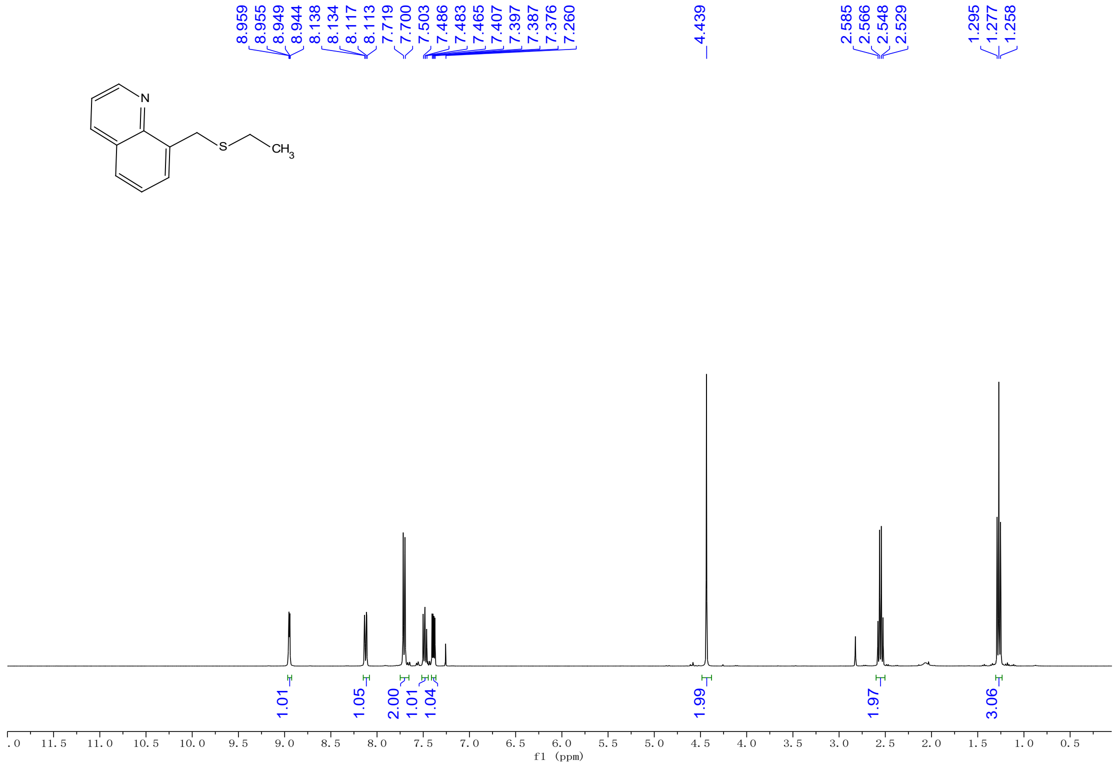

^{13}C NMR (100 MHz, $CDCl_3$) spectrum of compound 3x

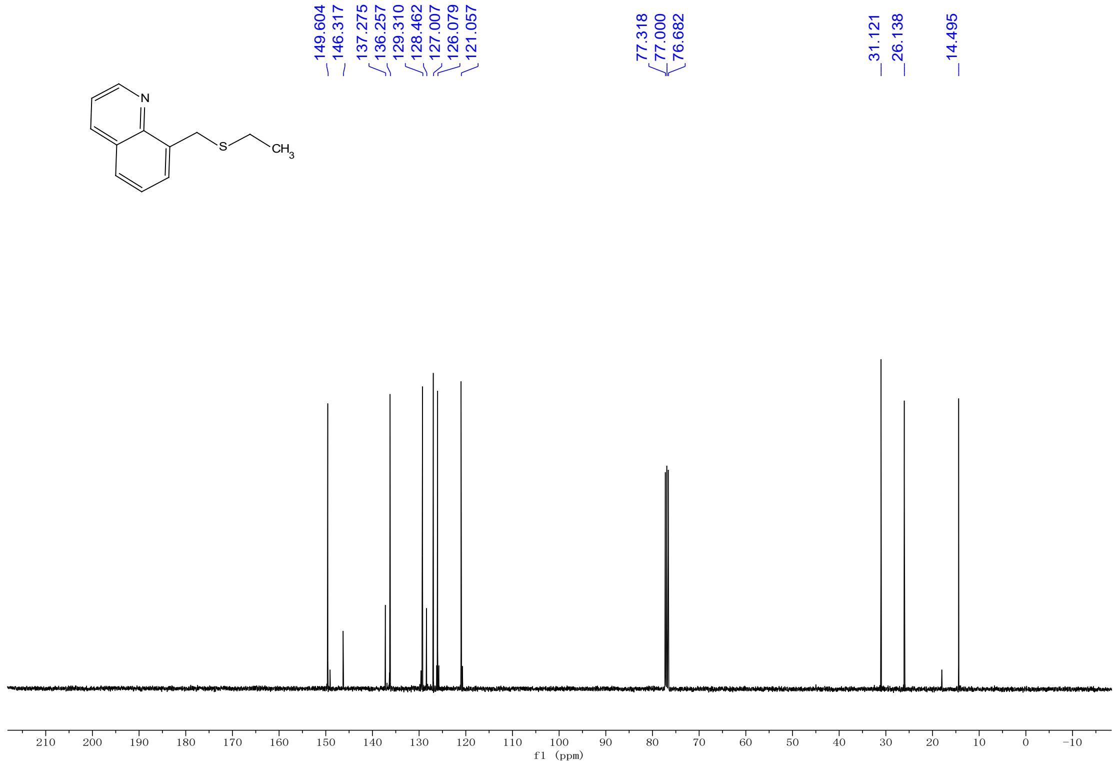

^{1}H NMR (400 MHz, CDCl3) spectrum of compound 3y

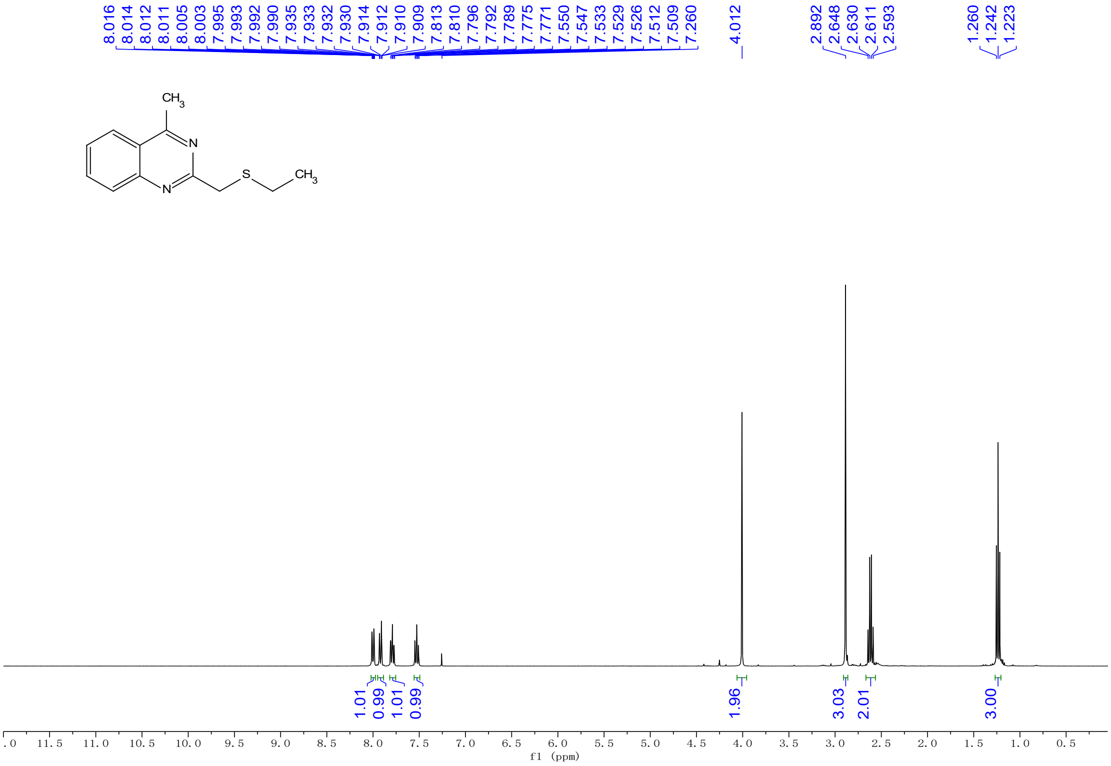

^{13}C NMR (100 MHz, CDCl3) spectrum of compound 3y

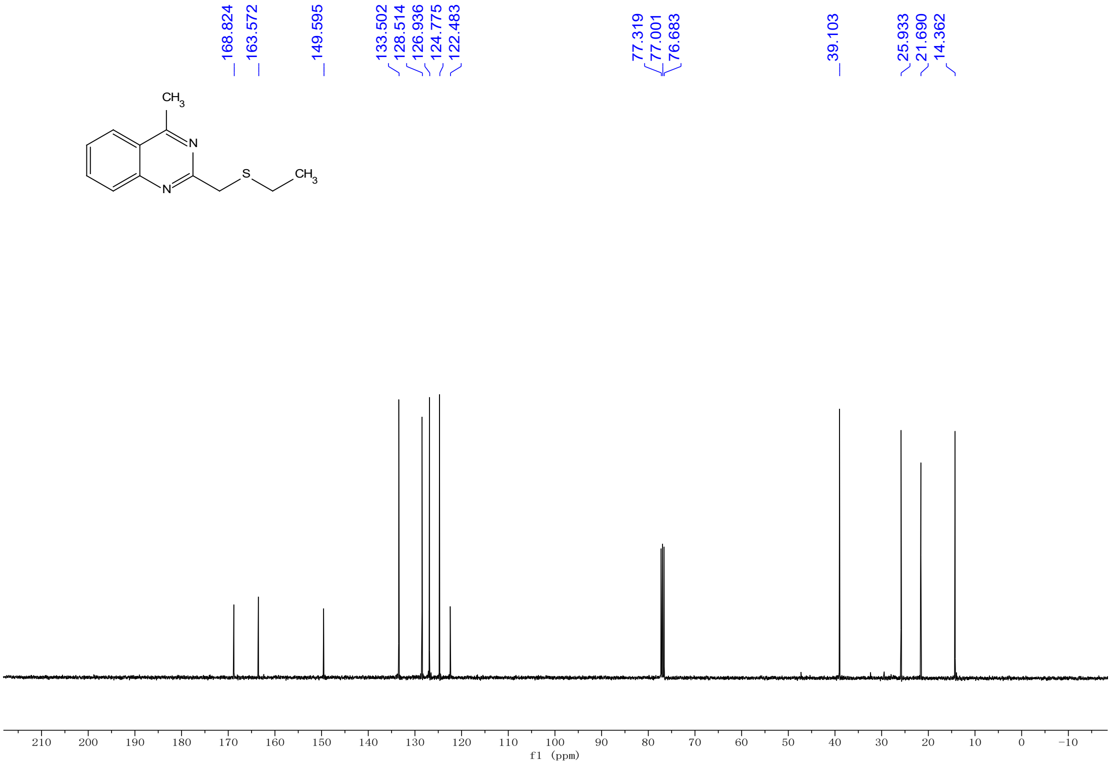

^{1}H NMR (400 MHz, $CDCl_3$) spectrum of compound 3z

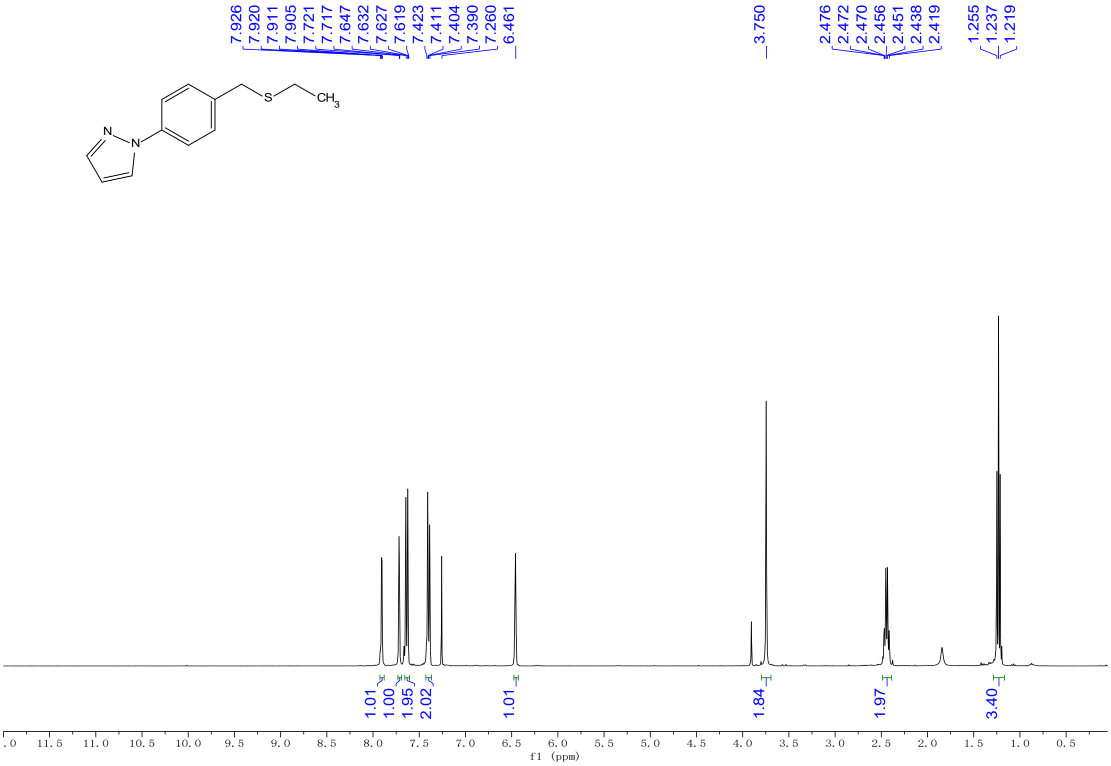

^{13}C NMR (100 MHz, $CDCl_3$) spectrum of compound 3z

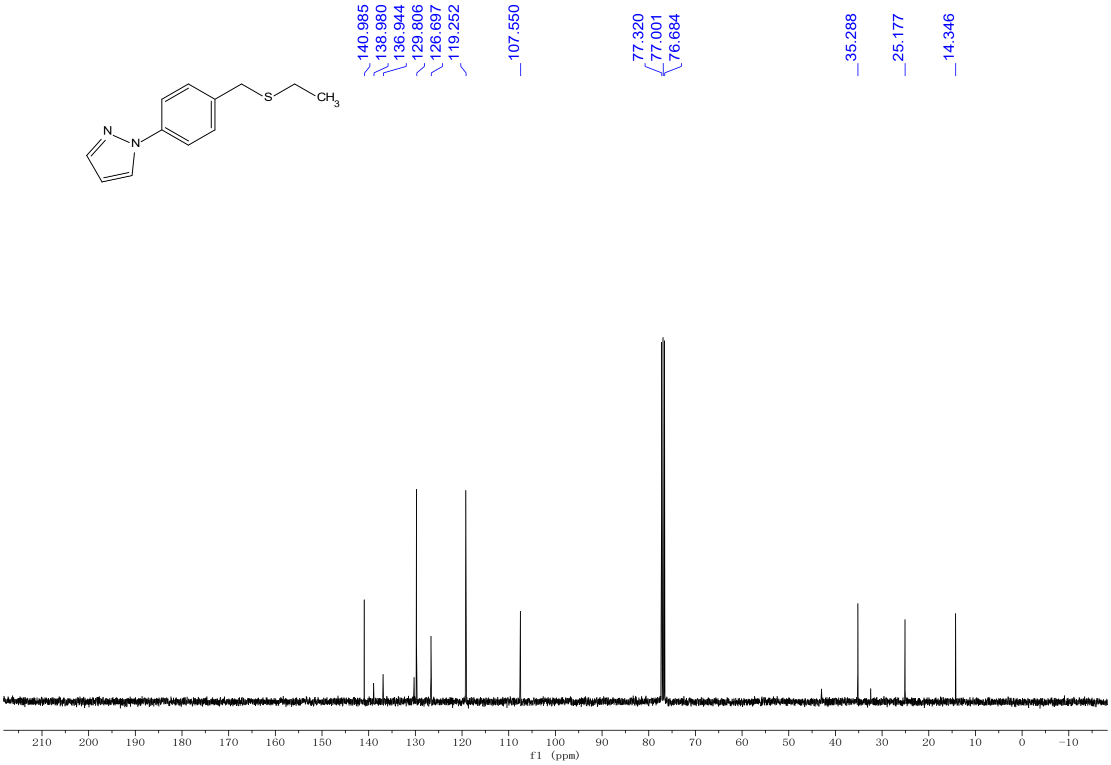

^{1}H NMR (400 MHz, $CDCl_3$) spectrum of compound 3aa

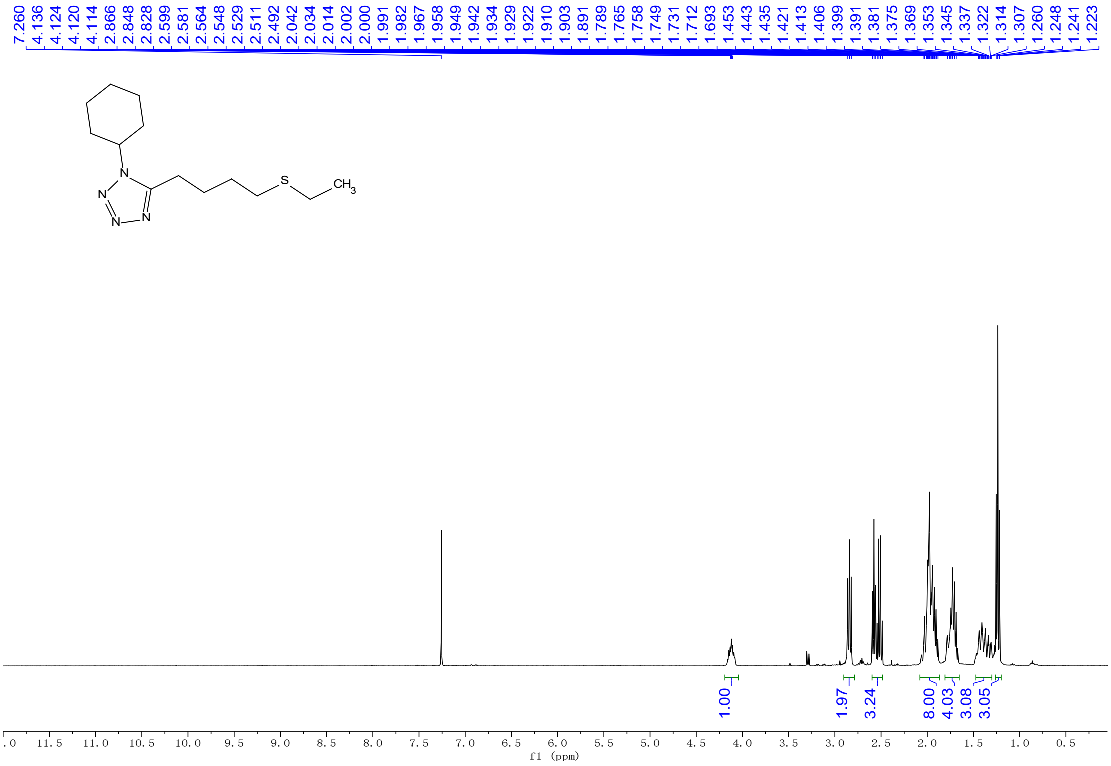

^{13}C NMR (100 MHz, $CDCl_3$) spectrum of compound 3aa

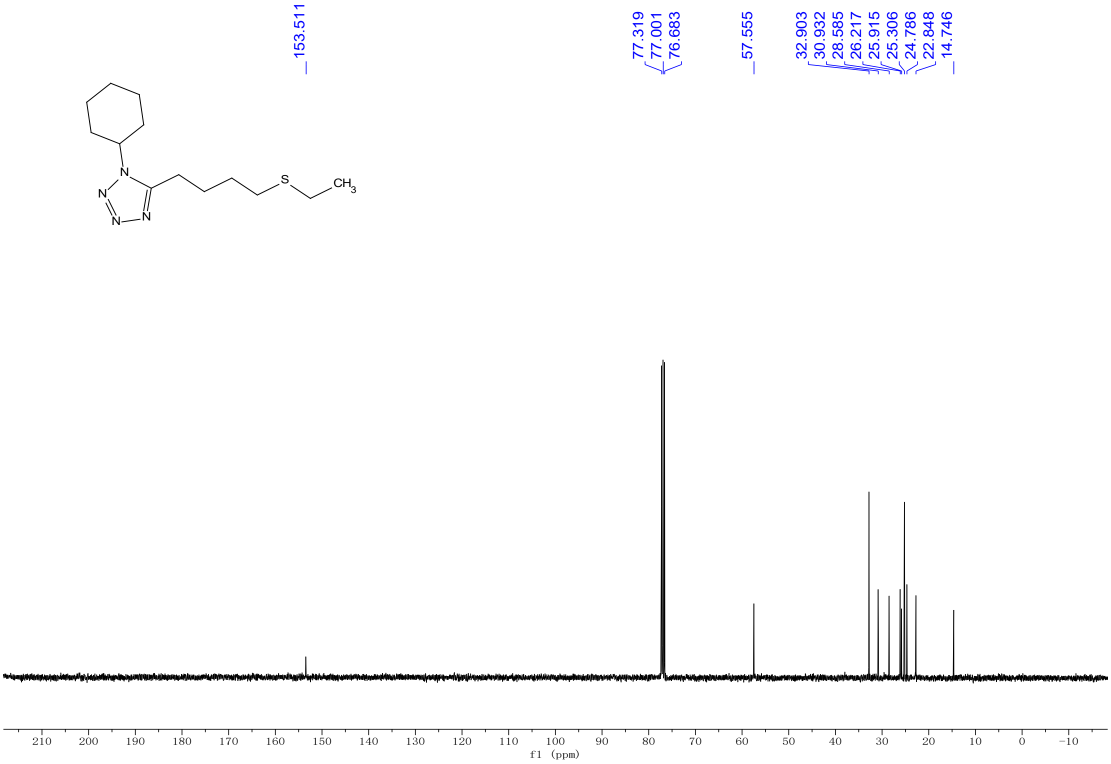

^{1}H NMR (400 MHz, $CDCl_3$) spectrum of compound 3ab

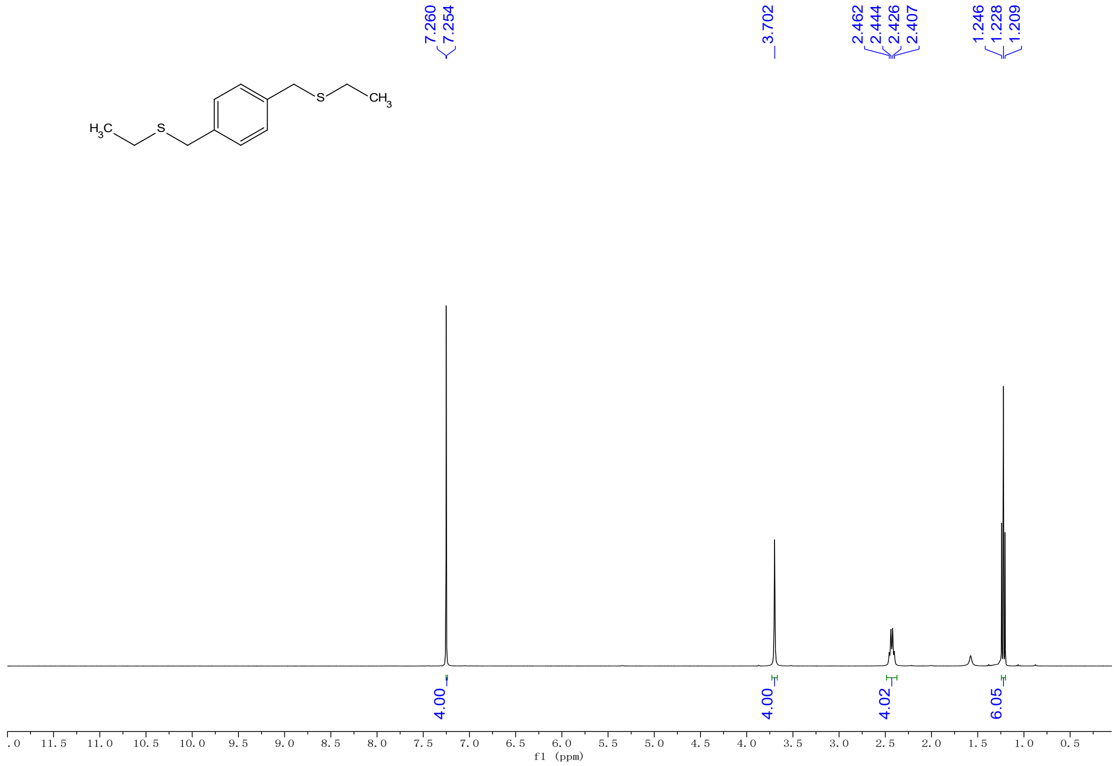

^{13}C NMR (100 MHz, $CDCl_3$) spectrum of compound 3ab

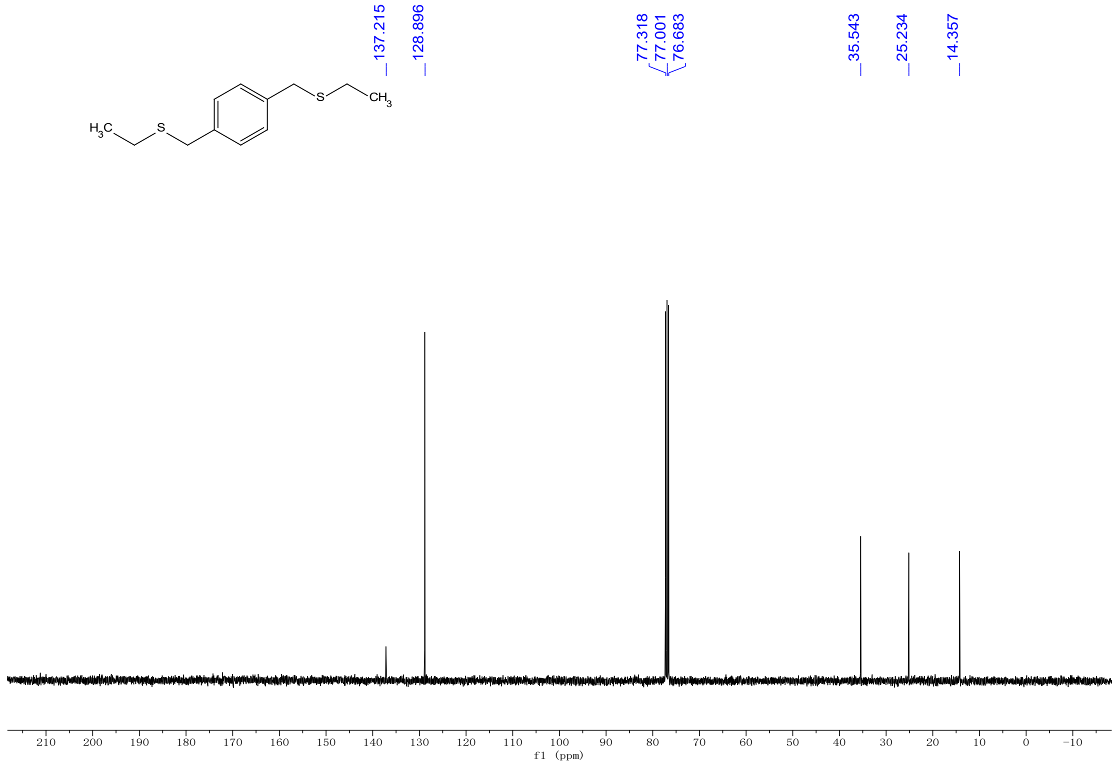

^{1}H NMR (400 MHz, $CDCl_3$) spectrum of compound 3ac

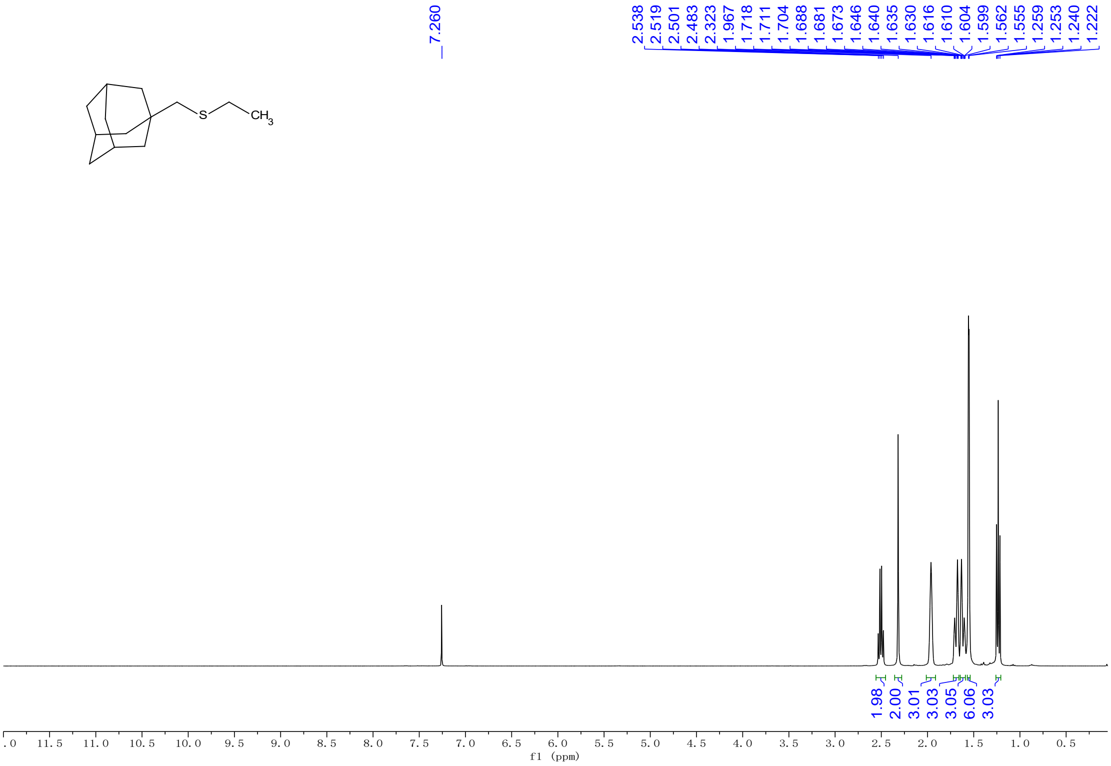

^{13}C NMR (100 MHz, $CDCl_3$) spectrum of compound 3ac

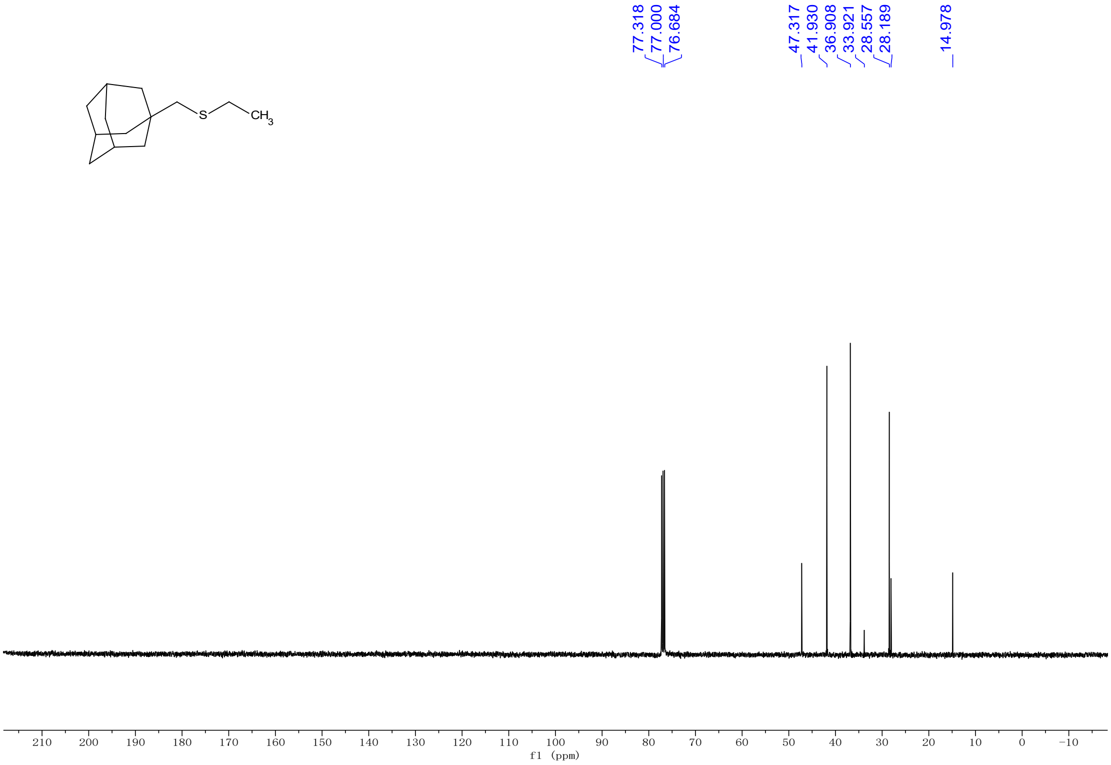

^{1}H NMR (400 MHz, $CDCl_3$) spectrum of compound 3ad

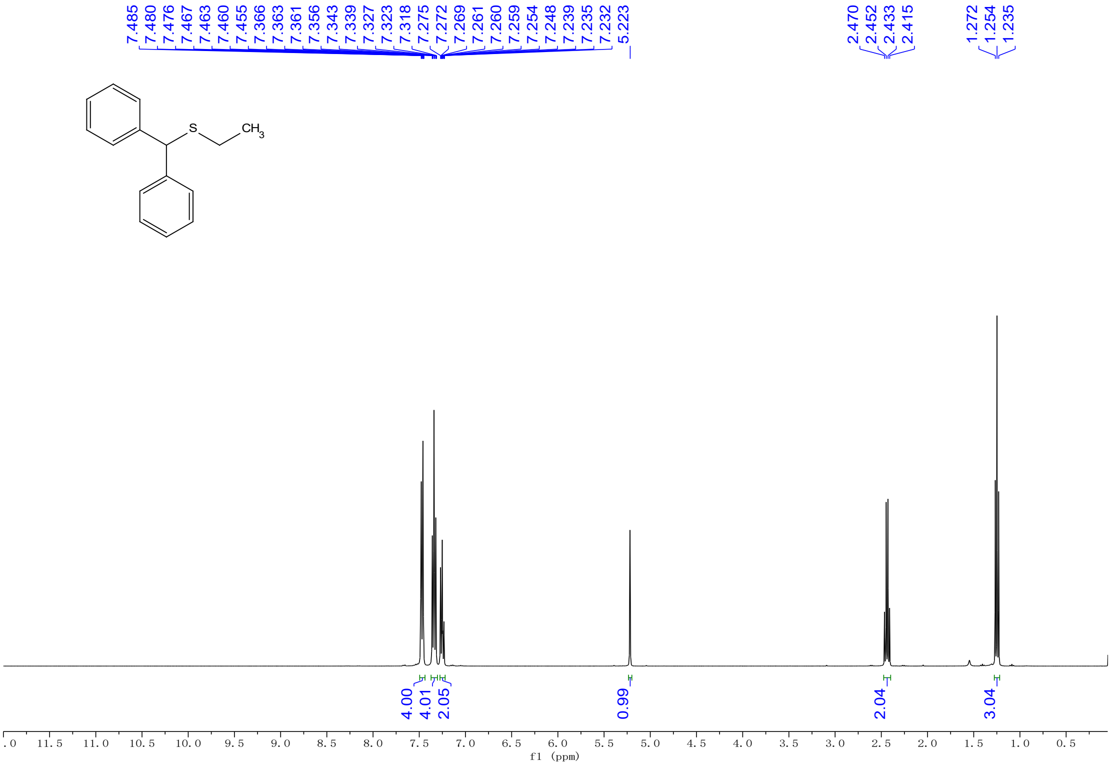

^{13}C NMR (100 MHz, $CDCl_3$) spectrum of compound 3ad

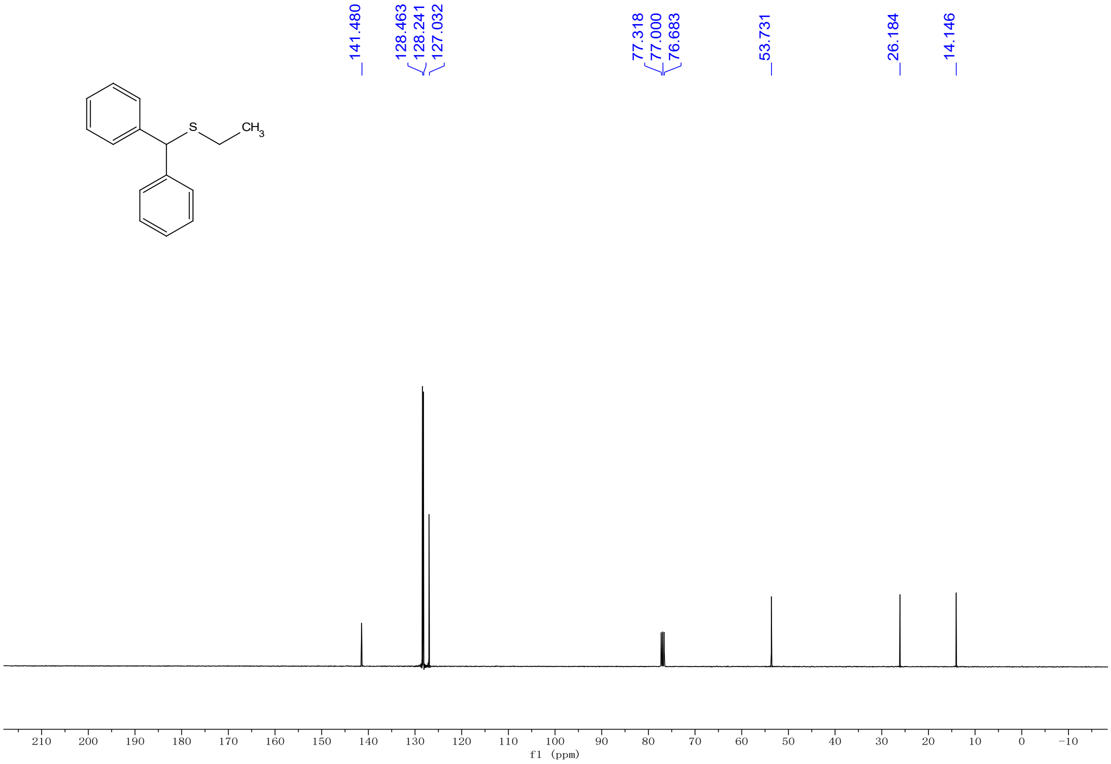

^{1}H NMR (400 MHz, CDCl$_3$) spectrum of compound 3ae

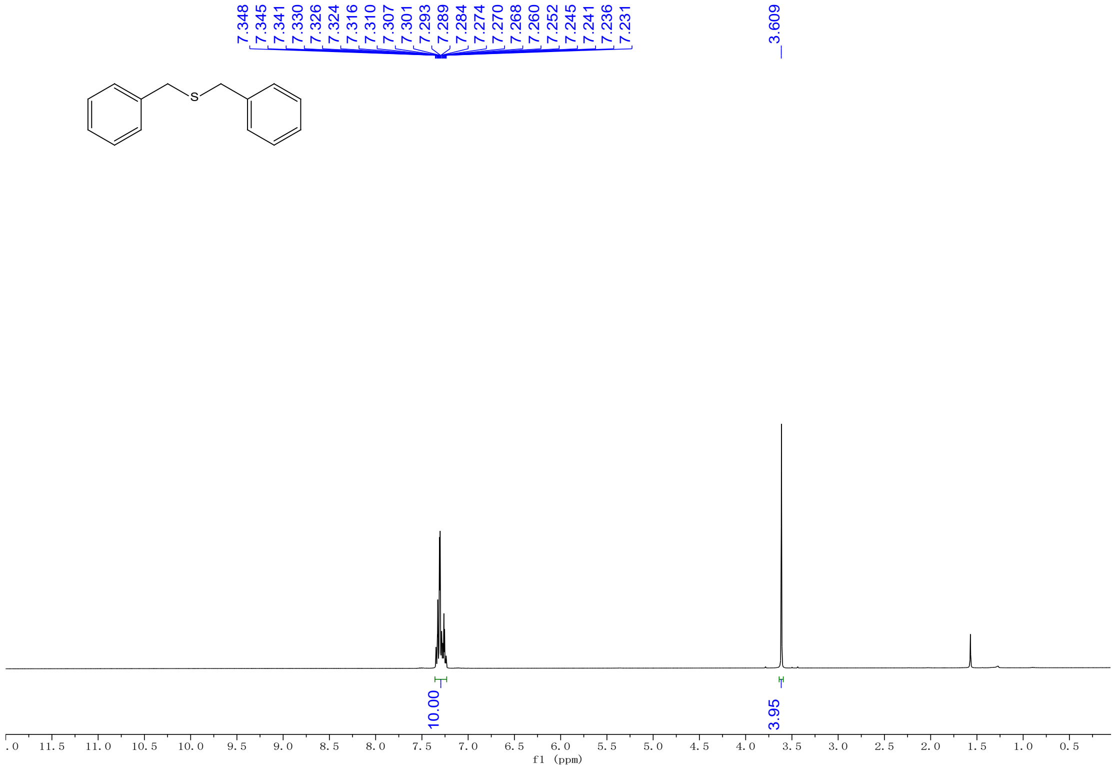

^{13}C NMR (100 MHz, CDCl$_3$) spectrum of compound 3ae

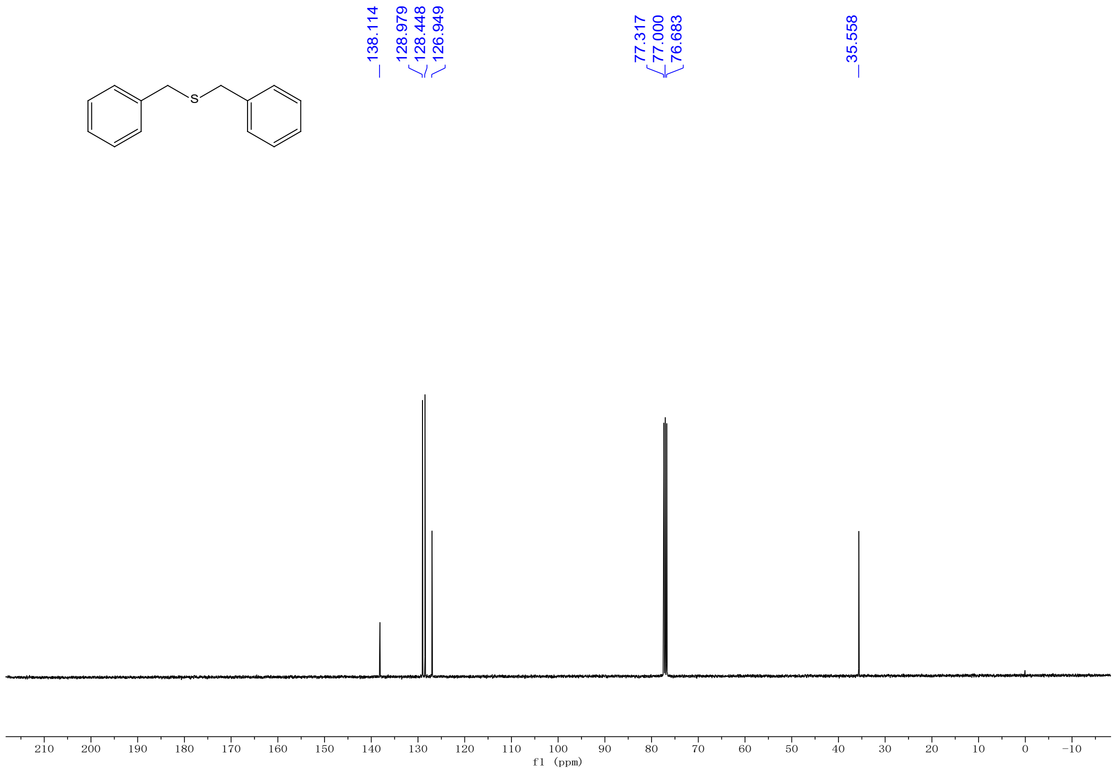

^{1}H NMR (400 MHz, CDCl$_3$) spectrum of compound 3af

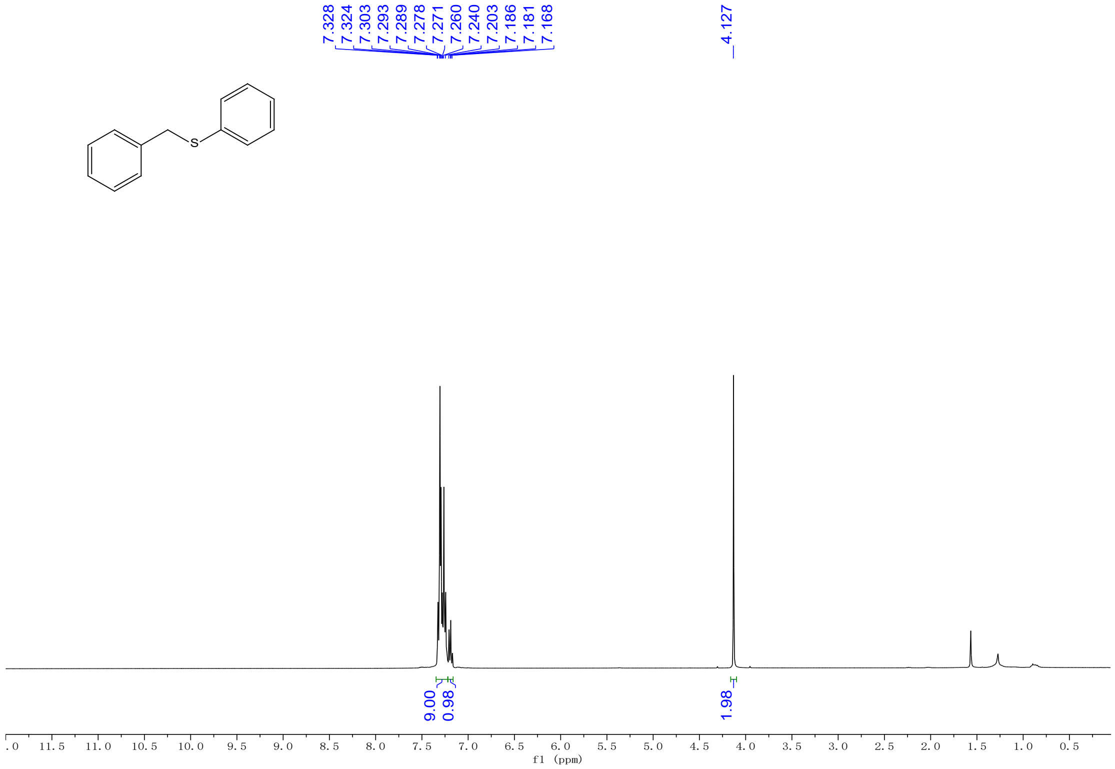

^{13}C NMR (100 MHz, CDCl$_3$) spectrum of compound 3af

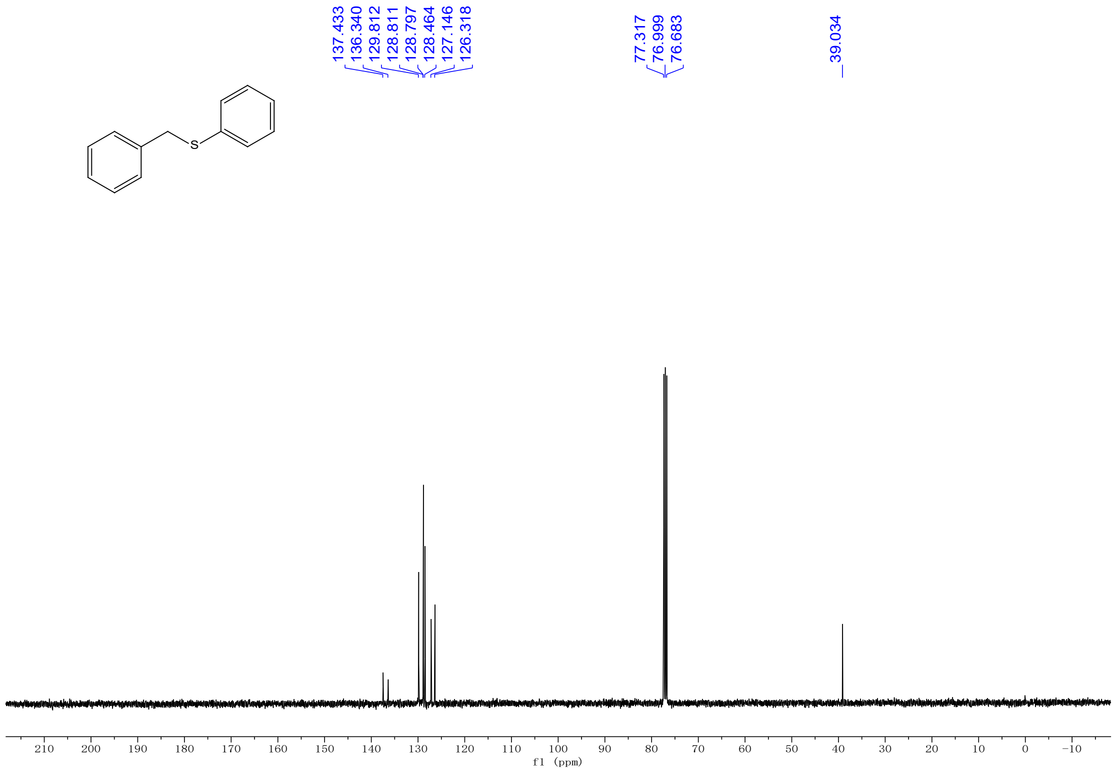

^{1}H NMR (500 MHz, $CDCl_3$) spectrum of compound 3ag

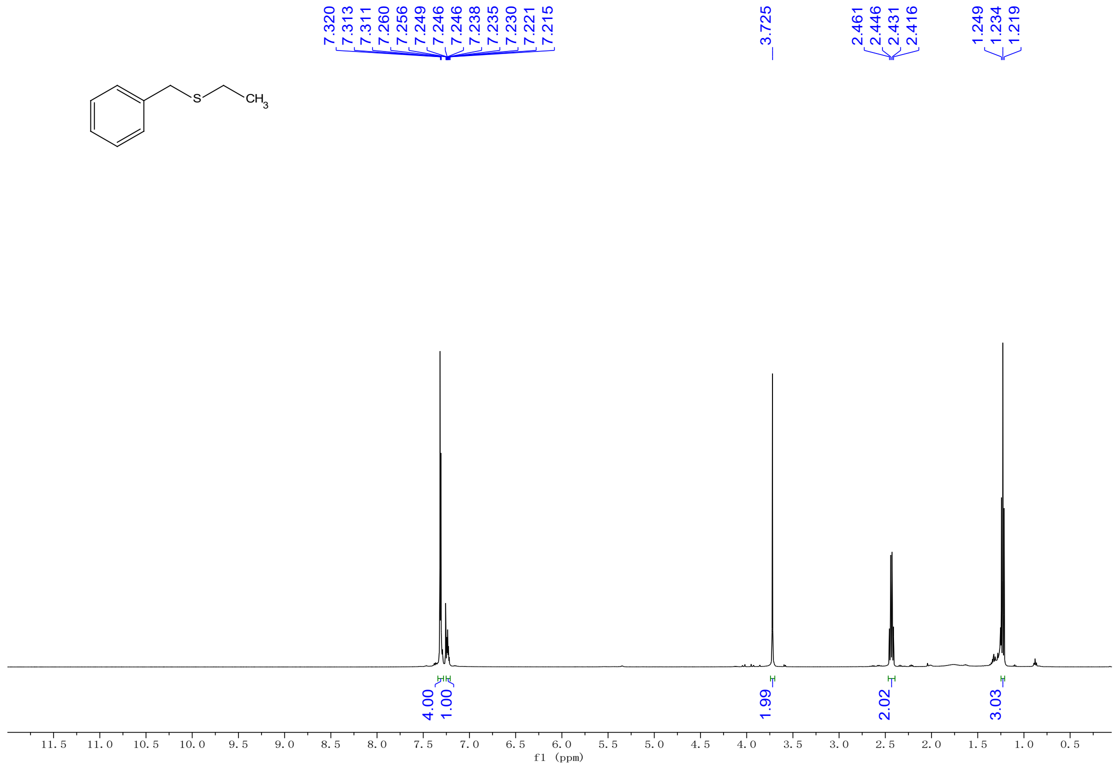

^{13}C NMR (125 MHz, $CDCl_3$) spectrum of compound 3ag

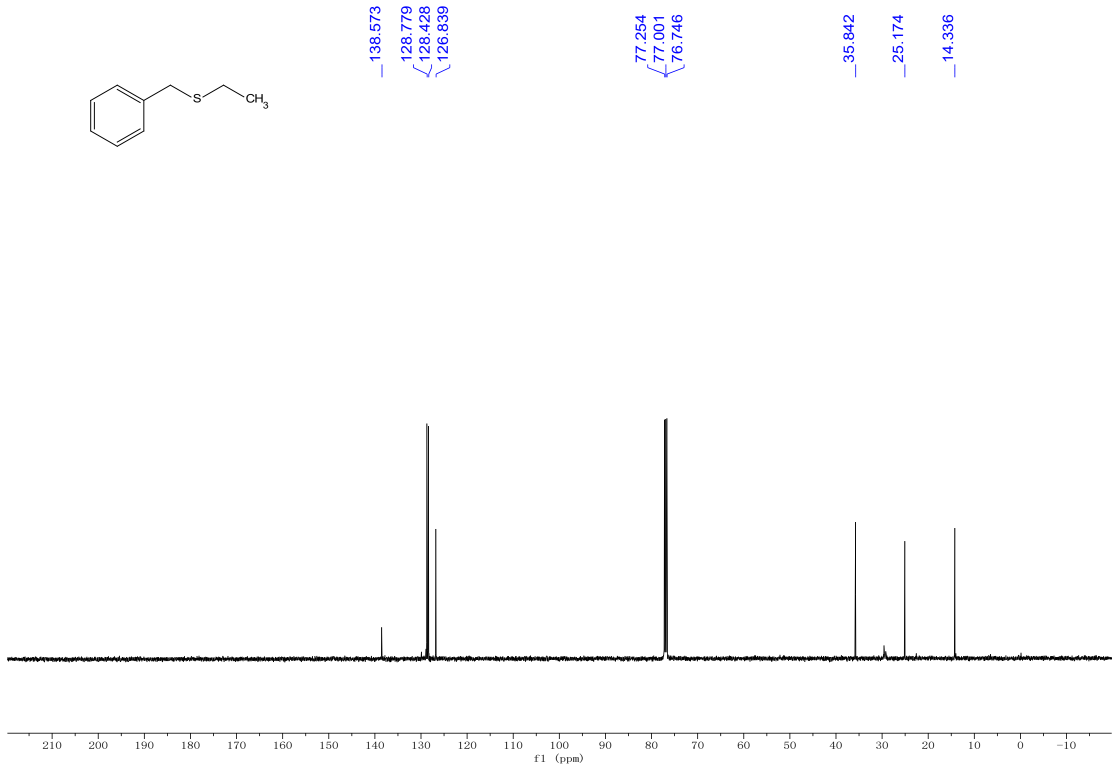

^{1}H NMR (400 MHz, $CDCl_3$) spectrum of compound 3ah

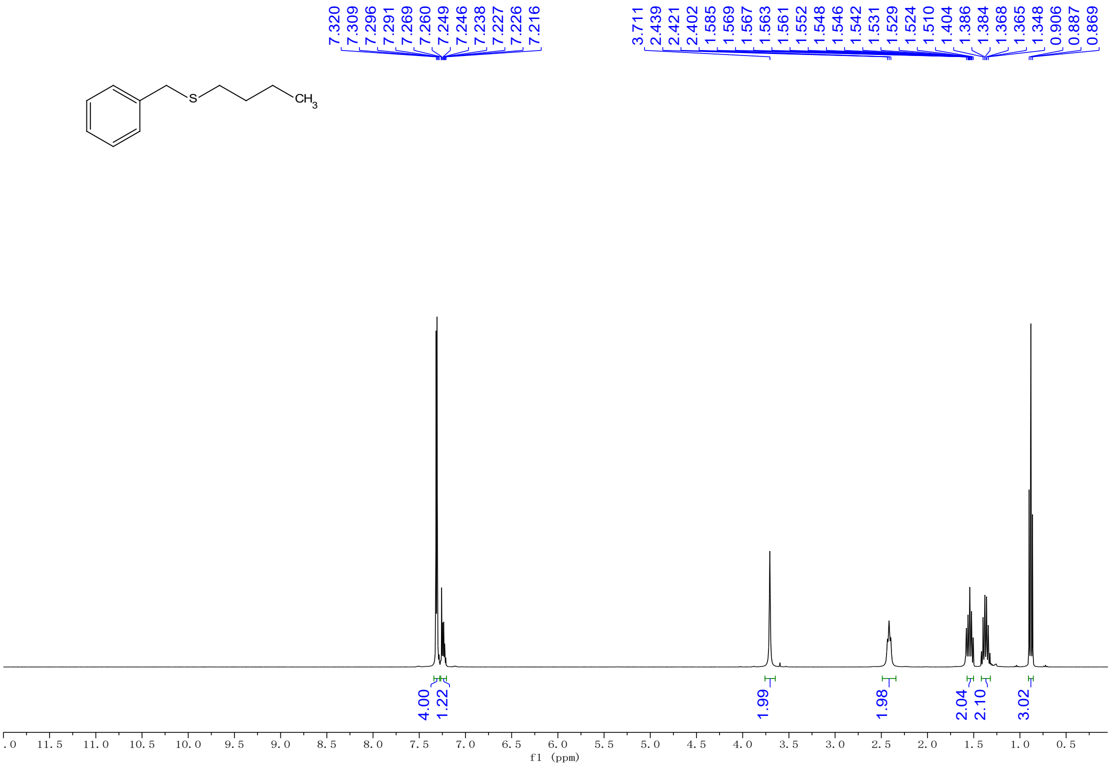

^{13}C NMR (100 MHz, $CDCl_3$) spectrum of compound 3ah

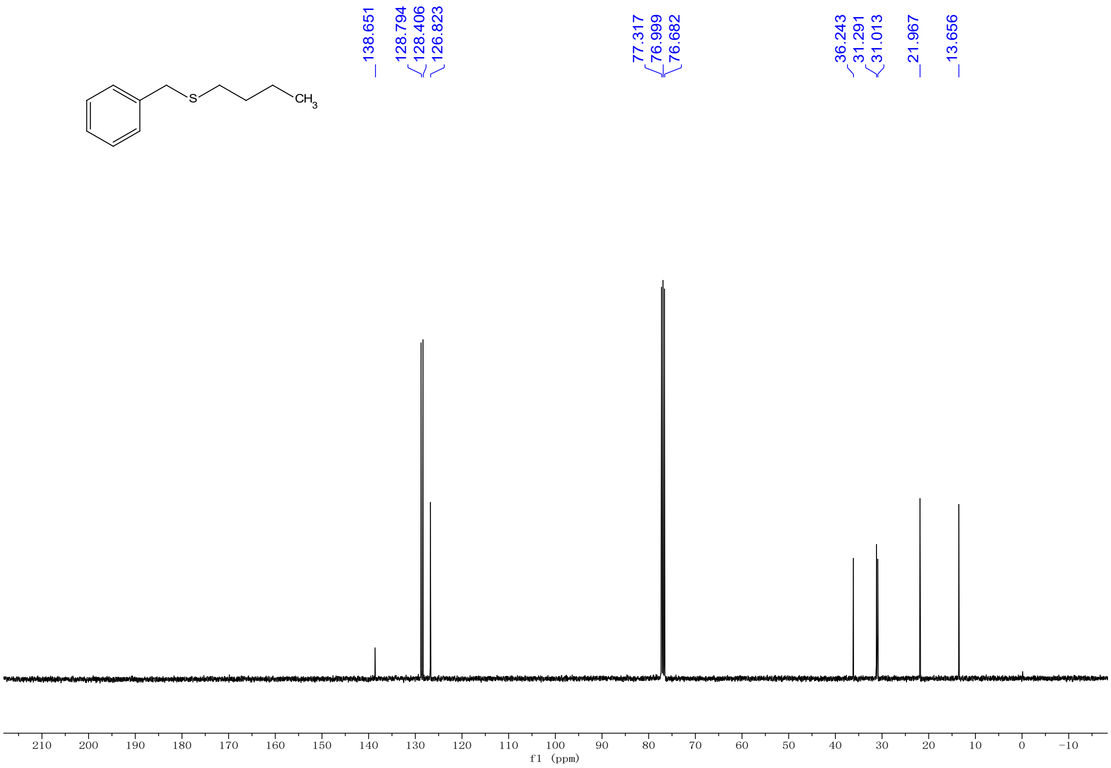

^{1}H NMR (500 MHz, $CDCl_3$) spectrum of compound 4a

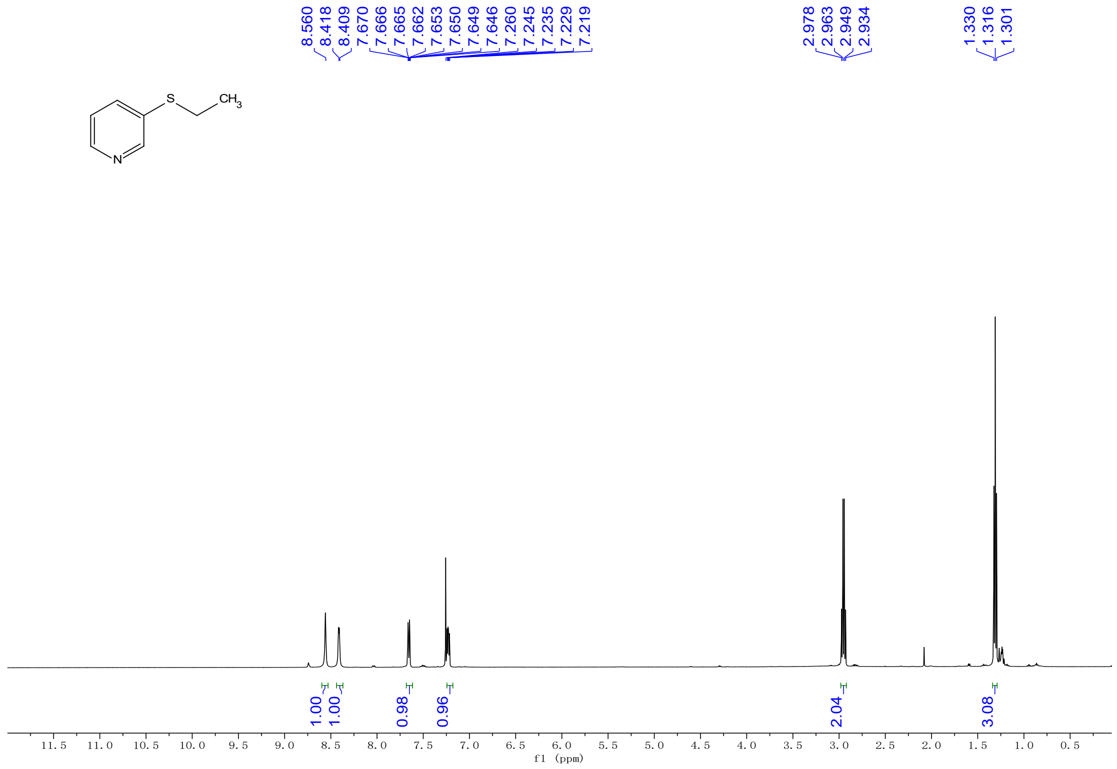

^{13}C NMR (125 MHz, $CDCl_3$) spectrum of compound 4a

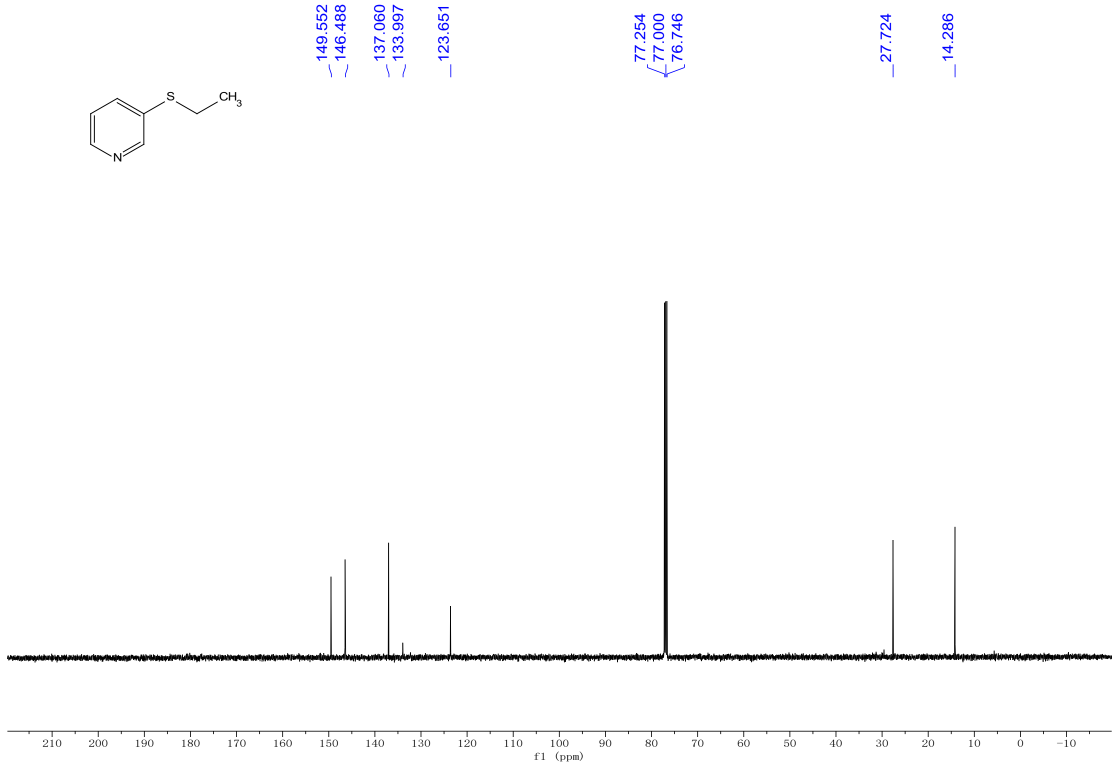

^1H NMR (500 MHz, $CDCl_3$) spectrum of compound 4b

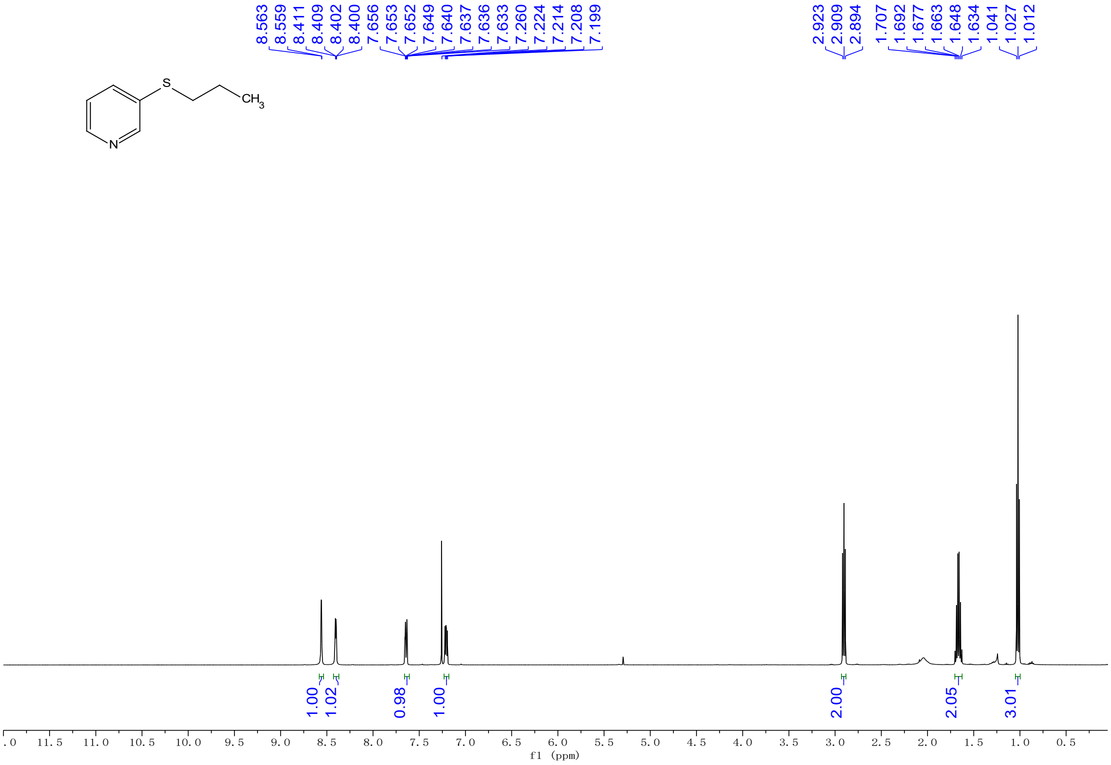

^{13}C NMR (125 MHz, $CDCl_3$) spectrum of compound 4b

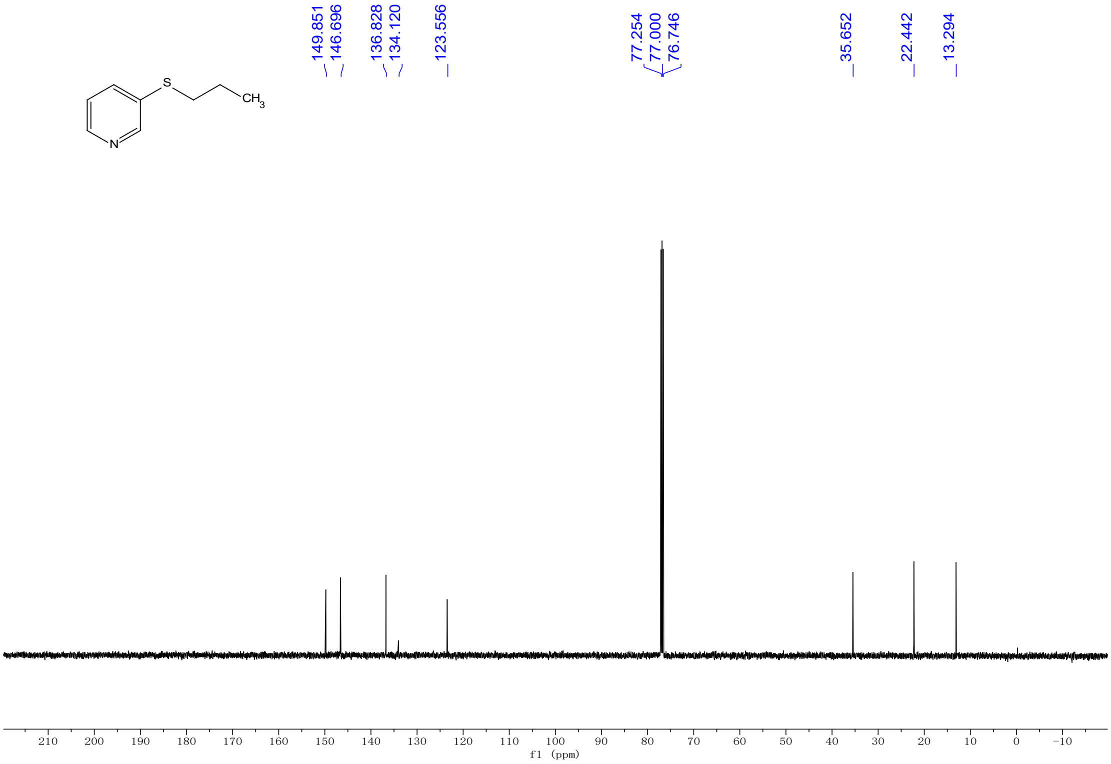

^{1}H NMR (500 MHz, $CDCl_3$) spectrum of compound 4c

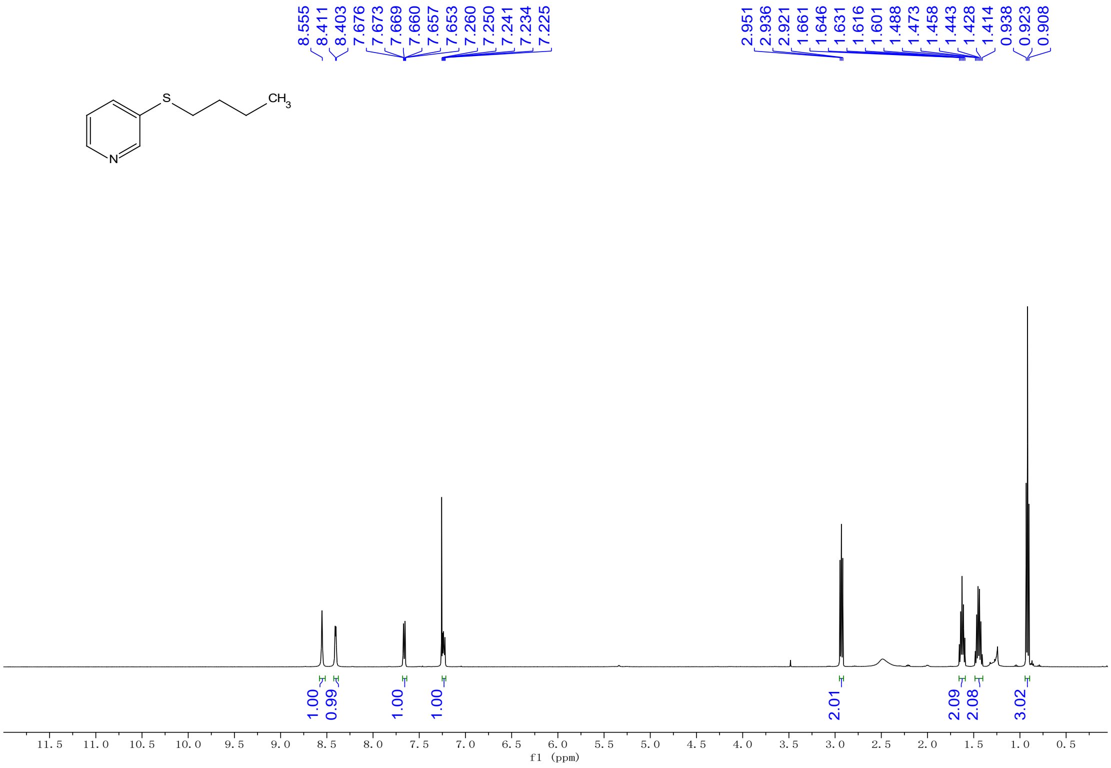

^{13}C NMR (125 MHz, $CDCl_3$) spectrum of compound 4c

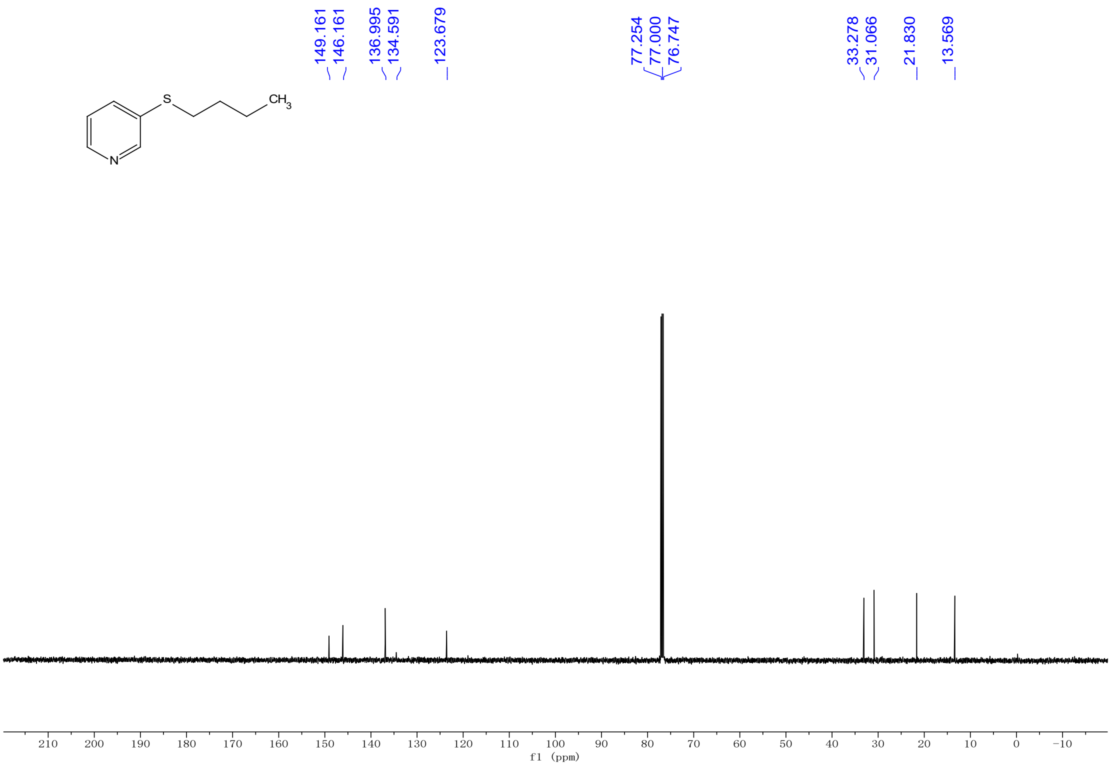

^{1}H NMR (500 MHz, CDCl$_3$) spectrum of compound 4d

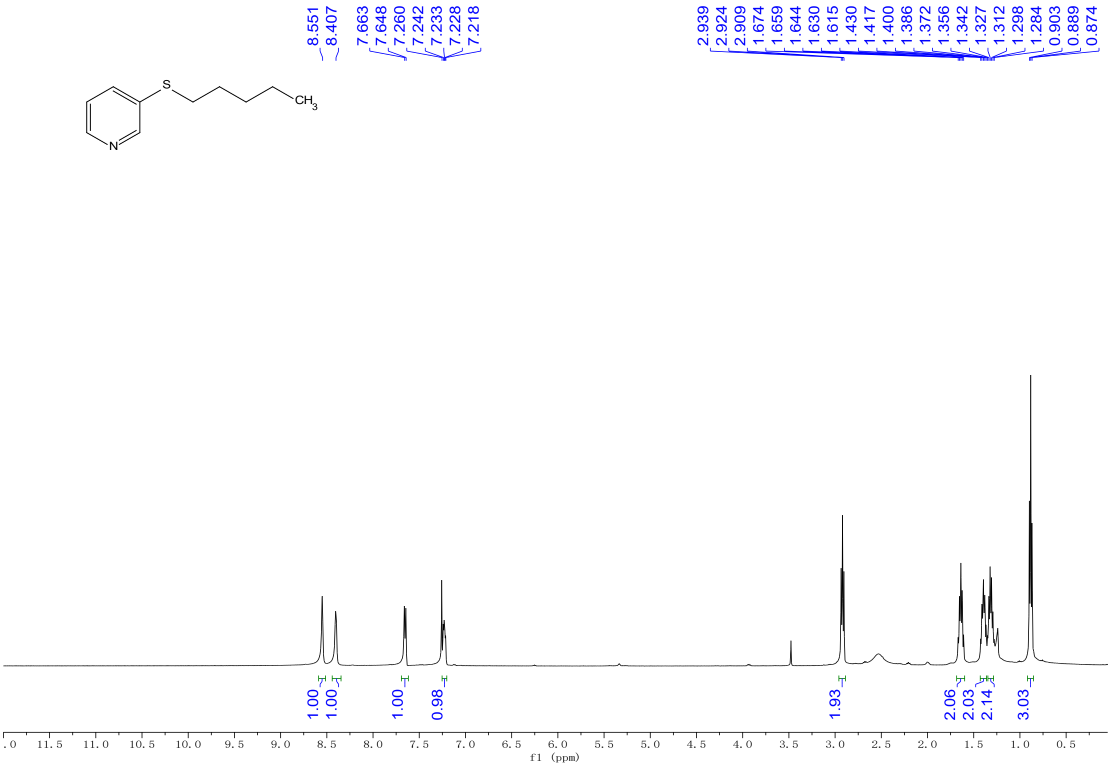

^{13}C NMR (125 MHz, CDCl$_3$) spectrum of compound 4d

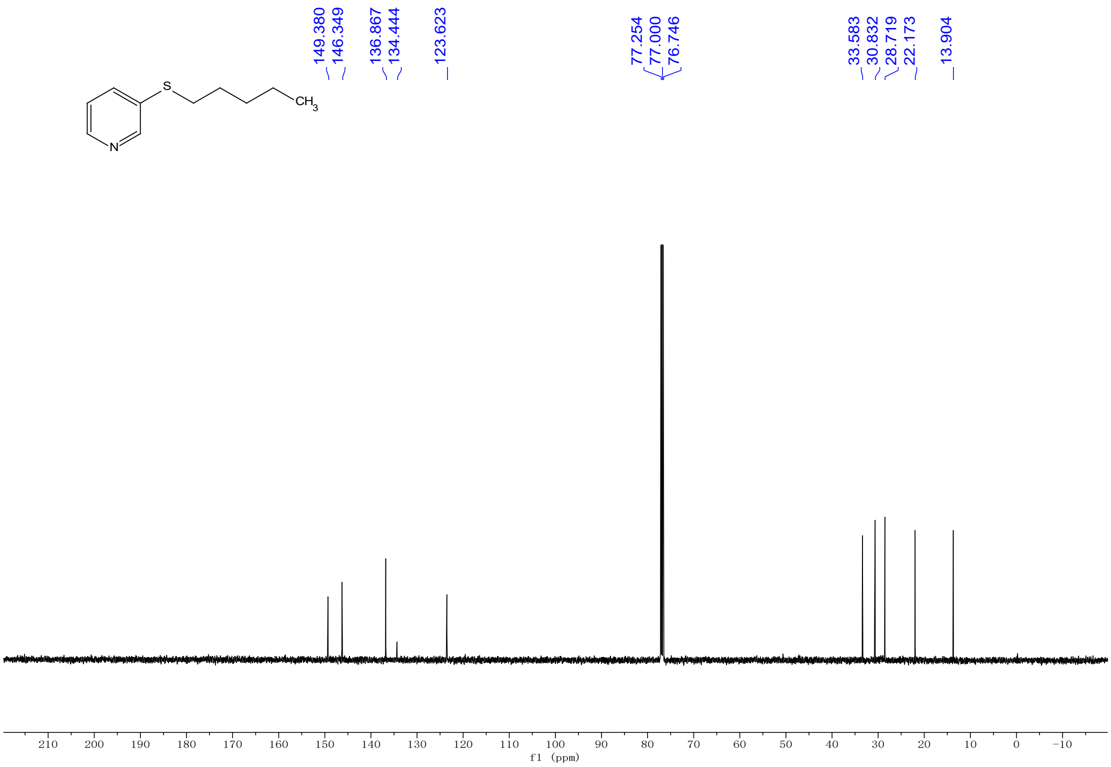

^1H NMR (500 MHz, $CDCl_3$) spectrum of compound 4e

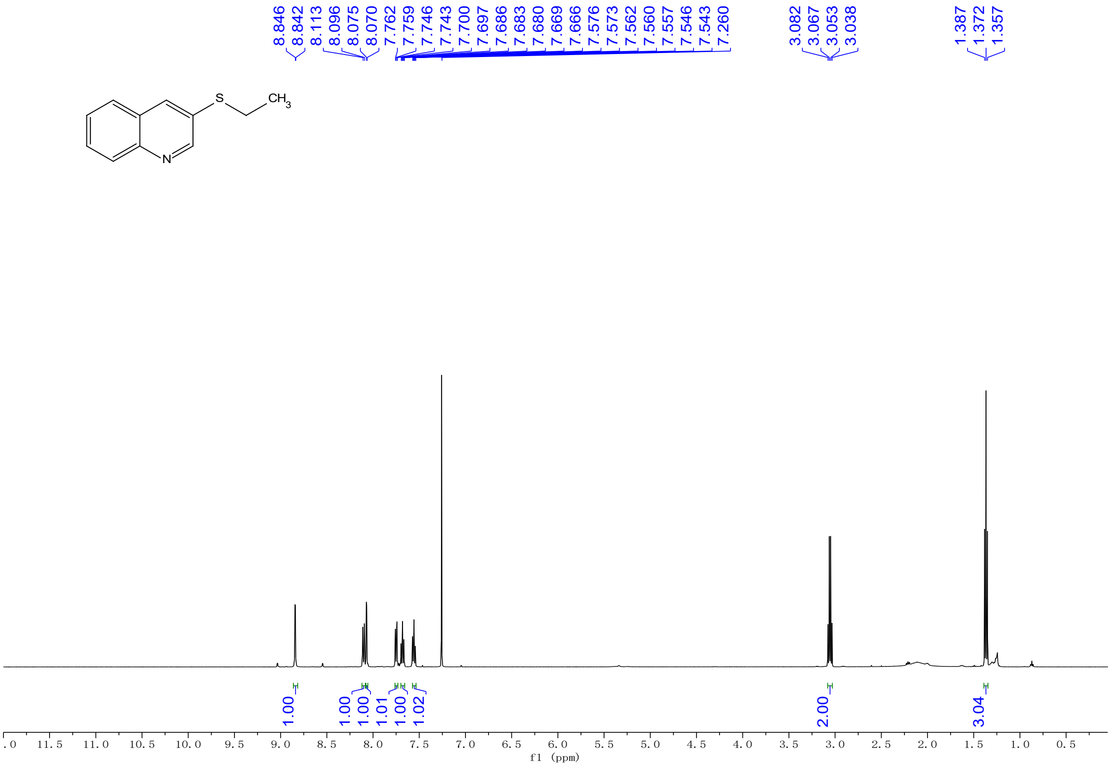

^{13}C NMR (125 MHz, $CDCl_3$) spectrum of compound 4e

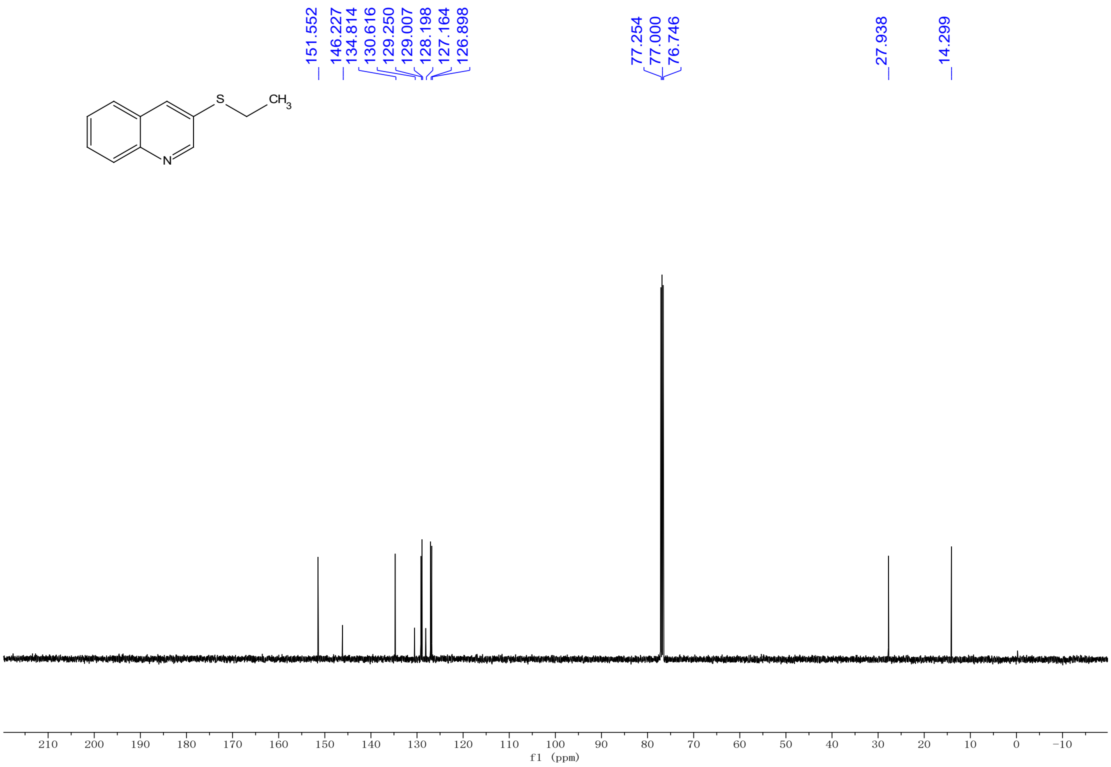

^{1}H NMR (500 MHz, $CDCl_3$) spectrum of compound 4f

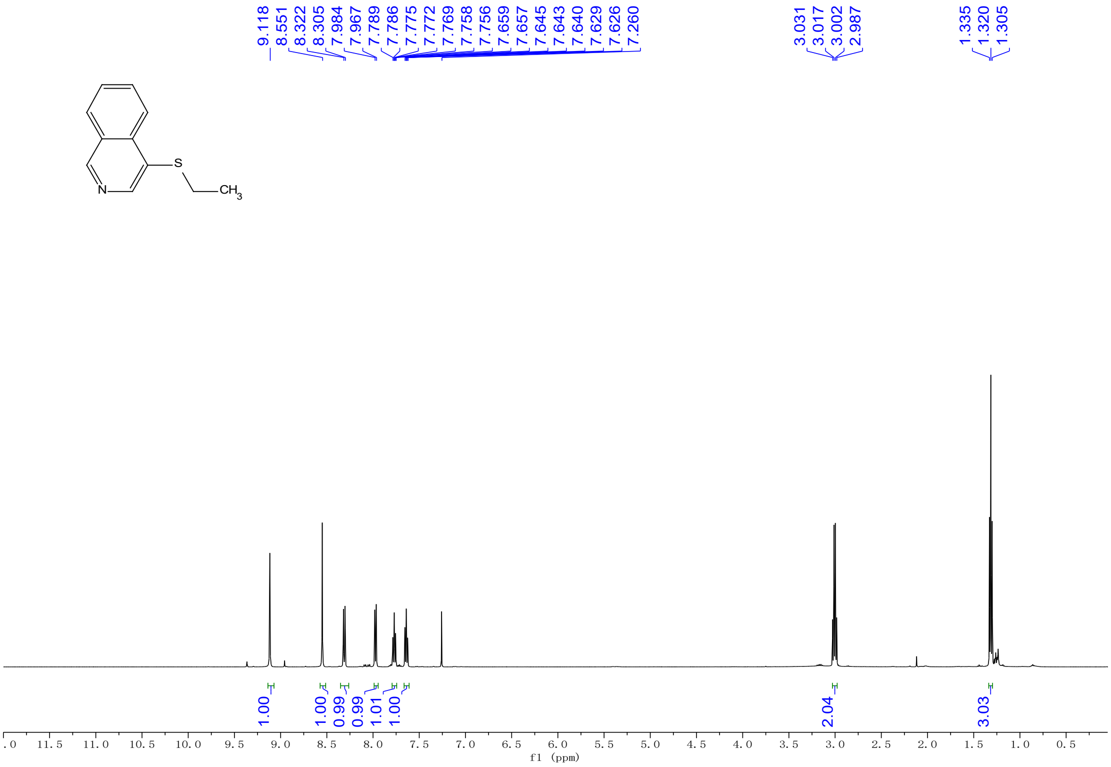

^{13}C NMR (125 MHz, $CDCl_3$) spectrum of compound 4f

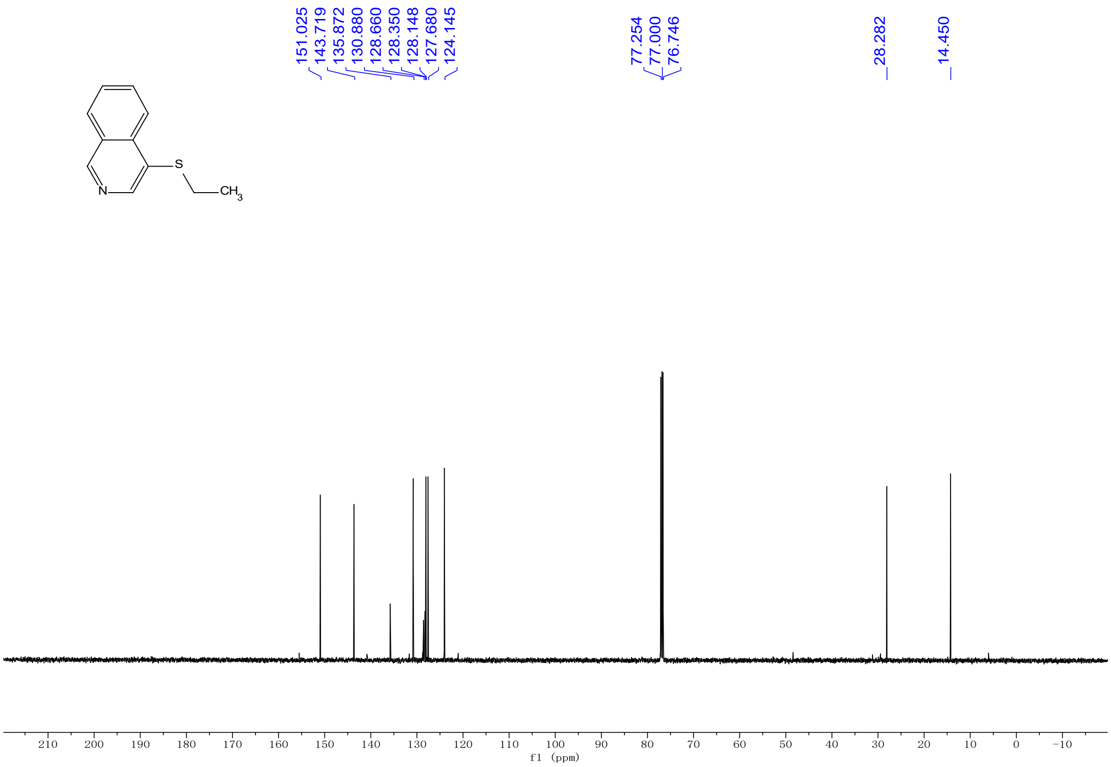

^{1}H NMR (500 MHz, $CDCl_3$) spectrum of compound 4g

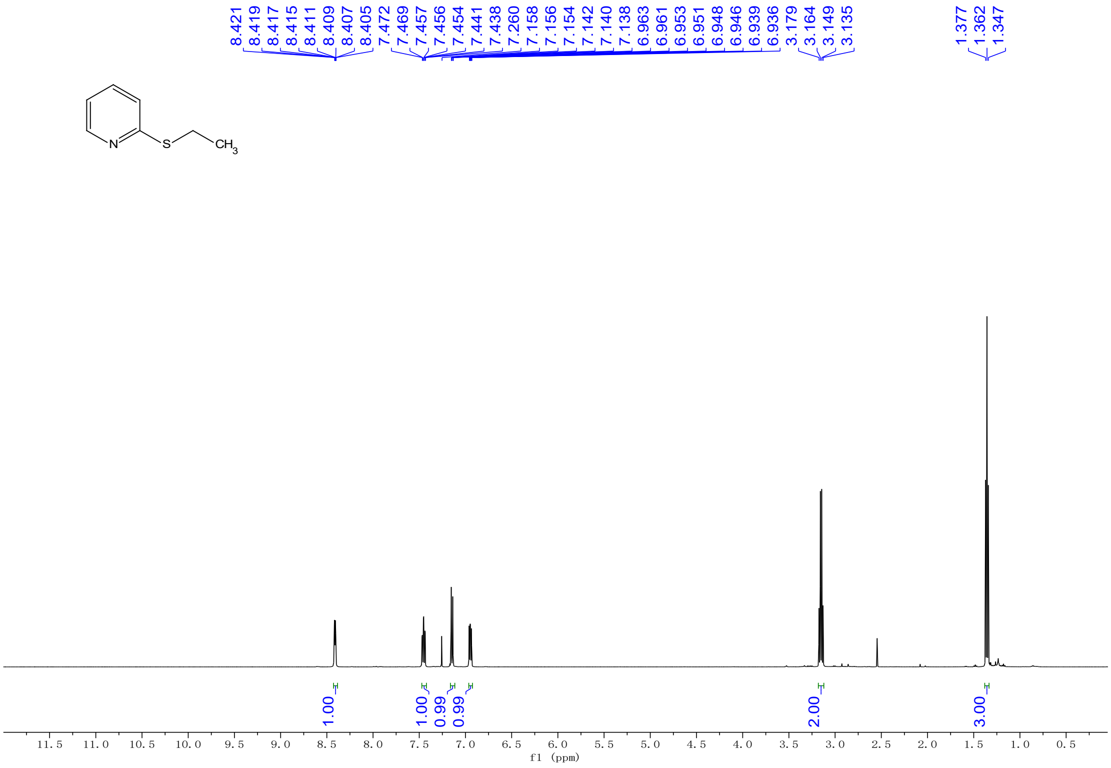

^{13}C NMR (125 MHz, $CDCl_3$) spectrum of compound 4g

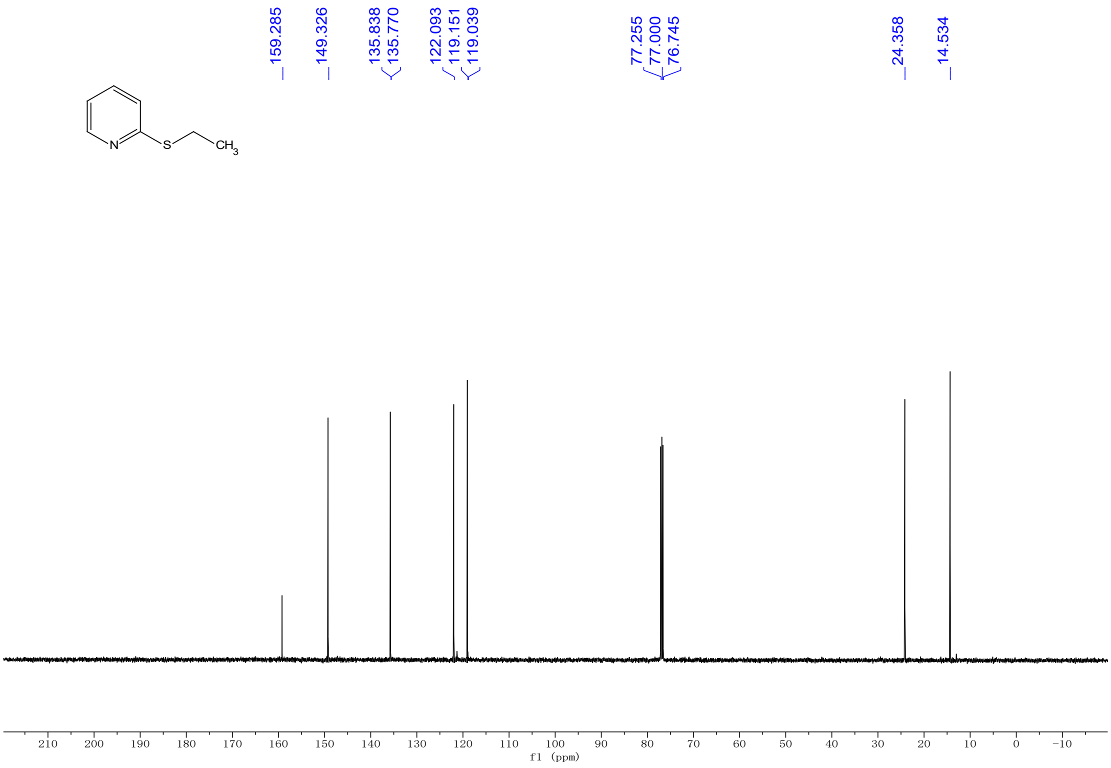

^{1}H NMR (500 MHz, CDCl$_3$) spectrum of compound 4h

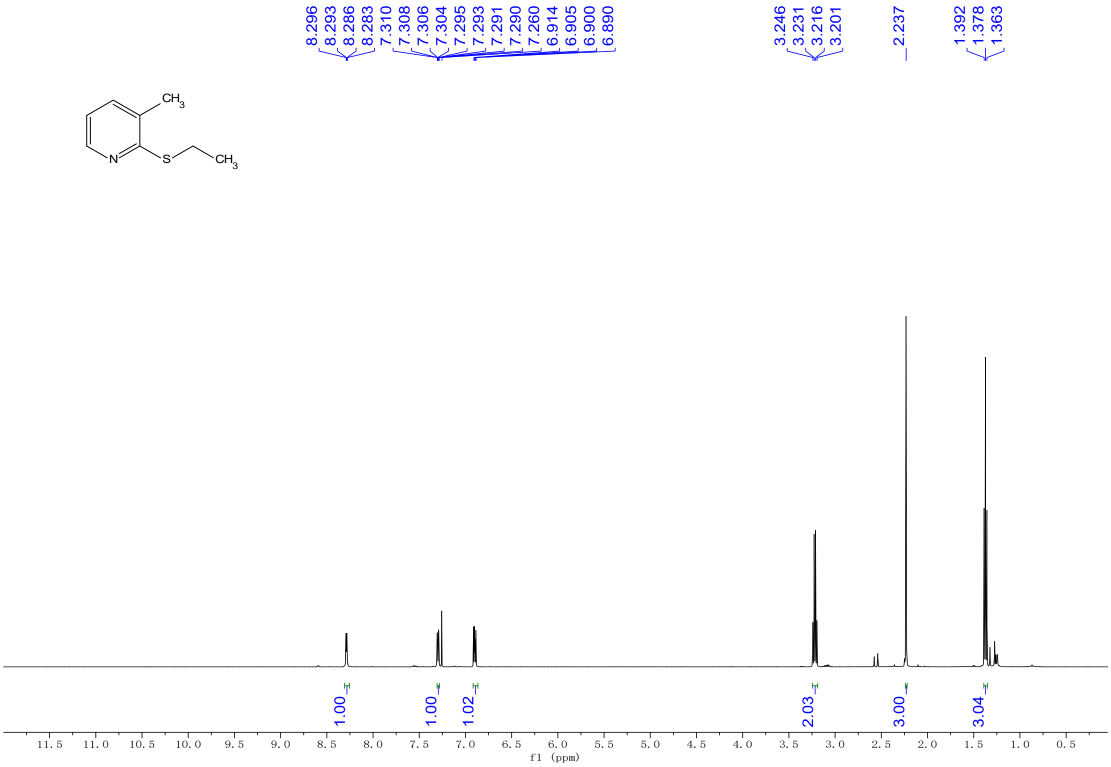

^{13}C NMR (125 MHz, CDCl$_3$) spectrum of compound 4h

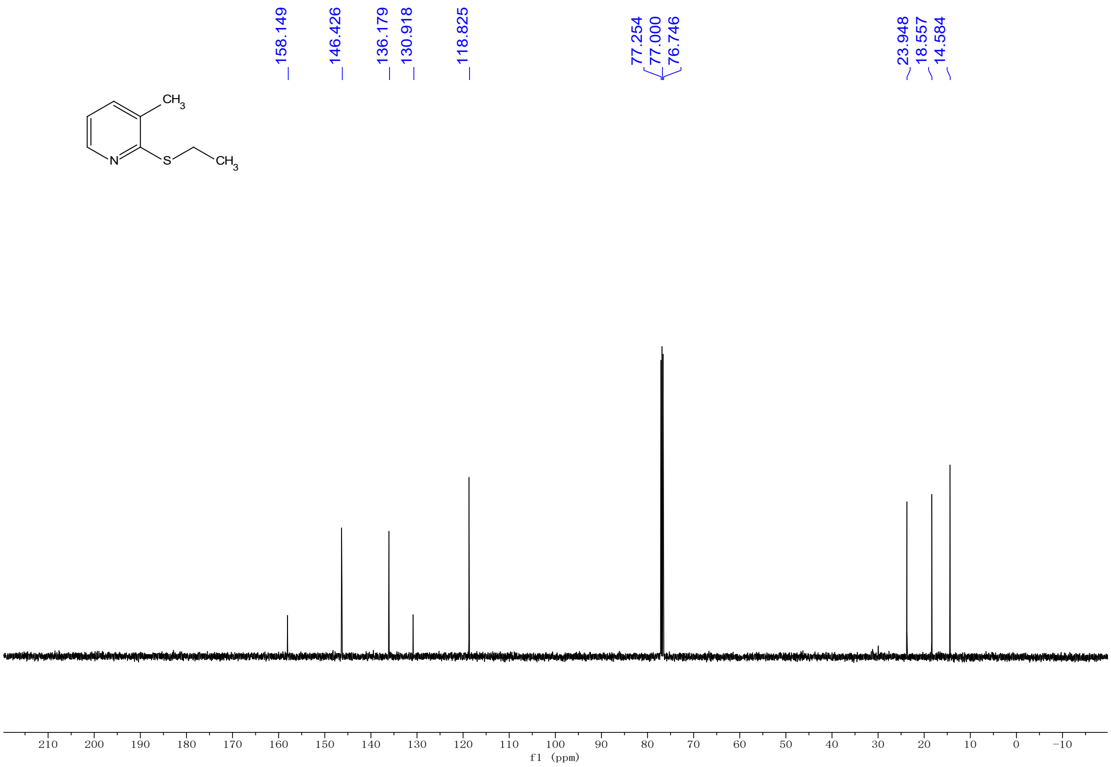

^{1}H NMR (500 MHz, $CDCl_3$) spectrum of compound 4i

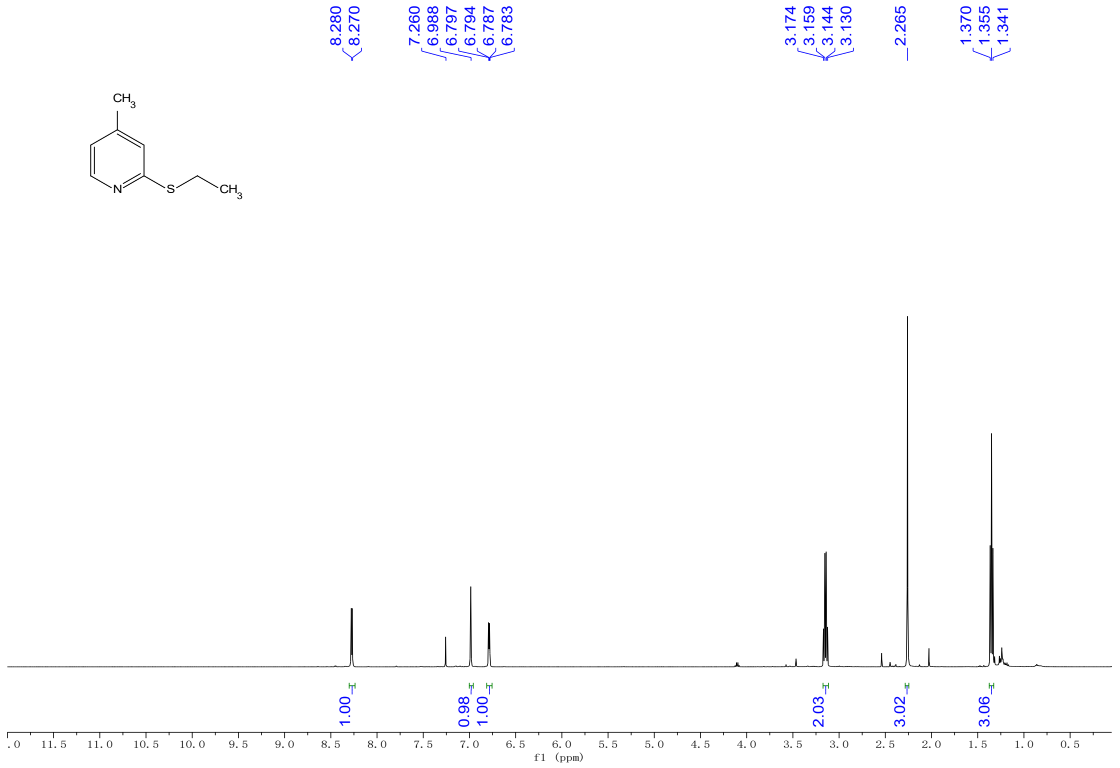

^{13}C NMR (125 MHz, $CDCl_3$) spectrum of compound 4i

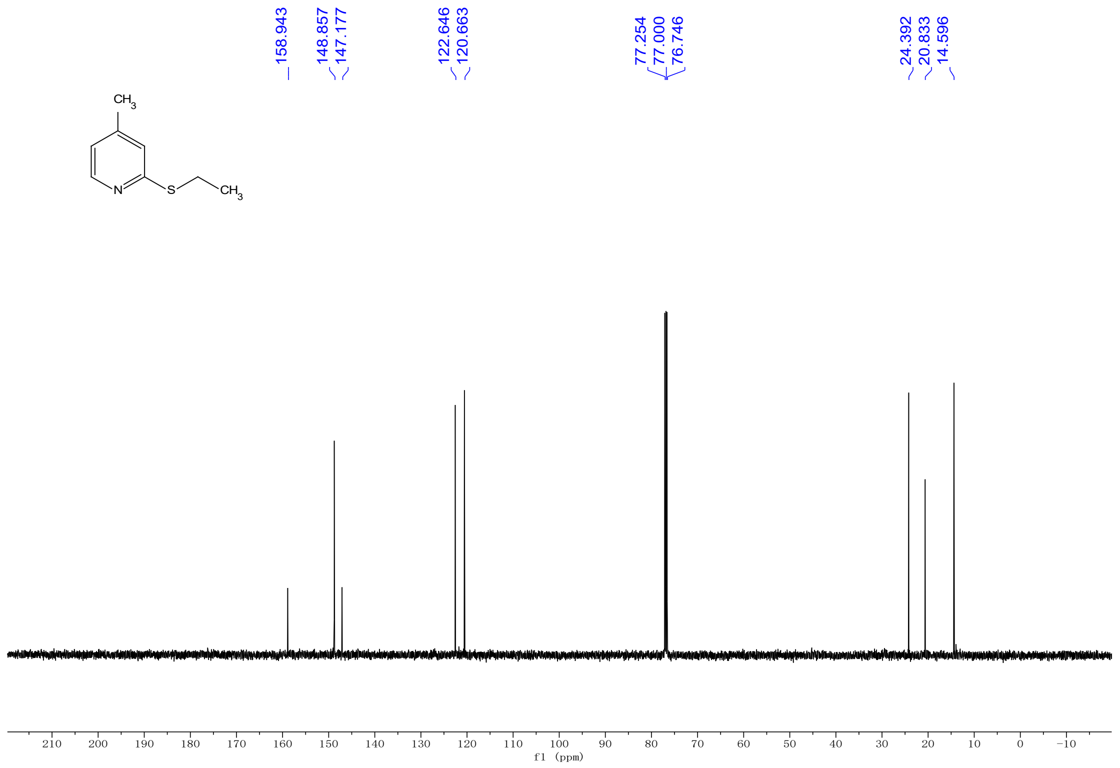

^{1}H NMR (500 MHz, $CDCl_3$) spectrum of compound 4j

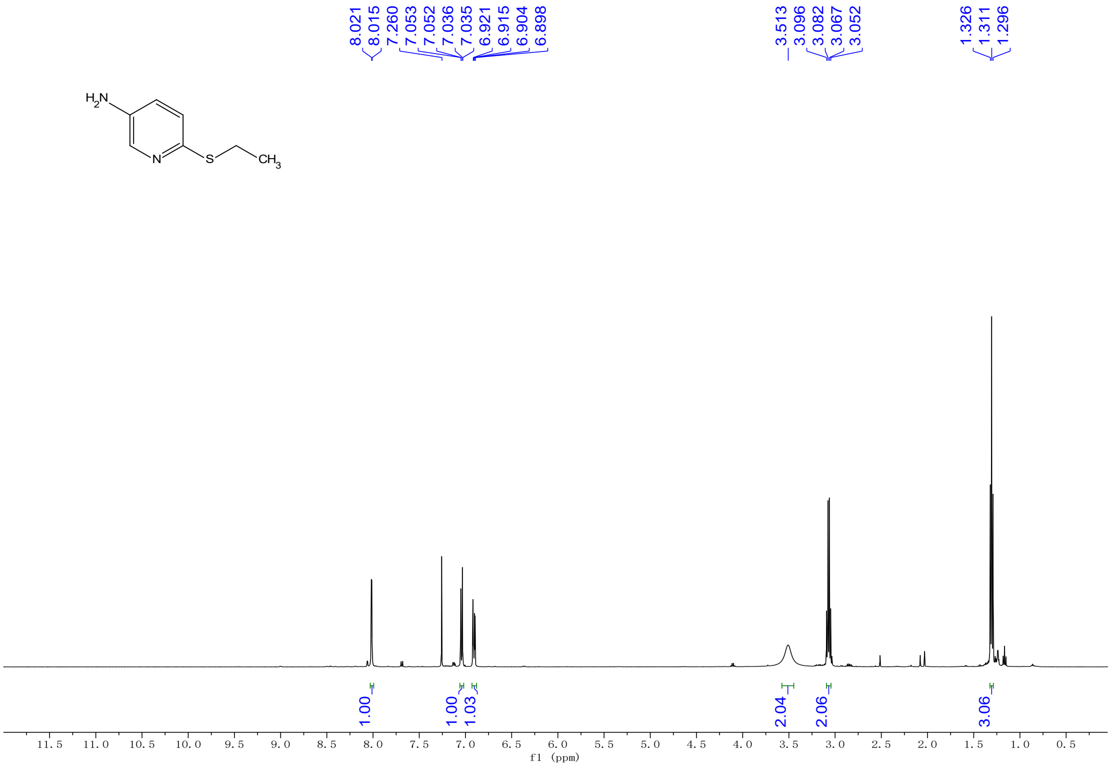

^{13}C NMR (125 MHz, $CDCl_3$) spectrum of compound 4j

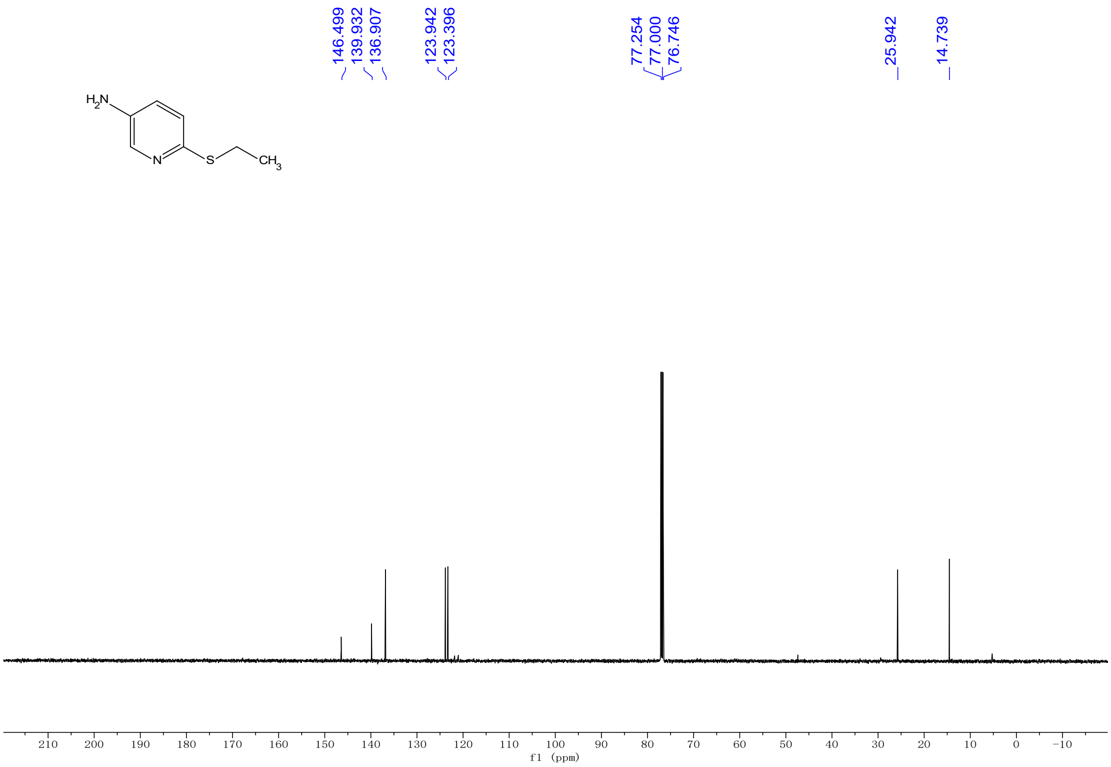

^{1}H NMR (500 MHz, $CDCl_3$) spectrum of compound 4k

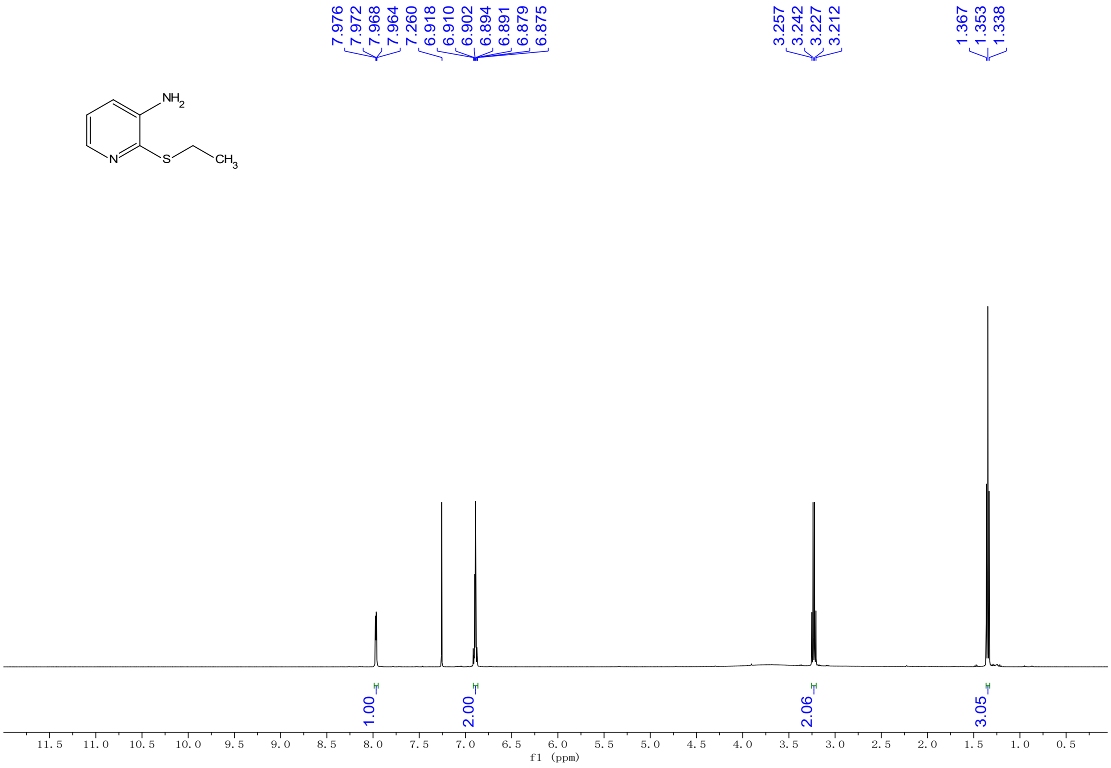

^{13}C NMR (125 MHz, $CDCl_3$) spectrum of compound 4k

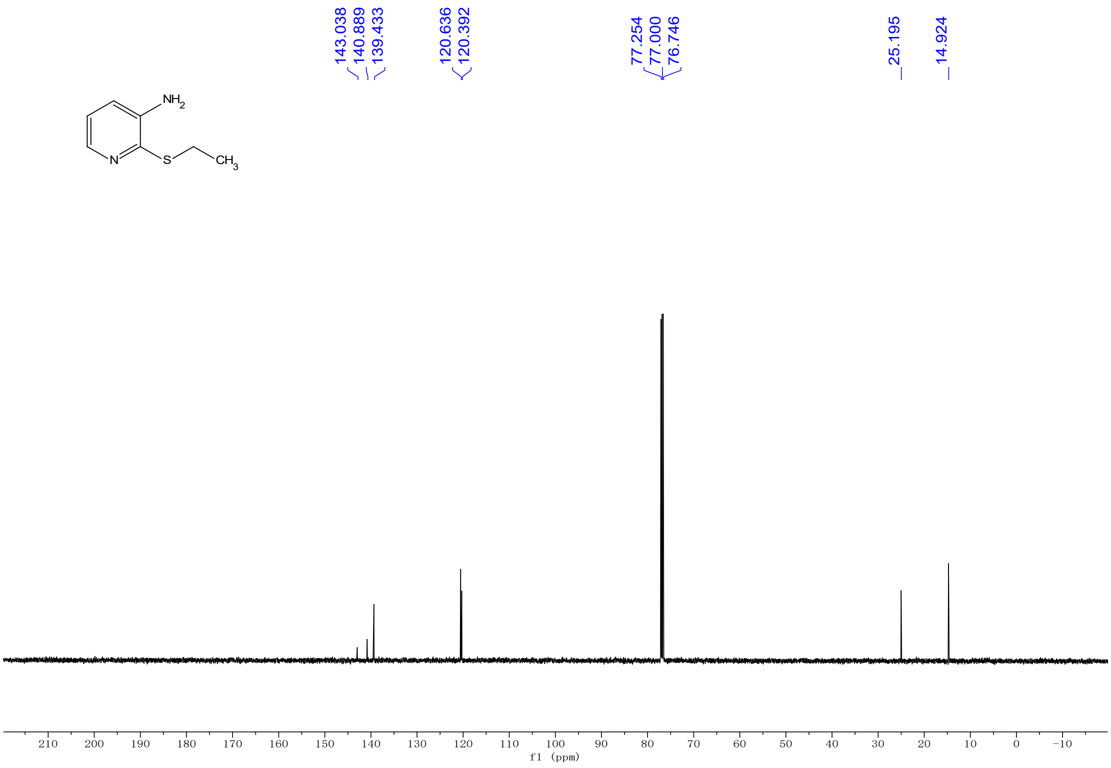

^{1}H NMR (500 MHz, $CDCl_3$) spectrum of compound 4l

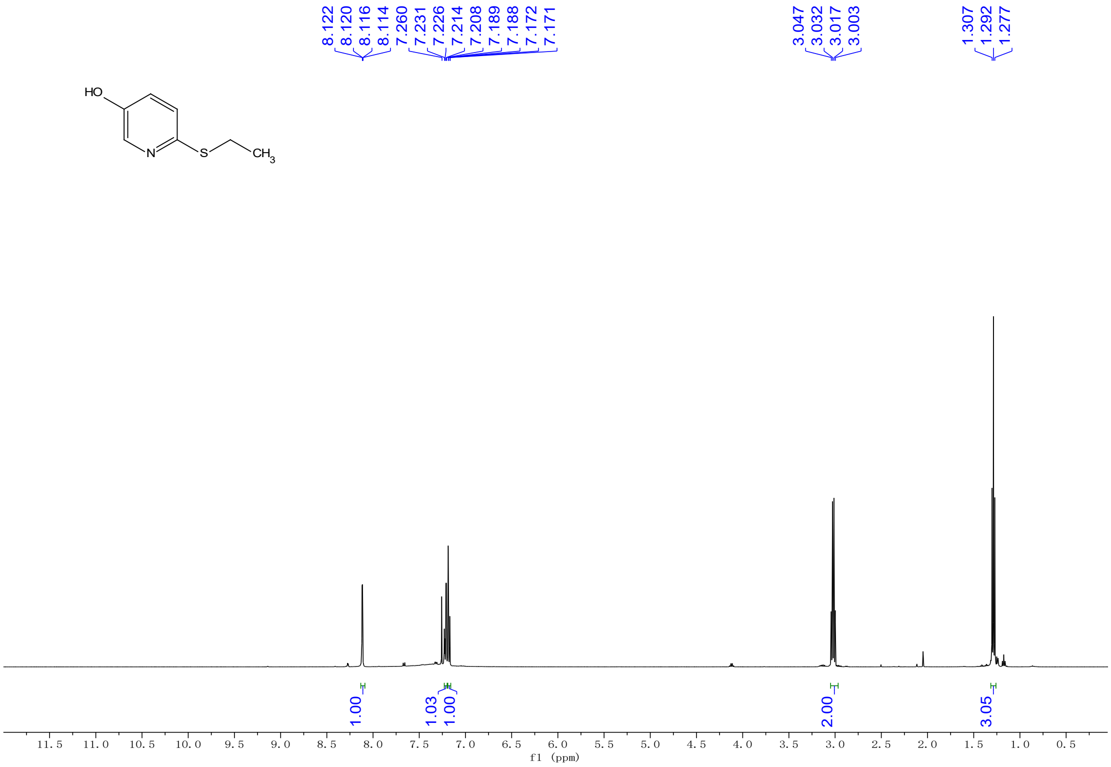

^{13}C NMR (125 MHz, $CDCl_3$) spectrum of compound 4l

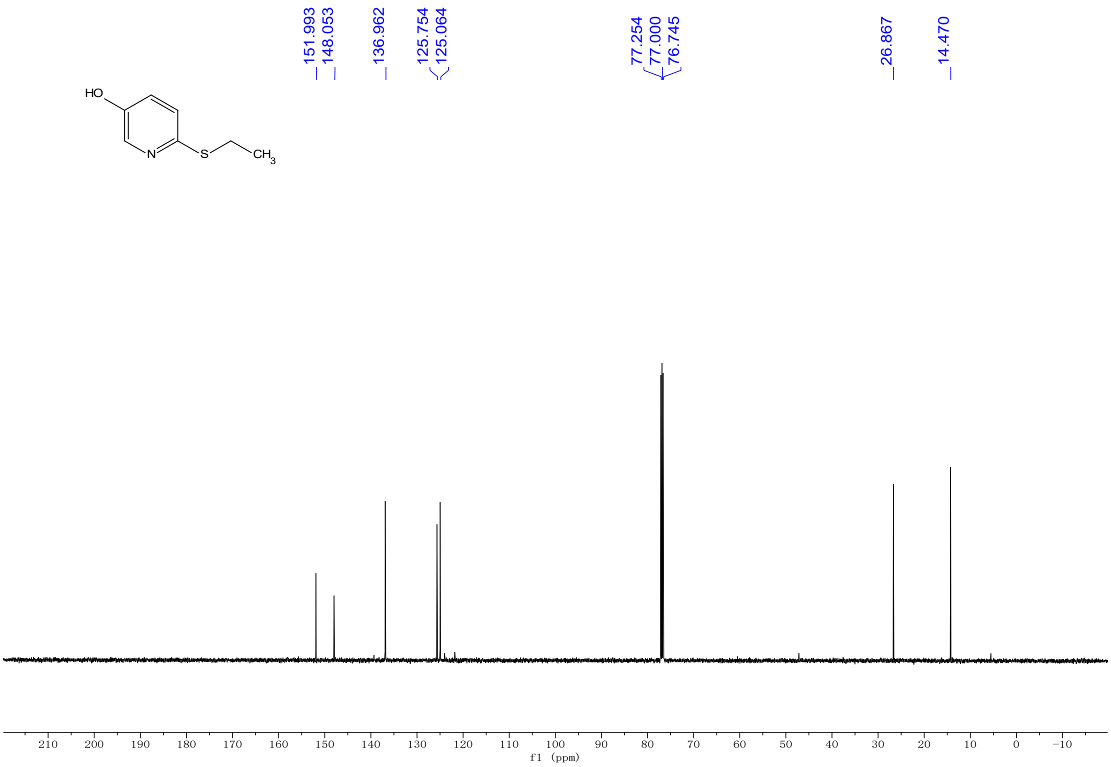

^{1}H NMR (500 MHz, $CDCl_3$) spectrum of compound 4m

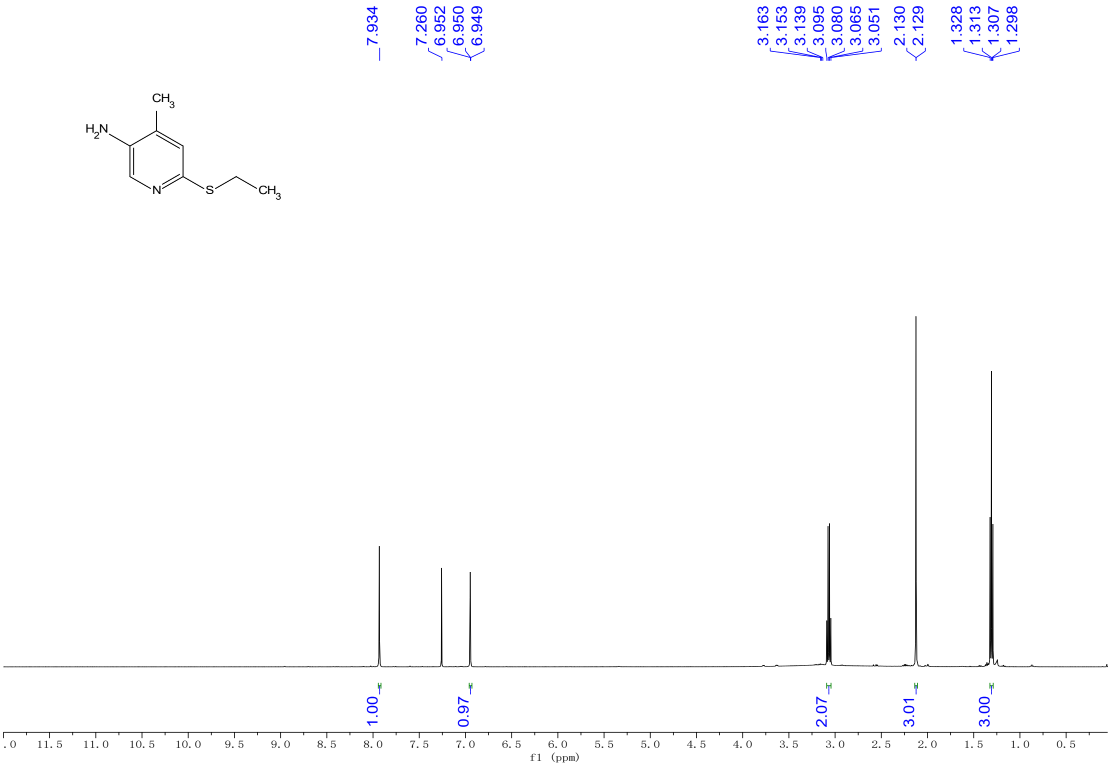

^{13}C NMR (125 MHz, $CDCl_3$) spectrum of compound 4m

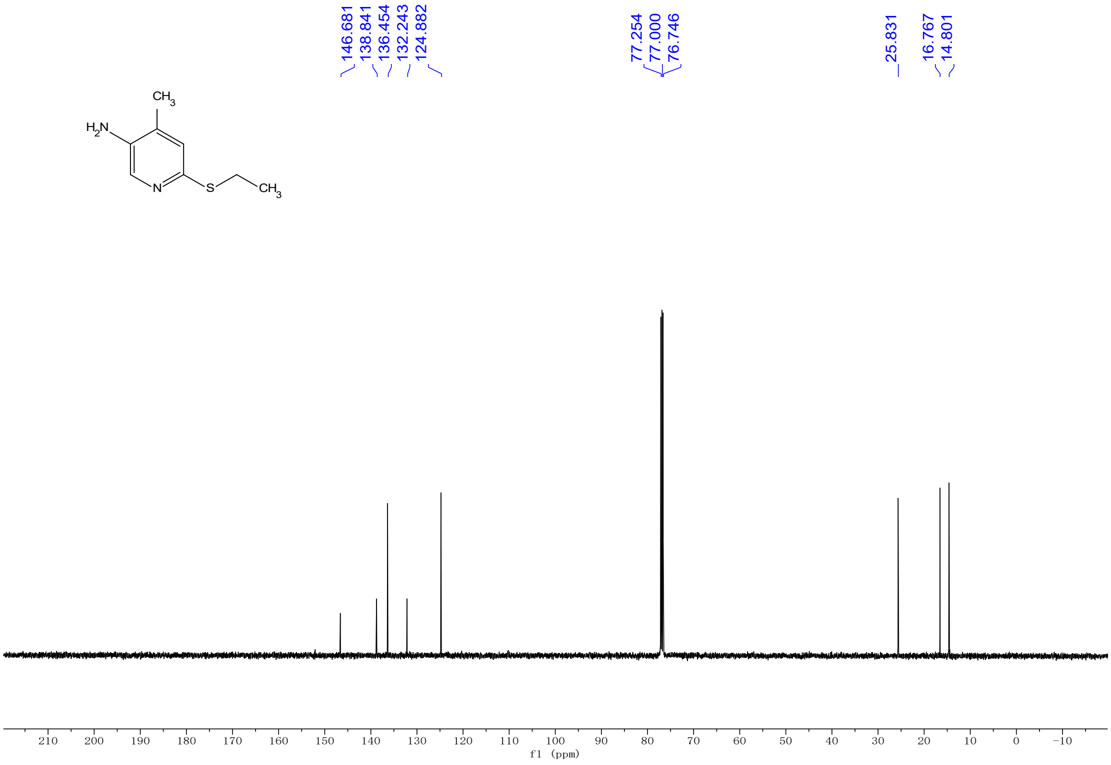

^{1}H NMR (500 MHz, $CDCl_3$) spectrum of compound 4n

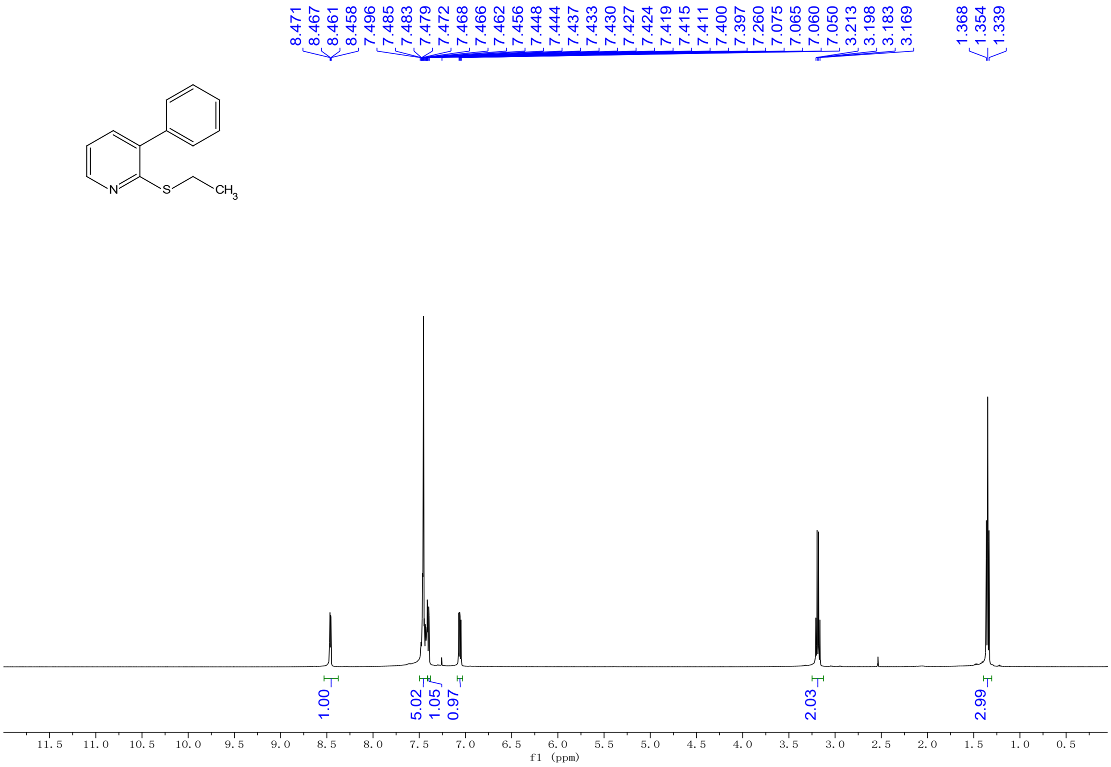

^{13}C NMR (125 MHz, $CDCl_3$) spectrum of compound 4n

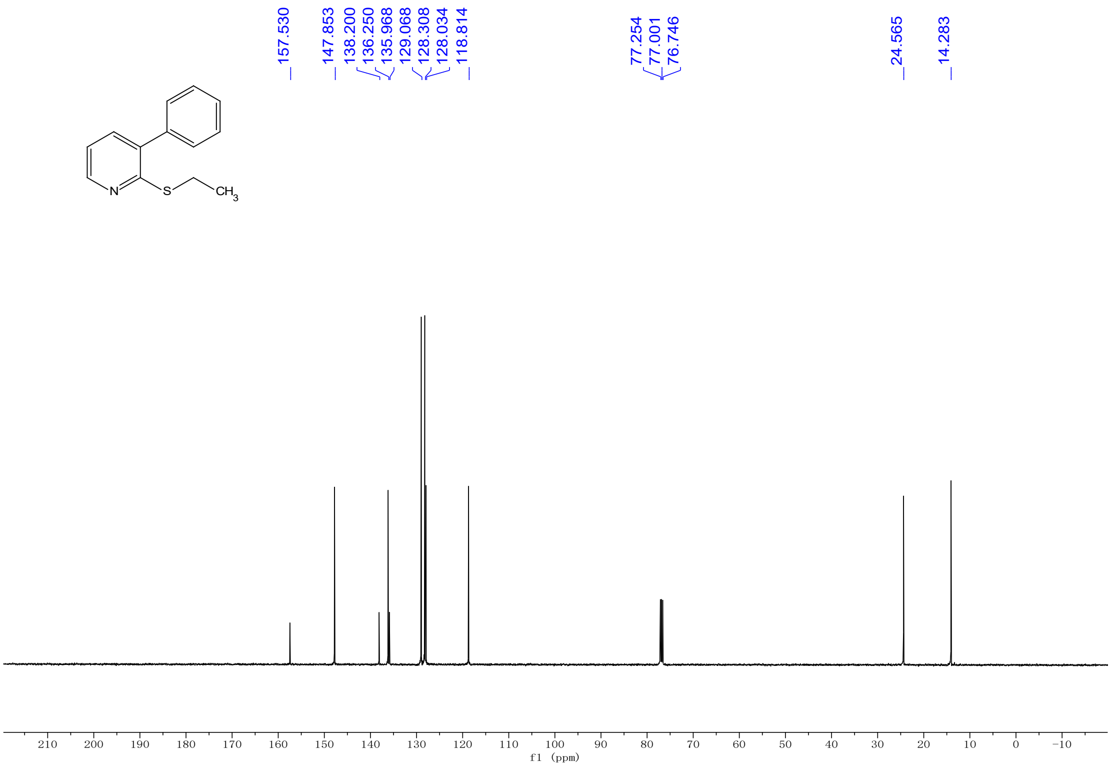

^1H NMR (500 MHz, $CDCl_3$) spectrum of compound 4o

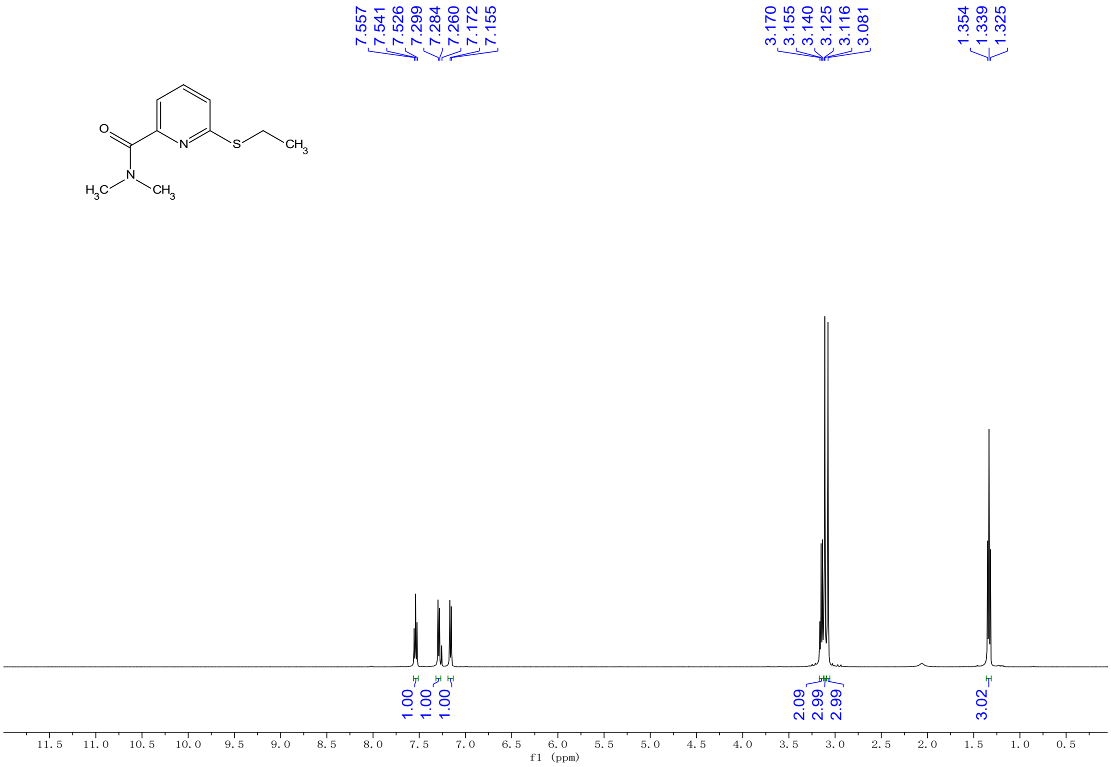

^{13}C NMR (125 MHz, $CDCl_3$) spectrum of compound 4o

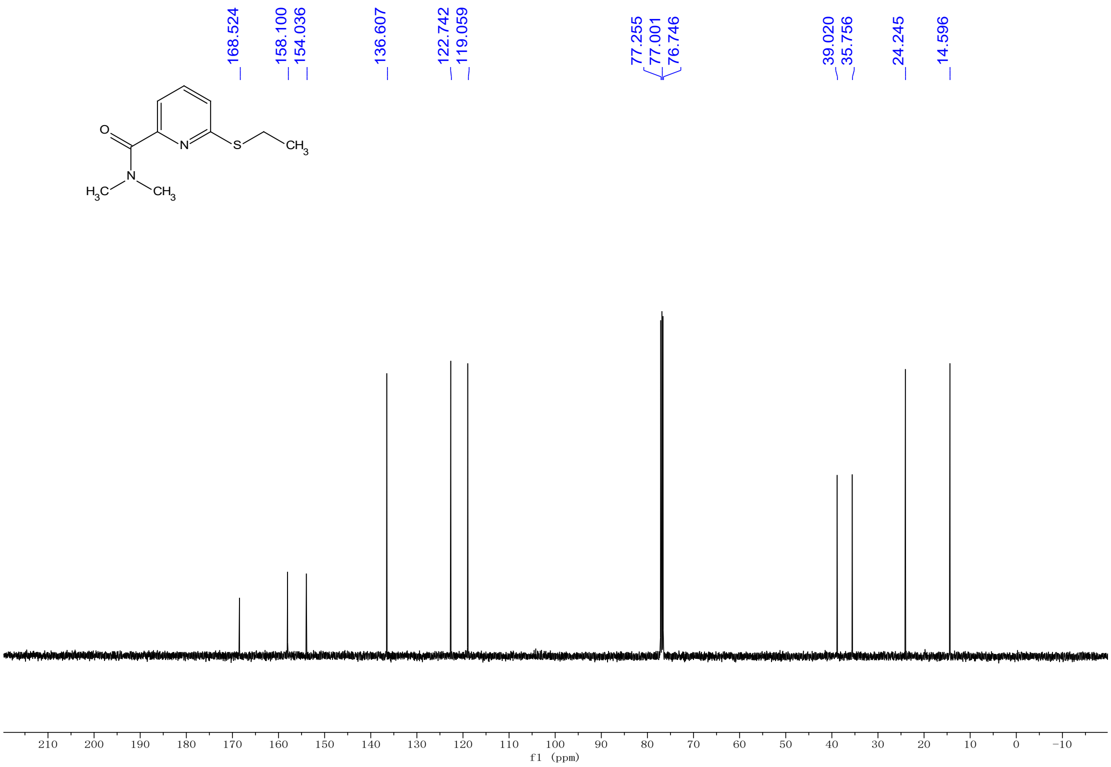

^{1}H NMR (500 MHz, $CDCl_3$) spectrum of compound 4p

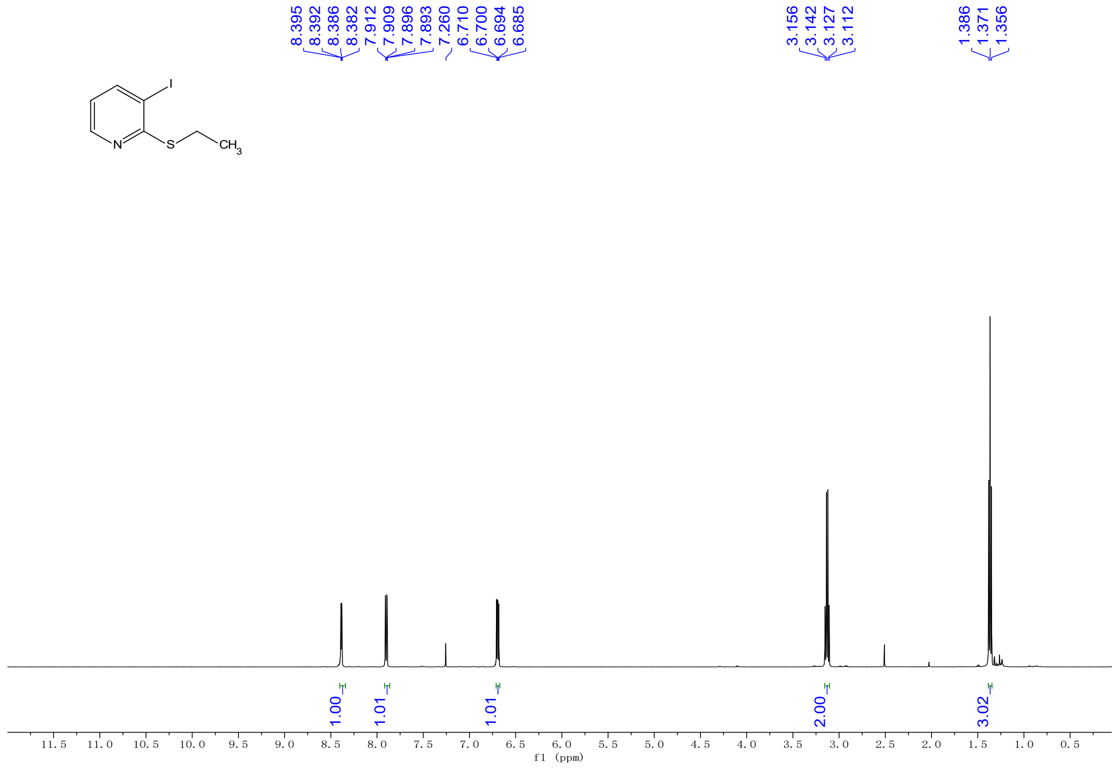

^{13}C NMR (125 MHz, $CDCl_3$) spectrum of compound 4p

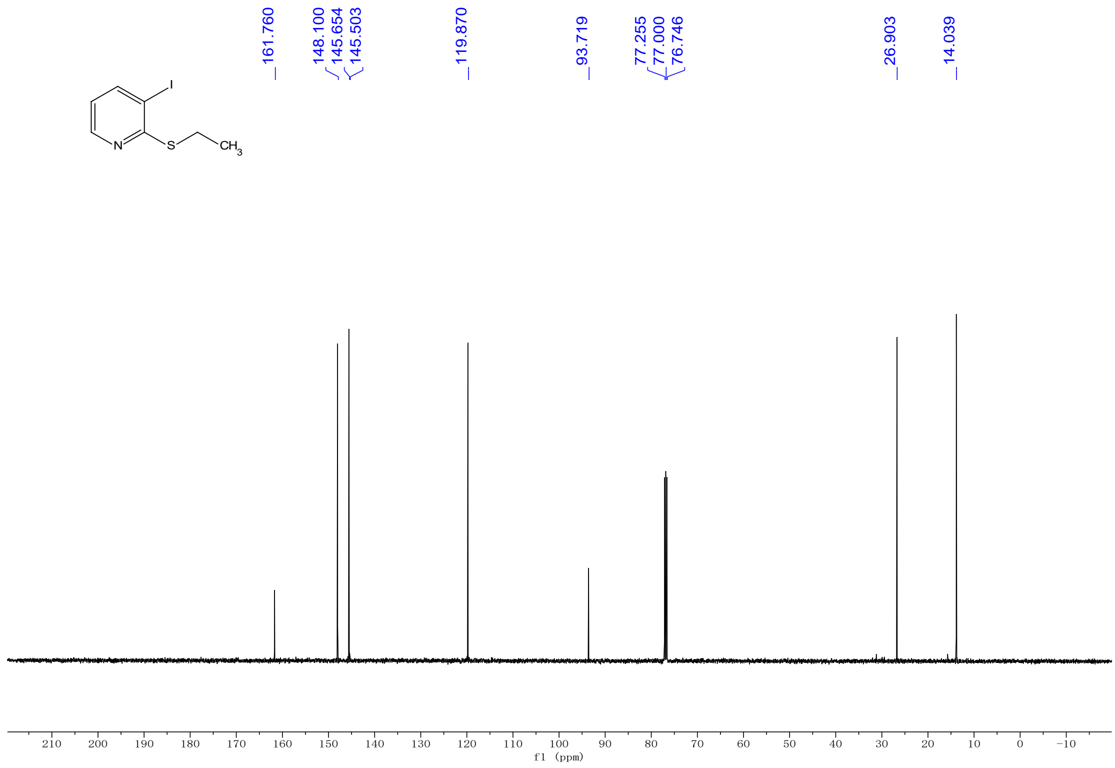

^{1}H NMR (500 MHz, $CDCl_3$) spectrum of compound 4q

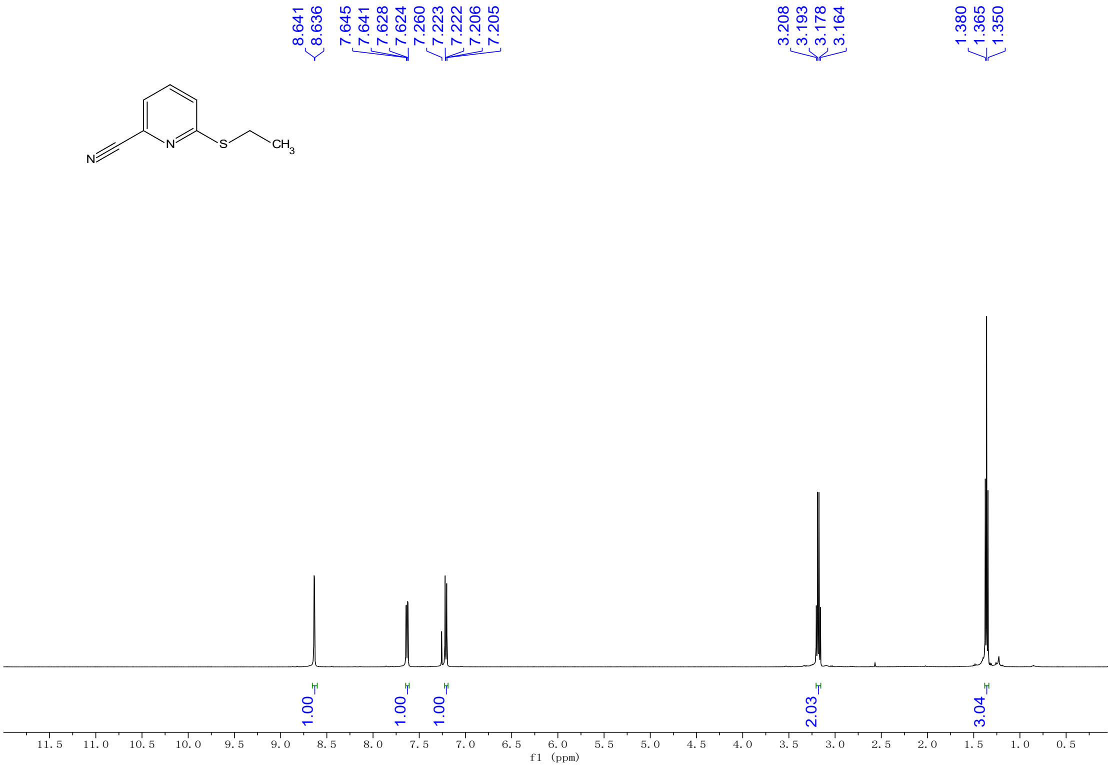

^{13}C NMR (125 MHz, $CDCl_3$) spectrum of compound 4q

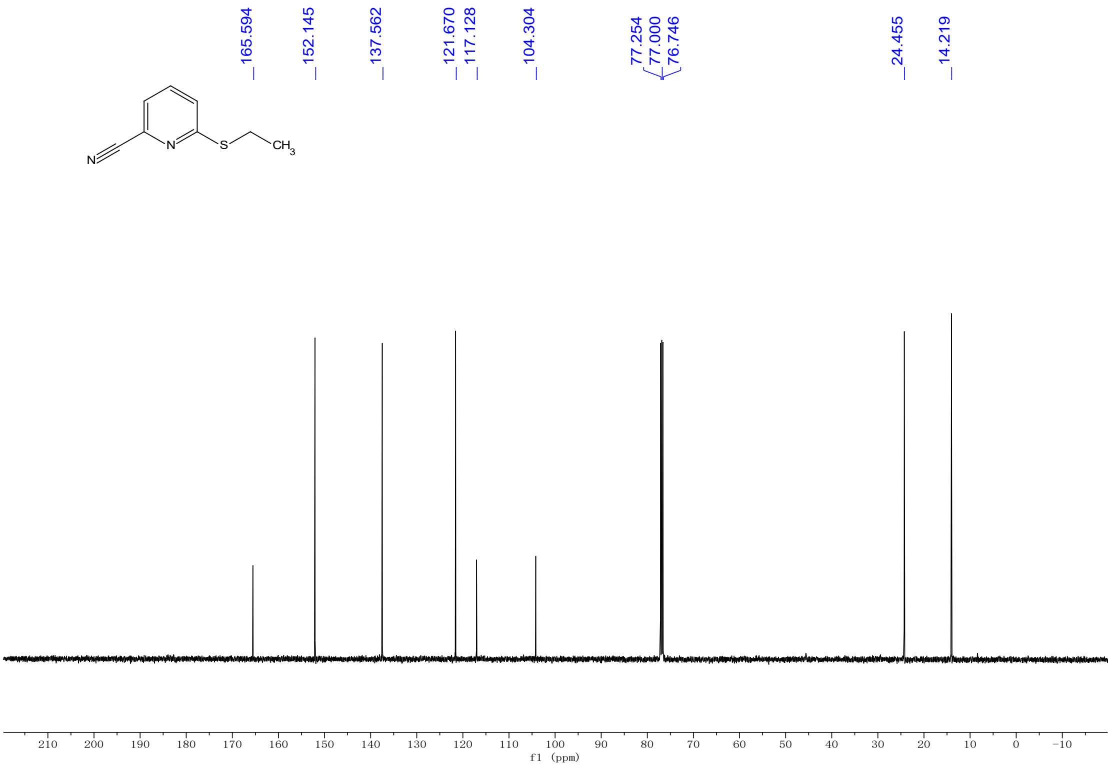

^1H NMR (500 MHz, $CDCl_3$) spectrum of compound 4r

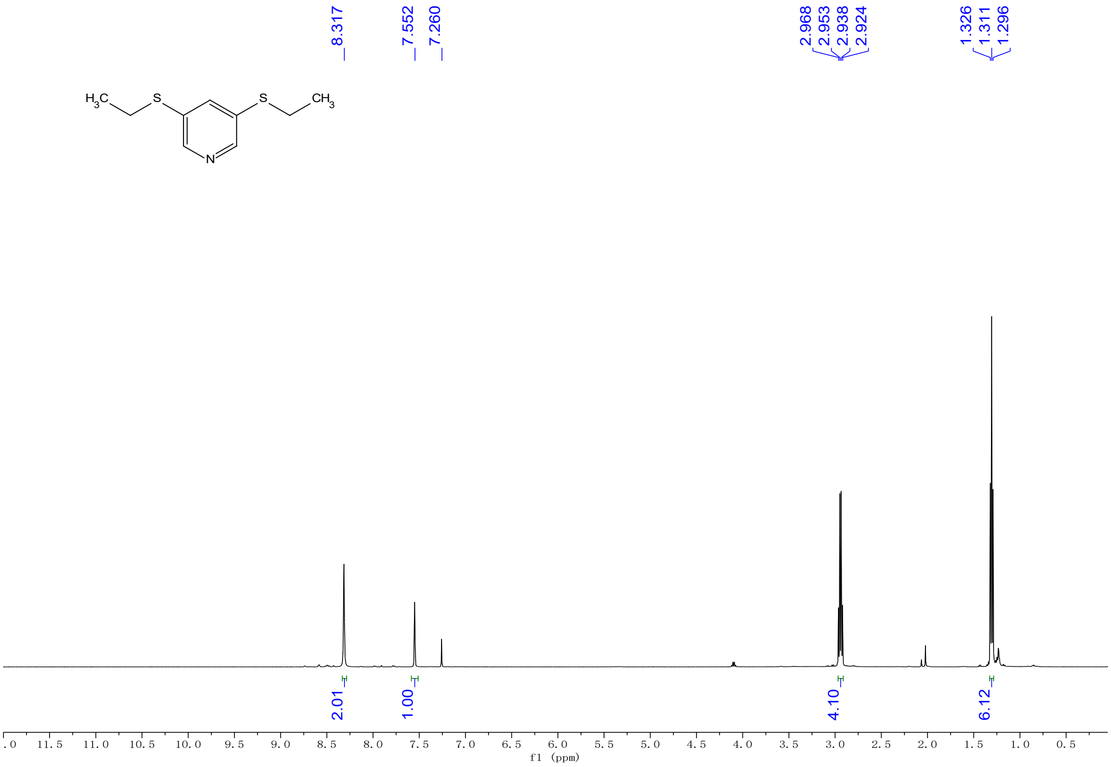

^{13}C NMR (125 MHz, $CDCl_3$) spectrum of compound 4r

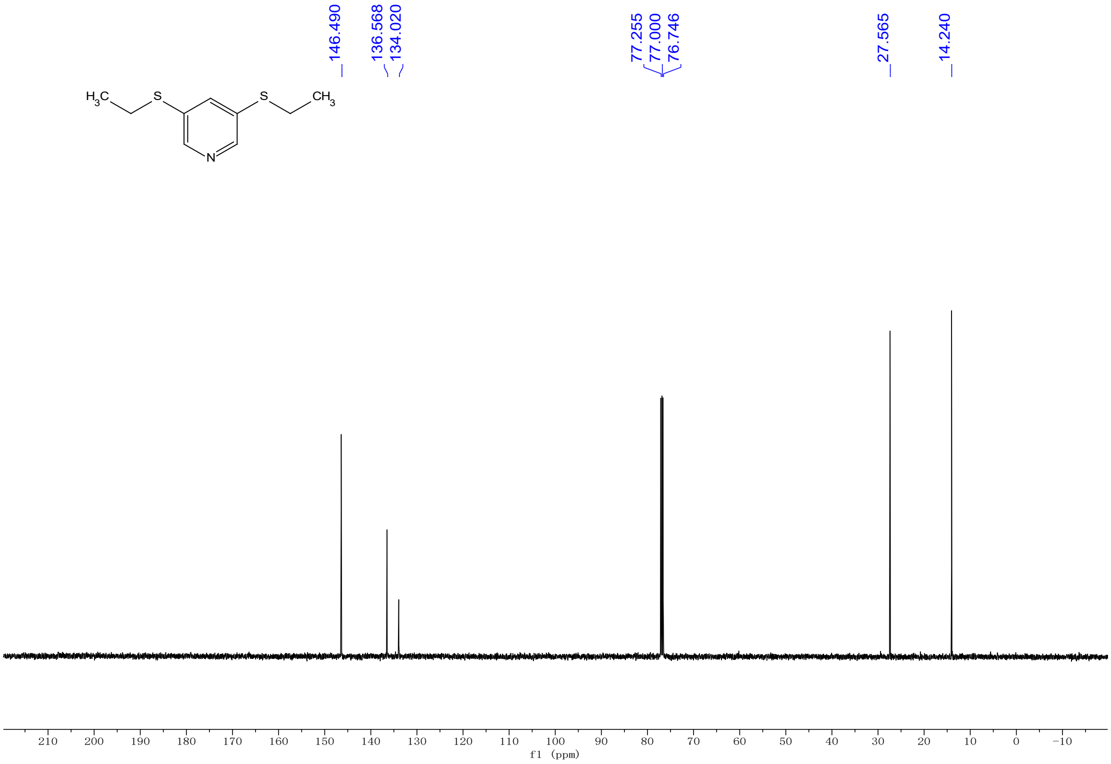

^{1}H NMR (500 MHz, $CDCl_3$) spectrum of compound 4s

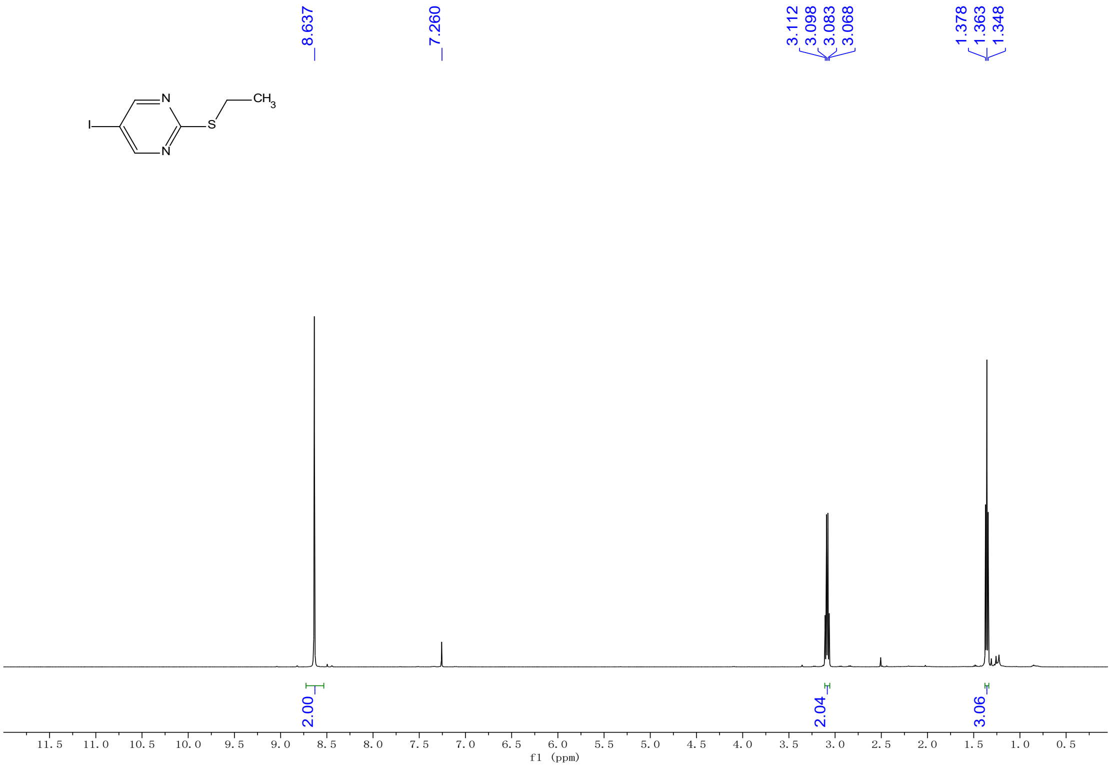

^{13}C NMR (125 MHz, $CDCl_3$) spectrum of compound 4s

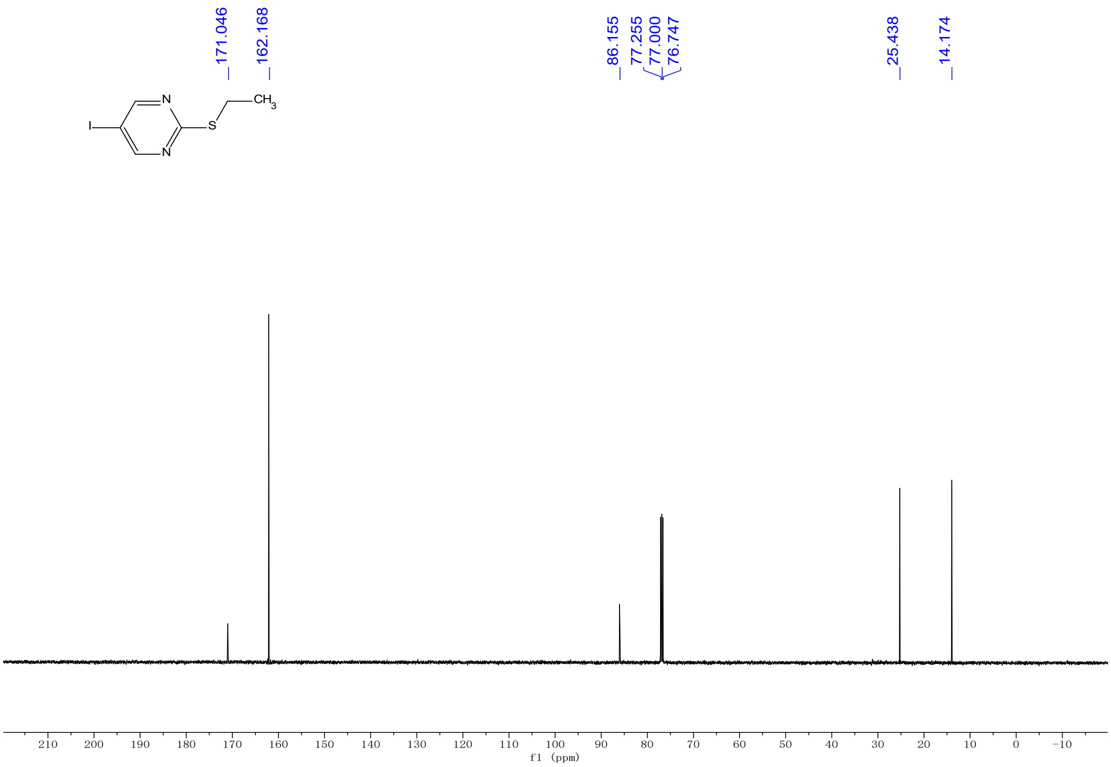

^{1}H NMR (500 MHz, $CDCl_3$) spectrum of compound 5a

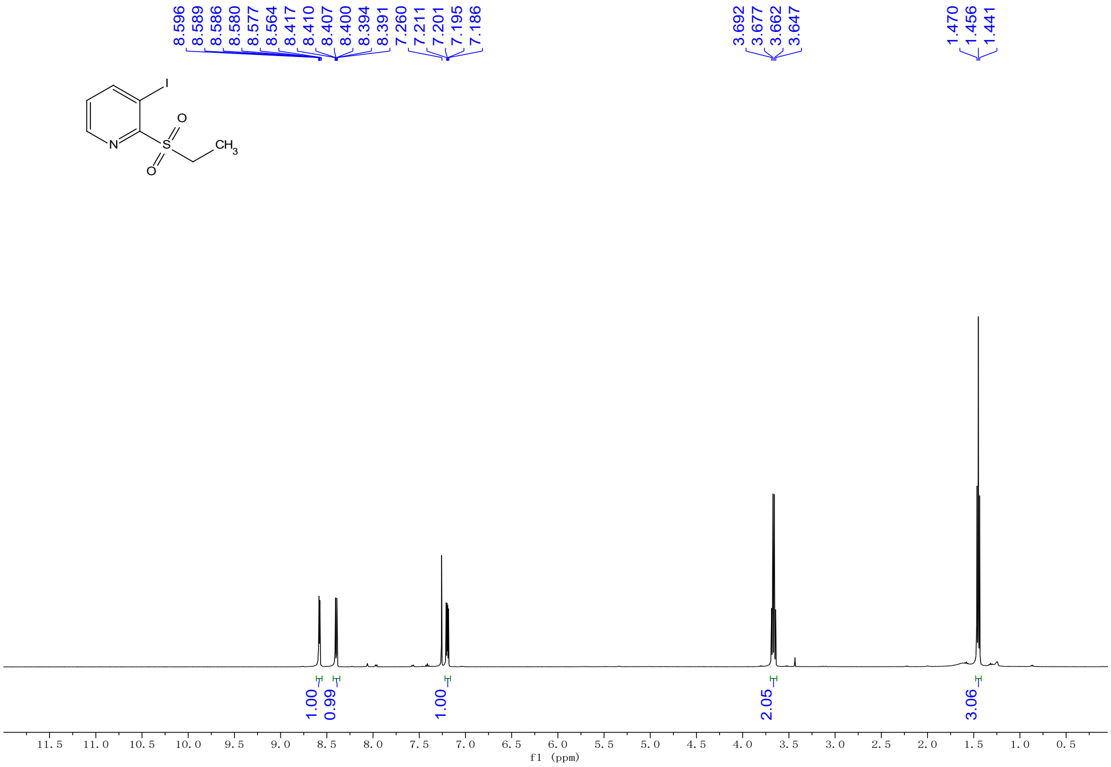

^{13}C NMR (125 MHz, $CDCl_3$) spectrum of compound 5a

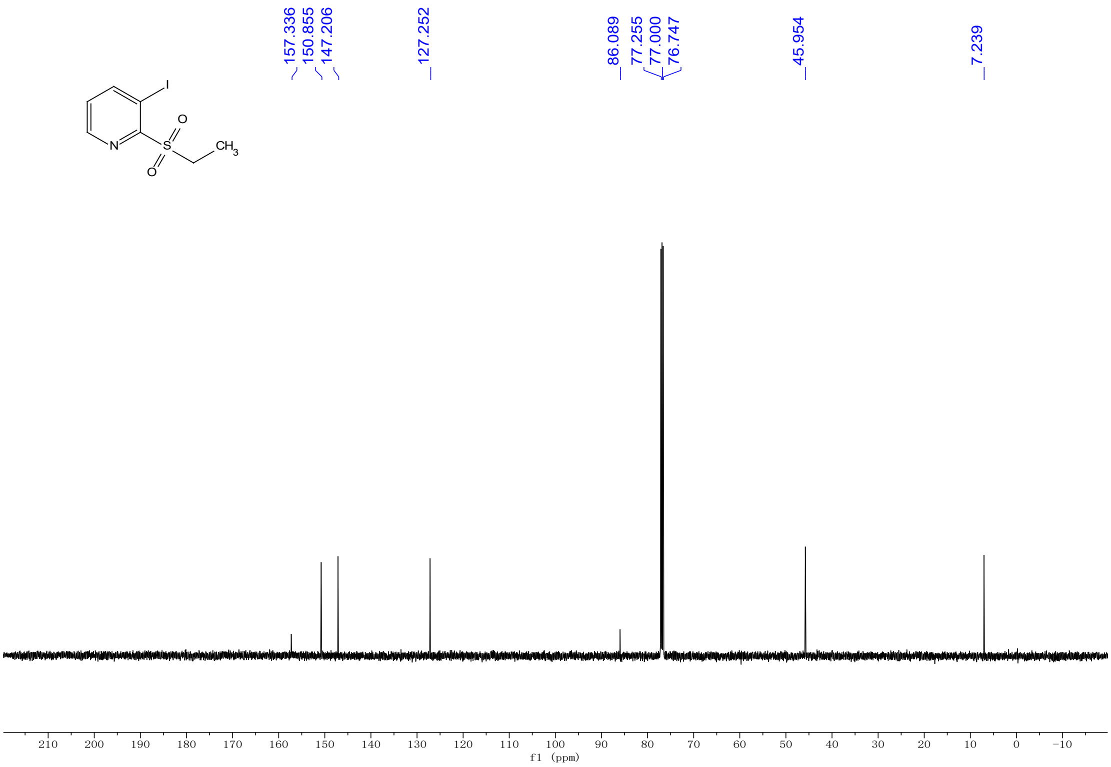

^{1}H NMR (500 MHz, $CDCl_3$) spectrum of compound 5b

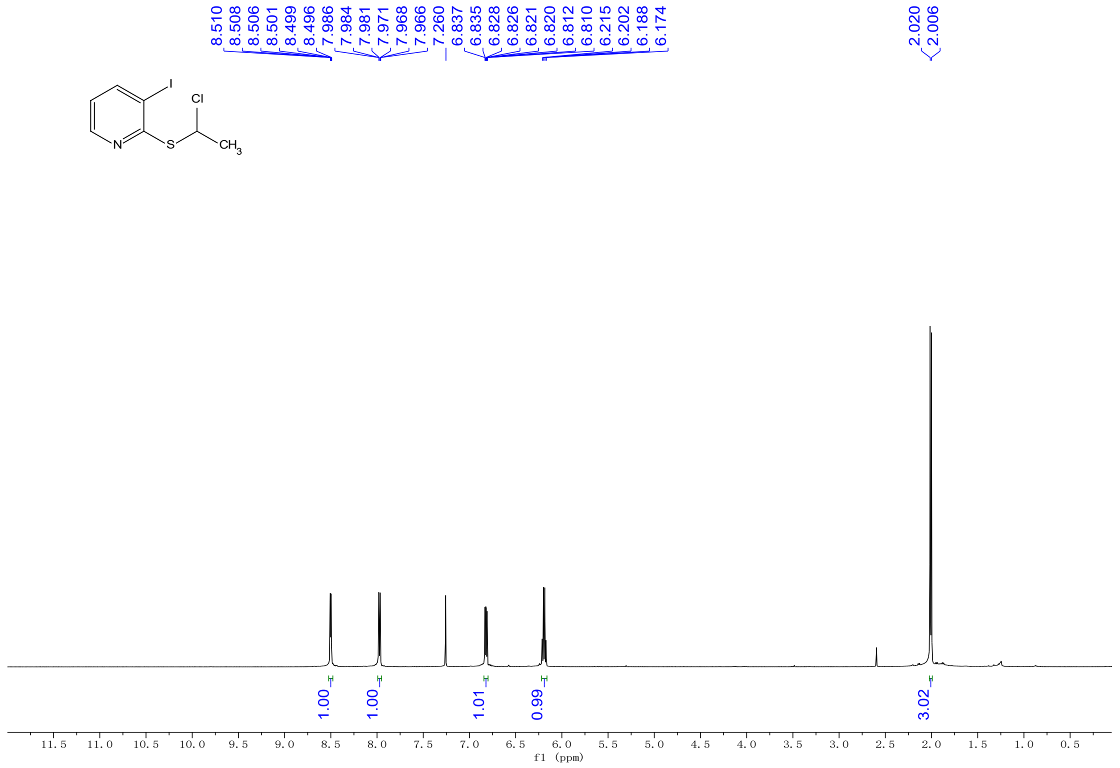

^{13}C NMR (125 MHz, $CDCl_3$) spectrum of compound 5b

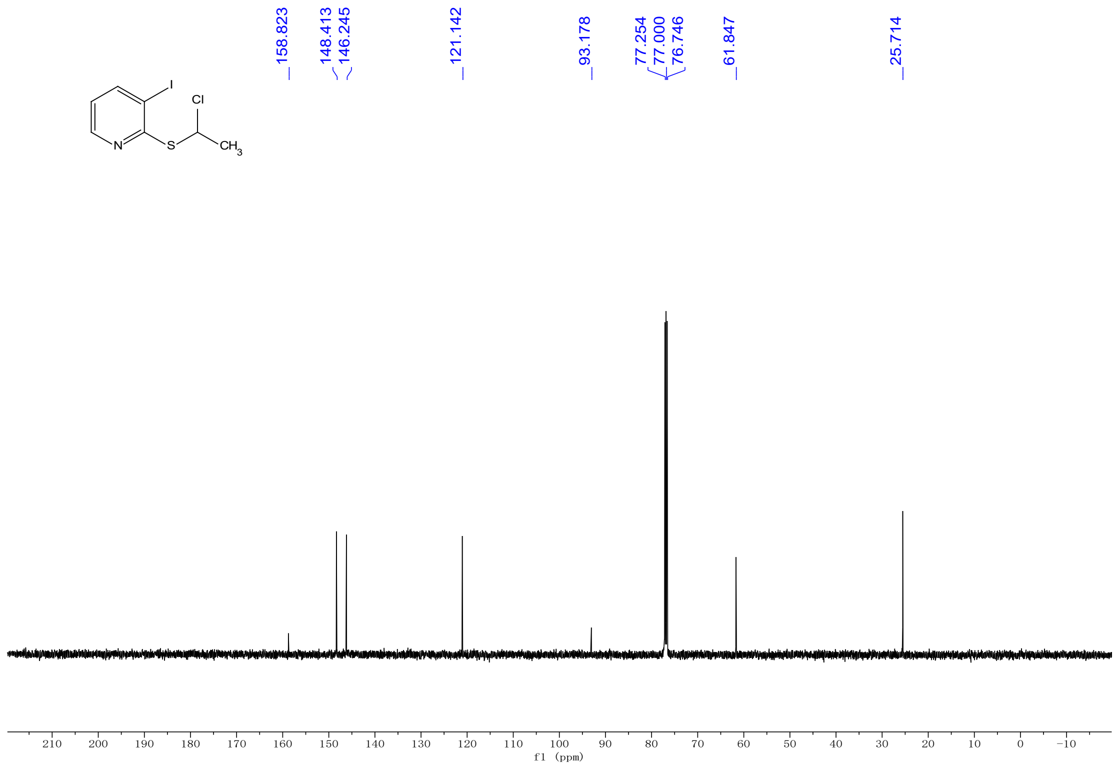

^{1}H NMR (500 MHz, $CDCl_3$) spectrum of compound 5c

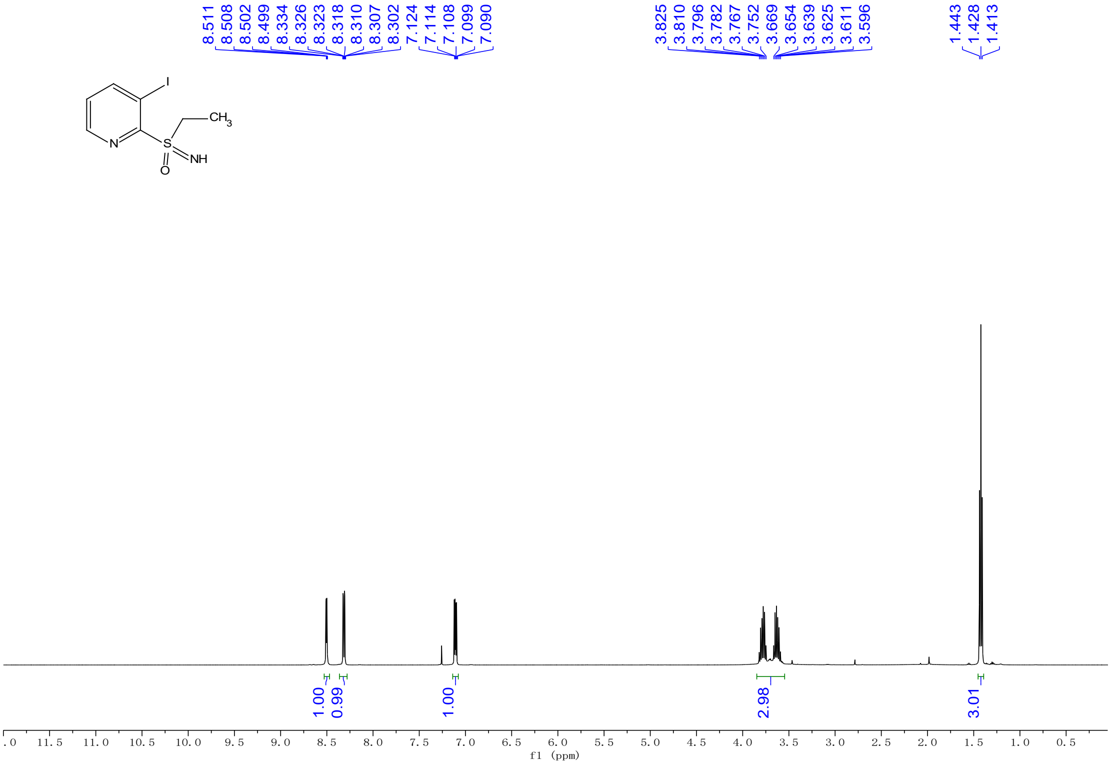

^{13}C NMR (125 MHz, $CDCl_3$) spectrum of compound 5c

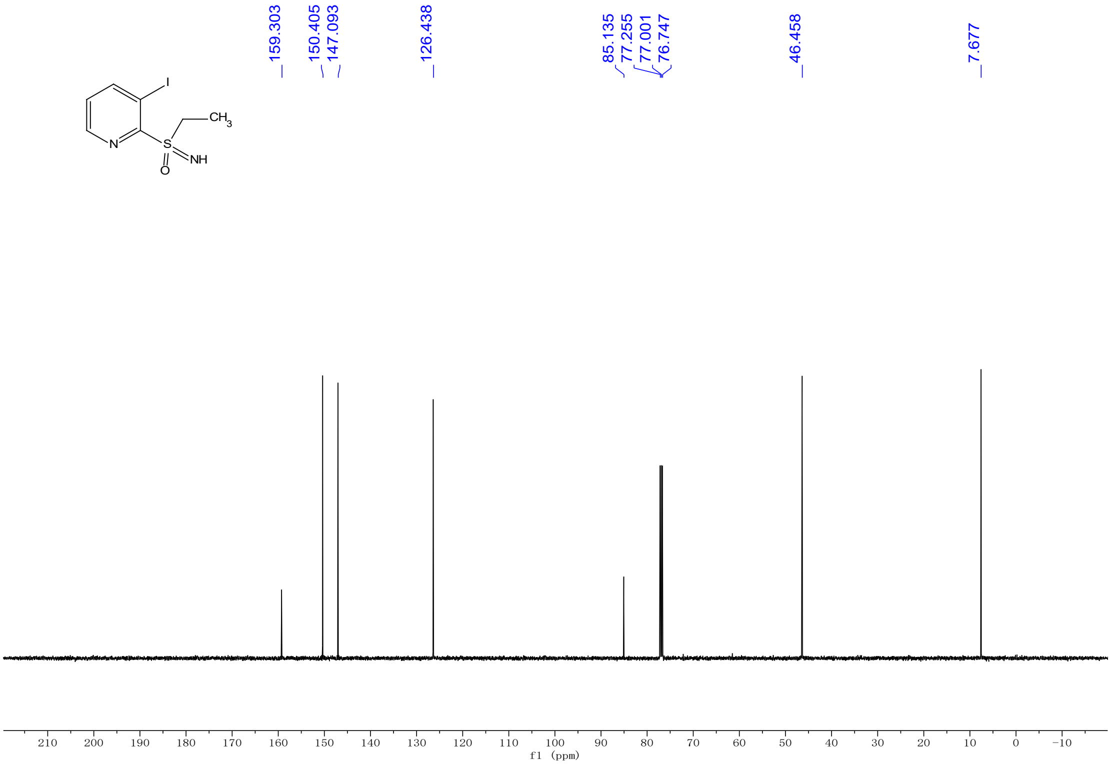

^{1}H NMR (500 MHz, $CDCl_3$) spectrum of compound 5e

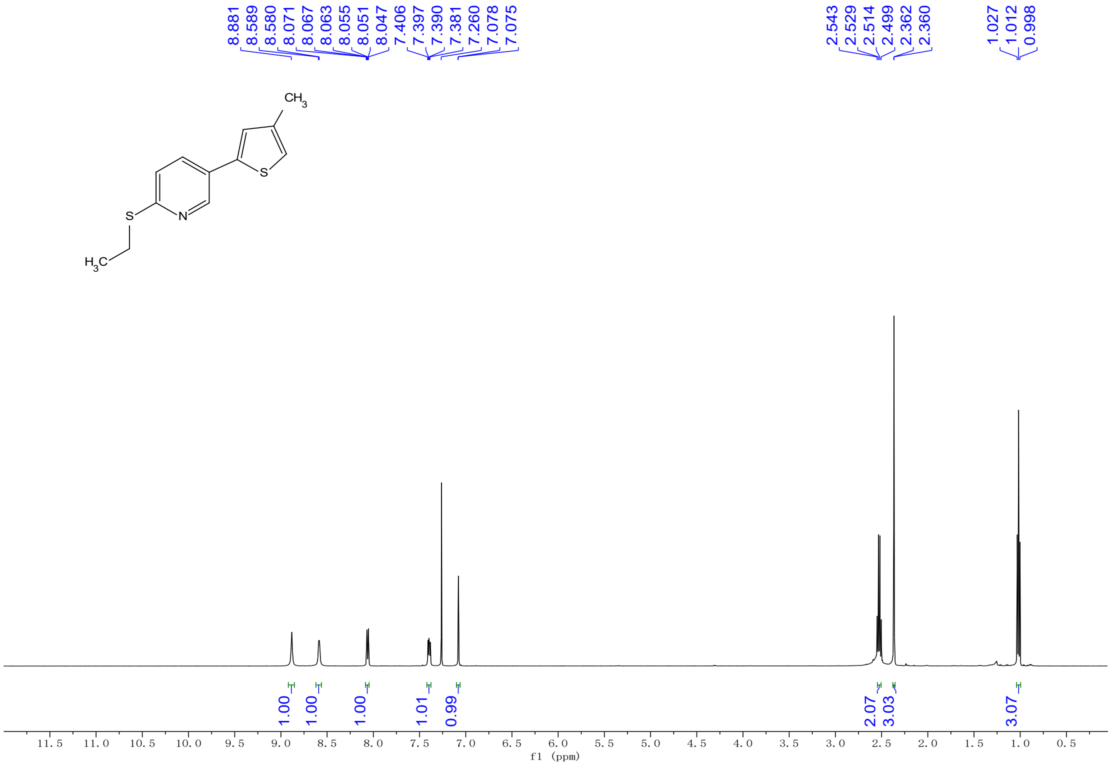

^{13}C NMR (125 MHz, $CDCl_3$) spectrum of compound 5e

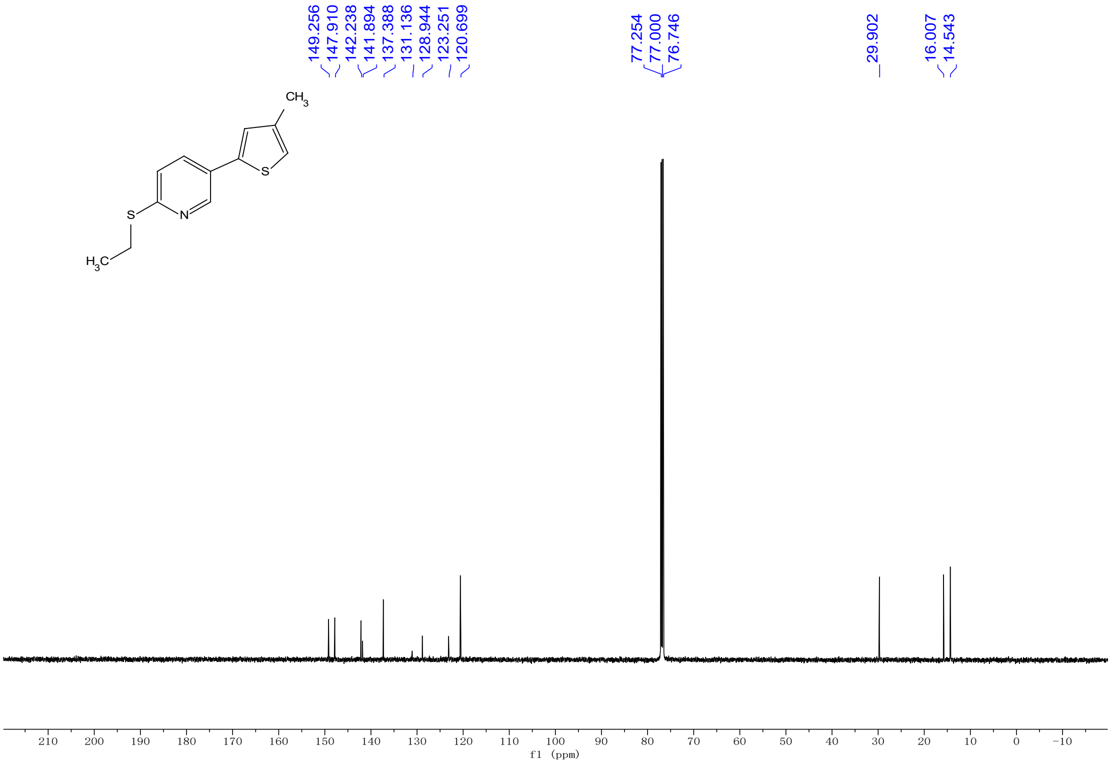

1H NMR (400 MHz, $CDCl_3$) spectrum of compound 5f

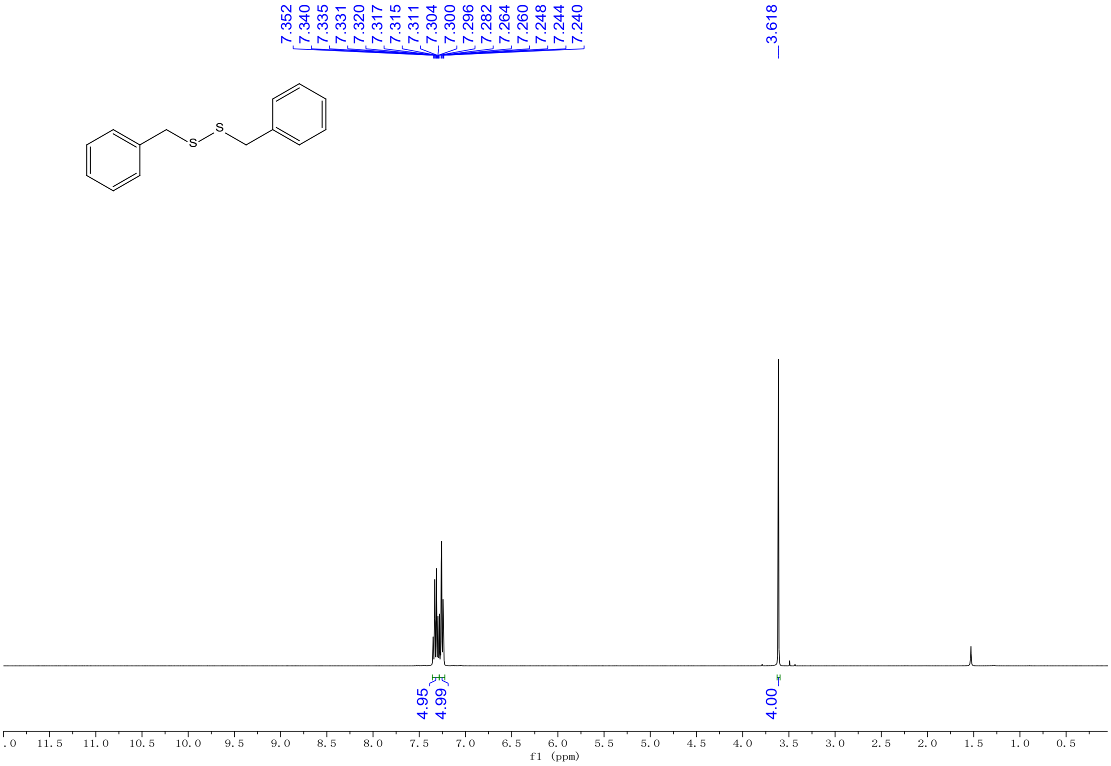

13C NMR (100 MHz, $CDCl_3$) spectrum of compound 5f

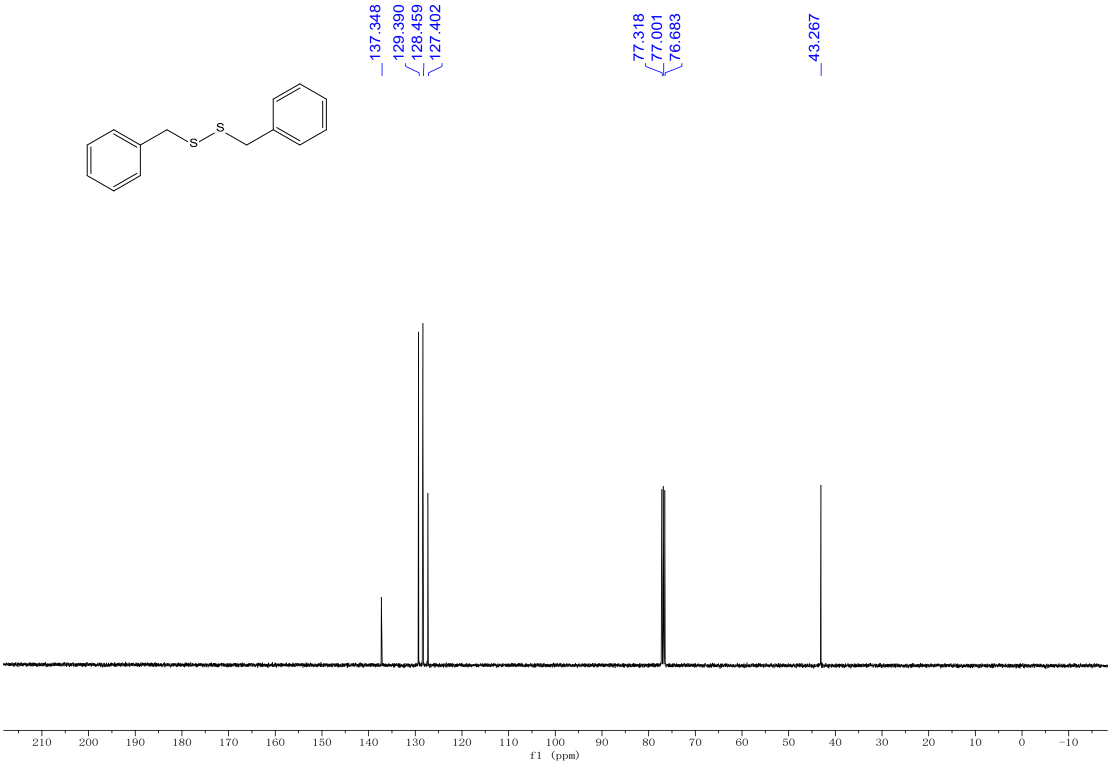

Supplement: Supplementary file 1 [file molecules-29-02485-s001.zip › molecules-3004043-supplementary.pdf]
